# PyBox–La(OTf)_3_-Catalyzed Enantioselective Diels–Alder Cycloadditions of 2-Alkenoylpyridines with Cyclopentadiene

**DOI:** 10.3390/molecules29132978

**Published:** 2024-06-22

**Authors:** Hao Wei, Yujie Zhang, Sanlin Jin, Ying Yu, Ning Chen, Jiaxi Xu, Zhanhui Yang

**Affiliations:** 1 Department of Organic Chemistry, College of Chemistry, Beijing University of Chemical Technology, Beijing 100029, China; w18439551277@126.com (H.W.); 2022201038@buct.edu.cn (Y.Z.); 2022201048@buct.edu.cn (S.J.); 2011500030@buct.edu.cn (N.C.); 2007500055@buct.edu.cn (J.X.); 2 China United Test & Certification Co., Ltd., Beijing 100088, China

**Keywords:** PyBox, rare earth, asymmetric catalysis, Diels–Alder cycloaddition, norbornene derivatives

## Abstract

The PyBox–La(OTf)_3_-catalyzed enantioselective Diels–Alder cycloaddition of 2-alk-2-enoylpyridines with cyclopentadiene is realized, producing enantiopure disubstituted norbornenes, which possess four contiguous stereocenters and are biologically relevant structures in up to 92:8 dr and 99:1 er.

## 1. Introduction

**Figure 1:**
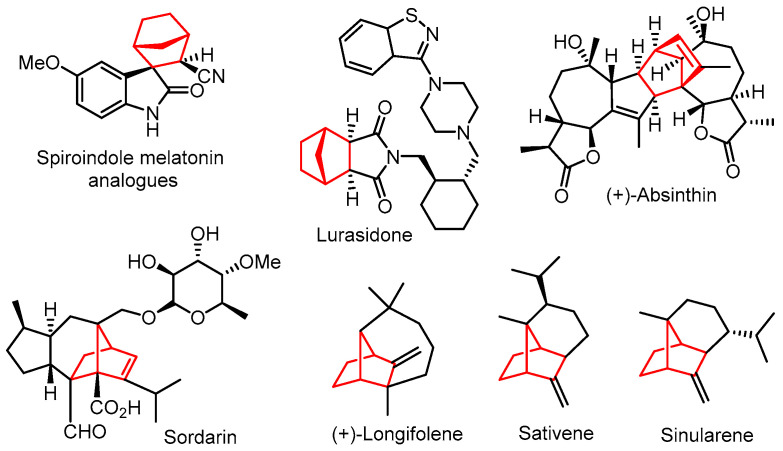
Representative bioactive compunds.

**Scheme 1:**
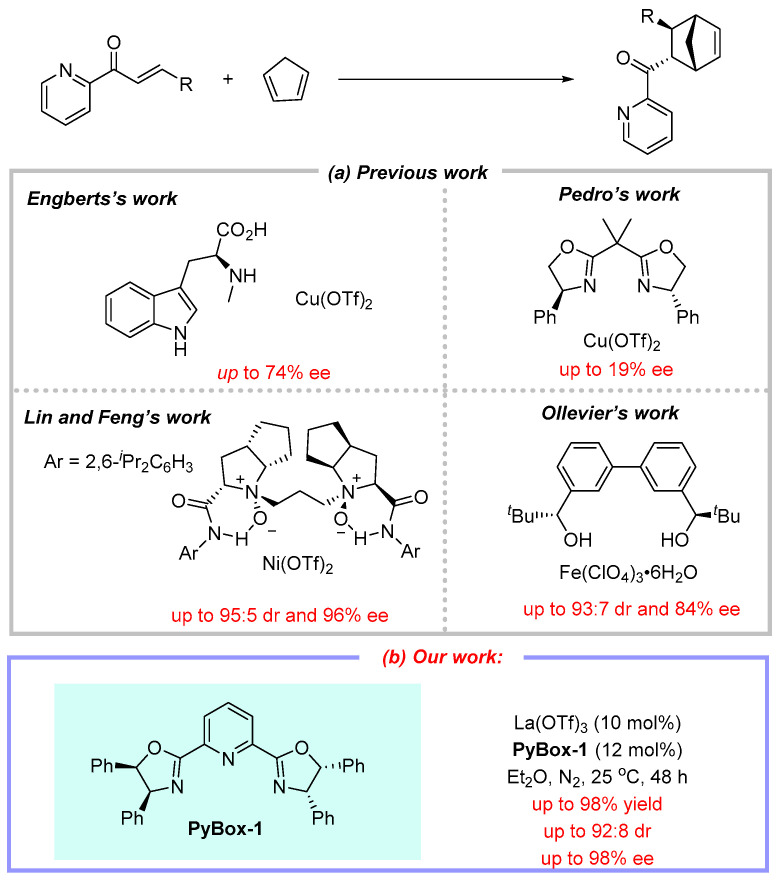
Previous work (**a**) and our work (**b**) on the asymmetric Diels–Alder cycloadditions of 2-alk-2-enoylpyridines with cyclopentadiene.

Enantioenriched norbornene scaffolds with multiple stereocenters are prevalent motifs in many pharmaceutically relevant and naturally occurring compounds (Figure 1) [1,2,3,4,5,6]. Therefore, the construction of such scaffolds constitutes an intriguing topic in the synthetic community. The catalytic asymmetric Diels–Alder cycloadditions of auxiliary-substituted alkenes with cyclopentadienes provide a straightforward and atom-economical access to the norbornene scaffolds, and are anticipated to produce good levels of chirality transfer and diastereocontrol by virtue of interactions between auxiliary groups and chiral catalysts [7]. In this regard, alkenoyl pyridines, which can chelate their pyridinyl nitrogen and carbonyl oxygen atoms to the metal center of a chiral catalyst, are demonstrated as robust dienophiles to undergo asymmetric Diels–Alder cycloadditions with cyclopentadienes, even in aqueous media (Scheme 1). Documented catalysts and ligands that are applicable to this useful type of reaction include Engberts’s chiral amino acid–Cu(II) system (74% ee) [8], Pedro’s chiral bis(oxazoline)–Cu(II) system (19% ee) [9], Lin and Feng’s *N*,*N*’-dioxide–Ni(II) system (up to 95:5 dr and 96% ee) [10] and Ollevier’s chiral bipyridine diol–Fe(III) system (up to 93:7 dr and 84% ee) [11]. In addition, numerous chiral artificial metalloenzymes and DNA-modified catalysts were also developed [12,13,14,15,16,17]. In spite of the above advances, new alternative catalytic systems are still in demand for effecting high levels of diastereo- and enantiocontrol, a purpose that was hitherto only satisfied by Lin and Feng’s *N*,*N*’-dioxide–Ni(II) catalysts [10].

The past decades have witnessed fruitful advancements on rare earth-catalyzed asymmetric Diels–Alder cycloadditions [18], which encouraged us to provide a rare-earth protocol for enantiocontrol. The key factor that underlies this protocol is the choice of a chiral ligand. Our previous research with didentate pyridyl imidazolines [19,20,21,22] led us to consider similar chirally modified nitrogen ligands. Considering the special coordination numbers of rare earth salts, we designed and synthesized several tridentate chiral nitrogen ligands and evaluated their chirality transfer ability in the rare earth-catalyzed Diels–Alder cycloadditions between 2-alk-2-enoylpyridines and cyclopentadiene. We eventually found that the combination of chiral pyridine-2,6-bisoxazolines and La(OTf)_3_ displays the best results in terms of diastereo- and enantiocontrol, producing enantioenriched norbornenes in good yields and enantiopurities (Scheme 1b).

## 2. Results and Discussion

We first optimized the reaction conditions using (*E*)-styrenoyl pyridine (**1a**) and cyclopentadiene (**2A**) as the model substrates. The selected attempts on ligand screening are listed in Table 1 (for more details, see Tables S1 and S2 in the Supplementary Materials). Pyridine-2,6-bisimidazoline ligands **PyBim-1** and **PyBim-2** produced **3aA** in 84:16 and 75:25 er, respectively [23,24,25]. In contrast, **3aA** was produced in 94:6 and 68:32 er values when the respective pyridine-2,6-bisoxazoline ligands **PyBox-1** and **PyBox-2** were used. The reaction with 1,10-phenanthroline-2-oxazoline (**PhenOx**) delivered **3aA** as a nearly racemic mixture. Apparently, **PyBox-1** produced the best result in terms of enantioselectivity. It deserves mention that similar ligands were also employed by Desimoni’s group in 2002 in the Diels–Alder *exo*-cycloadditions between alkenoyl-1,3-oxazolidin-2-ones and cyclopentadiene [26]. With this ligand, other lanthanum(III) salts such as LaCl_3_ and La(BF_4_)_3_ were evaluated, but nearly no enantioselectivity was observed. Thus, from the conditions listed in Table 1, entry 3 was selected as the optimal one.

**Figure 2:**
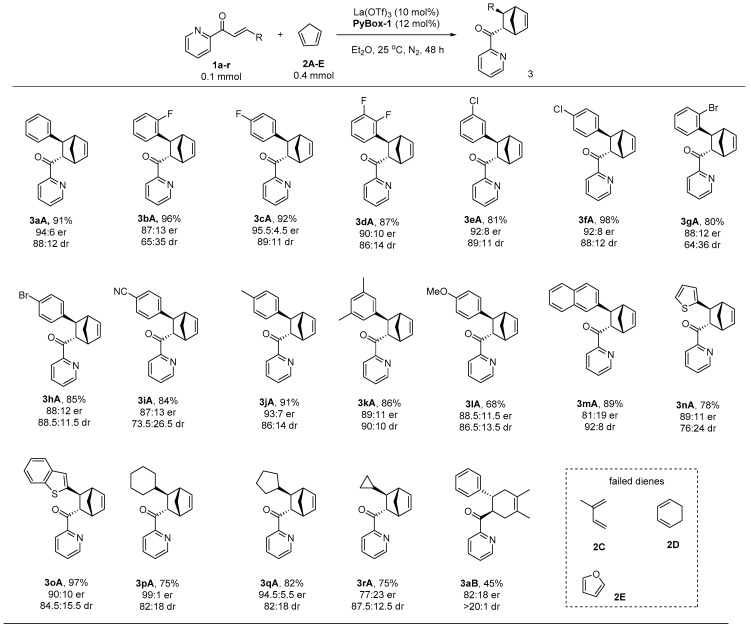
Scope of substrates.

With the optimal conditions at hand, a number of dienophiles, that is, 2-alk-2-enoylpyridines **1a–r**, were reacted with cyclopentadiene. The results regarding enantio- and diastereoselectivities (*endo*/*exo*) are summarized in Figure 2. 2-Alk-2-enoylpyridines bearing electronically diverse aryl substituents were first screened. In addition to (*E*)-styrenoylpyridine (**1a**), the halogen-substituted dienophiles **1b–1h** all productively gave desired products **3bA–3hA** in 80–98 yields, 64:36–89:11 drs, and most importantly, 87:13–95.5:4.5 ers. The highest enantioselectivity (95.5:4.5 er) was observed in the reaction producing **3cA**. Strongly electron-withdrawing cyano-substituted dienophile **1i**, as well as those with electron-donating alkyl and methoxy substituents, produced desired products **3iA–3lA** in 87:13–93:7 ers with varying diastereoselectivities and yields. Dienophiles with heteroaryls and fused aryls were also amenable, and the corresponding products were procured in 81:19 to 90:10 er. The alkyl-substituted 2-alk-2-enoylpyridines **1p–1r** were also tested under the optimal conditions. Dienophiles **1p** and **1q** with more steric hindrance delivered products **3pA** and **3qA** in excellent 99:1 and 95:5 ers, respectively, whilst dienophile **1r** with less steric cyclopropyl only afforded moderate enantioselectivity of 77:23. Acyclic dienes were also examined. 2,3-Dimethylbuta-1,3-diene (**2B**) afforded **3aB** at a 45% yield with 82:18 er and >20:1 dr. However, other attempted dienes, including 2-methylbuta-1,3-diene (**2C**), cyclohexa-1,3-diene (**2D**) and furan (**2E**), did not react with **1a**.

**Scheme 2:**
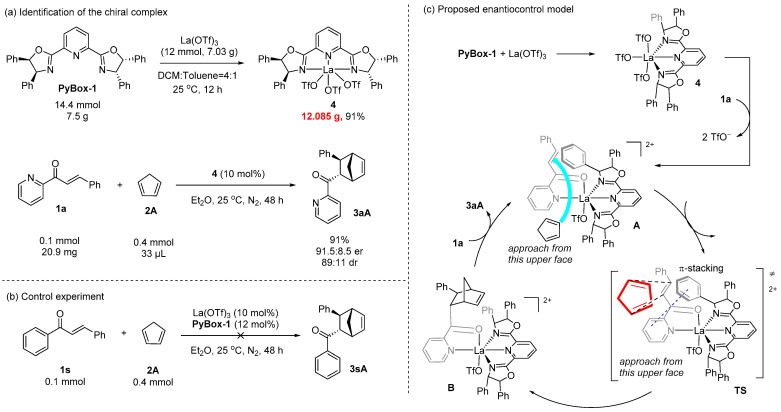
Proposed mechanism.

Mechanistic studies were performed (Scheme 2). Mixing **PyBox-1** with La(OTf)_3_ afforded a stable complex **4** (Scheme 2a). The complex can even be prepared in a decagram scale at a 91% yield. In the presence of 10 mol% of **4**, the cycloaddition of **1a** and **2A** produced **3aA** at a 91% yield, 91.5:8.5 er and 89:11 dr, a result almost identical with that obtained with separately added **PyBox-1** and La(OTf)_3_. In the control experiment (Scheme 2b), the reaction of *E*-chalcone (**1s**) with cyclopentadiene (**2A**) did not yield the desired product 3sA, highlighting the essence of the pyridine auxiliary and nitrogen coordination to the metal center. Based on the above results, a plausible enantiocontrol model is proposed (Scheme 2c). Ligand exchange between **A** and substrate **1a** afforded chiral complex **B**. The C_2_-asymmetry created a chiral pocket that allowed cyclopentadiene to approach the dienophile from the southwest upper face of **B**. A π-stacking interaction between the pyridinyl ring and the upward phenyl ring in proximity to the nitrogen atom was proposed to stabilize the transition state **TS** [27]. Once product **3a** was formed, it was still coordinated with the rare earth metal. Ligand exchange between **C** and **1a** ultimately produced **3aA** and opened a new catalytic cycle.

**Scheme 3:**
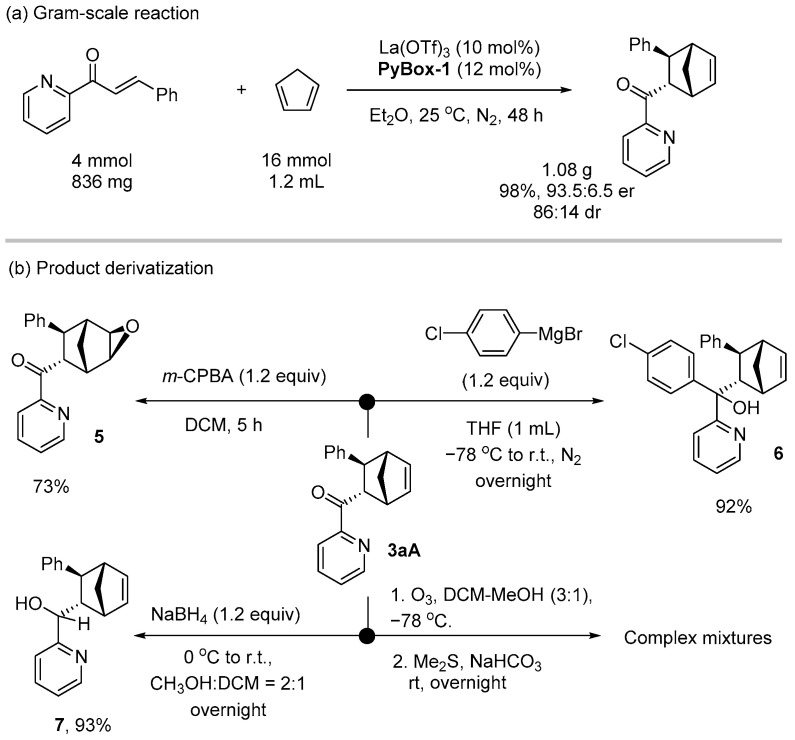
Gram-scale reaction and product derivatization.

The **PyBox**-La(OTf)_3_-catalyzed cycloaddition is featured with good scalability (Scheme 3a). In the gram-scale reaction, **3aA** was obtained in 93.5:6.5 er, 86:14 dr with a 98% yield. The obtained difunctionalized norbornene **3aA** possesses two reactive chemical handles to undergo further manipulations (Scheme 3b). Epoxidation of the endocyclic C=C bond yielded epoxide **5**, with two new stereocenters generated at a 73% yield, leaving the electron-rich pyridine nitrogen intact. The facial selectivity was assigned according to the empirical rule proposed by Brown and coworkers [27]. Grignard addition to the carboxyl group led to a tertiary alcohol **6** at a 92% yield, whereas the NaBH_4_ reduction produced a secondary alcohol **7** at a 93% yield. However, the ozonolysis of **3aA** led to complex mixtures with all the starting material consumed.

## 3. Materials and Methods

### 3.1. Materials and Instruments

Unless otherwise noted, all materials were purchased from commercial suppliers. Dichloromethane (DCM) and dichloroethane (DCE) were refluxed over CaH_2_; tetrahydrofuran (THF) and toluene (PhMe) were refluxed over lithium aluminum hydride. The solvents were freshly distilled prior to use. Column chromatography was performed on silica gel (normal phase, 200–300 mesh) from Anhui Liangchen Silicon Material Co., Ltd. (Lu’An, China), with petroleum ether (PE, bp. 60–90 °C) and ethyl acetate (EA) as eluent. Reactions were monitored using thin-layer chromatography (TLC) on GF_254_ silica gel plates (0.2 mm) from Anhui Liangchen Silicon Material Co., Ltd. The plates were visualized via UV light using other TLC stains (1% potassium permanganate in water; 10 g of iodine absorbed on 30 g of silica gel). ^1^H and ^13^C NMR spectra were recorded on a Bruker 400 MHz spectrometer, usually in CDCl_3_ as an internal standard, and the chemical shifts (*δ*) were reported in parts per million (ppm). Multiplicities are indicated as s (singlet), d (doublet), t (triplet), q (quartet), dd (double doublet), m (multiplet) and b (broad). Coupling constants (*J*) are reported in Hertz (Hz). HRMS measurements were carried out on an Agilent LC/MSD TOF mass spectrometer. The enantiomeric excesses were determined via HPLC analysis using Agilent Technologies 1260 Infinity equipment, and the employed chiral stationary phase column are specified in the individual experiment by comparing the enantiomeric samples with the appropriate racemic mixtures. 

Substrate **1a–r** were prepared according to Caggiano’s study [28]. The nuclear magnetic spectra of **1a** to **1c**, **1e** to **1p** and **1r** are in agreement with the predecessors. **PyBim-2** [23], and **PhenOx-1** [29] were prepared according to published procedures.

### 3.2. General Procedure for Reduction of 2-Alkenoylpyridines

To a solution of ketone (5 mmol) and aldehyde (5.5 mmol) in ethanol (15mL), aqueous sodium hydroxide solution (5 mL, 2.5 M) was added dropwise at 0 °C. The reaction mixture was further stirred at room temperature until the completion of reaction (detected by TLC). Then, the reaction mixture was filtered and washed with ethanol–water solution (1/1, *v*/*v*) and dried. The precipitate was recrystallized in methanol to obtain pure unsaturated ketone products.

#### 3.2.1. (*E*)-3-(2,3-Difluorophenyl)-1-(pyridin-2-yl)prop-2-en-1-one (**1d**)

Yellow solid, 1.018 g, yield 83%, R*_f_* = 0.5 (PE/EA = 5:1, *v/v*). **^1^H NMR** (400 MHz, CDCl_3_) δ 8.75 (ddd, *J* = 4.8, 1.7, 0.9 Hz, 1H), 8.38 (d, *J* = 16.3 Hz, 1H), 8.19 (dt, *J* = 7.8, 1.1 Hz, 1H), 8.04 (d, *J* = 16.3 Hz, 1H), 7.89 (td, *J* = 7.7, 1.7 Hz, 1H), 7.52 (dddd, *J* = 15.1, 7.5, 4.5, 1.5 Hz, 2H), 7.23–7.09 (m, 2H). **^13^C NMR** (101 MHz, CDCl_3_) δ 189.2, 153.8, 148.9, 137.0, 135.5, 127.1, 125.5 (d, *J* = 8.7 Hz), 124.3 (d, *J* = 5.6 Hz), 124.2 (t, *J* = 5.8, 11.4 Hz), 123.8, 123.0, 118.7 (d, *J* = 17.3 Hz), 118.6. **^19^F NMR** (376 MHz, CDCl_3_) δ −137.72, −139.62. **HRMS** (ESI): *m*/*z* [M+H]^+^ calculated for C_14_H_10_F_2_NO^+^ 246.0725, found 246.0732.

#### 3.2.2. (*E*)-3-Cyclopentyl-1-(pyridin-2-yl)prop-2-en-1-one (**1q**)

Colorless oil, 532.6 mg, yield 53%, R*_f_* = 0.65 (PE/EA = 5:1, *v/v*). **^1^H NMR** (400 MHz, CDCl_3_) δ 8.70 (ddd, *J* = 4.8, 1.7, 0.9 Hz, 1H), 8.12 (dt, *J* = 7.9, 1.1 Hz, 1H), 7.84 (td, *J* = 7.7, 1.7 Hz, 1H), 7.59 (dd, *J* = 15.6, 1.1 Hz, 1H), 7.45 (ddd, *J* = 7.5, 4.8, 1.3 Hz, 1H), 7.22 (dd, *J* = 15.6, 8.2 Hz, 1H), 2.77 (h, *J* = 8.1, 7.6 Hz, 1H), 1.96–1.87 (m, 2H), 1.75–1.48 (m, 6H). **^13^C NMR** (101 MHz, CDCl_3_) δ 189.6, 154.6, 154.2, 148.7, 136.8, 126.6, 122.8, 122.4, 43.5, 32.6, 25.4. **HRMS** (ESI): *m*/*z* [M+H]^+^ calculated for C_13_H_16_NO^+^ 202.1226, found 202.1234.

### 3.3. General Procedure for Reduction of ***PyBim-1*** and ***PyBox***


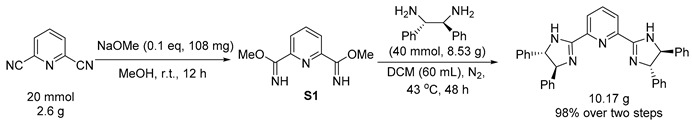


This was prepared according to Beller’s procedure, with slight modification [24]. The ^1^H and ^13^C NMR spectra are in agreement with those reported [24]. ***Procedure***: To an oven-dried round-bottom flask equipped with a magnetic stirring bar was added 2,6-pyridinedicarbonitrile (2.6 g, 20 mmol), sodium methoxide (108 mg, 2 mmol), and methanol (40 mL). The mixture was stirred at ambient temperature. After 12 h, the solution was transferred to an oven-dried flask and then quenched with acetic acid (0.24 mL). The solvent was evaporated under reduced pressure to obtain product **S1** (>99% yield).

To an oven-dried round-bottom flask equipped with a magnetic stirring bar was added **S1** and (1*S*, 2*S*)-1,2-diphenylethane-1,2-diamine (8.53 g, 40 mmol). The flask was sealed immediately with a rubber stopper and protected with a nitrogen balloon by evacuation-backfill operations (repeated three times). Dry dichloromethane (DCM, 60 mL) was injected to the flask via a syringe, and the reaction mixture was kept at 43 °C for 48 h. The solvent was evaporated under reduced pressure, and the crude mixture was subjected to column chromatography (PE:EA:Et_3_N = 20:10:1) on silica gel to afford the corresponding crude product. Final purification by recrystallization from ethyl acetate and petroleum ether afforded **PyBim-1** in 98% yield (10.17 g) as a white solid.


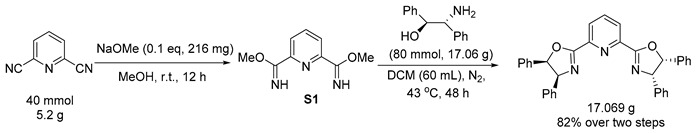


This was prepared according to Fokin’s procedure, with slight modification [30]. The ^1^H and ^13^C NMR spectrum and data are in agreement with those reported. ***Procedure***: To an oven-dried round-bottom flask equipped with a magnetic stirring bar was added 2,6-pyridinedicarbonitrile (5.2 g, 40 mmol), sodium methoxide (216 mg, 4 mmol), and methanol (80 mL). The reaction mixture was stirred at ambient temperature. After 12 h, the solution was transferred to an oven-dried flask and quenched with acetic acid (0.48 mL); the solvent was evaporated under reduced pressure to obtain product **S1** (>99% yield). To an oven-dried round-bottom flask equipped with a magnetic stirring bar was added **S1** and (1*S*, 2*R*)-2-amino-1,2-diphenylethan-1-ol (17.06 g, 80 mmol). The flask was sealed immediately with a rubber stopper and protected with a nitrogen balloon by evacuation-backfill operations (repeated three times). Dry DCM (120 mL) was injected to the flask via a syringe, and the reaction mixtures was kept at 43 °C. After 48 h, the solvent was evaporated, and the remaining mixture was solidified by MeOH and washed with MeOH and EA to obtain **PyBox-1** (17.069 g, 82%) as a white solid.


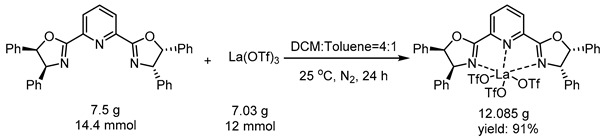


***Procedure***: To an oven-dried round-bottom flask equipped with a magnetic stirring bar was successively added **PyBox-1** (7.5 g, 14.4 mmol) and La(OTf)_3_ (7.03 g, 12 mmol). The flask was sealed immediately with a rubber stopper and protected with a nitrogen balloon by evacuation-backfill operations (repeated three times). A mixture of DCM and toluene (*v*/*v* = 4:1) (120 mL) was injected to the tube via a syringe, and the mixture was stirred for about 24 h. The solvent was evaporated under reduced pressure. The crude mixture was washed with Et_2_O to obtain the product (12.085 g, 91%) as a white solid.

### 3.4. General Procedure for Reduction of Chiral Product ***3***


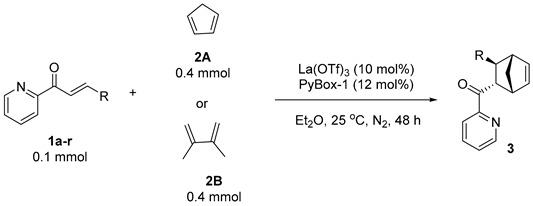


***General Procedure***: To an oven-dried reaction tube equipped with a magnetic stirring bar was added 2-alkenoyl pyridines **1** (0.1 mmol, 1.0 equiv), La(OTf)_3_ (5.9 mg, 0.01 mmol) and ligand **PyBox-1** (6.3 mg, 0.012 mmol). The tube was sealed immediately with a rubber stopper and protected with a nitrogen balloon by evacuation-backfill operations (repeated three times). Dry Et_2_O (1 mL) was injected to the tube via a syringe. The resultant mixture was stirred for about 1 h, followed by addition of cyclopentadiene (**2A**) (33 μL, 0.4 mmol, 4.0 equiv) or 2,3-dimethylbuta-2,3-diene (**2B**) (90 μL, 0.4 mmol, 4.0 equiv) via a microsyringe. The mixture was stirred at 25 °C for 48 h. The solvent was evaporated under reduced pressure, and the crude mixture was subjected to column chromatography on silica gel to afford the corresponding products.

#### 3.4.1. ((1*R*,2*S*,3*S*,4*S*)-3-Phenylbicyclo[2.2.1]hept-5-en-2-yl)(pyridin-2-yl)methanone (**3aA**)

Colorless oil, 25 mg, yield 91%, R*_f_* = 0.5 (PE/EA = 10:1, *v*/*v*). **^1^H NMR** (400 MHz, CDCl_3_) δ 8.76–8.60 (d, *J* = 4.4 Hz, 1H), 8.00 (d, *J* = 7.8 Hz, 1H), 7.79 (td, *J* = 7.7, 1.8 Hz, 1H), 7.42 (ddd, *J* = 7.6, 4.8, 1.3 Hz, 1H), 7.36–7.06 (m, 6H), 6.49 (dd, *J* = 5.6, 3.2 Hz, 1H), 5.82 (dd, *J* = 5.6, 2.8 Hz, 1H), 4.53 (dd, *J* = 5.2, 3.4 Hz, 1H), 3.54 (s, 1H), 3.46 (d, *J* = 4.8 Hz, 1H), 3.09 (s, 1H), 2.07 (d, *J* = 8.4 Hz, 1H), 1.61 (dd, *J* = 8.4, 2.0 Hz, 1H). **^13^C NMR** (101 MHz, CDCl_3_) δ 201.0, 153.5, 148.8, 144.6, 139.3, 136.7, 132.8, 128.3, 127.6, 126.8, 125.7, 122.1, 54.2, 49.3, 48.7, 48.2, 45.5.

**HPLC** (Daicel Chiralpak OD-H, *^n^*hexane/*^i^*PrOH = 97:3, 0.65 mL/min, T = 15 °C): t_R1_ (major) = 17.172 min, t_R2_ (minor) = 12.070 min; dr: 89:11; er: 94:6.

**[α]^20^_D_** = +135.7 (c = 1.0, CHCl_3_).

#### 3.4.2. ((1*R*,2*S*,3*S*,4*S*)-3-(2-Fluorophenyl)bicyclo[2.2.1]hept-5-en-2-yl)(pyridin-2-yl)methanone (**3bA**)

Colorless oil, 28 mg, yield 96%, R*_f_* = 0.55 (PE/EA = 10:1, *v*/*v*). **^1^H NMR** (400 MHz, CDCl_3_) δ 8.66 (ddd, *J* = 4.8, 1.8, 0.9 Hz, 1H), 8.05 (dt, *J* = 7.9, 1.1 Hz, 1H), 7.82 (td, *J* = 7.7, 1.8 Hz, 1H), 7.48–7.40 (m, 2H), 7.19–7.08 (m, 2H), 6.94 (ddd, *J* = 10.5, 7.9, 1.5 Hz, 1H), 6.48 (dd, *J* = 5.7, 3.2 Hz, 1H), 5.88 (dd, *J* = 5.6, 2.8 Hz, 1H), 4.53 (dd, *J* = 5.1, 3.5 Hz, 1H), 3.53 (d, *J* = 5.0 Hz, 1H), 3.49 (s, 1H), 3.19 (d, *J* = 2.1 Hz, 1H), 1.97 (d, *J* = 8.5 Hz, 1H), 1.62 (dd, *J* = 8.5, 1.8 Hz, 1H). **^13^C NMR** (101 MHz, CDCl_3_) δ 200.2,160.2 (d, *J* = 246 Hz), 153.5, 148.8, 138.3, 136.8, 133.3, 131.8, 127.2 (d, *J* = 9.3 Hz), 126.8, 123.8 (d, *J* = 2.8 Hz), 122.2, 115.2 (d, *J* = 22.7 Hz), 52.5, 48.5, 48.1, 47.6, 39.2. **^19^F NMR** (376 MHz, CDCl_3_) δ −139.01. **HRMS** (ESI): *m*/*z* [M+H]^+^ calculated for C_19_H_17_FNO^+^ 294.1289, found 294.1288.

**HPLC** (Daicel Chiralpak OD-H, *^n^*hexane/*^i^*PrOH = 97:3, 0.65 mL/min, T = 15 °C): t_R1_ (major) = 14.393 min, t_R2_ (minor) = 13.080 min; dr: 65:35; er: 87:13.

**[α]^20^_D_** = +81.4 (c = 1.7, CHCl_3_).

#### 3.4.3. ((1*R*,2*S*,3*S*,4*S*)-3-(4-Fluorophenyl)bicyclo[2.2.1]hept-5-en-2-yl)(pyridin-2-yl)methanone (**3cA**)

Colorless oil, 27 mg, yield 92%, R*_f_* = 0.55 (PE/EA = 10:1, *v*/*v*). **^1^H NMR** (400 MHz, CDCl_3_) δ 8.68 (d, *J* = 4.6 Hz, 1H), 8.00 (d, *J* = 7.8 Hz, 1H), 7.82 (td, *J* = 7.7, 1.8 Hz, 1H), 7.46 (ddd, *J* = 7.6, 4.8, 1.3 Hz, 1H), 7.30–7.21 (m, 2H), 7.01–6.85 (m, 2H), 6.48 (dd, *J* = 5.7, 3.2 Hz, 1H), 5.83 (dd, *J* = 5.6, 2.8 Hz, 1H), 4.46 (dd, *J* = 5.2, 3.4 Hz, 1H), 3.54 (s, 1H), 3.42 (d, *J* = 4.7 Hz, 1H), 3.07–3.01 (dd, *J* = 1.2 Hz, 1H), 2.03 (d, *J* = 8.5 Hz, 1H), 1.62 (dd, *J* = 8.5, 1.8 Hz, 1H). **^13^C NMR** (101 MHz, CDCl_3_) δ 201.0, 160.2, 153.5, 148.9, 140.2, 139.3, 136.8, 132.9, 129.0, 128.9 (d, *J* = 7.8 Hz), 126.9, 122.2, 115.1 (d, *J* = 21.0 Hz), 54.5, 49.4, 48.7, 48.1, 44.9. **^19^F NMR** (376 MHz, CDCl_3_) δ −117.84.

**HPLC** (Daicel Chiralpak OD-H, *^n^*hexane/*^i^*PrOH = 97:3, 0.65 mL/min, T = 15 °C): t_R1_ (major) = 14.534 min, t_R2_ (minor) = 11.045 min; dr: 89:11; er:95.5:4.5.

**[α]^20^_D_** = +127.6 (c = 2.3, CHCl_3_).

#### 3.4.4. ((1*R*,2*S*,3*S*,4*S*)-3-(2,3-Difluorophenyl)bicyclo[2.2.1]hept-5-en-2-yl)(pyridin-2-yl)methanone (**3dA**)

Colorless oil, 27 mg, yield 87%, R*_f_* = 0.5 (PE/EA = 10:1, *v*/*v*). **^1^H NMR** (400 MHz, CDCl_3_) δ 8.66 (ddd, *J* = 4.8, 1.8, 0.9 Hz, 1H), 8.05 (dt, *J* = 7.9, 1.1 Hz, 1H), 7.83 (td, *J* = 7.7, 1.8 Hz, 1H), 7.46 (ddd, *J* = 7.6, 4.8, 1.3 Hz, 1H), 7.17 (ddd, *J* = 7.0, 4.6, 2.0 Hz, 1H), 7.04–6.93 (m, 2H), 6.47 (dd, *J* = 5.6, 3.2 Hz, 1H), 5.89 (dd, *J* = 5.6, 2.8 Hz, 1H), 4.51 (dd, *J* = 5.1, 3.5 Hz, 1H), 3.55 (d, *J* = 5.2 Hz, 1H), 3.51 (s, 1H), 3.17 (d, *J* = 1.7 Hz, 1H), 1.95 (d, *J* = 8.6 Hz, 1H), 1.63 (dd, *J* = 8.6, 1.8 Hz, 1H). **^13^C NMR** (101 MHz, CDCl_3_) δ 199.8, 153.3, 148.8, 138.2, 136.9, 134.5 (d, *J* = 10.7 Hz), 133.5, 127.0, 123.6 (t, *J* = 5.8, 11.9 Hz), 122.3, 121.9 (d, *J* = 2.7 Hz), 114.6 (d, *J* = 5.0 Hz), 52.6, 48.5, 48.2, 47.8, 39.1. **^19^F NMR** (376 MHz, CDCl_3_) δ −111.33, 116.03. **HRMS** (ESI): *m/z* [M+H]^+^ calculated for C_19_H_16_F_2_NO^+^ 312.1194, found 312.1199.

**HPLC** (Daicel Chiralpak OD-H, *^n^*hexane/*^i^*PrOH = 97:3, 0.65 mL/min, T = 15 °C): t_R1_ (major) = 16.212 min, t_R2_ (minor) = 11.443 min; dr: 86:14; er: 90:10.

**[α]^20^_D_** = +93.5 (c = 2.1, CHCl_3_).

#### 3.4.5. ((1*S*,2*R*,3*R*,4*R*)-3-(3-Chlorophenyl)bicyclo[2.2.1]hept-5-en-2-yl)(pyridin-2-yl)methanone (**3eA**)

Colorless oil, 25 mg, yield 81%, R*_f_* = 0.5 (PE/EA = 10:1, *v*/*v*). **^1^H NMR** (400 MHz, CDCl_3_) δ 8.68 (d, *J* = 4.3 Hz, 1H), 8.01 (d, *J* = 7.9 Hz, 1H), 7.83 (td, *J* = 7.7, 1.8 Hz, 1H), 7.46 (ddd, *J* = 7.6, 4.7, 1.3 Hz, 1H), 7.30–7.07 (m, 4H), 6.47 (dd, *J* = 5.7, 3.2 Hz, 1H), 5.83 (dd, *J* = 5.6, 2.8 Hz, 1H), 4.46 (dd, *J* = 5.2, 3.4 Hz, 1H), 3.56 (s, 1H), 3.42 (d, *J* = 4.2 Hz, 1H), 3.07 (d, *J* = 1.8 Hz, 1H), 2.03 (d, *J* = 8.5 Hz, 1H), 1.63 (dd, *J* = 8.6, 1.8 Hz, 1H). **^13^C NMR** (101 MHz, CDCl_3_) δ 200.7, 153.4, 148.9, 146.8, 139.2, 136.9, 134.2, 133.0, 129.6, 127.6, 127.0, 126.0, 122.2, 54.4, 49.1, 48.7, 48.2, 45.3.

**HPLC** (Daicel Chiralpak AD-H, *^n^*hexane/*^i^*PrOH = 99.5:0.5, 0.5 mL/min, T = 12 °C): t_R1_ (major) = 36.171 min, t_R2_ (minor) = 38.433 min; dr: 89:11; er: 92:8.

**[α]^20^_D_** = +112.8 (c = 1.6, CHCl_3_).

#### 3.4.6. ((1*R*,2*S*,3*S*,4*S*)-3-(4-Chlorophenyl)bicyclo[2.2.1]hept-5-en-2-yl)(pyridin-2-yl)methanone (**3fA**)

Colorless oil, 30 mg, yield 98%, R*_f_* = 0.5 (PE/EA = 10:1, *v*/*v*). **^1^H NMR** (400 MHz, CDCl_3_) δ 8.70 (d, *J* = 4.8 Hz, 1H), 8.03 (d, *J* = 7.9 Hz, 1H), 7.85 (td, *J* = 7.7, 1.8 Hz, 1H), 7.48 (ddd, *J* = 7.5, 4.7, 1.3 Hz, 1H), 7.26 (m, 4H), 6.50 (dd, *J* = 5.7, 3.2 Hz, 1H), 5.85 (dd, *J* = 5.6, 2.8 Hz, 1H), 4.48 (dd, *J* = 5.2, 3.4 Hz, 1H), 3.57 (s, 1H), 3.43 (dd, *J* = 5.2, 1.8 Hz, 1H), 3.07 (d, *J* = 1.7 Hz, 1H), 2.03 (d, *J* = 8.5 Hz, 1H), 1.64 (dd, *J* = 8.5, 1.8 Hz, 1H). **^13^C NMR** (101 MHz, CDCl_3_) δ 200.9, 153.4, 148.9, 143.1, 139.2, 136.8, 133.0, 131.5, 128.9, 128.4, 127.0, 122.2, 54.4, 49.2, 48.7, 48.1, 45.0.

**HPLC** (Daicel Chiralpak OD-H, *^n^*hexane/*^i^*PrOH = 97:3, 0.65 mL/min, T = 15 °C): t_R1_ (ma*J*or) = 14.238 min, t_R2_ (minor) = 10.897 min; dr: 88:12; er: 92:8.

**[α]^20^_D_** = +104.4 (c = 1.4, CHCl_3_).

#### 3.4.7. ((1*R*,2*S*,3*S*,4*S*)-3-(2-Bromophenyl)bicyclo[2.2.1]hept-5-en-2-yl)(pyridin-2-yl)methanone (**3gA**)

Colorless oil, 28 mg, yield 80%, R*_f_* = 0.5 (PE/EA = 10:1, *v*/*v*). **^1^H NMR** (400 MHz, CDCl_3_) δ 8.66 (d, *J* = 4.9 Hz, 1H), 8.02 (d, *J* = 7.9 Hz, 1H), 7.81 (td, *J* = 7.7, 1.8 Hz, 1H), 7.53 (ddd, *J* = 11.1, 8.0, 1.6 Hz, 2H), 7.44 (ddd, *J* = 7.6, 4.8, 1.3 Hz, 1H), 7.32–7.25 (m, 1H), 7.05 (td, *J* = 7.7, 1.7 Hz, 1H), 6.53 (dd, *J* = 5.6, 3.2 Hz, 1H), 5.90 (dd, *J* = 5.6, 2.8 Hz, 1H), 4.68 (dd, *J* = 5.1, 3.5 Hz, 1H), 3.60 (d, *J* = 3.5 Hz, 1H), 3.48 (s, 1H), 3.06 (dd, *J* = 3.3, 1.7 Hz, 1H), 1.99 (d, *J* = 8.5 Hz, 1H), 1.59 (dd, *J* = 8.5, 1.8 Hz, 1H). **^13^C NMR** (101 MHz, CDCl_3_) δ 200.3, 153.6 148.8, 143.4, 138.5, 136.8, 133.6, 133.1, 127.9, 127.4, 127.2, 126.8, 126.3, 122.2, 51.2, 49.9, 48.4, 47.7, 46.3.

**HPLC** (Daicel Chiralpak OD-H, *^n^*hexane/*^i^*PrOH = 99:1, 0.65 mL/min, T = 15 °C): t_R1_ (major) = 18.929 min, t_R2_ (minor) = 17.916 min; dr: 64:36; er: 88:12.

**[α]^20^_D_** = +61.9 (c = 2.3, CHCl_3_).

#### 3.4.8. ((1*R*,2*S*,3*S*,4*S*)-3-(4-Bromophenyl)bicyclo[2.2.1]hept-5-en-2-yl)(pyridin-2-yl)methanone (**3hA**)

Colorless oil, 30 mg, yield 85%, R*_f_* = 0.5 (PE/EA = 10:1, *v*/*v*). **^1^H NMR** (400 MHz, CDCl_3_) δ 8.67 (ddd, *J* = 4.8, 1.8, 0.9 Hz, 1H), 8.00 (dt, *J* = 7.9, 1.1 Hz, 1H), 7.82 (td, *J* = 7.7, 1.8 Hz, 1H), 7.46 (ddd, *J* = 7.5, 4.7, 1.3 Hz, 1H), 7.41–7.35 (m, 2H), 7.21–7.15 (m, 2H), 6.47 (dd, *J* = 5.6, 3.2 Hz, 1H), 5.83 (dd, *J* = 5.7, 2.8 Hz, 1H), 4.46 (dd, *J* = 5.2, 3.4 Hz, 1H), 3.55 (s, 1H), 3.39 (d, *J* = 4.6 Hz, 1H), 3.04 (d, *J* = 2.2 Hz, 1H), 2.01 (d, *J* = 8.6 Hz, 1H), 1.62 (dd, *J* = 8.6, 1.8 Hz, 1H). **^13^C NMR** (101 MHz, CDCl_3_) δ 200.8, 153.4, 148.8, 143.7, 139.2, 136.9, 133.0, 131.3, 129.4, 127.0, 122.2, 119.5, 54.4, 49.2, 48.7, 48.1, 45.1.

**HPLC** (Daicel Chiralpak OD-H, *^n^*hexane/*^i^*PrOH = 97:3, 0.65 mL/min, T = 15 °C): t_R1_ (major) = 15.277 min, t_R2_ (minor) = 11.426 min; dr: 88.5:11.5; er: 88:12.

**[α]^20^_D_** = +90.2 (c = 2.3, CHCl_3_).

#### 3.4.9. 4-((1*S*,2*S*,3*S*,4*R*)-3-Picolinoylbicyclo[2.2.1]hept-5-en-2-yl)benzonitrile (**3iA**)

Colorless oil, 25 mg, yield 84%, R*_f_* = 0.6 (PE/EA = 10:1, *v*/*v*). **^1^H NMR** (400 MHz, CDCl_3_) δ 8.67 (d, *J* = 4.5 Hz, 1H), 8.01 (d, *J* = 7.6 Hz 1H), 7.88–7.82 (m, 1H), 7.60–7.54 (m, 2H), 7.48 (ddd, *J* = 7.6, 4.0, 2.8 Hz, 1H), 7.40 (d, *J* = 8.3 Hz, 2H), 6.48 (dd, *J* = 5.7, 3.2 Hz, 1H), 5.86 (dd, *J* = 5.6, 2.8 Hz, 1H), 4.45 (dd, *J* = 5.3, 3.4 Hz, 1H), 3.59 (s, 1H), 3.49 (d, *J* = 4.8 Hz, 1H), 3.10 (dd, *J* = 3.3, 1.6 Hz, 1H), 1.99 (d, *J* = 8.6 Hz, 1H), 1.65 (dd, *J* = 8.6, 1.8 Hz, 1H). **^13^C NMR** (101 MHz, CDCl_3_) δ 200.4, 153.2, 150.5, 148.9, 139.0, 136.9, 133.3, 132.2, 128.4, 127.1, 122.3, 119.0, 109.6, 54.5, 48.8, 48.7, 48.2, 45.8.

**HPLC** (Daicel Chiralpak OD-H, *^n^*hexane/*^i^*PrOH = 97:3, 0.65 mL/min, T = 13 °C): t_R1_ (major) = 34.765 min, t_R2_ (minor) = 40.230 min; dr: 73.5:26.5; er:87:13.

**[α]^20^_D_** = +79.6 (c = 2.0, CHCl_3_).

#### 3.4.10. Pyridin-2-yl((1*R*,2*S*,3*S*,4*S*)-3-(p-tolyl)bicyclo[2.2.1]hept-5-en-2-yl)methanone (**3jA**)

Colorless oil, 26 mg, yield 91%, R*_f_* = 0.6 (PE/EA = 10:1, *v*/*v*). **^1^H NMR** (400 MHz, CDCl_3_) δ 8.67 (d, *J* = 4.0 Hz, 1H), 7.99 (d, *J* = 7.8 Hz, 1H), 7.80 (td, *J* = 7.7, 1.8 Hz, 1H), 7.44 (ddd, *J* = 7.6, 4.8, 1.3 Hz, 1H), 7.21 (d, *J* = 8.0 Hz, 2H), 7.08 (d, *J* = 7.9 Hz, 2H), 6.48 (dd, *J* = 5.6, 3.2 Hz, 1H), 5.81 (dd, *J* = 5.6, 2.8 Hz, 1H), 4.52 (dd, *J* = 5.2, 3.4 Hz, 1H), 3.53 (s, 1H), 3.41 (d, *J* = 4.8 Hz, 1H), 3.05 (s, 1H), 2.30 (s, 3H), 2.06 (d, *J* = 8.4 Hz, 1H), 1.59 (dd, *J* = 8.5, 1.8 Hz, 1H). **^13^C NMR** (101 MHz, CDCl_3_) δ 201.2, 153.6, 148.8, 141.5, 139.4, 136.8, 135.2, 132.7, 129.0, 127.5, 126.8, 122.1, 54.1, 49.6, 48.7, 48.2, 45.2, 20.9.

**HPLC** (Daicel Chiralpak OD-H, *^n^*hexane/*^i^*PrOH = 97:3, 0.65 mL/min, T = 12 °C): t_R1_ (major) = 16.212 min, t_R2_ (minor) = 11.443 min; dr: 86:14; er: 93:7.

**[α]^20^_D_** = +111.6 (c = 2.3, CHCl_3_).

#### 3.4.11. ((1*R*,2*S*,3*S*,4*S*)-3-(3,5-Dimethylphenyl)bicyclo[2.2.1]hept-5-en-2-yl)(pyridin-2-yl)methanone (**3kA**)

Colorless oil, 24 mg, yield 86%, R*_f_* = 0.6 (PE/EA = 10:1, *v*/*v*). **^1^H NMR** (400 MHz, CDCl_3_) δ 8.69 (d, *J* = 4.6 Hz, 1H), 8.00 (d, *J* = 7.8 Hz, 1H), 7.81 (td, *J* = 7.7, 1.7 Hz, 1H), 7.44 (ddd, *J* = 7.5, 4.7, 1.2 Hz, 1H), 6.94 (s, 2H), 6.81 (s, 1H), 6.48 (dd, *J* = 5.7, 3.2 Hz, 1H), 5.80 (dd, *J* = 5.6, 2.8 Hz, 1H), 4.49 (dd, *J* = 5.2, 3.4 Hz, 1H), 3.54 (s, 1H), 3.38 (d, *J* = 4.9 Hz, 1H), 3.05 (s, 1H), 2.27 (s, 6H), 2.09 (d, *J* = 8.4 Hz, 1H), 1.60 (dd, *J* = 8.5, 1.8 Hz, 1H). **^13^C NMR** (101 MHz, CDCl_3_) δ 201.1, 153.6, 148.8, 144.5, 139.5, 137.8, 136.8, 132.7, 127.5, 126.8, 125.5, 122.2, 54.2, 49.5, 48.7, 48.3, 45.3, 21.4. **HRMS** (ESI): *m*/*z* [M+H]^+^ calculated for C_21_H_22_NO^+^ 304.1696, found 304.1692.

**HPLC** (Daicel Chiralpak OD-H, *^n^*hexane/*^i^*PrOH = 97:3, 0.65 mL/min, T = 11 °C): t_R1_ (major) = 25.518 min, t_R2_ (minor) = 9.663 min; dr: 90:10; er: 89:11.

**[α]^20^_D_** = +111.8 (c = 1.9, CHCl_3_).

#### 3.4.12. ((1*R*,2*S*,3*S*,4*S*)-3-(4-Methoxyphenyl)bicyclo[2.2.1]hept-5-en-2-yl)(pyridin-2-yl)methanone (**3lA**)

Colorless oil, 21 mg, yield 68%, R*_f_* = 0.4 (PE/EA = 10:1, *v*/*v*). **^1^H NMR** (400 MHz, CDCl_3_) δ 8.68 (ddd, *J* = 4.8, 1.7, 0.9 Hz, 1H), 8.00 (dt, *J* = 7.8, 1.1 Hz, 1H), 7.81 (td, *J* = 7.7, 1.8 Hz, 1H), 7.44 (ddd, *J* = 7.6, 4.8, 1.3 Hz, 1H), 7.26–7.22 (m, 2H), 6.86–6.80 (m, 2H), 6.48 (dd, *J* = 5.7, 3.2 Hz, 1H), 5.81 (dd, *J* = 5.7, 2.8 Hz, 1H), 4.49 (dd, *J* = 5.2, 3.4 Hz, 1H), 3.77 (s, 3H), 3.53 (s, 1H), 3.38 (d, *J* = 4.4 Hz, 1H), 3.05–3.00 (m, 1H), 2.08–2.03 (m, 1H), 1.60 (dd, *J* = 8.5, 1.8 Hz, 1H). **^13^C NMR** (101 MHz, CDCl_3_) δ 201.2, 157.7, 153.6, 148.8, 139.4, 136.8, 132.7, 128.5, 126.8, 122.1, 113.7, 55.2, 54.2, 49.6, 48.7, 48.1, 44.9.

**HPLC** (Daicel Chiralpak OD-H, *^n^*hexane/*^i^*PrOH = 99:1, 0.65 mL/min, T = 12 °C): t_R1_ (major) = 40.987 min, t_R2_ (minor) = 22.953 min; dr: 90:10; er: 89:11.

**[α]^20^_D_** = +91.5 (c = 2.2, CHCl_3_).

#### 3.4.13. ((1*R*,2*S*,3*S*,4*S*)-3-(Naphthalen-2-yl)bicyclo[2.2.1]hept-5-en-2-yl)(pyridin-2-yl)methanone (**3mA**)

Colorless oil, 29 mg, yield 89%, R*_f_* = 0.65 (PE/EA = 10:1, *v*/*v*). **^1^H NMR** (400 MHz, CDCl_3_) δ 8.66 (d, *J* = 4.5 Hz, 1H), 8.16–8.11 (m, 1H), 8.08 (d, *J* = 8.0 Hz, 1H), 7.83 (td, *J* = 7.5, 1.7 Hz, 2H), 7.73 (d, *J* = 8.2 Hz, 1H), 7.66 (d, *J* = 7.2 Hz, 1H), 7.50–7.41 (m, 4H), 6.66 (dd, *J* = 5.7, 3.2 Hz, 1H), 5.96 (dd, *J* = 5.6, 2.8 Hz, 1H), 4.73 (dd, *J* = 5.1, 3.5 Hz, 1H), 4.10 (d, *J* = 4.9 Hz, 1H), 3.57 (s, 1H), 3.24 (s, 1H), 2.23 (d, *J* = 8.4 Hz, 1H), 1.72 (dd, *J* = 8.5, 1.8 Hz, 1H). **^13^C NMR** (101 MHz, CDCl_3_) δ 201.2, 153.5, 148.8, 140.7, 139.0, 136.8, 133.8, 133.4, 132.7, 128.6, 126.9, 126.6, 125.9, 125.4, 125.3, 124.3, 123.0, 122.2, 52.7, 49.9, 48.8, 48.5, 41.6. **HRMS** (ESI): *m*/*z* [M+H]^+^ calculated for C_23_H_20_NO^+^ 326.1539, found 326.1543.

**HPLC** (Daicel Chiralpak OD-H, *^n^*hexane/*^i^*PrOH = 98:2, 0.65 mL/min, T = 15 °C): t_R1_ (major) = 25.574 min, t_R2_ (minor) = 12.468 min; dr: 92:2; er: 81:19.

**[α]^20^_D_** = +92.6 (c = 1.2, CHCl_3_).

#### 3.4.14. Pyridin-2-yl((1*R*,2*S*,3*S*,4*S*)-3-(thiophen-2-yl)bicyclo[2.2.1]hept-5-en-2-yl)methanone (**3nA**)

Colorless oil, 22 mg, yield 78%, R*_f_* = 0.4 (PE/EA = 10:1, *v*/*v*). **^1^H NMR** (400 MHz, CDCl_3_) δ 8.70 (ddd, *J* = 4.8, 1.7, 0.9 Hz, 1H), 8.00 (dt, *J* = 7.8, 1.1 Hz, 1H), 7.82 (td, *J* = 7.7, 1.8 Hz, 1H), 7.46 (ddd, *J* = 7.6, 4.8, 1.3 Hz, 1H), 7.14–7.08 (m, 1H), 6.93–6.88 (m, 2H), 6.45 (dd, *J* = 5.7, 3.2 Hz, 1H), 5.80 (dd, *J* = 5.6, 2.8 Hz, 1H), 4.57 (dd, *J* = 4.9, 3.4 Hz, 1H), 3.63 (dd, *J* = 4.9, 1.7 Hz, 1H), 3.55 (s, 1H), 3.06 (s, 1H), 2.11 (d, *J* = 8.7 Hz, 1H), 1.64 (dd, *J* = 8.7, 1.8 Hz, 1H). **^13^C NMR** (101 MHz, CDCl_3_) δ 200.4, 153.4, 148.9, 138.6, 136.8, 132.7, 126.9, 126.6, 123.7, 122.9, 122.2, 55.7, 51.5, 48.6, 48.5, 41.6.

**HPLC** (Daicel Chiralpak OD-H, *^n^*hexane/*^i^*PrOH = 99:1, 0.65 mL/min, T = 17 °C): t_R1_ (major) = 20.617 min, t_R2_ (minor) = 16.158 min; dr: 76:24; er: 89:11.

**[α]^20^_D_** = +125.6 (c = 1.4, CHCl_3_).

#### 3.4.15. ((1*R*,2*S*,3*S*,4*S*)-3-(Benzo[b]thiophen-2-yl)bicyclo[2.2.1]hept-5-en-2-yl)(pyridin-2-yl)methanone (**3oA**)

Colorless oil, 32 mg, yield 97%, R*_f_* = 0.4 (PE/EA = 10:1, *v*/*v*). **^1^H NMR** (400 MHz, CDCl_3_) δ 8.70 (d, *J* = 4.6 Hz, 1H), 8.01 (d, *J* = 7.8 Hz, 1H), 7.81 (td, *J* = 7.7, 1.8 Hz, 1H), 7.76–7.71 (m, 1H), 7.64 (dd, *J* = 7.6, 1.4 Hz, 1H), 7.45 (ddd, *J* = 7.6, 4.8, 1.3 Hz, 1H), 7.29–7.21 (m, 2H), 7.13 (s, 1H), 6.48 (dd, *J* = 5.7, 3.2 Hz, 1H), 5.84 (dd, *J* = 5.7, 2.8 Hz, 1H), 4.67 (dd, *J* = 5.0, 3.4 Hz, 1H), 3.69 (d, *J* = 5.1 Hz, 1H), 3.59 (s, 1H), 3.18 (s, 1H), 2.14 (d, *J* = 8.7 Hz, 1H), 1.67 (dd, *J* = 8.7, 1.8 Hz, 1H). **^13^C NMR** (101 MHz, CDCl_3_) δ 200.2, 153.3, 149.8, 148.9, 140.0, 139.0, 138.6, 136.8, 132.9, 127.0, 124.1, 123.5, 122.8, 122.2, 122.0, 120.0, 55.1, 51.0, 48.7, 48.6, 42.3. **HRMS** (ESI): *m/z* [M+H]^+^ calculated for C_21_H_18_NOS^+^ 332.1104, found 332.1110.

**HPLC** (Daicel Chiralpak OD-H, *^n^*hexane/*^i^*PrOH = 97:3, 0.65 mL/min, T = 11 °C): t_R1_ (major) = 28.571 min, t_R2_ (minor) = 14.914 min; dr: 84.5:15.5; er: 90:10.

**[α]^20^_D_** = +109.6 (c = 2.3, CHCl_3_).

#### 3.4.16. ((1*R*,2*S*,3*S*,4*S*)-3-Cyclohexylbicyclo[2.2.1]hept-5-en-2-yl)(pyridin-2-yl)methanone (**3pA**)

Colorless oil, 21 mg, yield 78%, R*_f_* = 0.6 (PE/EA = 10:1, *v*/*v*). **^1^H NMR** (400 MHz, CDCl_3_) δ 8.75–8.71 (m, 1H), 7.98 (dt, *J* = 7.8, 1.2 Hz, 1H), 7.83 (dd, *J* = 7.7, 1.8 Hz, 1H), 7.46 (ddd, *J* = 7.6, 4.8, 1.4 Hz, 1H), 6.33 (dd, *J* = 5.7, 3.3 Hz, 1H), 5.68 (dd, *J* = 5.6, 2.8 Hz, 1H), 4.12 (dd, *J* = 5.0, 3.4 Hz, 1H), 3.30 (s, 1H), 2.91 (d, *J* = 2.3 Hz, 1H), 2.02–1.94 (m, 1H), 1.80 (ddd, *J* = 10.3, 5.0, 1.7 Hz, 1H), 1.77–1.71 (m, 2H), 1.63–1.56 (m, 3H), 1.47–1.43 (m, 1H), 1.24–1.07 (m, 6H). **^13^C NMR** (101 MHz, CDCl_3_) δ 201.8, 153.6, 148.8, 139.3, 136.8, 131.7, 126.7, 122.2, 51.4, 48.5, 48.1, 47.8, 44.6, 42.3, 32.9, 32.5, 26.6, 26.5, 26.3.

**HPLC** (Daicel Chiralpak AD-H, *^n^*hexane/*^i^*PrOH = 99:1, 0.8 mL/min, T = 12 °C): t_R1_ (major) = 10.062 min, t_R2_ (minor) = 8.462 min; dr: 82:18; er: 99:1.

**[α]^20^_D_** = +70.2 (c = 2.8, CHCl_3_).

#### 3.4.17. ((1*R*,2*S*,3*S*,4*S*)-3-Cyclopentylbicyclo[2.2.1]hept-5-en-2-yl)(pyridin-2-yl)methanone (**3qA**)

Colorless oil, 22 mg, yield 82%, R*_f_* = 0.6 (PE/EA = 10:1, *v*/*v*). **^1^H NMR** (400 MHz, CDCl_3_) δ 8.73 (d, *J* = 4.7 Hz, 1H), 7.99 (d, *J* = 7.8 Hz, 1H), 7.83 (td, *J* = 7.7, 1.7 Hz, 1H), 7.47 (ddd, *J* = 7.6, 4.8, 1.3 Hz, 1H), 6.36 (dd, *J* = 5.7, 3.2 Hz, 1H), 5.68 (dd, *J* = 5.7, 2.8 Hz, 1H), 4.14–4.09 (m, 1H), 3.33 (s, 1H), 2.80–2.73 (m, 1H), 1.97–1.39 (m, 12H), 1.33–1.00 (m, 3H). **^13^C NMR** (101 MHz, CDCl_3_) δ 201.9, 153.8, 148.8, 139.2, 136.8, 131.4, 126.7, 122.1, 52.3, 48.4, 48.2, 47.5, 47.1, 45.6, 32.4, 32.2, 25.3, 25.0. **HRMS** (ESI): *m/z* [M+H]^+^ calculated for C_18_H_22_NO^+^ 268.1696, found 268.1700.

**HPLC** (Daicel Chiralpak AD-H, *^n^*hexane/*^i^*PrOH = 99:1, 0.5 mL/min, T = 16 °C): t_R1_ (major) = 13.391 min, t_R2_ (minor) = 14.348 min; dr: 82:18; er: 94.5:5.5.

**[α]^20^_D_** = +99.4 (c = 3.7, CHCl_3_).

#### 3.4.18. ((1*R*,2*S*,3*S*,4*S*)-3-Cyclopropylbicyclo[2.2.1]hept-5-en-2-yl)(pyridin-2-yl)methanone (**3rA**)

Colorless oil, 18 mg, yield 75%, R*_f_* = 0.55 (PE/EA = 10:1, *v*/*v*). **^1^H NMR** (400 MHz, CDCl_3_) δ 8.72 (dt, *J* = 4.8, 1.2 Hz, 1H), 7.95 (d, *J* = 7.8 Hz, 1H), 7.81 (td, *J* = 7.7, 1.8 Hz, 1H), 7.46 (ddd, *J* = 7.5, 4.8, 1.3 Hz, 1H), 6.29 (dd, *J* = 5.7, 3.2 Hz, 1H), 5.66 (dd, *J* = 5.7, 2.8 Hz, 1H), 4.21–4.16 (m, 1H), 3.37 (s, 1H), 2.80 (s, 1H), 1.91 (d, *J* = 8.4 Hz, 1H), 1.53 (dd, *J* = 8.5, 1.9 Hz, 1H), 1.41 (dd, *J* = 4.8, 2.5 Hz, 1H), 0.76 (dddd, *J* = 12.8, 9.8, 8.0, 4.9 Hz, 1H), 0.49–0.36 (m, 2H), 0.22 (dq, *J* = 9.3, 4.8 Hz, 1H), 0.06–0.00 (m, 1H). **^13^C NMR** (101 MHz, CDCl_3_) δ 201.7, 153.8, 148.8, 139.0, 136.8, 131.8, 126.7, 122.1, 53.5, 48.6, 48.1, 48.0, 47.2, 15.9, 5.0, 4.4. **HRMS** (ESI): *m/z* [M+H]^+^ calculated for C_16_H_18_NO^+^ 240.1383, found 240.1389.

**HPLC** (Daicel Chiralpak AD-H, *^n^*hexane/*^i^*PrOH = 99.5:0.5, 0.5 mL/min, T = 18 °C): t_R1_ (major) = 15.321 min, t_R2_ (minor) = 16.065 min; dr: 87.5:12.5; er: 77:23.

**[α]^20^_D_** = +46.2 (c = 3.8, CHCl_3_).

#### 3.4.19. ((1*S*,2*S*)-4,5-Dimethyl-1,2,3,6-tetrahydro-[1,1’-biphenyl]-2-yl)(pyridin-2-yl)methanone (**3aB**)

Colorless oil, 13 mg, yield 45%, R*_f_* = 0.75 (PE/EA = 5:1, *v*/*v*). **^1^H NMR** (400 MHz, CDCl_3_) δ 8.66 (dt, *J* = 4.9, 1.3 Hz, 1H), 7.78–7.73 (m, 1H), 7.68 (td, *J* = 7.6, 1.7 Hz, 1H), 7.38 (ddd, *J* = 7.4, 4.8, 1.4 Hz, 1H), 7.23–7.17 (m, 2H), 7.11 (dd, *J* = 8.4, 6.8 Hz, 2H), 7.06–6.99 (m, 1H), 4.74 (ddd, *J* = 11.6, 10.2, 5.7 Hz, 1H), 3.26 (ddd, *J* = 11.5, 9.9, 6.5 Hz, 1H), 2.36–2.20 (m, 4H), 1.67 (s, 6H). **^13^C NMR** (101 MHz, CDCl_3_) δ 204.7, 153.3, 148.7, 144.9, 136.7, 128.1, 127.5, 126.8, 125.9, 125.5, 124.2, 122.0, 45.3, 43.1, 41.2, 36.4, 18.7. **HRMS** (ESI): *m/z* [M+H]^+^ calculated for C_20_H_22_NO^+^ 292.1696, found 292.1697.

**HPLC** (Daicel Chiralpak OD-H, *^n^*hexane/*^i^*PrOH = 98:2, 1.0 mL/min, T = 14 °C): t_R1_ (major) = 7.349 min, t_R2_ (minor) = 6.717 min; dr: >20:1; er: 82:18.

**[α]^20^_D_** = −21.5 (c = 0.2, CHCl_3_).

### 3.5. Synthesis and Characterization of Product ***5***


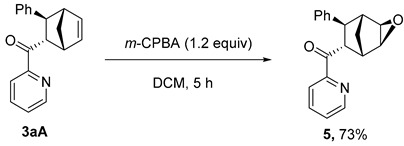


***Procedure***: To an oven-dried reaction tube equipped with a magnetic stirring bar was added **3aA** (55 mg, 0.2 mmol), *m*-CPBA (53 mg, 0.24 mmol) and DCM (2 mL). The reaction was stirred at ambient temperature for 5 h. After completion of the reaction, as monitored using TLC, the mixture was washed with NaHCO_3_ solution and dried over MgSO_4_. After removal of the solvent in vacuo, the reaction mixture was purified via column chromatography on silica gel with PE and EA as eluent to afford product **5**.

#### ((1*R*,2*R*,4*S*,5*S*,6*R*,7*S*)-7-Phenyl-3-oxatricyclo[3.2.1.02,4]octan-6-yl)(pyridin-2-yl)methanone (**5**)

Colorless oil, 42 mg, yield 73%, R*_f_* = 0.3 (PE/EA = 10:1, *v*/*v*). **^1^H NMR** (400 MHz, CDCl_3_) δ 8.69 (dt, *J* = 4.8, 1.2 Hz, 1H), 8.10 (d, *J* = 7.8 Hz, 1H), 7.88 (td, *J* = 7.7, 1.7 Hz, 1H), 7.50 (ddd, *J* = 7.6, 4.8, 1.3 Hz, 1H), 7.32–7.27 (m, 4H), 7.24–7.16 (m, 1H), 4.47 (dd, *J* = 5.6, 3.8 Hz, 1H), 3.70 (d, *J* = 5.1 Hz, 1H), 3.47 (dd, *J* = 3.7, 1.4 Hz, 1H), 3.28–3.24 (m, 1H), 3.02 (d, *J* = 3.3 Hz, 1H), 2.85 (d, *J* = 1.7 Hz, 1H), 1.66–1.57 (s, 2H). **^13^C NMR** (101 MHz, CDCl_3_) δ 200.6, 153.2, 149.0, 143.9, 137.0, 128.5, 128.2, 128.1, 126.1, 122.2, 57.4, 51.8, 50.0, 43.3, 43.2, 42.8, 26.7. **HRMS** (ESI): *m/z* [M+H]^+^ calculated for C_19_H_18_NO_2_^+^ 292.1332, found 292.1337.

### 3.6. Synthesis and Characterization of Product ***6***


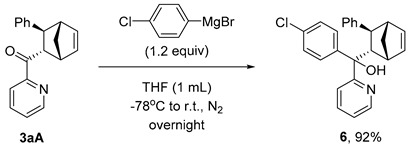


To an oven-dried reaction tube equipped with a magnetic stirring bar was added **3aA** (55 mg, 0.2 mmol). The tube was sealed immediately with a rubber stopper and protected with a nitrogen balloon by evacuation-backfill operations repeated three times. Dry THF (1 mL) and (4-chlorophenyl) magnesium bromide (58 μL, 1.2 equiv) was injected to the tube via a syringe at −78 °C. The mixture was stirred at 25 °C overnight. Water (3.0 mL) was added dropwise, and the mixture was dried over sodium sulfate and filtered. After removal of the solvent in vacuo, the reaction mixture was purified using column chromatography on silica gel with PE and EA as eluent to afford product **6**.

#### (4-Chlorophenyl)((1*R*,2*S*,3*S*,4*S*)-3-phenylbicyclo[2.2.1]hept-5-en-2-yl)(pyridin-2-yl)methanol (**6**)

Colorless oil, 91 mg, yield 92%, R*_f_* = 0.35 (PE/EA = 5:1, *v*/*v*). It is a mixture of three diastereoisomers; only the characteristic signals in ^1^H NMR are provided. **^1^H NMR** (400 MHz, CDCl_3_) **^1^H NMR** (400 MHz, chloroform-d) δ 8.50 (d, *J* = 4.5 Hz, 1H), 8.34 (d, *J* = 4.6 Hz, 1H), 8.24 (d, *J* = 4.7 Hz, 1H). **HRMS** (ESI): *m/z* [M+H]^+^ calculated for C_25_H_23_ClNO^+^ 388.1463, found 388.1465.

### 3.7. Synthesis and Characterization of Product ***7***


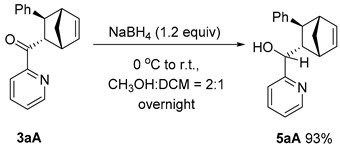


***Procedure***: To an oven-dried reaction tube equipped with a magnetic stirring bar was added **3aA** (55 mg, 0.2 mmol) and NaBH_4_ (53 mg, 0.24 mmol) in CH_3_OH-DCM (*v*/*v* = 2:1) (3 mL) at 0 °C. The reaction was stirred at ambient temperature overnight. Water (3.0 mL) was added dropwise, and the mixture was dried over sodium sulfate and filtered. After removal of the solvent in vacuo, the reaction mixture was purified using column chromatography on silica gel with PE and EA as eluent to afford product **7**.

#### (S)-((1*R*,2*S*,3*S*,4*S*)-3-Phenylbicyclo[2.2.1]hept-5-en-2-yl)(pyridin-2-yl)methanol (**7**)

Colorless oil, 51.5 mg, yield 93%, R*_f_* = 0.5 (PE/EA = 2:1, *v*/*v*). **^1^H NMR** (400 MHz, CDCl_3_) δ 8.38 (d, *J* = 4.6 Hz, 1H), 7.43 (td, *J* = 7.7, 1.8 Hz, 1H), 7.14–6.97 (m, 5H), 6.86 (dd, *J* = 6.8, 1.9 Hz, 2H), 6.44 (dd, *J* = 5.7, 3.1 Hz, 1H), 6.36 (dd, *J* = 5.6, 2.9 Hz, 1H), 4.26 (d, *J* = 9.3 Hz, 1H), 3.61 (s, 1H), 3.22 (s, 1H), 2.76 (s, 1H), 2.58 (ddd, *J* = 9.0, 5.4, 3.3 Hz, 1H), 2.35 (d, *J* = 5.0 Hz, 1H), 1.74 (d, *J* = 8.7 Hz, 1H), 1.52 (dd, *J* = 8.7, 1.8 Hz, 1H). **^13^C NMR** (101 MHz, CDCl_3_) δ 161.8, 148.6, 144.1, 138.4, 136.3, 135.0, 127.9, 127.4, 125.5, 122.4, 121.4, 54.2, 50.6, 47.7, 46.4, 44.8. **HRMS** (ESI): *m/z* [M+H]^+^ calculated for C_19_H_20_NO^+^ 278.1539, found 278.1535.

## 4. Conclusions

We have identified a chiral PyBox–La(OTf)_3_ complex for catalyzing enantioselective Diels–Alder cycloadditions of 2-alk-2-enoylpyridines with cyclopentadiene. The asymmetric reactions proceeded efficiently, displaying good levels of diastereo- and enantiocontrol (up to 92:8 dr and 99:1 er). Enantiopure disubstituted norbornenes, which possess four contiguous stereocenters and are biologically relevant structures, are produced conveniently in this way. Further manipulations of these structures were also demonstrated, yielding the more densely functionalized norbornene derivatives. We hope our catalytic protocol could benefit the synthetic and medicinal chemists who are associated with enantioenriched norbornenes.

## Figures and Tables

**Table 1:** Selected optimization of the reaction conditions.

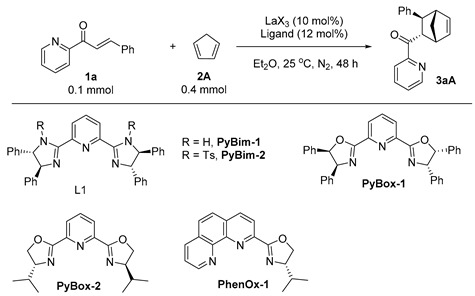					
Entry	Catalyst	Ligand	Yield ^a^ (%)	Er ^b^	Dr ^b^
1	La(OTf)_3_	**PyBim-1**	54	84:16	51:49
2	La(OTf)_3_	**PyBim-2**	67	85.5:14.5	79.5:20.5
3	La(OTf)_3_	**PyBox-1**	91	94:6	88:12
4	La(OTf)_3_	**PyBox-2**	95	68:32	89:11
5	La(OTf)_3_	**PhenOx-1**	87	58:42	86.5:13.5
6	LaCl_3_	**PyBox-1**	48	51.5:48.5	84:16
7	La(BF_4_)_3_	**PyBox-1**	71	51:49	89:11

^a^ Isolated yields on column chromatography. ^b^ Enantioselective and diastereoselective ratios obtained by chiral HPLC. dr = *endo*:*exo* ratios.

## Data Availability

Data are contained within the article.

## Supplementary Materials

The following supporting information can be downloaded at: https://www.mdpi.com/article/10.3390/molecules29132978/s1, Further results of reaction condition optimization, copies of ^1^H NMR, ^13^C NMR, ^19^F NMR and HPLC spectra of products and are included in the supporting information.
