# Supplementary material for: PyBox–La(OTf)3-Catalyzed Enantioselective Diels–Alder Cycloadditions of 2-Alkenoylpyridines with Cyclopentadiene
Source: Molecules. 2024 Jun 22;29(13):2978. doi: 10.3390/molecules29132978 (PMC11243330; doi:10.3390/molecules29132978)
Supplement: Supplementary file 1 [file molecules-29-02978-s001.zip › molecules-3023295-supplementary.pdf]

Supplementary Materials

PyBox–La(OTf)$_3$ catalysed enantioselective Diels-Alder cycloadditions of 2-alkenoylpyridines with cyclopentadiene

Hao Wei,[a] Yujie Zhang,[a] Sanlin Jin,[a] Ying Yu,*[b] Ning Chen,[a] Jiaxi Xu[a] and Zhanhui Yang *[a]

[a] *Department of Organic Chemistry, College of Chemistry, Beijing University of Chemical Technology, Beijing 100029, P. R. China. zhyang@mail.buct.edu.cn*

[b] *China United Test & Certification Co.,Ltd., Beijing 100088, P. R. China. yhb_5158@163.com*

Content

1. Experimental Sections ······ **S3**

1.1 Optimization of the reaction conditions. ······ S3

1.2 Gram scale reaction ······ S4

1.3 General procedure for reduction of Racemic Products 3 ······ S5

1.4 Methods for calculating the dr and er values ······ S6

2. Copies of Spectra of Products and Materials ······ **S8**

3. HPLC Spectra ······ **S46**

3.1 HPLC copies of compound ······ S46

3.2 HPLC copies of optimization of the reaction conditions. ······ S84

3.3 HPLC copies of gram scale reaction. ······ S135

1. Experimental Sections

1.1 Optimization of the reaction conditions.

Table S1. Screening of the metals with **PyBim-1** ligand

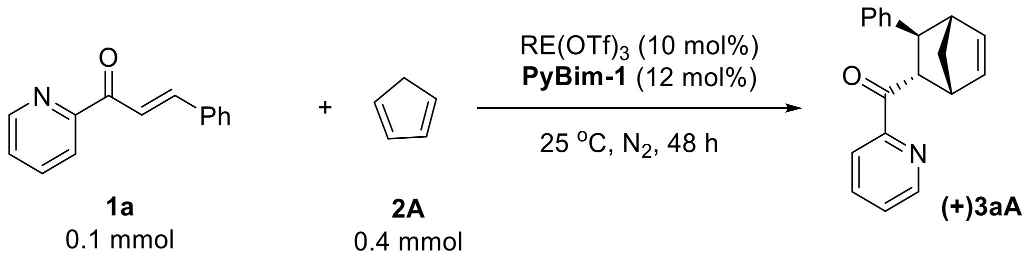

entry	catalyst	solvent (4 mL)	yield (%)[a]	er[b]	dr[b]
1	$La(OTf)_3$	DCM	42	65.5:34.5	51:49
2	$La(OTf)_3$[c]	DCM	54	72.5:27.5	25:75
3	$Pr(OTf)_3$	DCM	45	55.5:44.5	24:76
4	$Ce(OTf)_3$	DCM	14.5	67.5:32.5	42:58
5	$Sm(OTf)_3$	DCM	36	55:45	34:66
6	$Sc(OTf)_3$	DCM	84	50.5:49.5	13:87
7	$Yb(OTf)_3$	DCM	60	56.5:43.5	17:83
8	$Gd(OTf)_3$	DCM	62	52:48	32:68
9	$Y(OTf)_3$	DCM	51	51:49	34.5:65.5
10	$Lu(OTf)_3$	DCM	83	64:36	18:82
11	$Tb(OTf)_3$	DCM	47.3	51:49	33:67
12	$Ho(OTf)_3$	DCM	87.3	50.5:49.5	33.5:66.5
13	$Er(OTf)_3$	DCM	81.8	55:45	29:71
14	$La(OTf)_3$	Toluene	46	64:36	29:71
15	$La(OTf)_3$	DCE	66	80:20	33:67
16	$La(OTf)_3$	THF	75	84:16	22:78
17	$La(OTf)_3$[c]	THF[C]	53	84.5:15.5	24:76
18	$La(OTf)_3$	MeCN	45	67.5:32.5	34:67
19	$La(OTf)_3$	EA	56	83.5:16.5	27:73
20	$La(OTf)_3$	Et_2O	60	88:12	43.5:56.5
21	$La(OTf)_3$	$CHCl_3$	36	57:43	19:81
22	$La(OTf)_3$	CH_3COCH_3	45	75:25	36:64

[a]Isolated yields on column chromatography. [b]Enantioselective and diastereoselective ratio obtained by chiral HPLC

[c]Reaction at 0 °C

Table S2. Screening of the metals with **PyBox-1** ligand

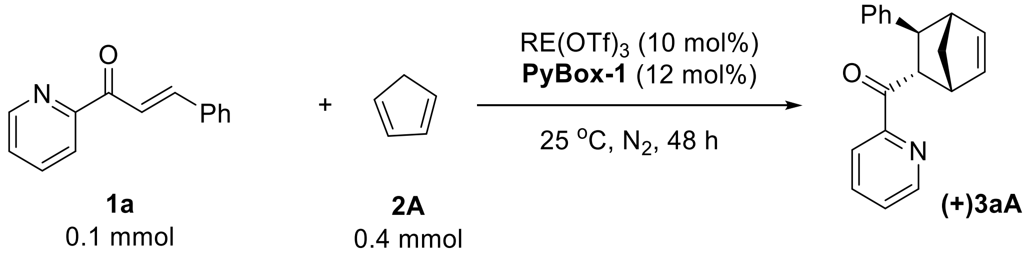

entry	catalyst	solvent (2 mL)	yield (%)[a]	er[b]	dr[b]
1	$La(OTf)_3$	THF	91	94:6	88:12
2	$Pr(OTf)_3$	THF	84	94:6	15:85
3	$Ce(OTf)_3$	THF	76	94:6	14:86
4	$Sm(OTf)_3$	THF	98	94:6	21:79
5	$Sc(OTf)_3$	THF	33	57:43	19:81
6	$Yb(OTf)_3$	THF	80	90:10	29:71
7	$Y(OTf)_3$	THF	87	90:10	29:71
8	$Lu(OTf)_3$	THF	91	50:50	14:86
9	$Tb(OTf)_3$	THF	76	91.5:8.5	27:73
10	$Ho(OTf)_3$	THF	69	89.5:10.5	31:69
11	$Er(OTf)_3$	THF	87	89:11	31:69
12	$Tm(OTf)_3$	THF	87	89.5:10.5	30:70
13	$Eu(OTf)_3$	THF	84	92:8	24:86
14	$Gd(OTf)_3$	THF	91	91.5:8.5	26:74
15	$La(OTf)_3$	THF (4 mL)	91	93.5:6.5	14.5:85.5
16	$La(OTf)_3$	Et_2O	91	94.5:5.5	11.5:85.5
17	$La(OTf)_3$	DCE:THF=1:1	76	92.5:7.5	15:85
18	$La(OTf)_3$	DCE:THF=1:3	58	93:7	14:86
19	$La(OTf)_3$	CH_3COCH_3:THF=1:1	66	82:18	19:81
20	$La(OTf)_3$	MeCN:THF=1:1	66	87:13	23:77
21	$La(OTf)_3$	Et_2O:THF=1:1	84	94:6	11:89

[a]Isolated yields on column chromatography. [b]Enantioselective and diastereoselective ratio obtained by chiral HPLC

1.2 Gram scale reaction

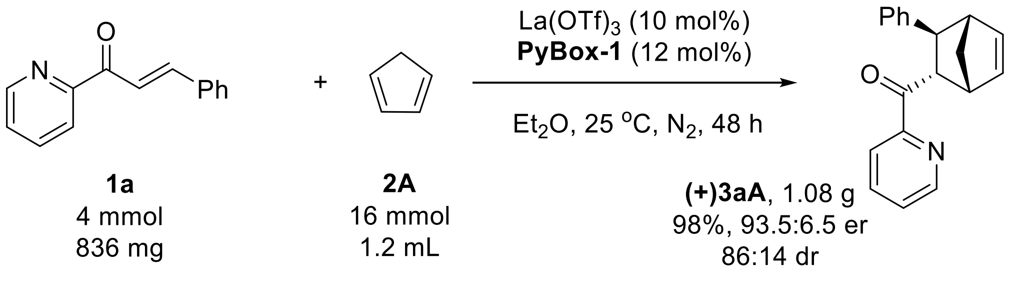

Procedure: To an oven-dried round-bottom flask equipped with a magnetic stirring bar was added **1a** (4 mmol, 836 mg), $La(OTf)_3$ (236 mg, 0.4 mmol) and ligand **PyBox-1** (252 mg, 0.48 mmol). The flask was sealed immediately with a rubber stopper and protected with a nitrogen balloon by evacuation-backfill operations for three times. Dry Et_2O (30 mL) was injected to the tube via a syringe, stirring for about 1 h, followed by addition of cyclopentadiene **2A** (1.2 mL, 16 mmol, 4.0 equiv) via a microsyringe. The mixture was stirred in 25 °C for 48 h. The solvent was evaporated under reduced pressure, and the crude mixture was subjected to column chromatography on silica gel to afford the corresponding products.

1.3 General procedure for reduction of Racemic Products 3

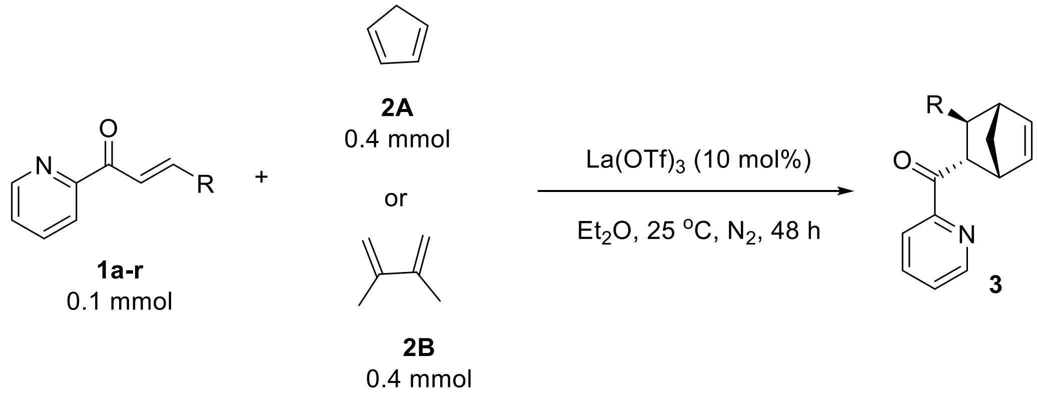

General Procedure: To an oven-dried reaction tube equipped with a magnetic stirring bar was added 2-alkenoyl pyridines **1** (0.1 mmol, 1.0 equiv), $La(OTf)_3$ (5.9 mg, 0.01 mmol) and ligand **PyBox-1** (6.3 mg, 0.012 mmol). The tube was sealed immediately with a rubber stopper and protected with a nitrogen balloon by evacuation-backfill operations for three times. Dry Et_2O (1 mL) was injected to the tube via a syringe. The resultant mixture was stirred for about 1

h, followed by addition of cyclopentadiene (**2A**) (33 μL, 0.4 mmol, 4.0 equiv) or 2,3-dimethylbuta-2,3-diene (**2B**) (90 μL, 0.4 mmol, 4.0 equiv) via a microsyringe. The mixture was stirred in 25 °C for 48 h. The solvent was evaporated under reduced pressure, and the crude mixture was subjected to column chromatography on silica gel to afford the corresponding products.

1.4 Methods for calculating the er and dr values

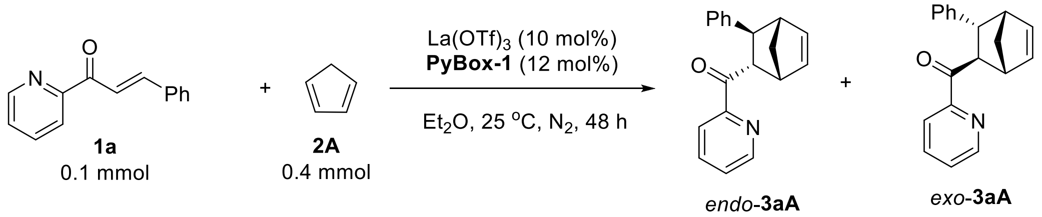

The dr and er values were determined by HPLC by referring to Lin and Feng's work (Lu, Y.; Zhou, Y.; Lin, L.; Zheng, H.; Fu, K.; Liu, X.; Feng, X. *Chem. Commun.* **2016,** 52, 8255-8258.). The absolute configuration of the endo-products was assigned by comparing our HPLC spectra and specific rotations with Lin and Feng's.

Taking the reaction of **1a** and **2A** producing **3aA** as an example, the peaks at 17.172 and 12.070 min corresponds to a pair of endo-enantiomers, and those at 9.708 and 11.103 min correspond to two *exo*-enantiomers. This statement is also verified by the HPLC spectrum of racemic product (±)-3aA, which was provided in Section 3.1 in the Supplementary Materials. Therefore, the dr and er values of the product come as follows:

er of *endo*-**3aA** = (area% of peak4) / (area% of peak3) = 82.3019/5.3146 = 94:6

dr = (sum of area% of peak3 and peak4) / (sum of area% of peak1 and peak2)

= (5.3146 + 82.3019)/(2.4584 + 9.9251) = 88:12.

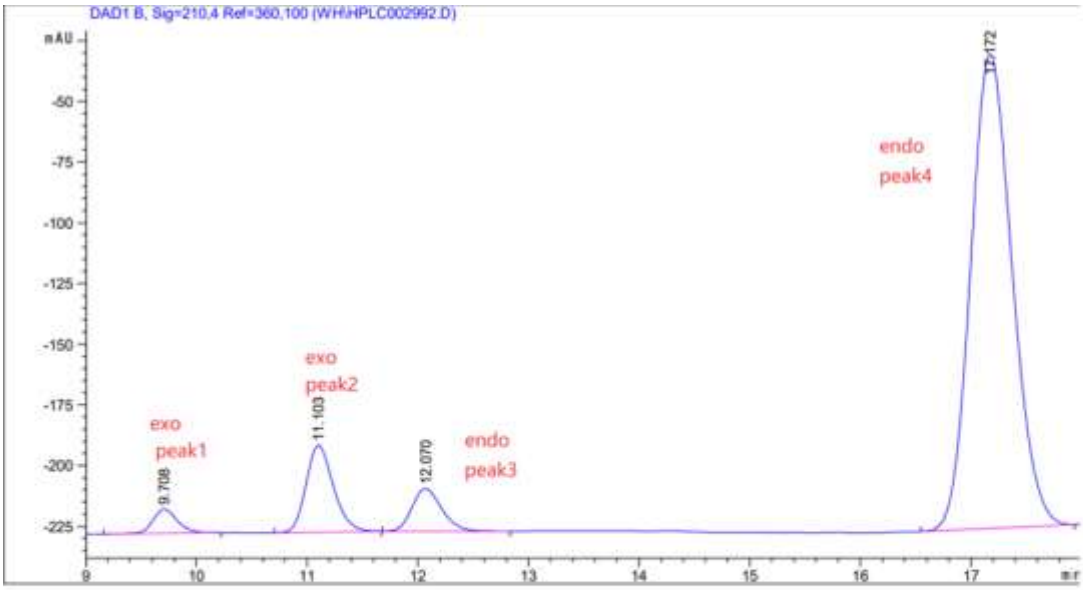

Peak #	Ret. Time [min]	Type	Width [min]	Area [mAU*s]	Height [mAU]	Area %
1	9.708	BB	0.2344	151.78452	9.92730	2.4584
2	11.103	BB	0.2641	612.79163	35.72356	9.9251
3	12.070	BB	0.2888	328.13419	17.49356	5.3146
4	17.172	BB	0.4035	5081.45313	195.32072	82.3019

With the same method, the dr and er values of other chiral products can also be calculated.

2. Copies of Spectra of Products and Materials

(*E*)-3-(2,3-difluorophenyl)-1-(pyridin-2-yl)prop-2-en-1-one (1d)

^{1}H NMR (400 MHz, $CDCl_3$)

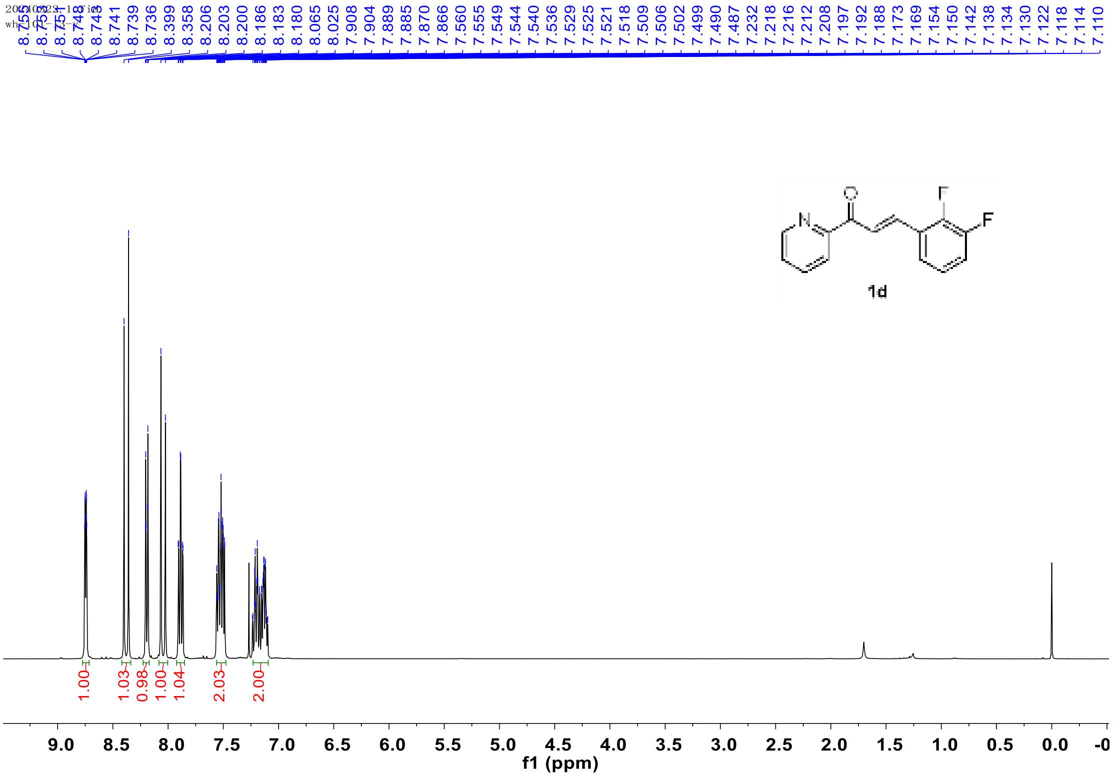

^{13}**C NMR** (101 MHz, $CDCl_3$)

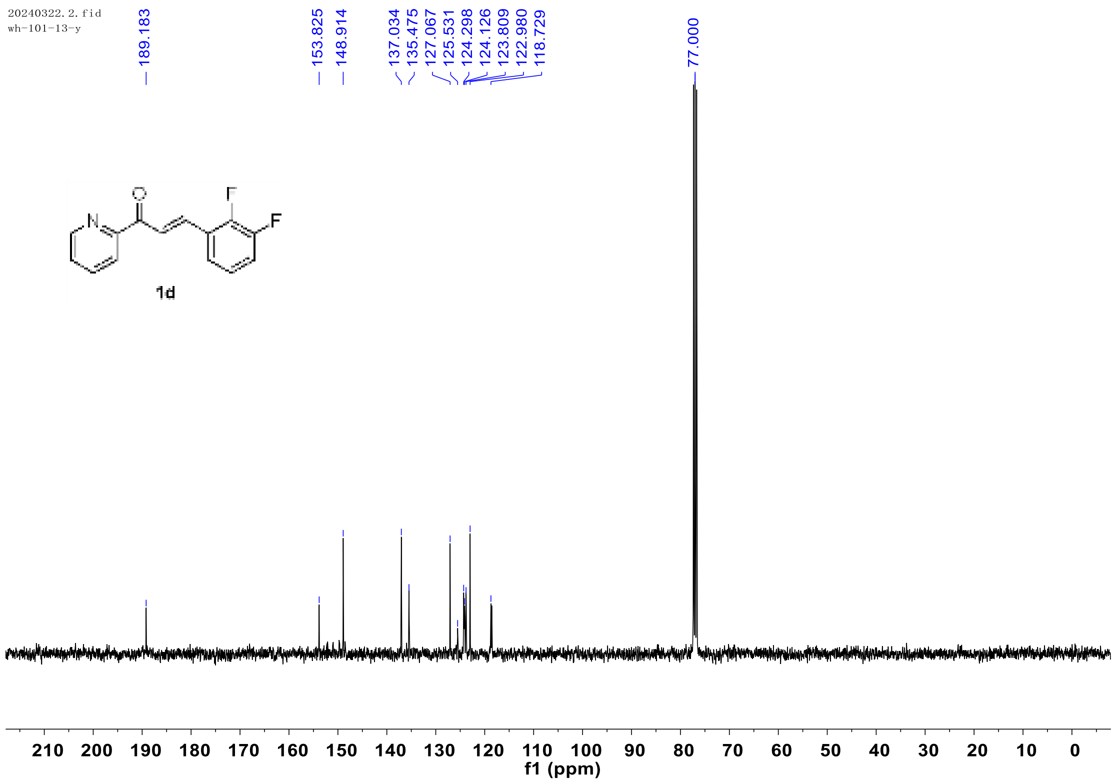

^{19}**F NMR** (377 MHz, $CDCl_3$)

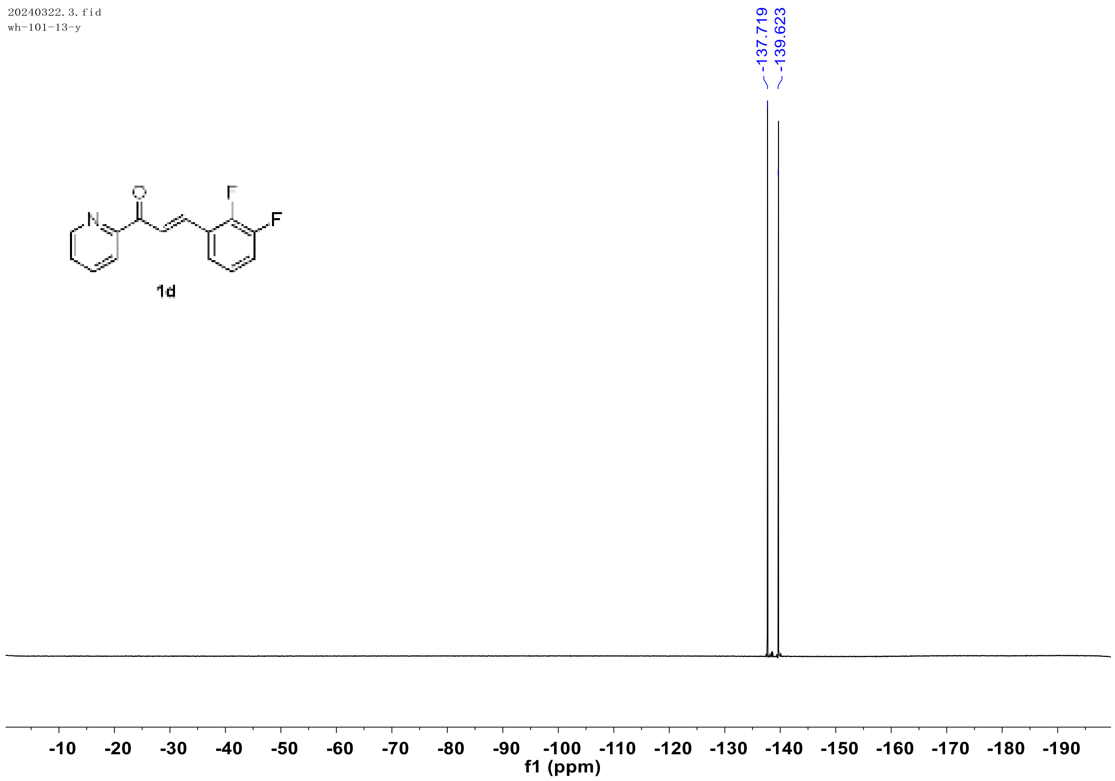

HRMS (ESI)

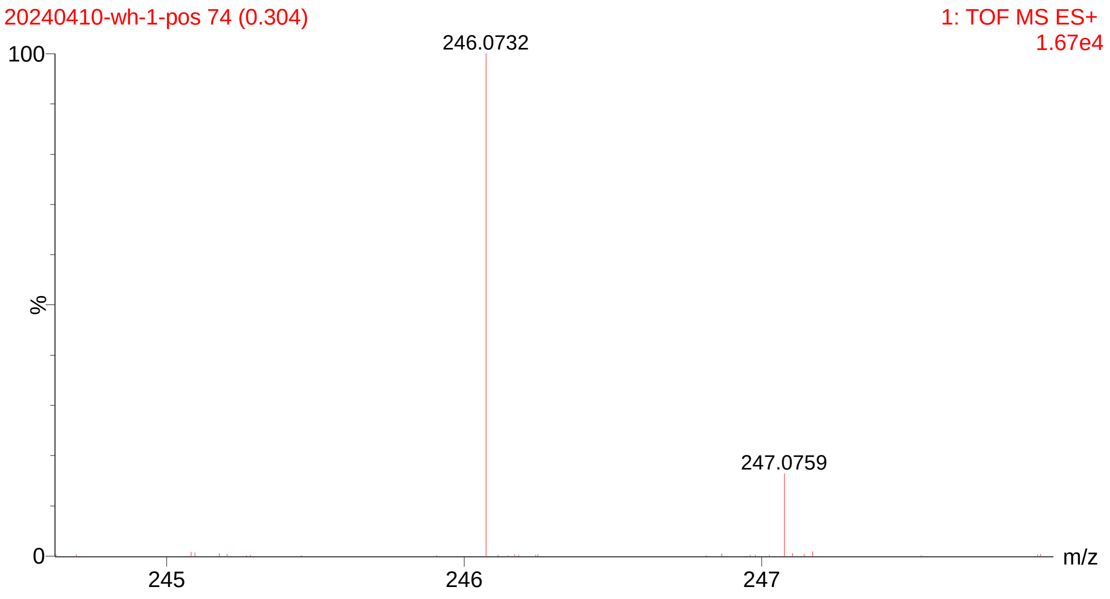
20240410-wh-1-pos 74 (0.304)
1: TOF MS ES+
1.67e4
246.0732
247.0759
100
%
0
245
246
247
m/z

(*E*)-3-cyclopentyl-1-(pyridin-2-yl)prop-2-en-1-one (1q)

^{1}H NMR (400 MHz, $CDCl_3$)

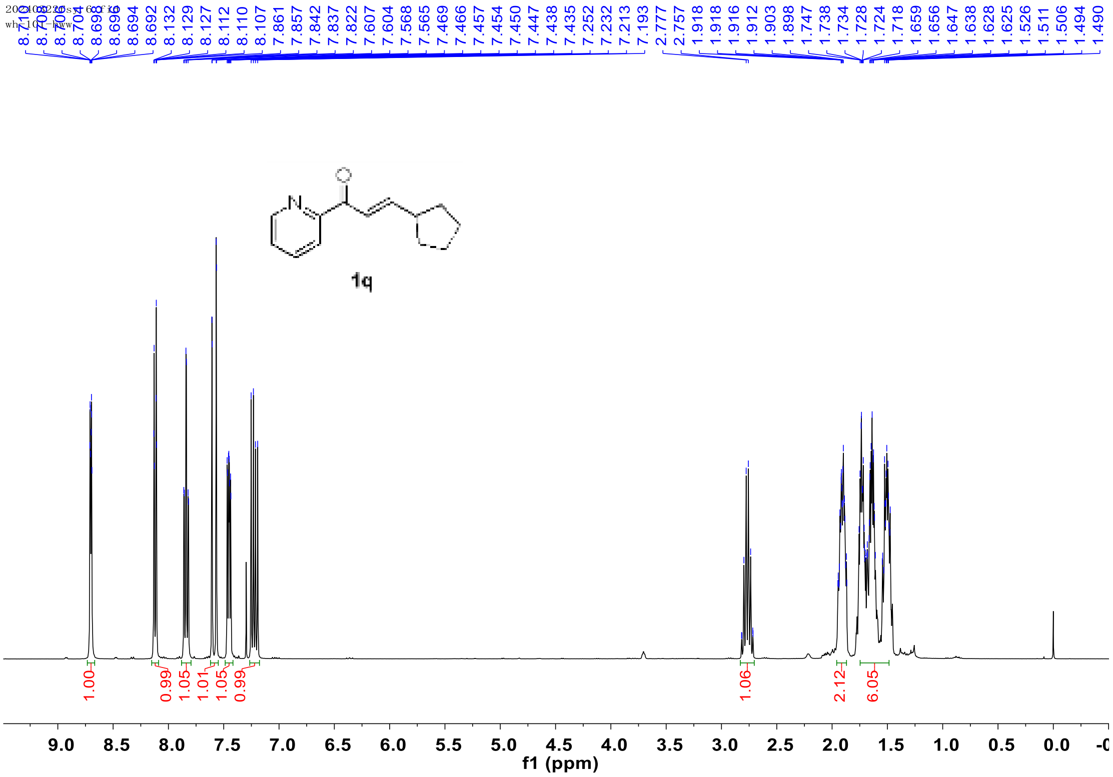

^{13}C NMR (101 MHz, $CDCl_3$)

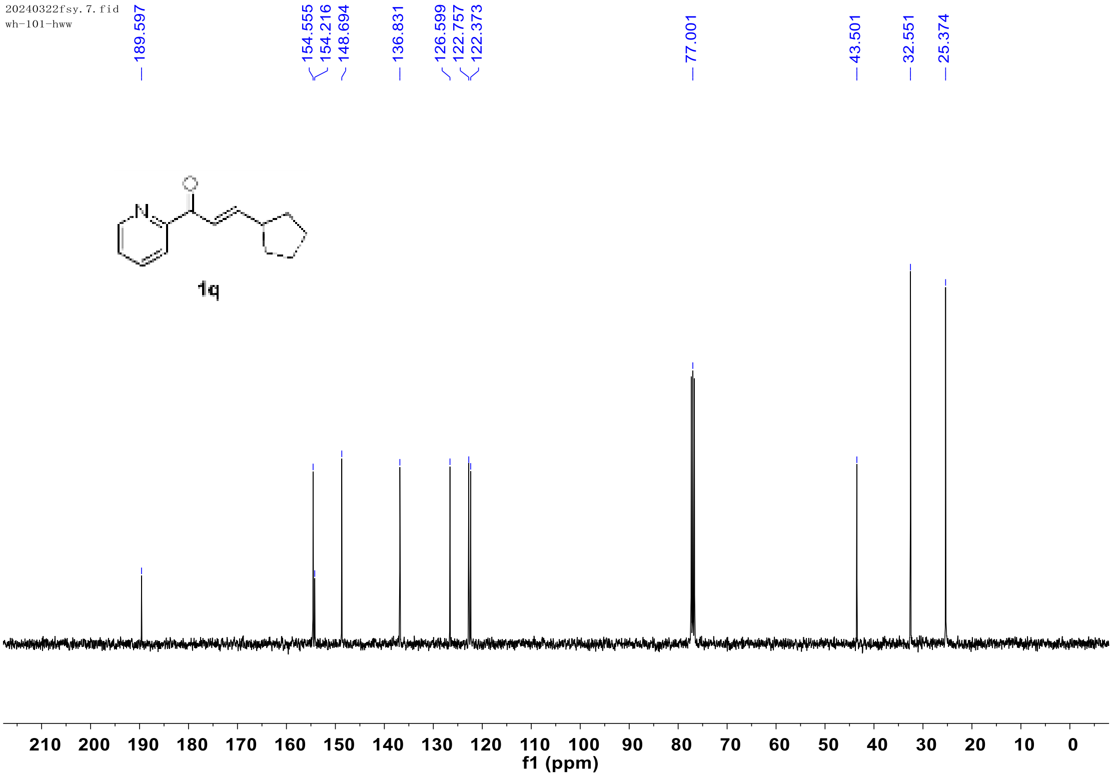

HRMS (ESI)

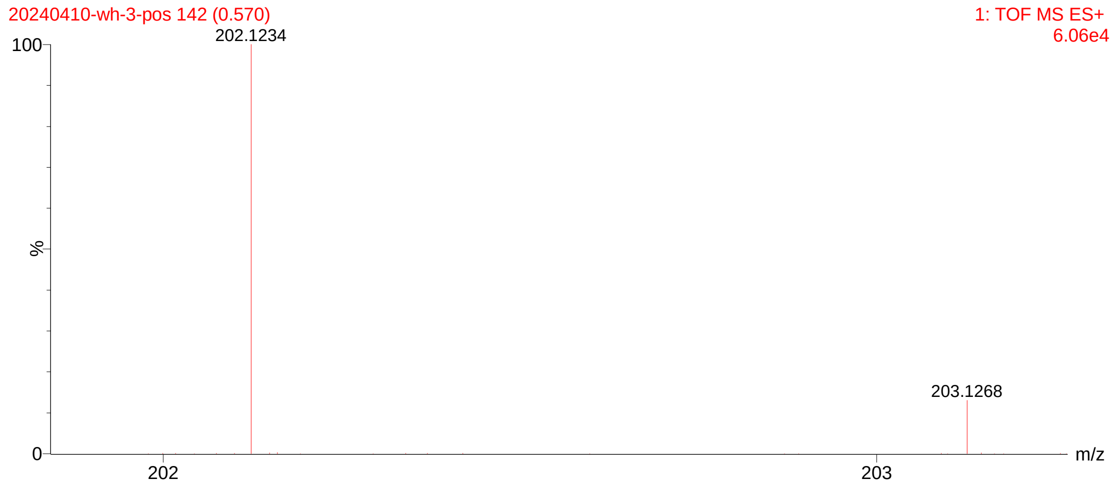

20240410-wh-3-pos 142 (0.570)
1: TOF MS ES+
6.06e4
202.1234
203.1268
100
%
0
202
203
m/z

((1*R*,2*S*,3*S*,4*S*)-3-phenylbicyclo[2.2.1]hept-5-en-2-yl)(pyridin-2-yl)methanone (+3aA)

^{1}H NMR (400 MHz, CDCl$_3$)

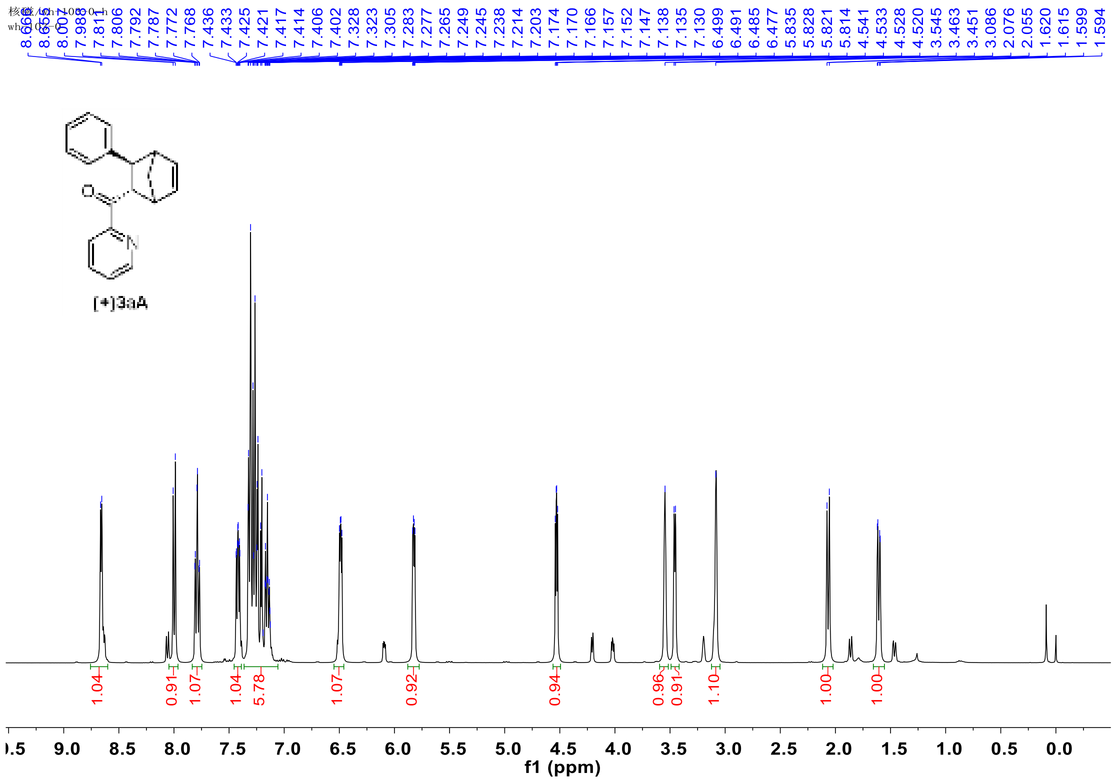

^{13}C NMR (101 MHz, CDCl$_3$)

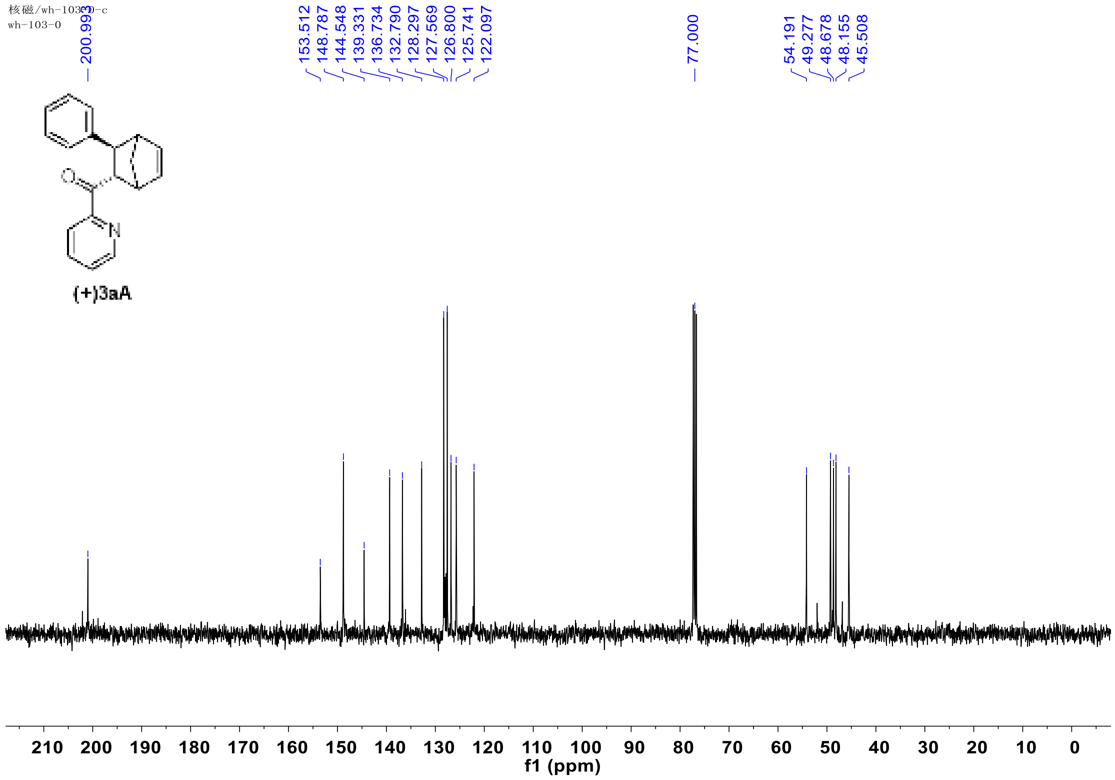

((1*R*,2*S*,3*S*,4*S*)-3-(2-fluorophenyl)bicyclo[2.2.1]hept-5-en-2-yl)(pyridin-2-yl)methanone (+3bA)

^{1}H NMR (400 MHz, $CDCl_3$)

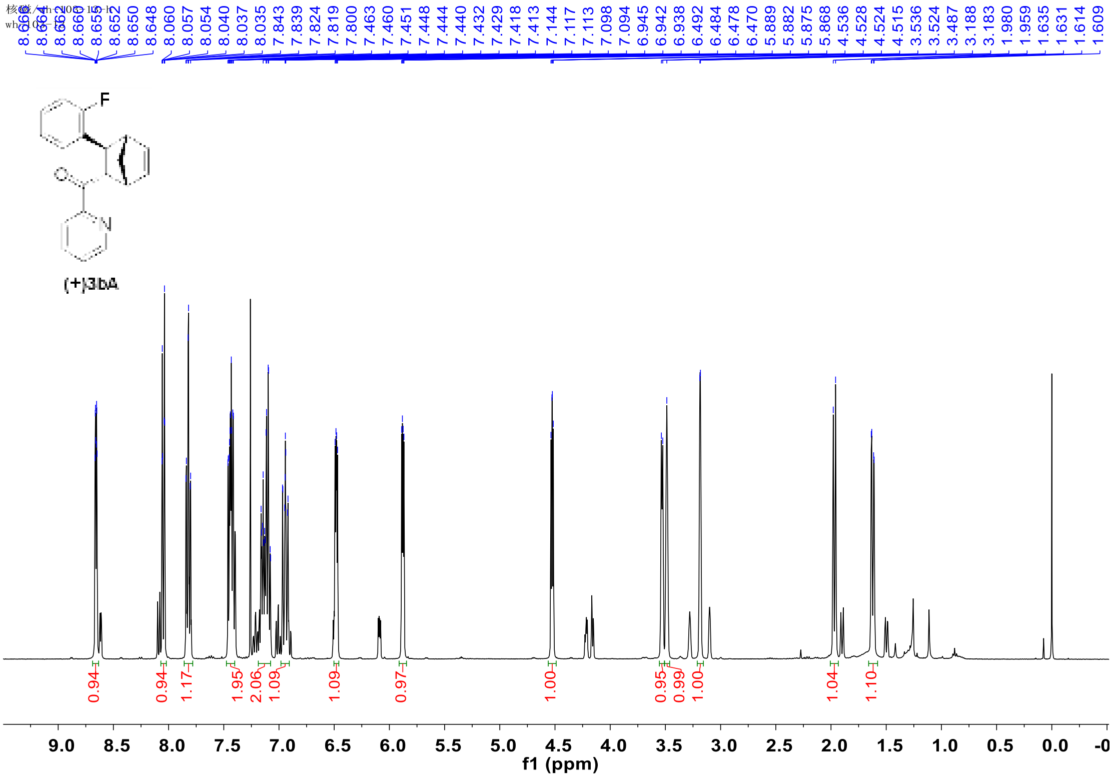

^{13}C NMR (101 MHz, $CDCl_3$)

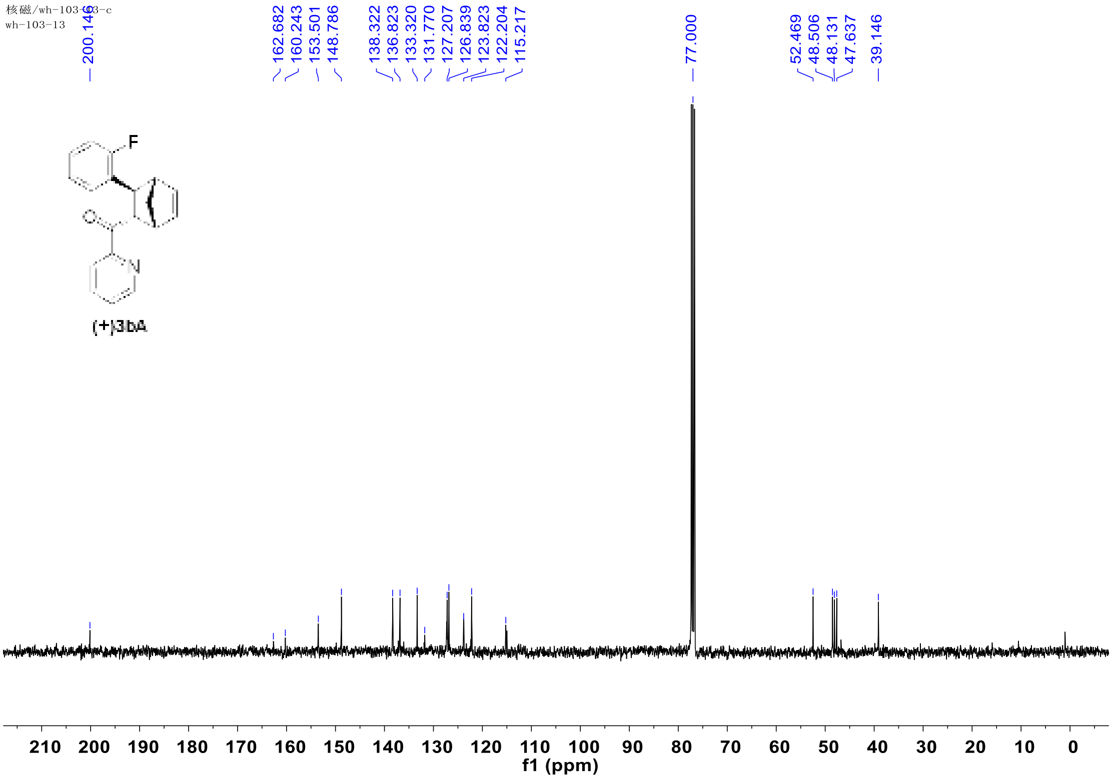

^{19}F NMR (377 MHz, $CDCl_3$)

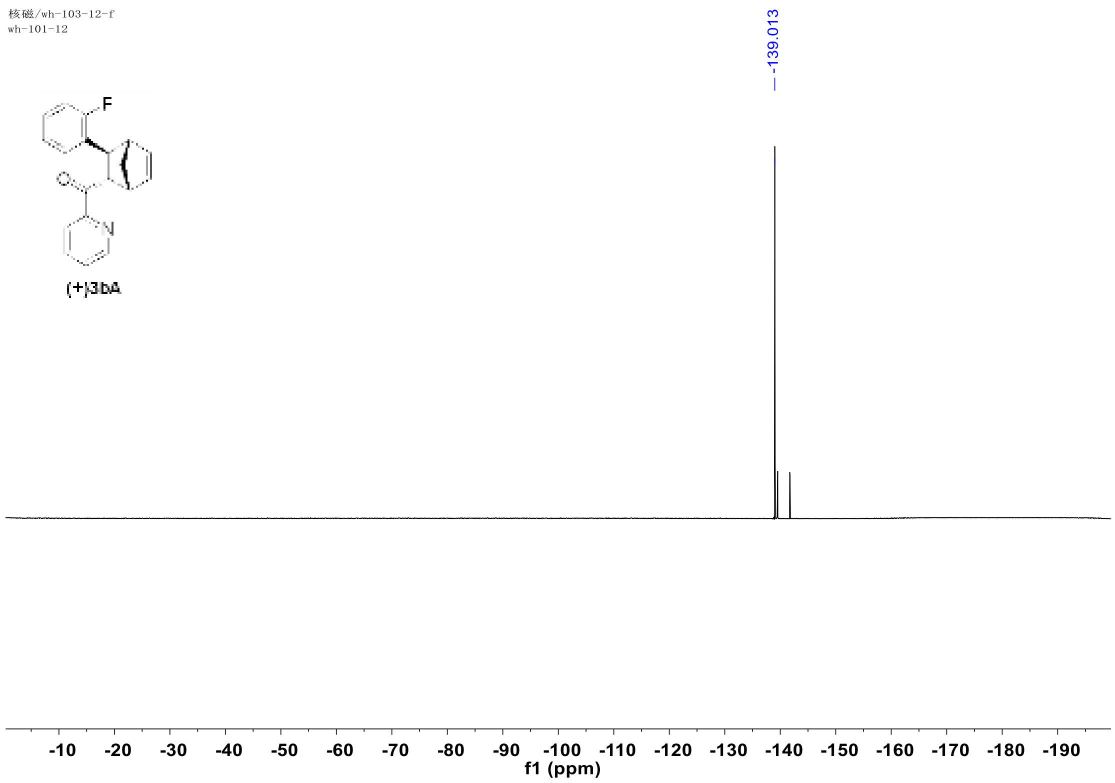

HRMS (ESI)

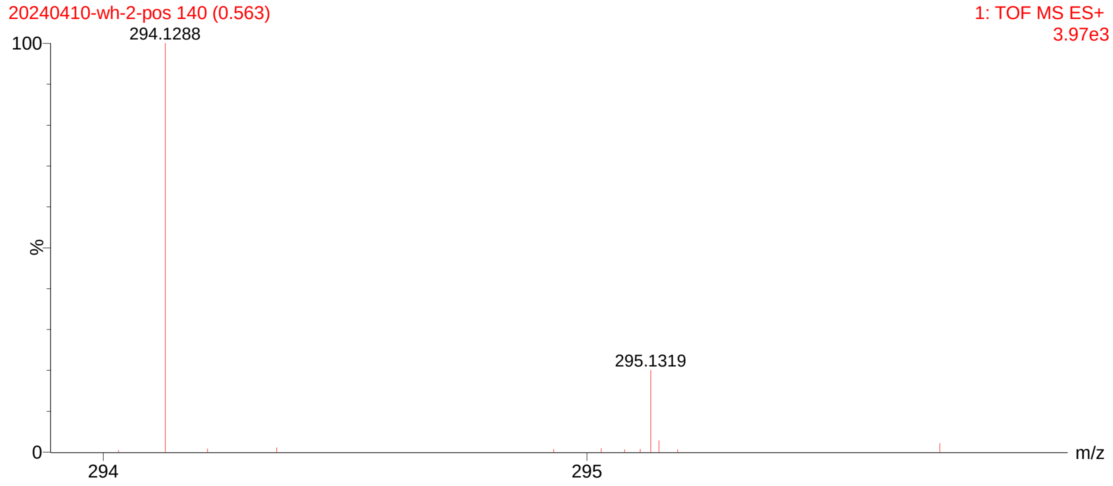

((1*R*,2*S*,3*S*,4*S*)-3-(4-fluorophenyl)bicyclo[2.2.1]hept-5-en-2-yl)(pyridin-2-yl)methanone (+3cA)

^{1}H NMR (400 MHz, $CDCl_3$)

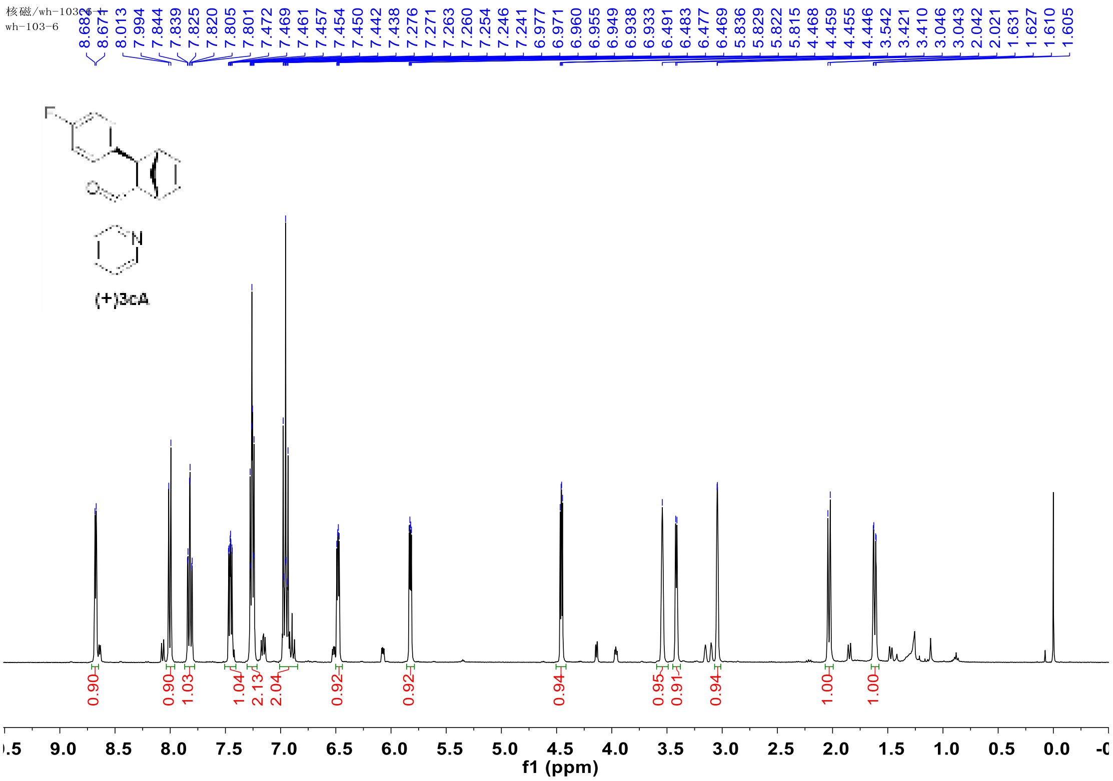

^{13}C NMR (101 MHz, $CDCl_3$)

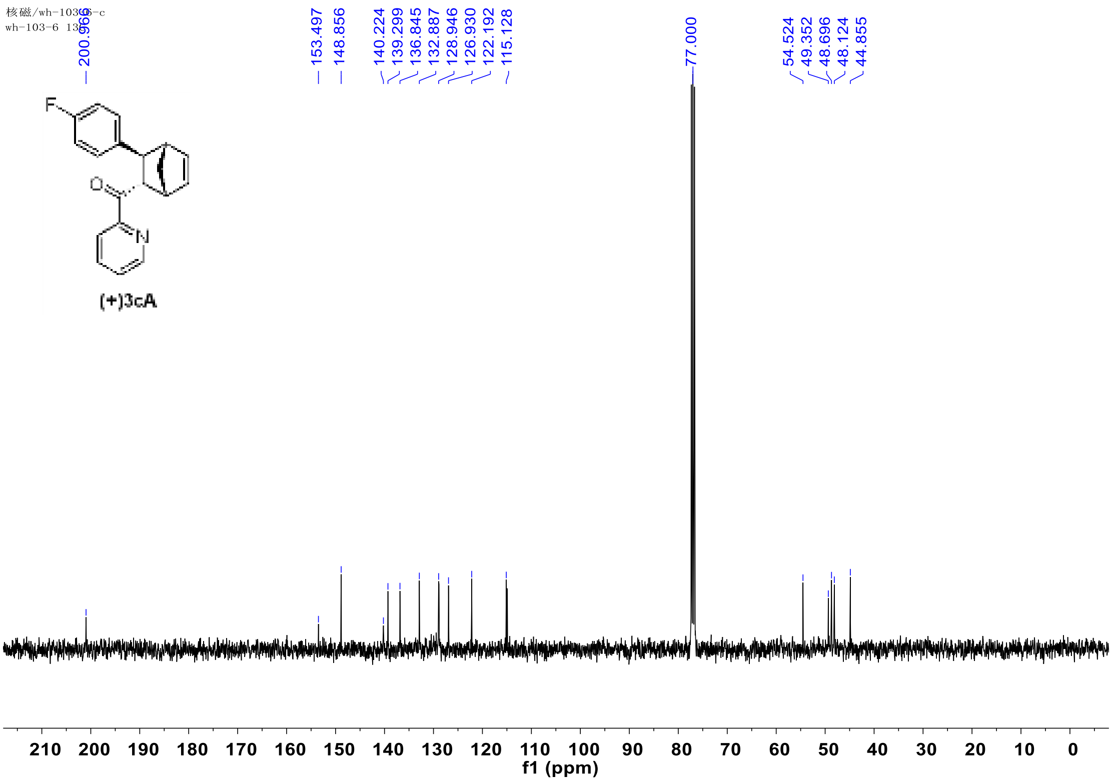

^{19}F NMR (377 MHz, $CDCl_3$)

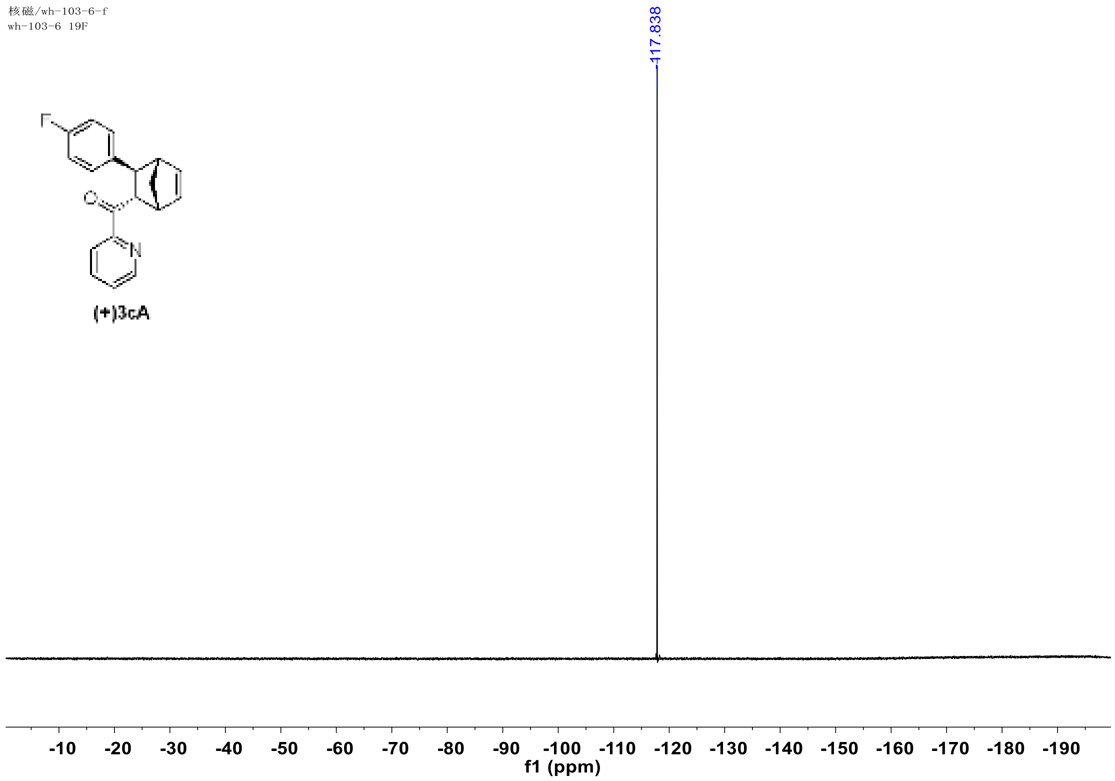

((1*R*,2*S*,3*S*,4*S*)-3-(2,3-difluorophenyl)bicyclo[2.2.1]hept-5-en-2-yl)(pyridin-2-yl)methanone (+3dA)

^{1}H NMR (400 MHz, CDCl3)

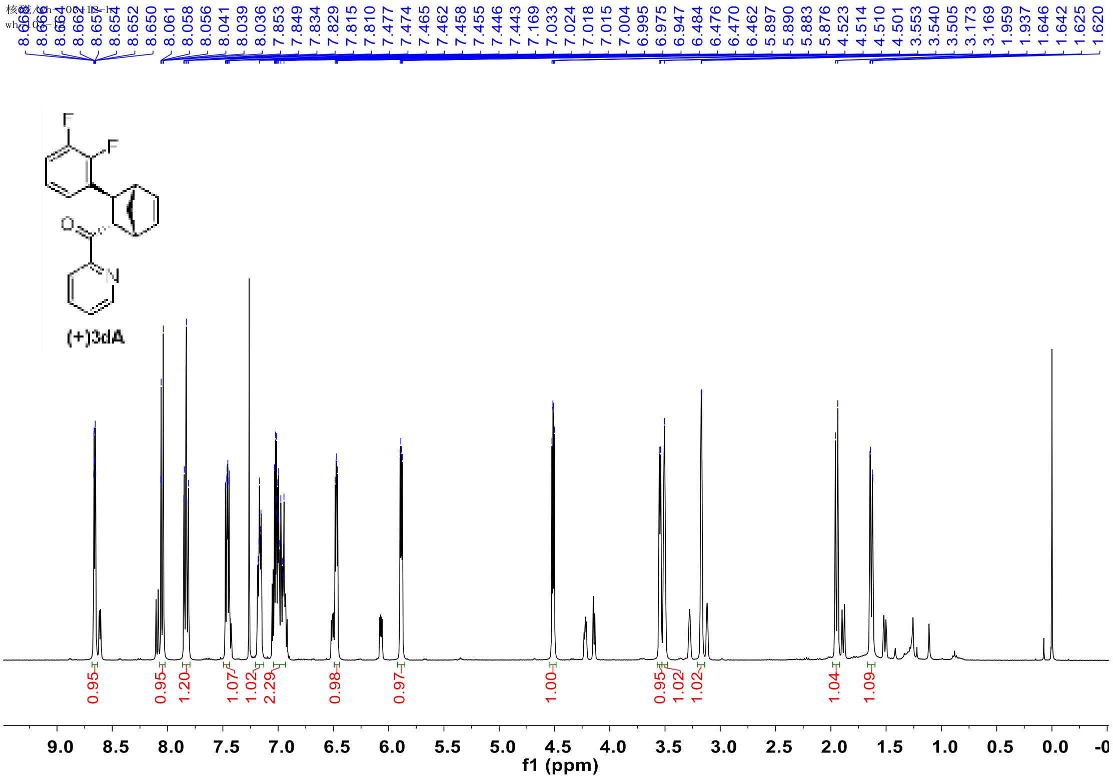

^{13}C NMR (101 MHz, CDCl3)

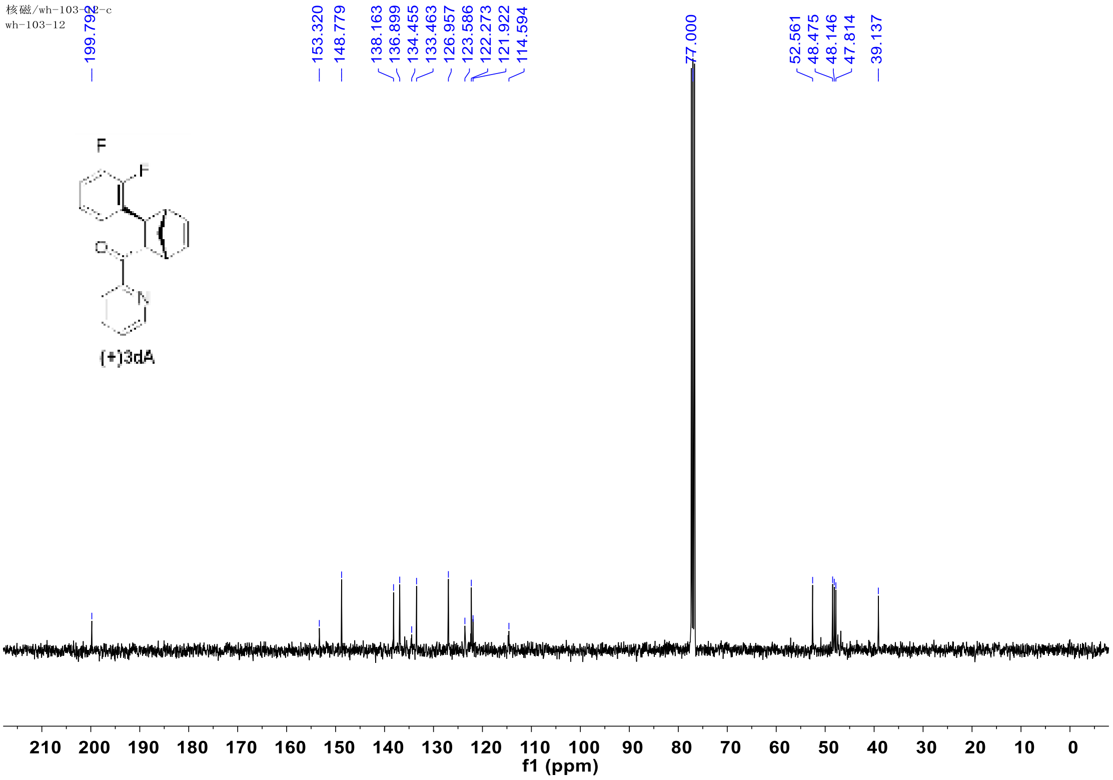

^{19}F NMR (377 MHz, $CDCl_3$)

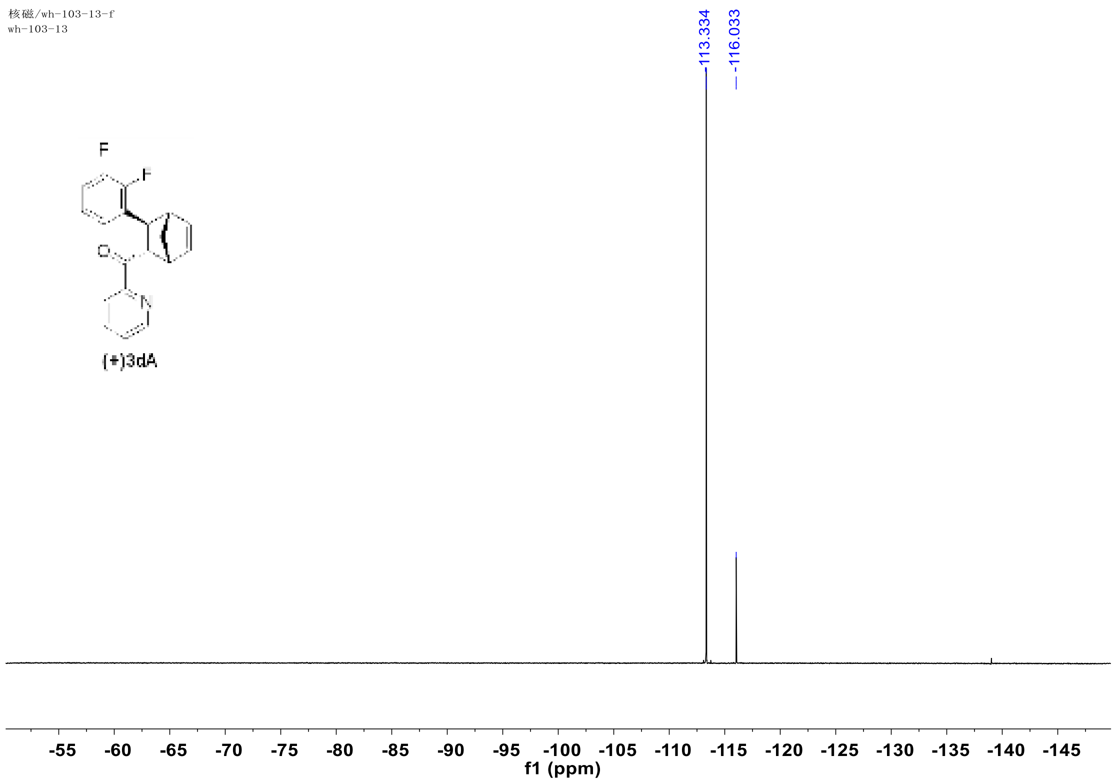

HRMS (ESI)

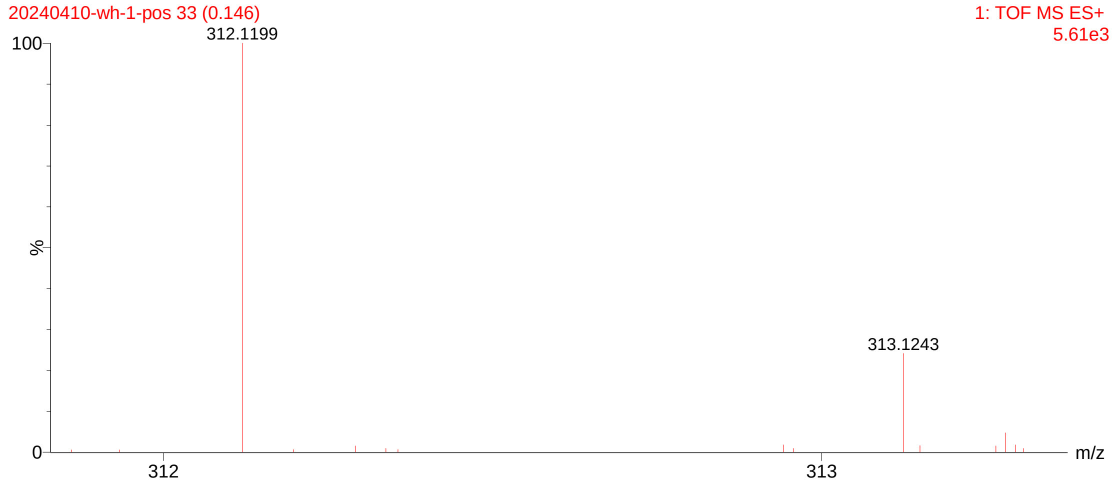

((1*S*,2*R*,3*R*,4*R*)-3-(3-chlorophenyl)bicyclo[2.2.1]hept-5-en-2-yl)(pyridin-2-yl)methanone (+3eA)

^{1}H NMR (400 MHz, $CDCl_3$)

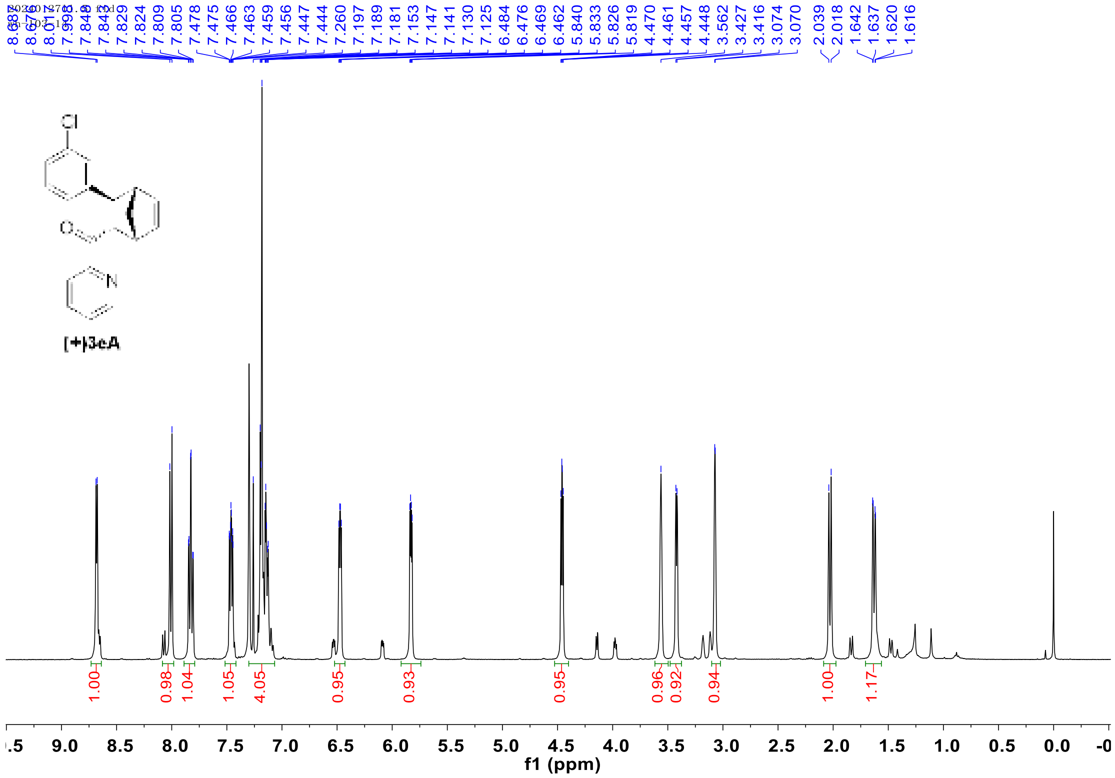

^{13}C NMR (101 MHz, $CDCl_3$)

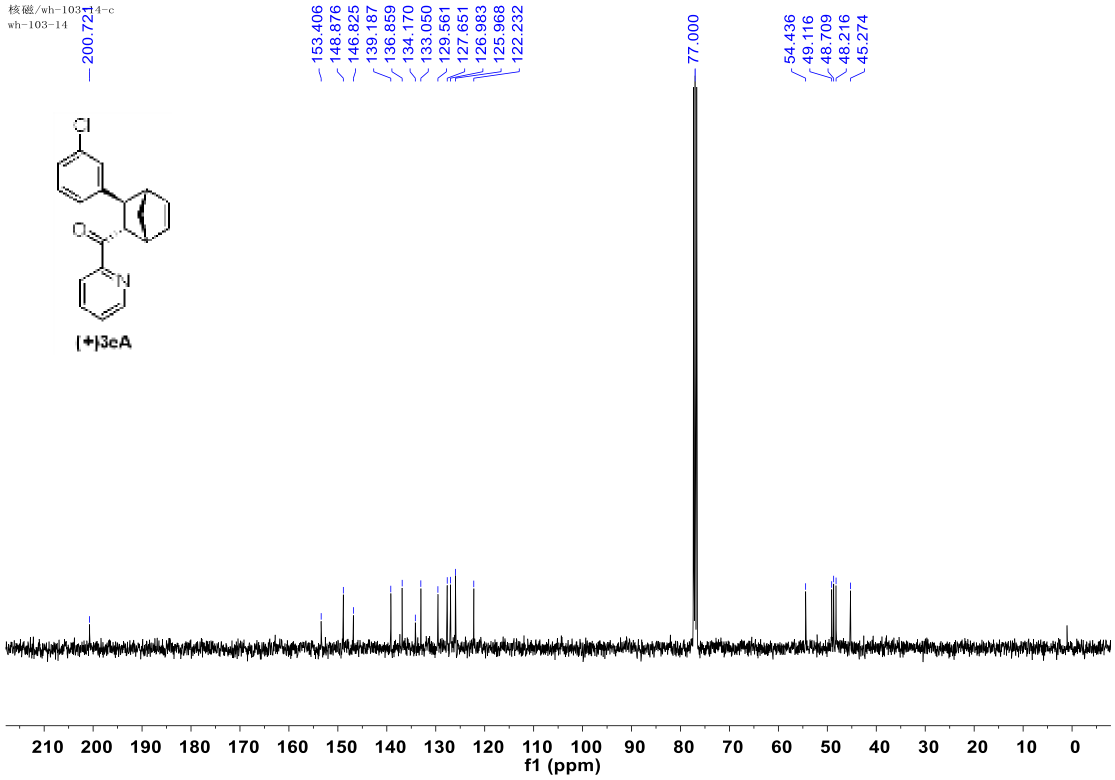

((1*R*,2*S*,3*S*,4*S*)-3-(4-chlorophenyl)bicyclo[2.2.1]hept-5-en-2-yl)(pyridin-2-yl)methanone (+3fA)

^{1}H NMR (400 MHz, $CDCl_3$)

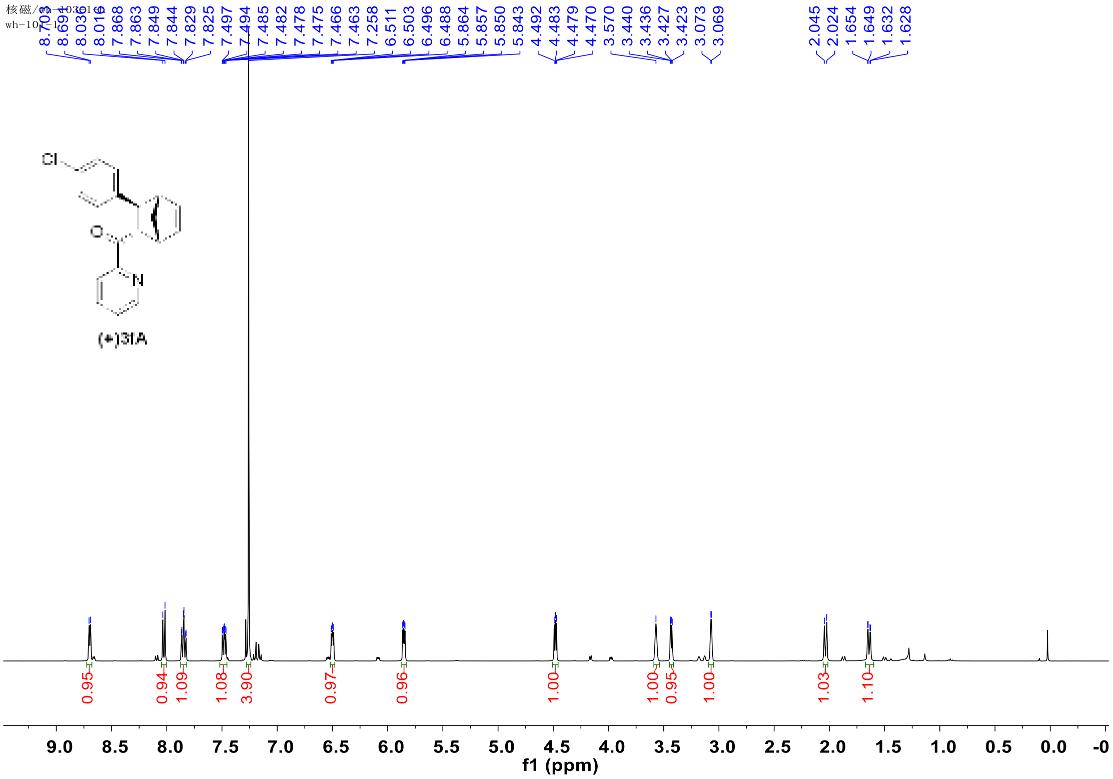

^{13}C NMR (101 MHz, $CDCl_3$)

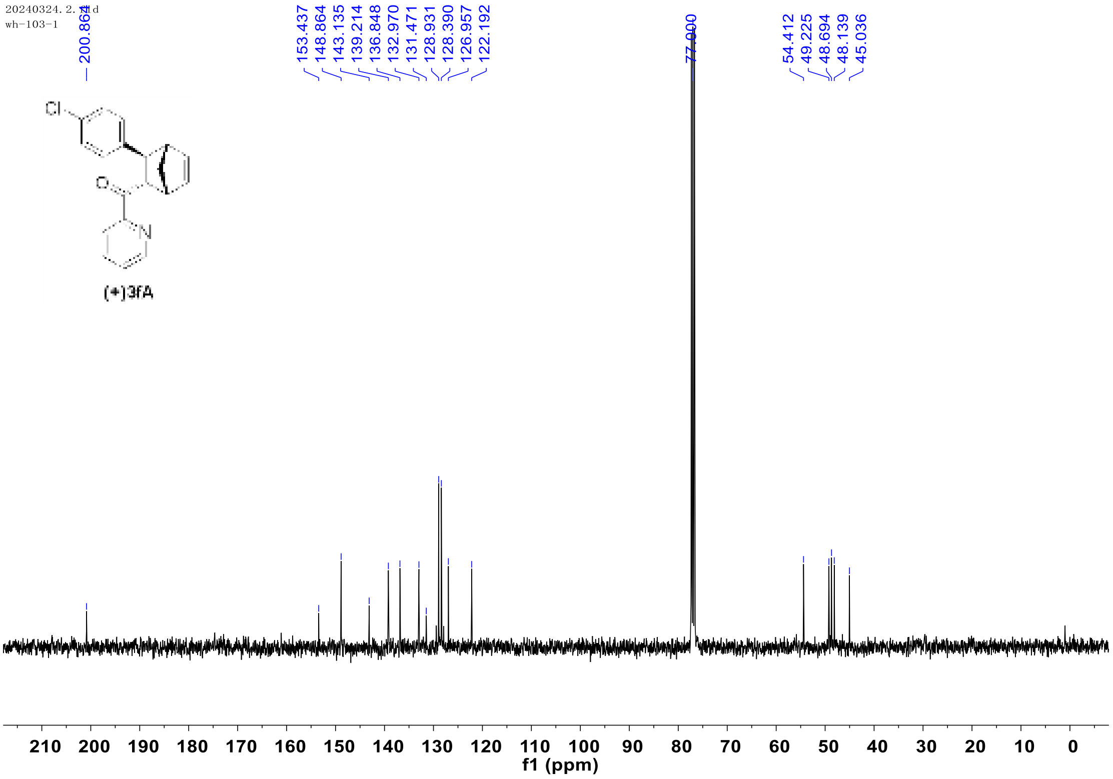

((1*R*,2*S*,3*S*,4*S*)-3-(2-bromophenyl)bicyclo[2.2.1]hept-5-en-2-yl)(pyridin-2-yl)methanone (+3gA)

^{1}H NMR (400 MHz, $CDCl_3$)

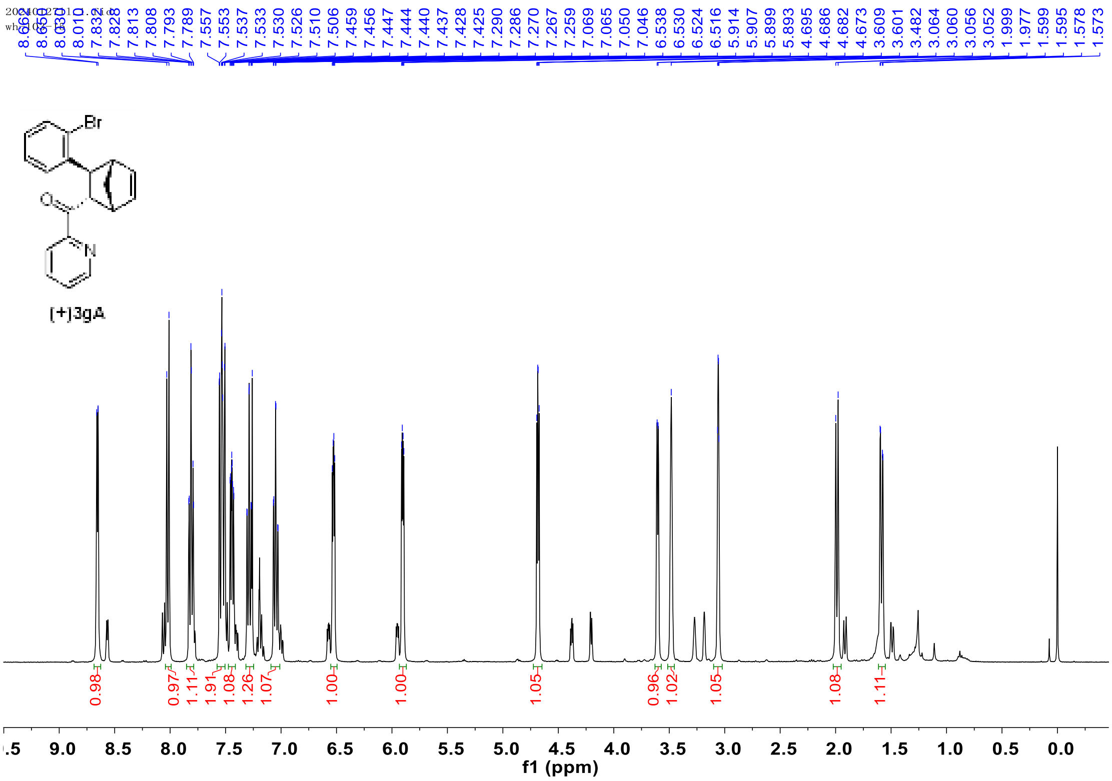

^{13}C NMR (101 MHz, $CDCl_3$)

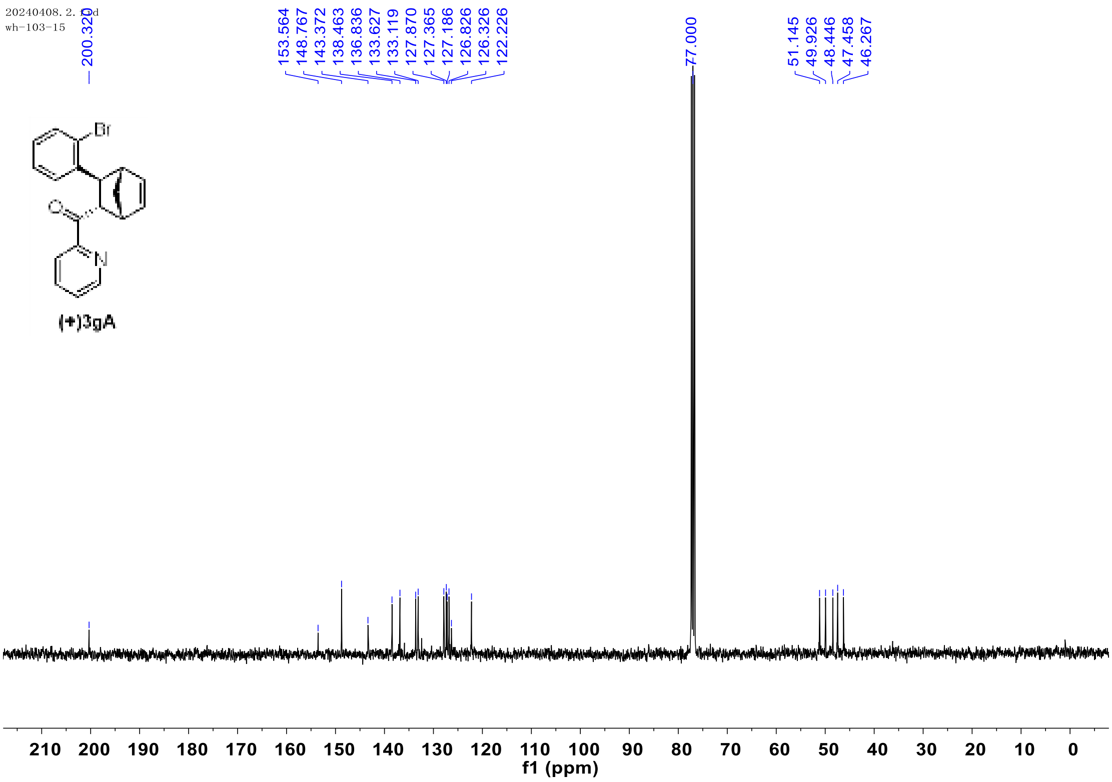

((1*R*,2*S*,3*S*,4*S*)-3-(4-bromophenyl)bicyclo[2.2.1]hept-5-en-2-yl)(pyridin-2-yl)methanone (+3hA)

^{1}H NMR (400 MHz, $CDCl_3$)

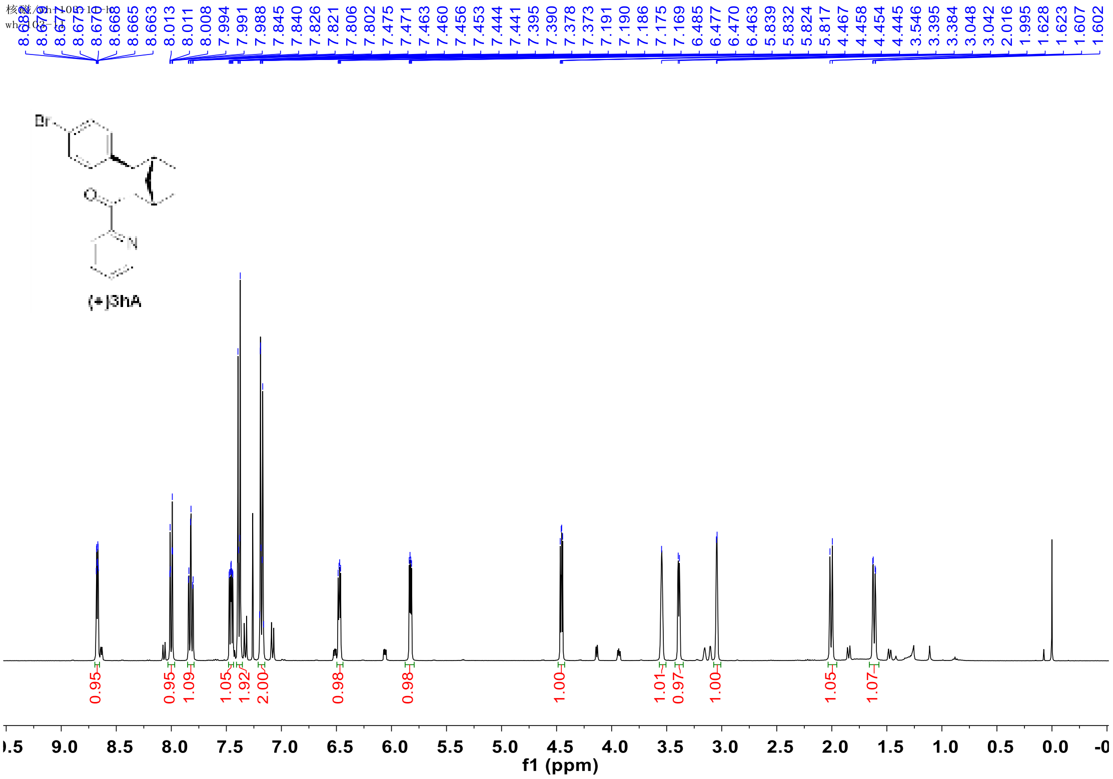

^{13}C NMR (101 MHz, $CDCl_3$)

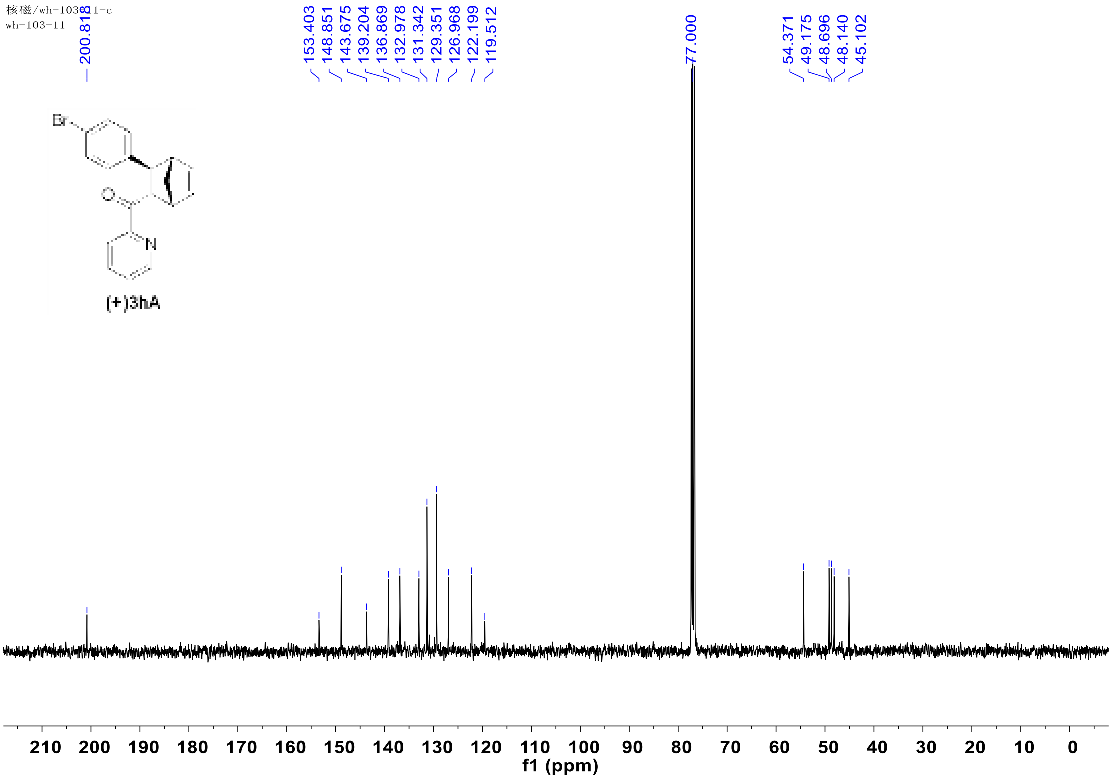

4-((1*S*,2*S*,3*S*,4*R*)-3-picolinoylbicyclo[2.2.1]hept-5-en-2-yl)benzonitrile (+3iA)

^{1}H NMR (400 MHz, $CDCl_3$)

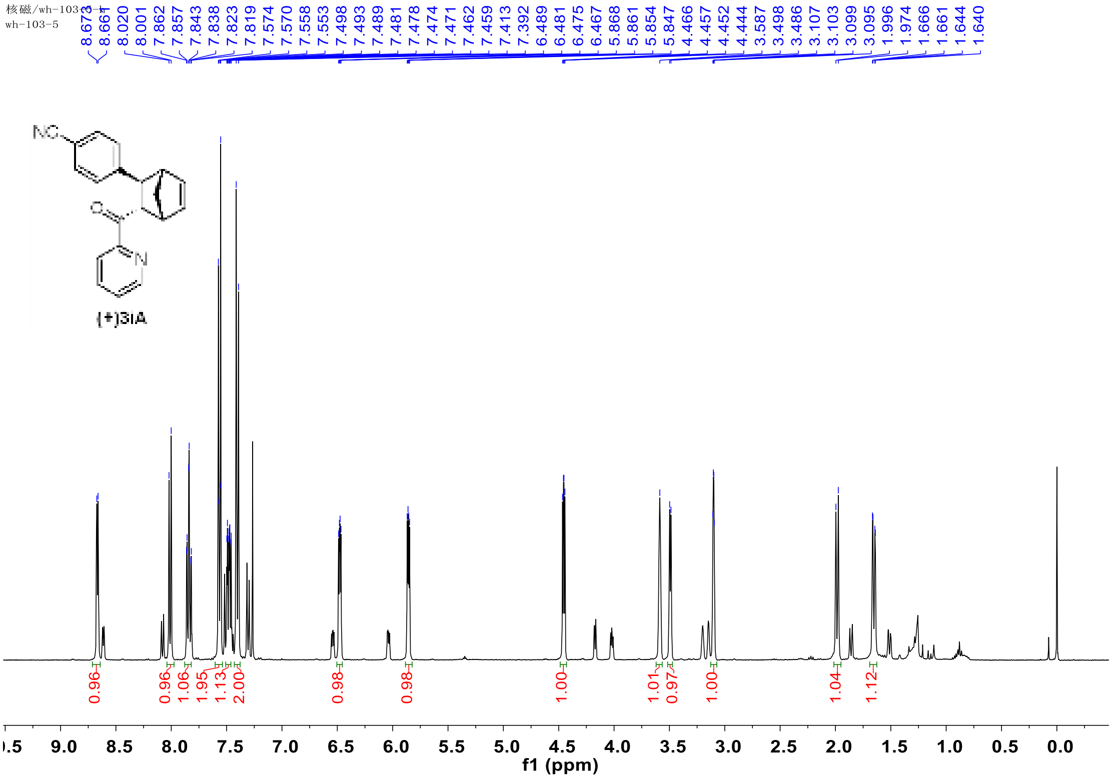

^{13}C NMR (101 MHz, $CDCl_3$)

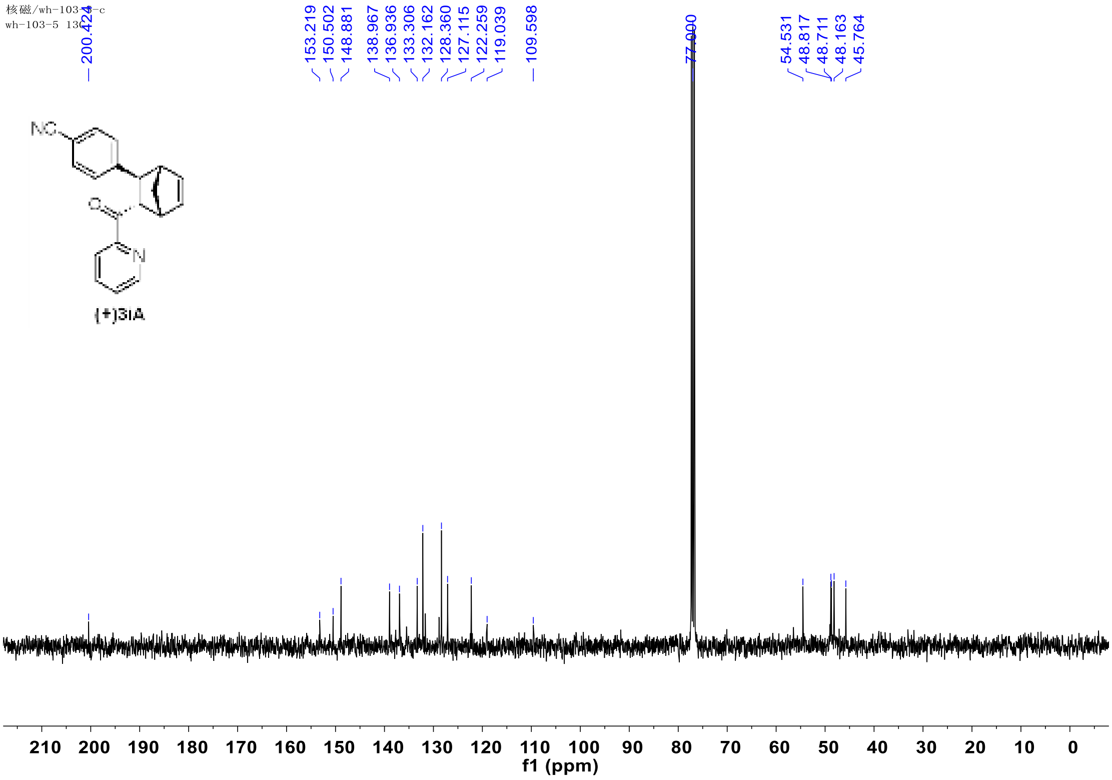

Pyridin-2-yl((1*R*,2*S*,3*S*,4*S*)-3-(p-tolyl)bicyclo[2.2.1]hept-5-en-2-yl)methanone (+3jA)

^{1}H NMR (400 MHz, $CDCl_3$)

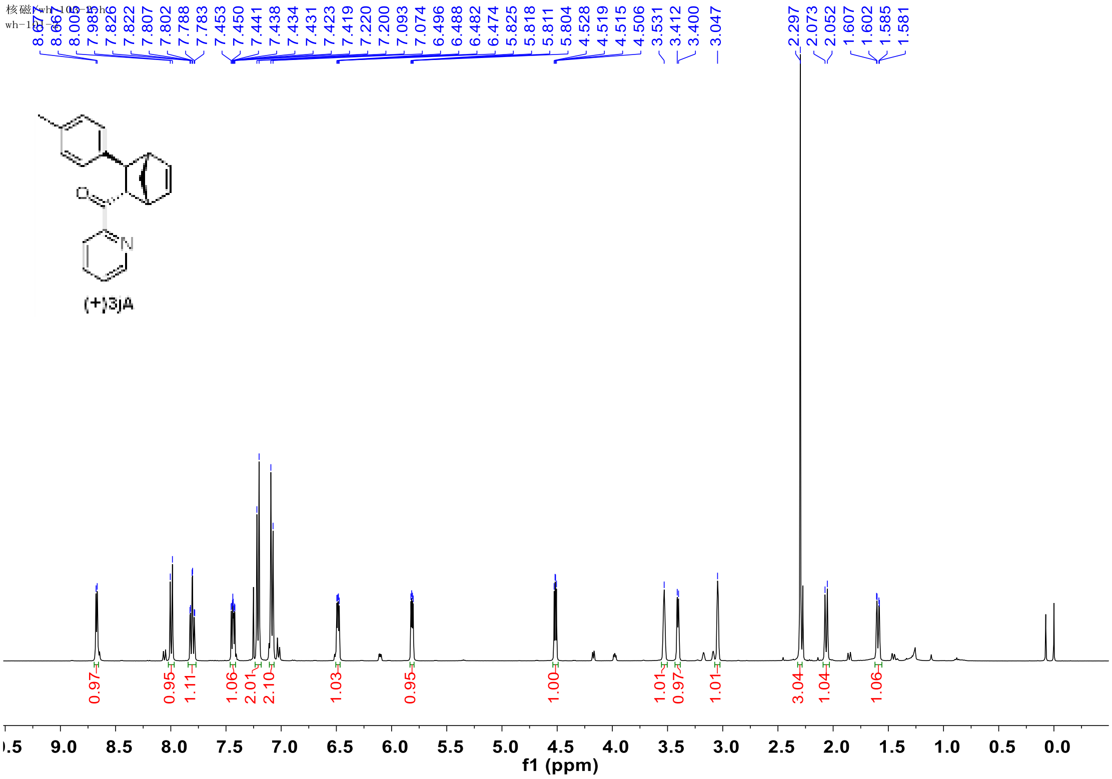

^{13}C NMR (101 MHz, $CDCl_3$)

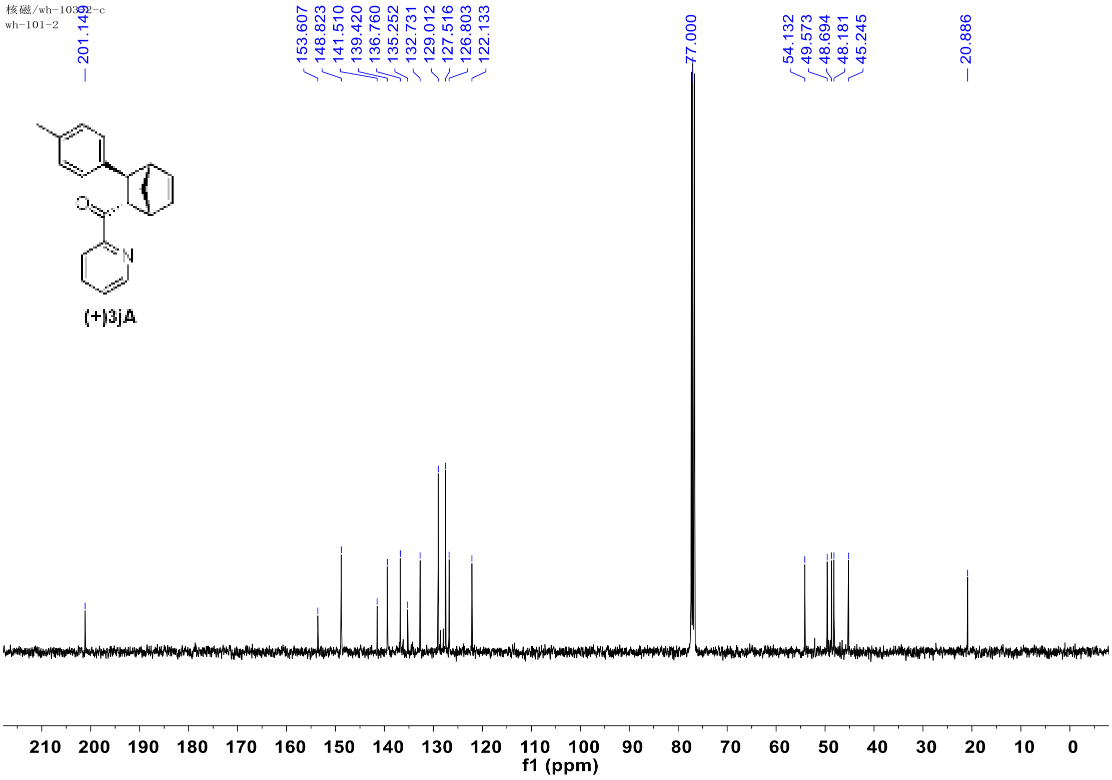

((1*R*,2*S*,3*S*,4*S*)-3-(3,5-dimethylphenyl)bicyclo[2.2.1]hept-5-en-2-yl)(pyridin-2-yl)methanone (+3kA)

^{1}H NMR (400 MHz, $CDCl_3$)

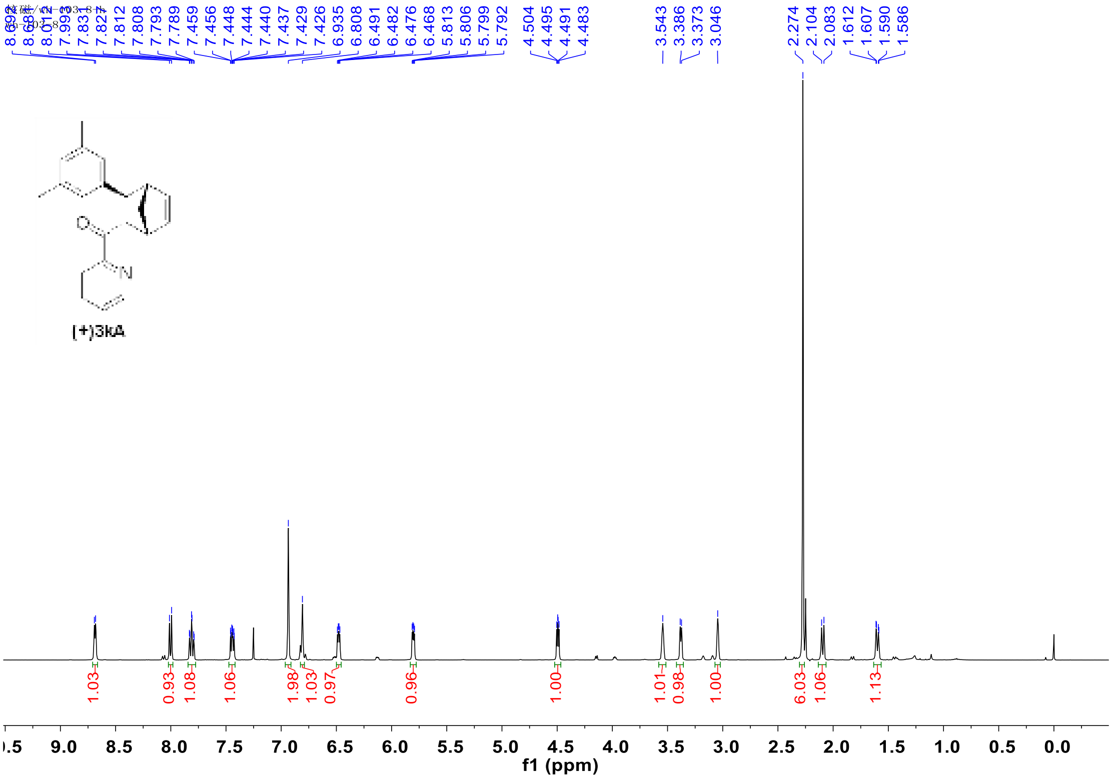

^{13}C NMR (101 MHz, $CDCl_3$)

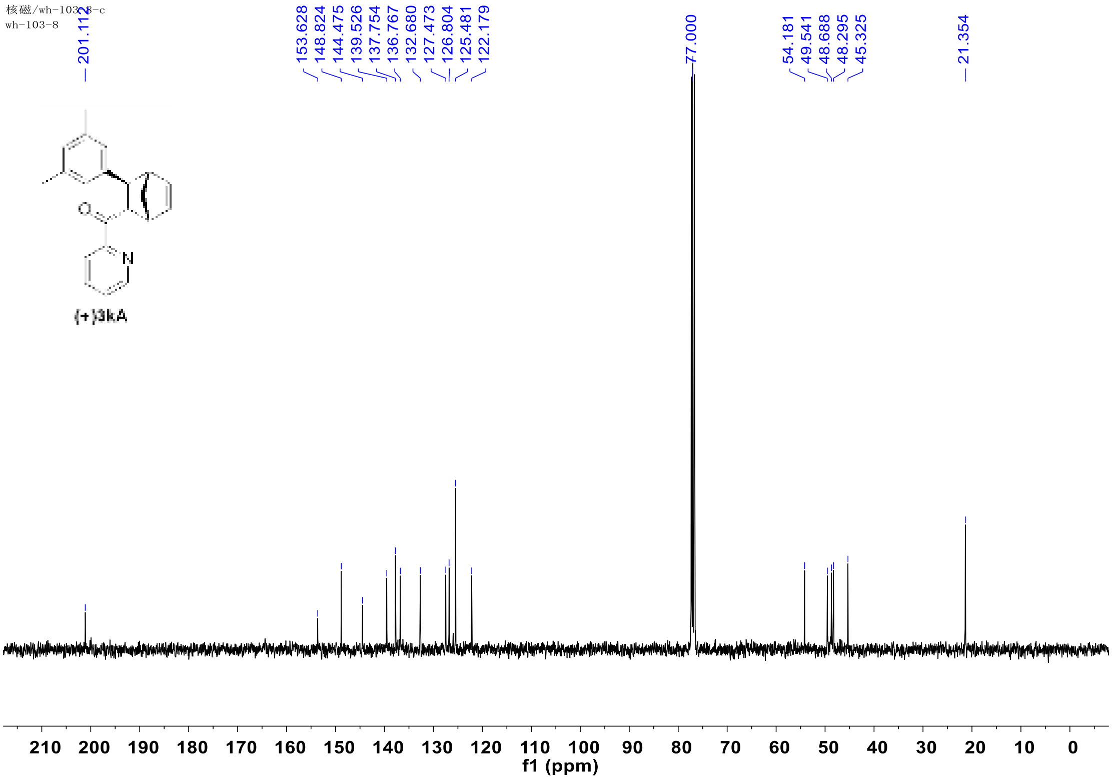

HRMS (ESI)

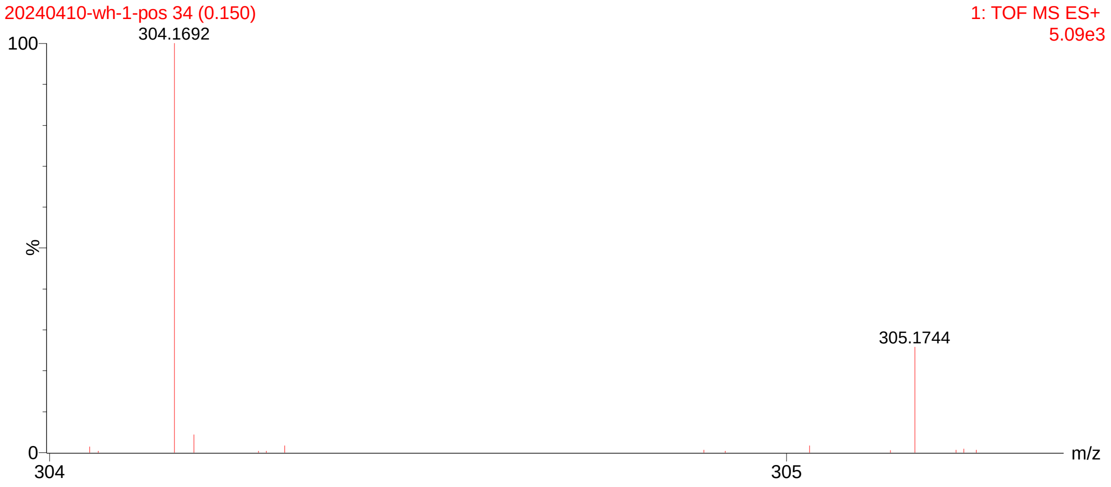

20240410-wh-1-pos 34 (0.150)
1: TOF MS ES+
5.09e3
304.1692
305.1744
100
%
0
304
305
m/z

((1*R*,2*S*,3*S*,4*S*)-3-(4-methoxyphenyl)bicyclo[2.2.1]hept-5-en-2-yl)(pyridin-2-yl)methanone (+3lA)

^{1}H NMR (400 MHz, CDCl$_3$)

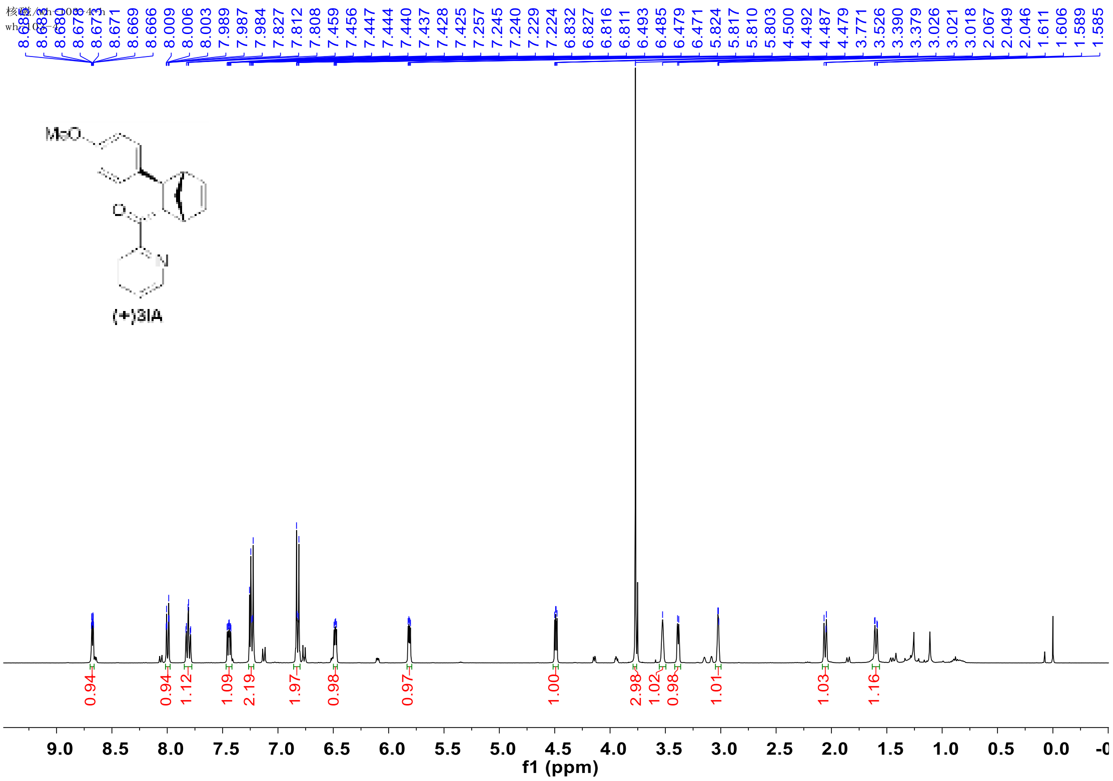

^{13}C NMR (101 MHz, CDCl$_3$)

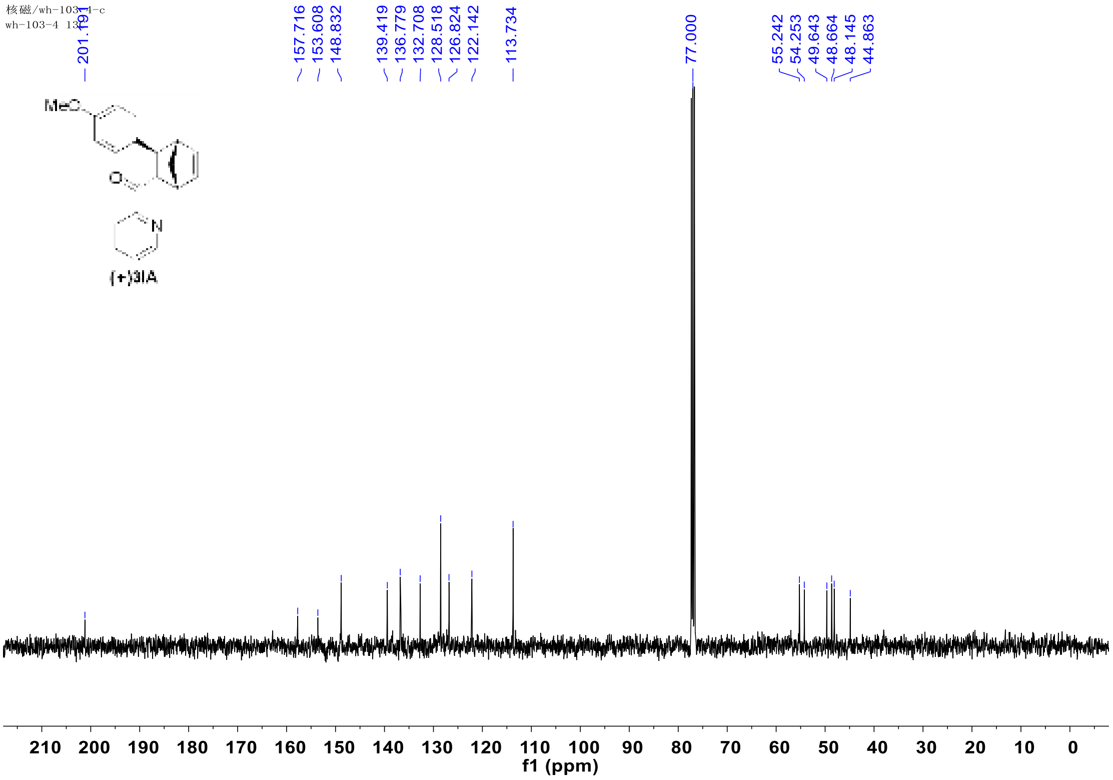

((1*R*,2*S*,3*S*,4*S*)-3-(naphthalen-2-yl)bicyclo[2.2.1]hept-5-en-2-yl)(pyridin-2-yl)methanone (+3mA)

^{1}H NMR (400 MHz, $CDCl_3$)

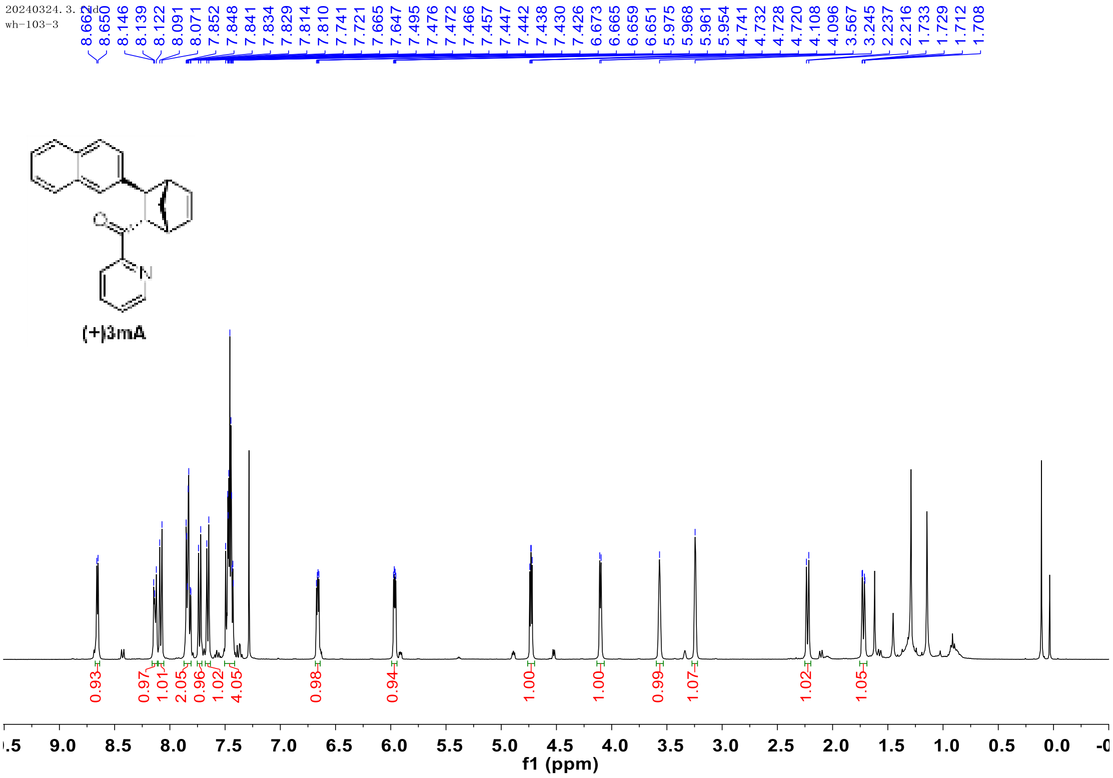

^{13}C NMR (101 MHz, $CDCl_3$)

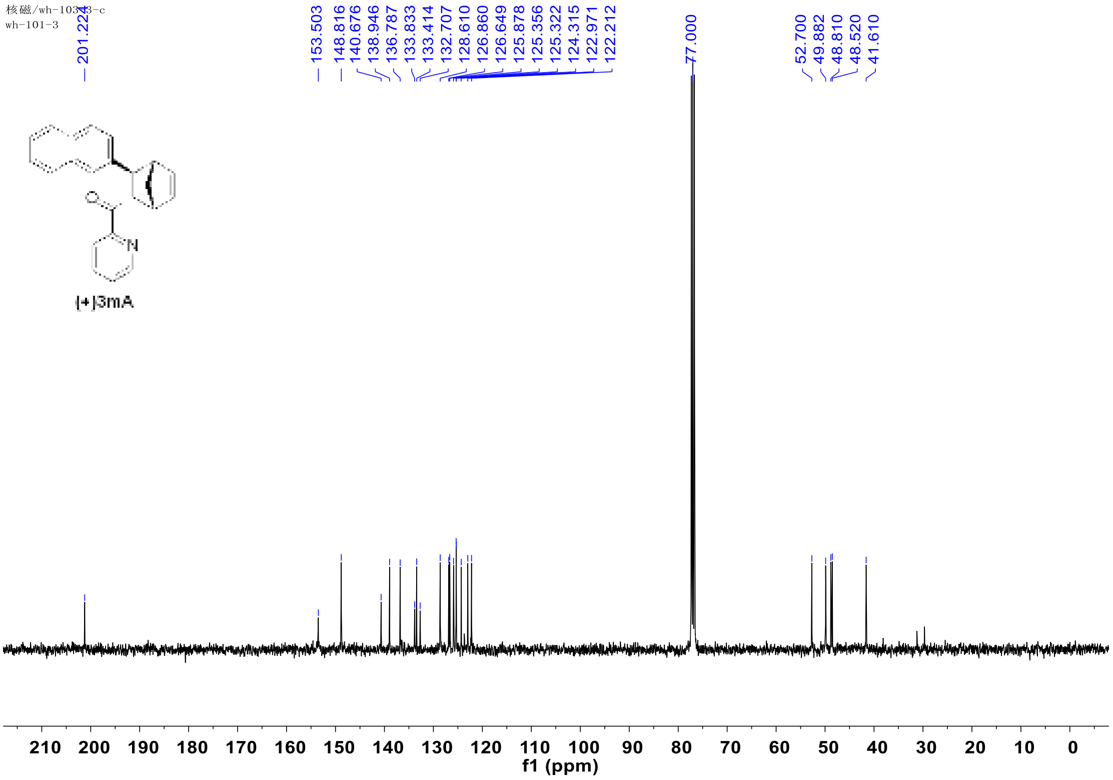

HRMS (ESI)

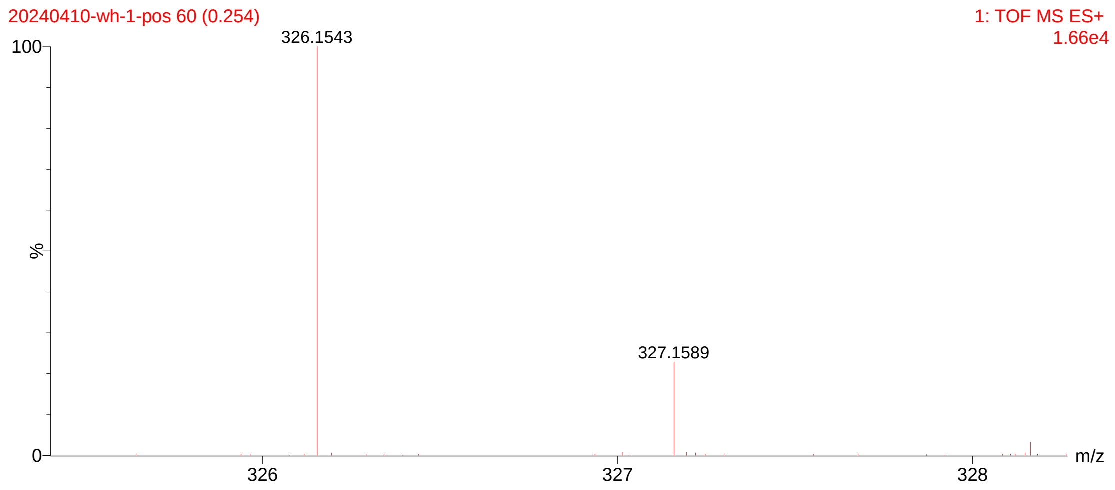

20240410-wh-1-pos 60 (0.254)
1: TOF MS ES+
1.66e4
326.1543
327.1589
100
%
0
326
327
328
m/z

Pyridin-2-yl((1*R*,2*S*,3*S*,4*S*)-3-(thiophen-2-yl)bicyclo[2.2.1]hept-5-en-2-yl)methanone (+3nA)

^{1}H NMR (400 MHz, $CDCl_3$)

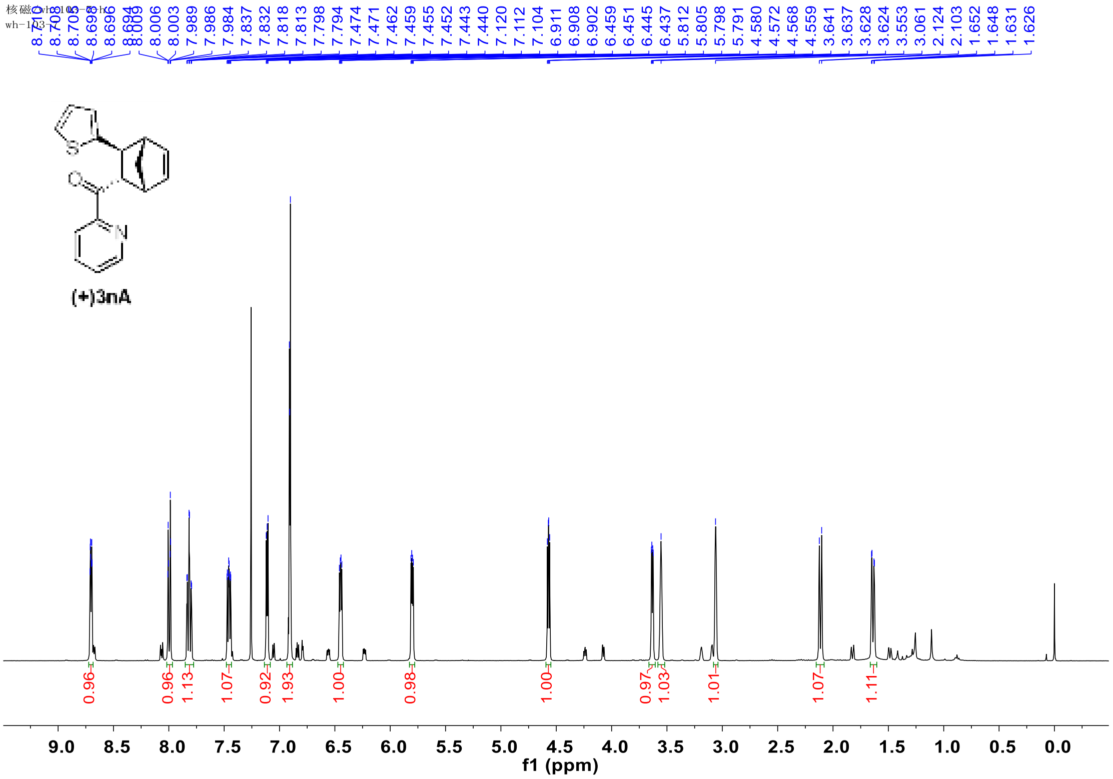

^{13}C NMR (101 MHz, $CDCl_3$)

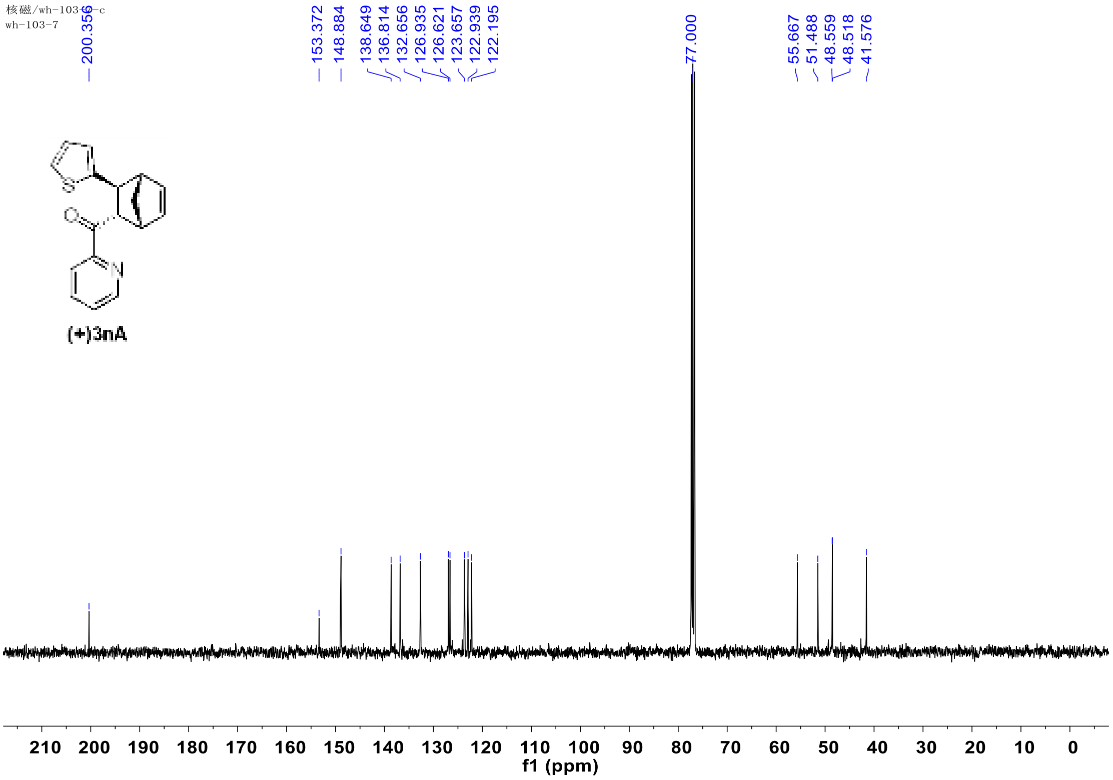

((1*R*,2*S*,3*S*,4*S*)-3-(benzo[b]thiophen-2-yl)bicyclo[2.2.1]hept-5-en-2-yl)(pyridin-2-yl)methanone (+3oA)

^{1}H NMR (400 MHz, CDCl$_3$)

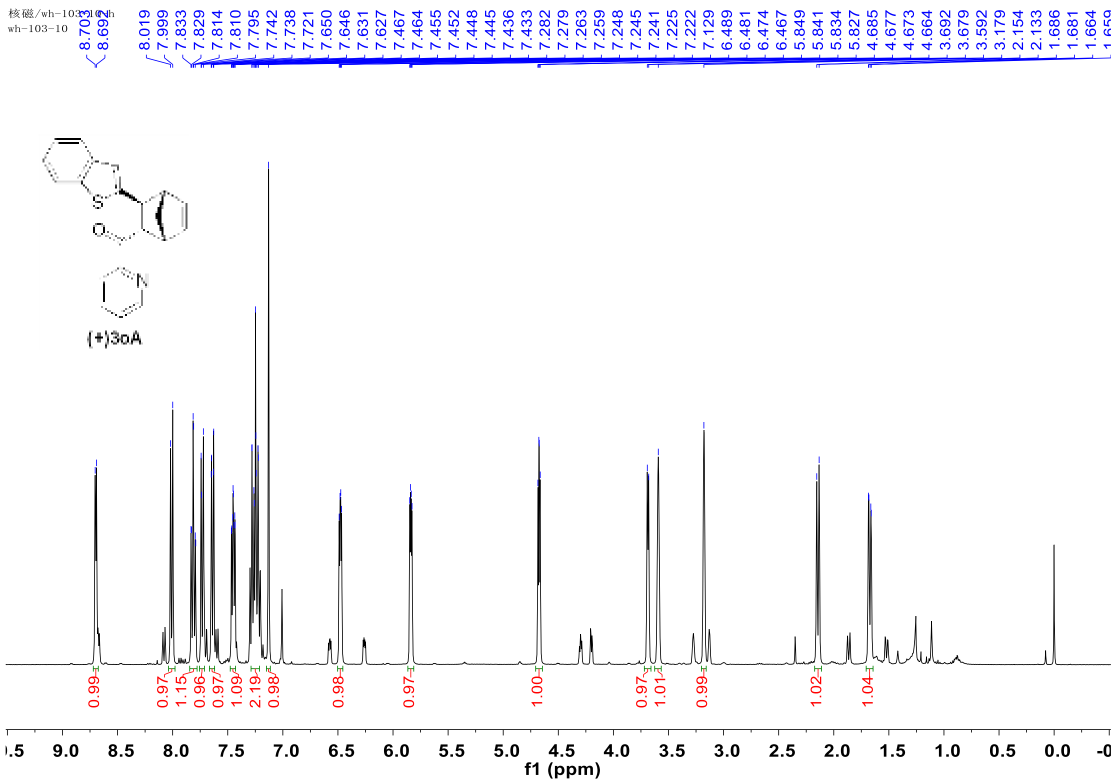

^{13}C NMR (101 MHz, CDCl$_3$)

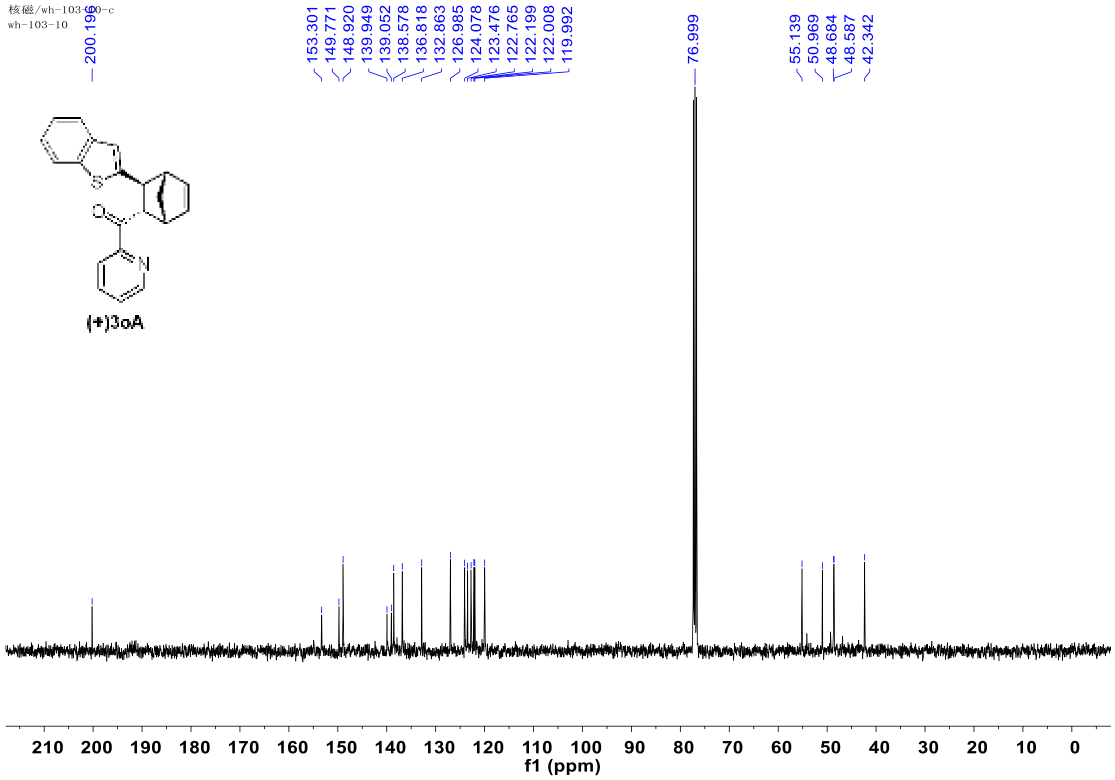

HRMS (ESI)

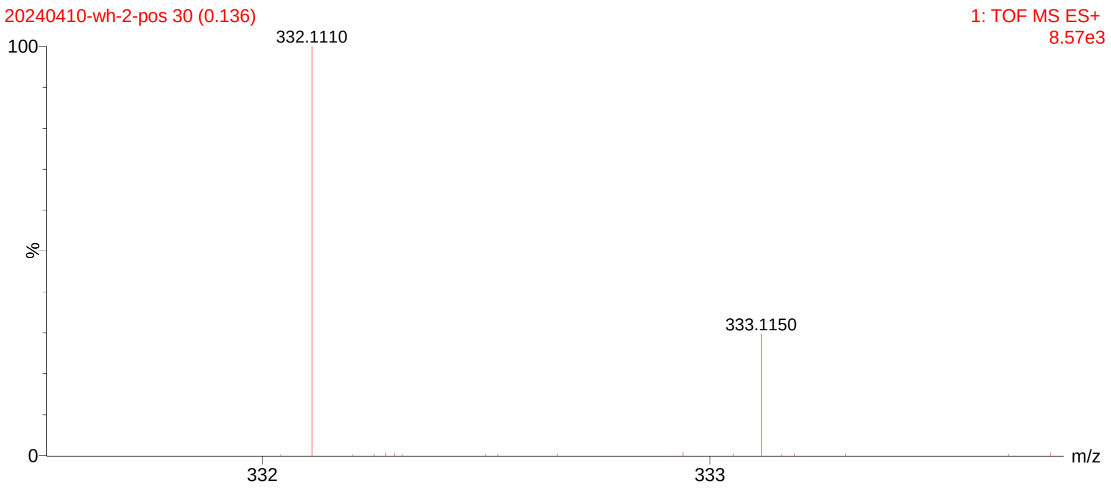
20240410-wh-2-pos 30 (0.136)
1: TOF MS ES+
8.57e3
332.1110
333.1150
100
%
0
332
333
m/z

((1*R*,2*S*,3*S*,4*S*)-3-cyclohexylbicyclo[2.2.1]hept-5-en-2-yl)(pyridin-2-yl)methanone (+3pA)

^{1}H NMR (400 MHz, $CDCl_3$)

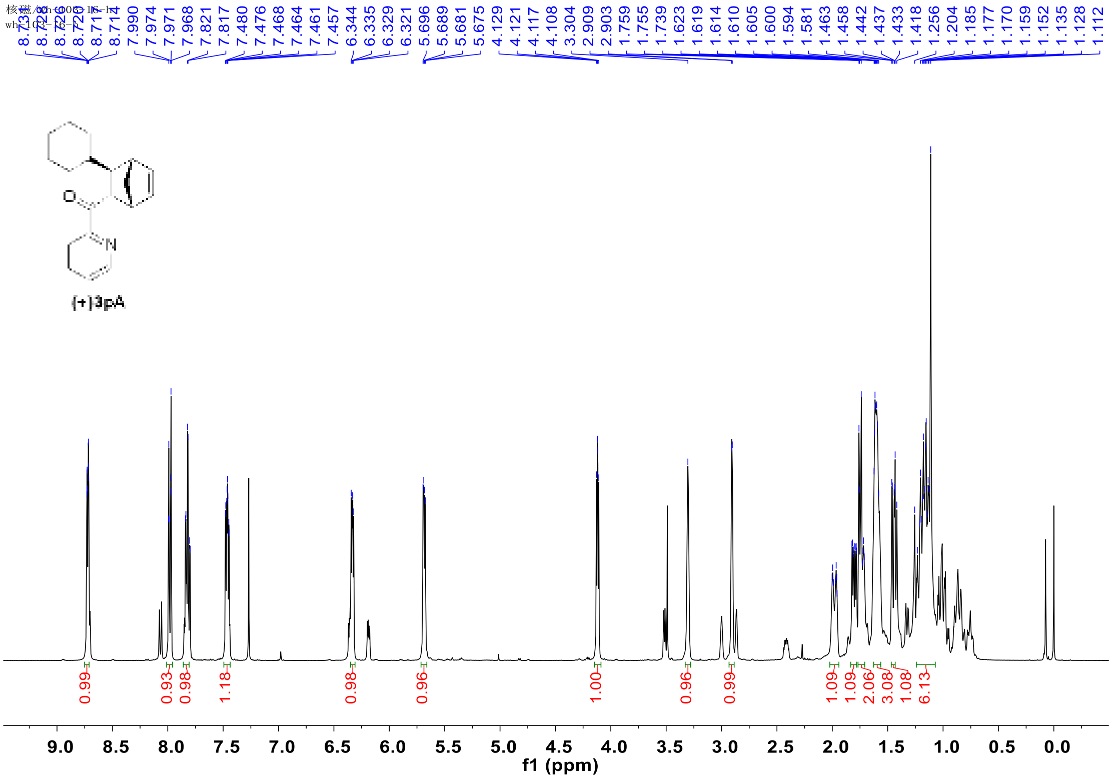

^{13}C NMR (101 MHz, $CDCl_3$)

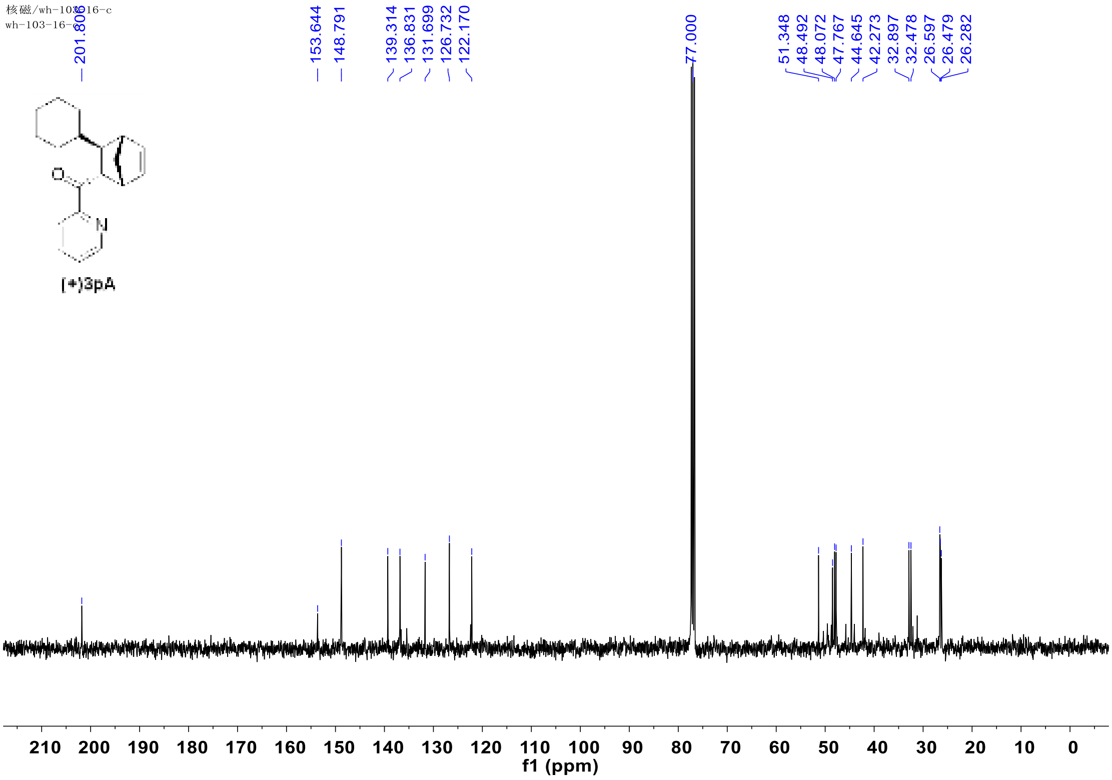

((1*R*,2*S*,3*S*,4*S*)-3-cyclopentylbicyclo[2.2.1]hept-5-en-2-yl)(pyridin-2-yl)methanone (+3qA)

^{1}H NMR (400 MHz, $CDCl_3$)

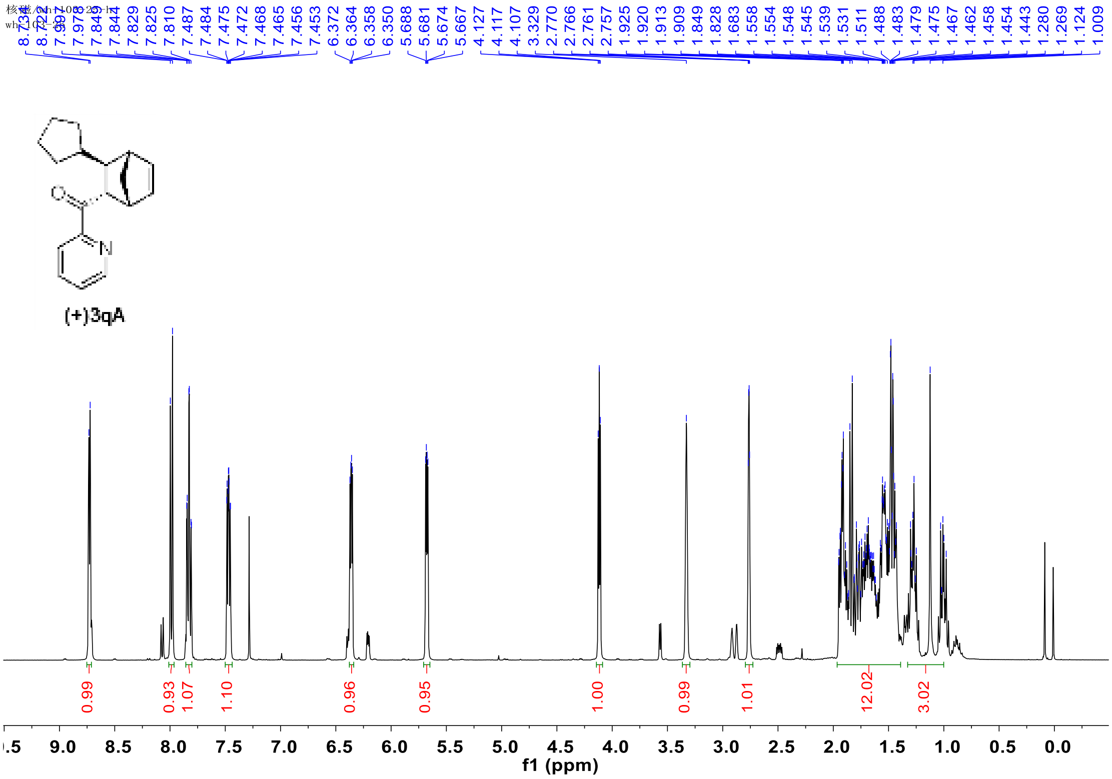

^{13}C NMR (101 MHz, $CDCl_3$)

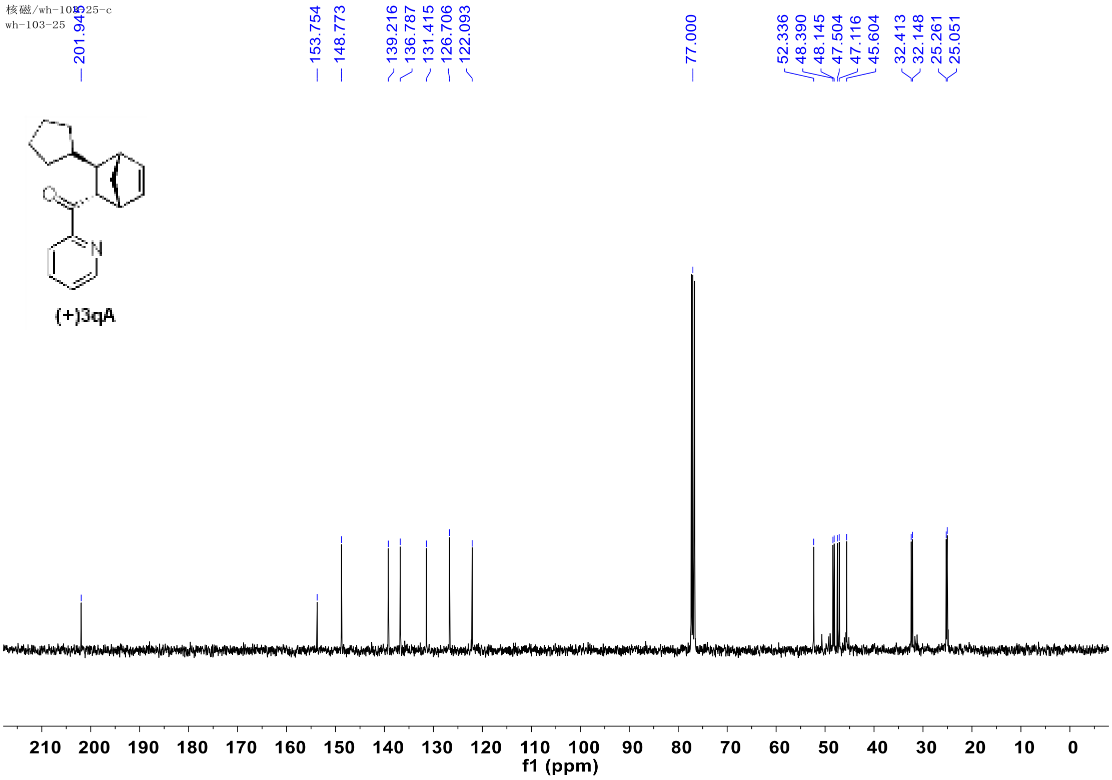

HRMS (ESI)

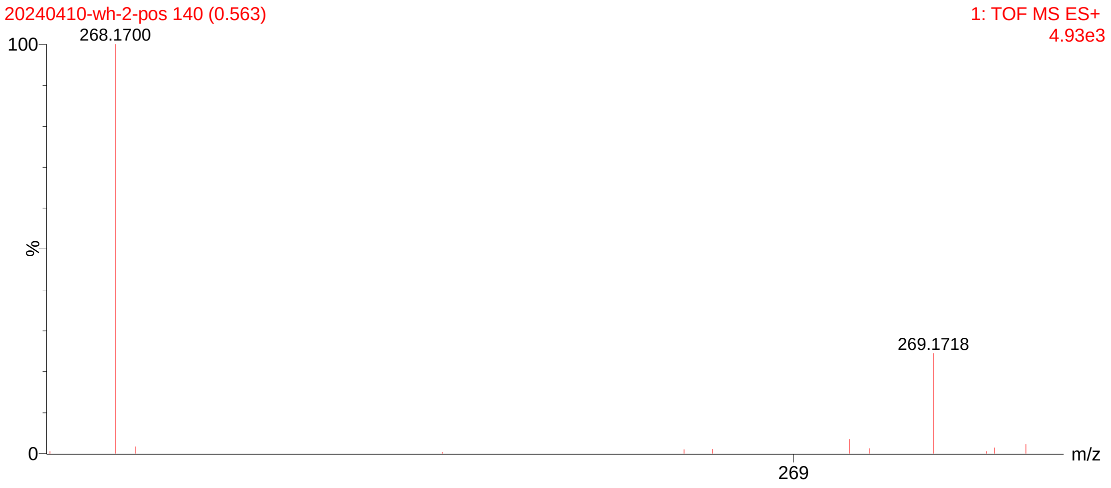

20240410-wh-2-pos 140 (0.563)
1: TOF MS ES+
4.93e3
100
268.1700
%
269.1718
0
269
m/z

((1*R*,2*S*,3*S*,4*S*)-3-cyclopropylbicyclo[2.2.1]hept-5-en-2-yl)(pyridin-2-yl)methanone (+3rA)

^{1}H NMR (400 MHz, $CDCl_3$)

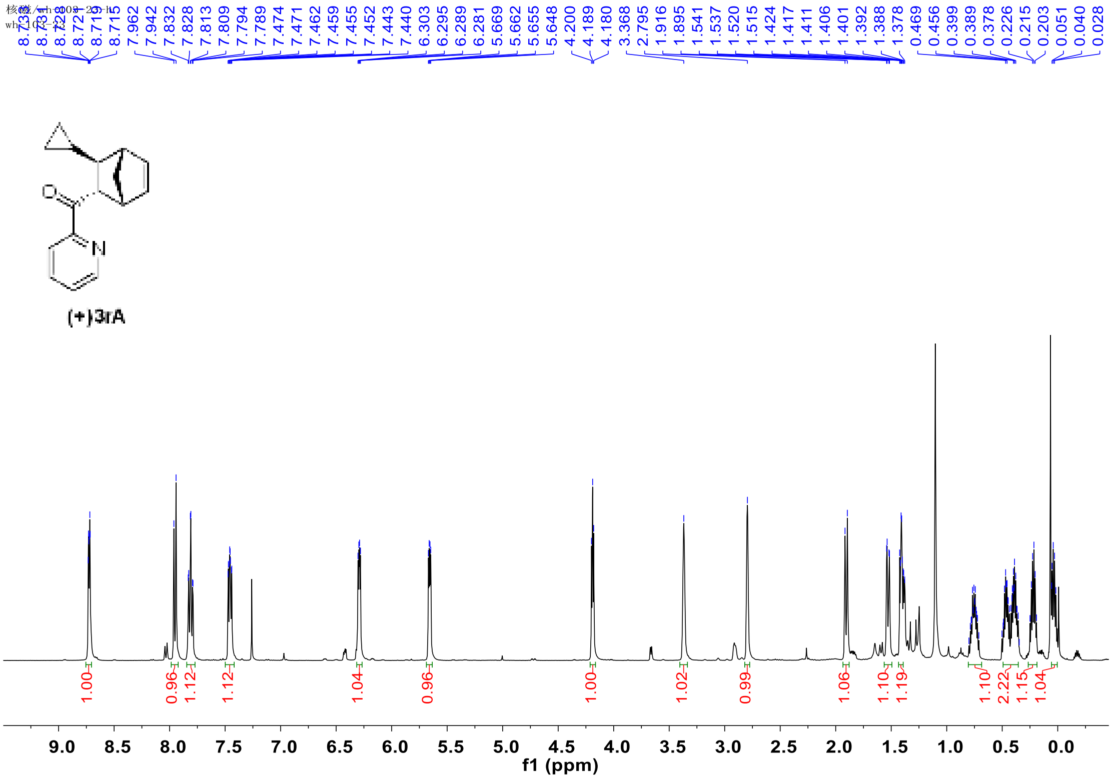

^{13}C NMR (101 MHz, $CDCl_3$)

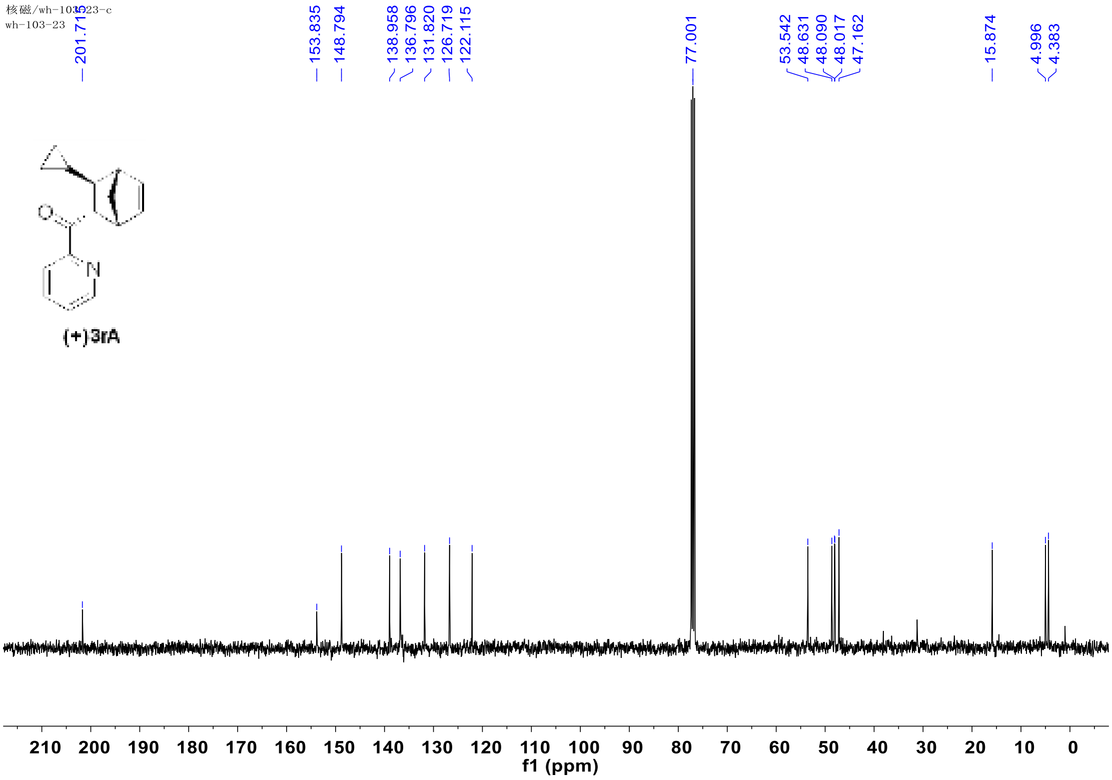

HRMS (ESI)

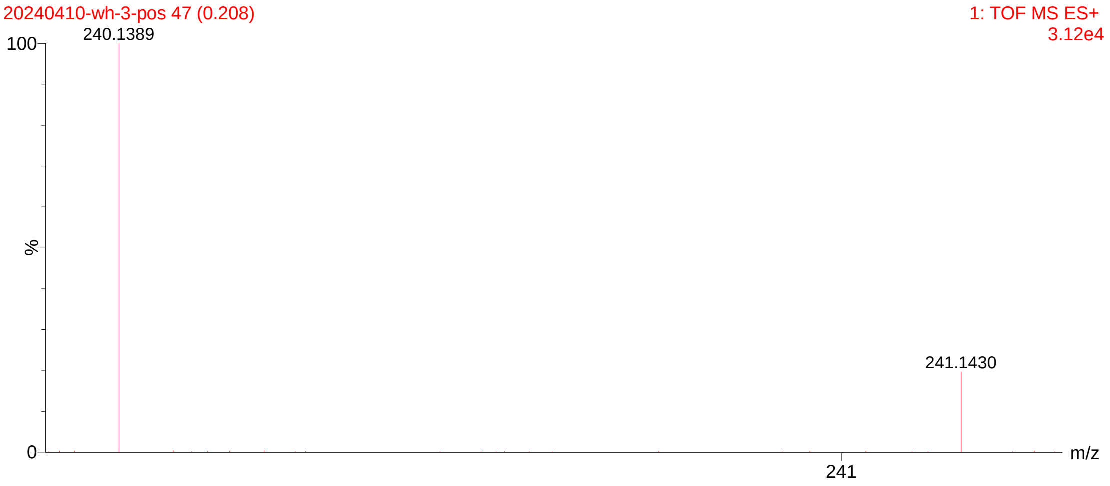
20240410-wh-3-pos 47 (0.208)
1: TOF MS ES+
3.12e4
100
240.1389
%
241.1430
0
241
m/z

((1*S*,2*S*)-4,5-dimethyl-1,2,3,6-tetrahydro-[1,1'-biphenyl]-2-yl)(pyridin-2-yl)methanone (+3aB)

^{1}H NMR (400 MHz, $CDCl_3$)

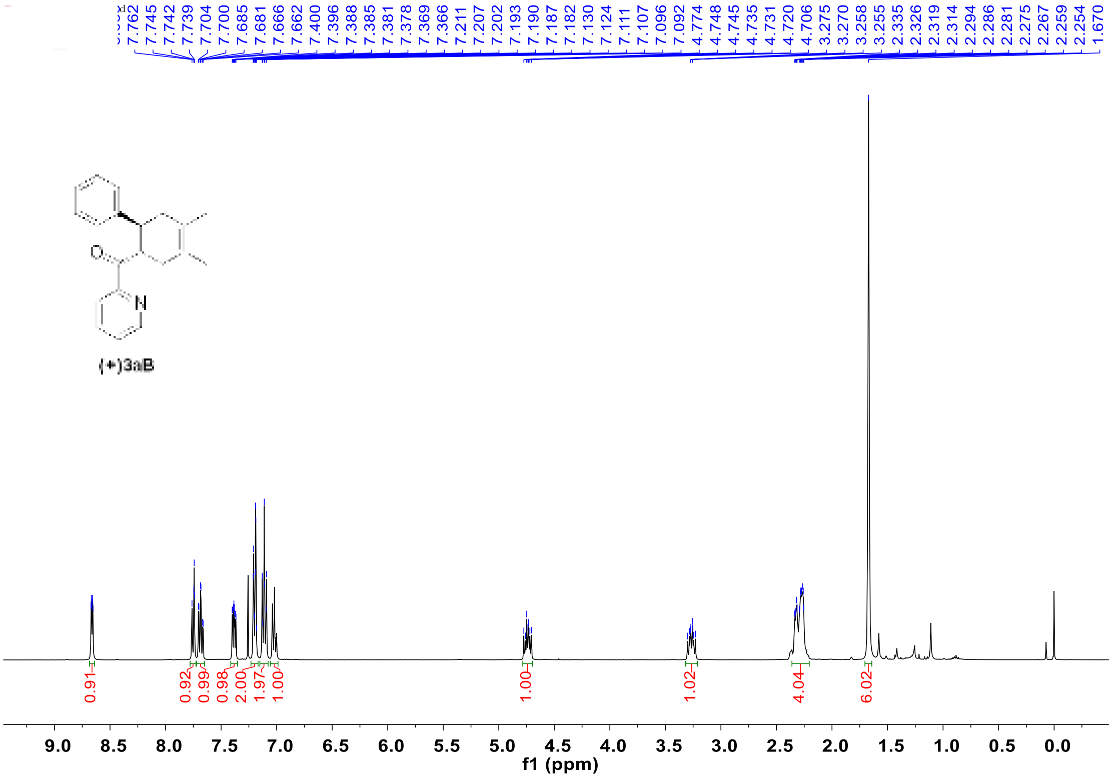

^{13}C NMR (101 MHz, $CDCl_3$)

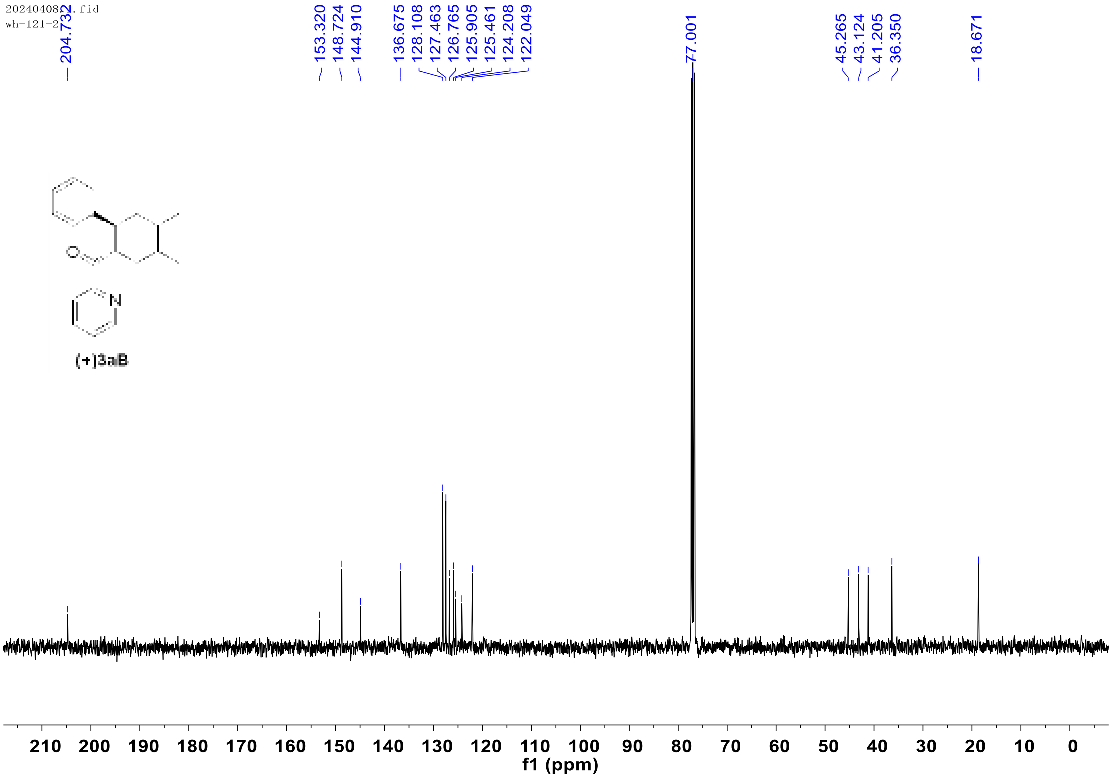

HRMS (ESI)

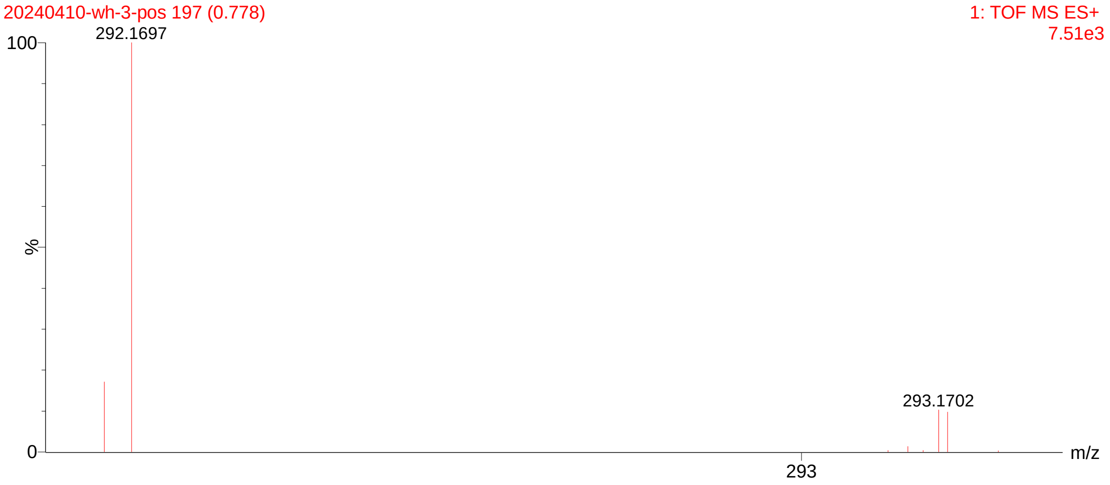
20240410-wh-3-pos 197 (0.778)
1: TOF MS ES+
7.51e3
292.1697
293.1702
100
%
0
293
m/z

((1*R*,2*R*,4*S*,5*S*,6*R*,7*S*)-7-phenyl-3-oxatricyclo[3.2.1.02,4]octan-6-yl)(pyridin-2-yl)methanone (5)

^{1}H NMR (400 MHz, $CDCl_3$)

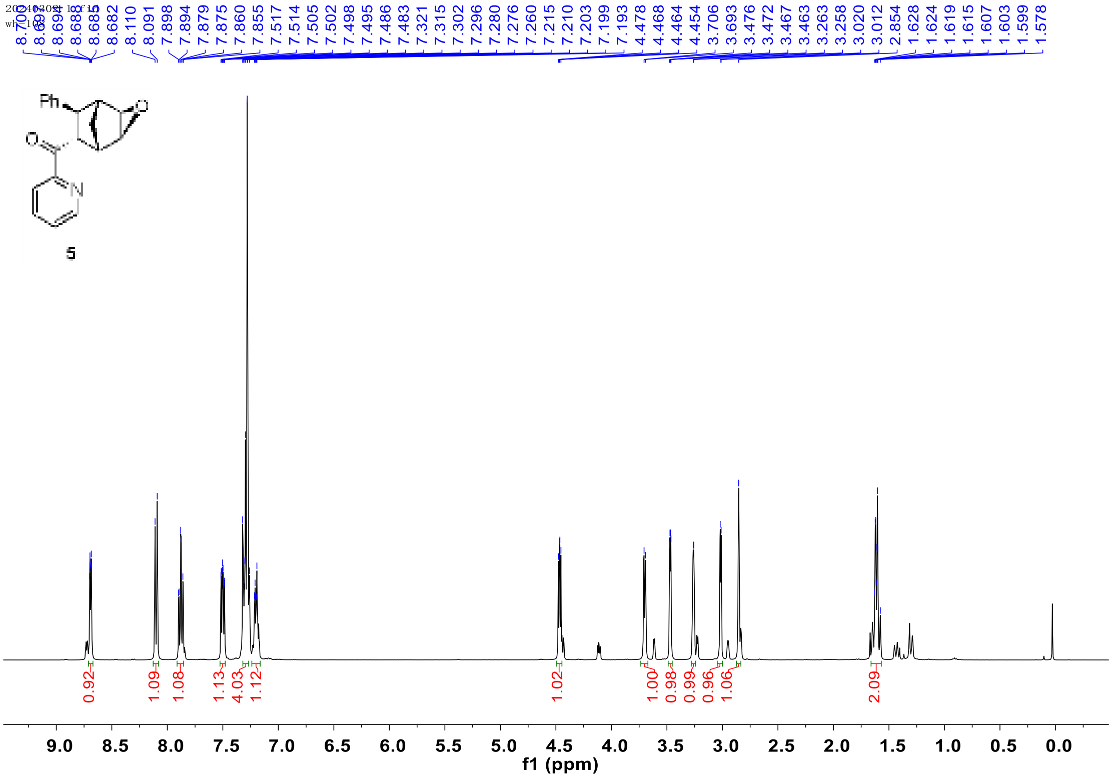

^{13}C NMR (101 MHz, $CDCl_3$)

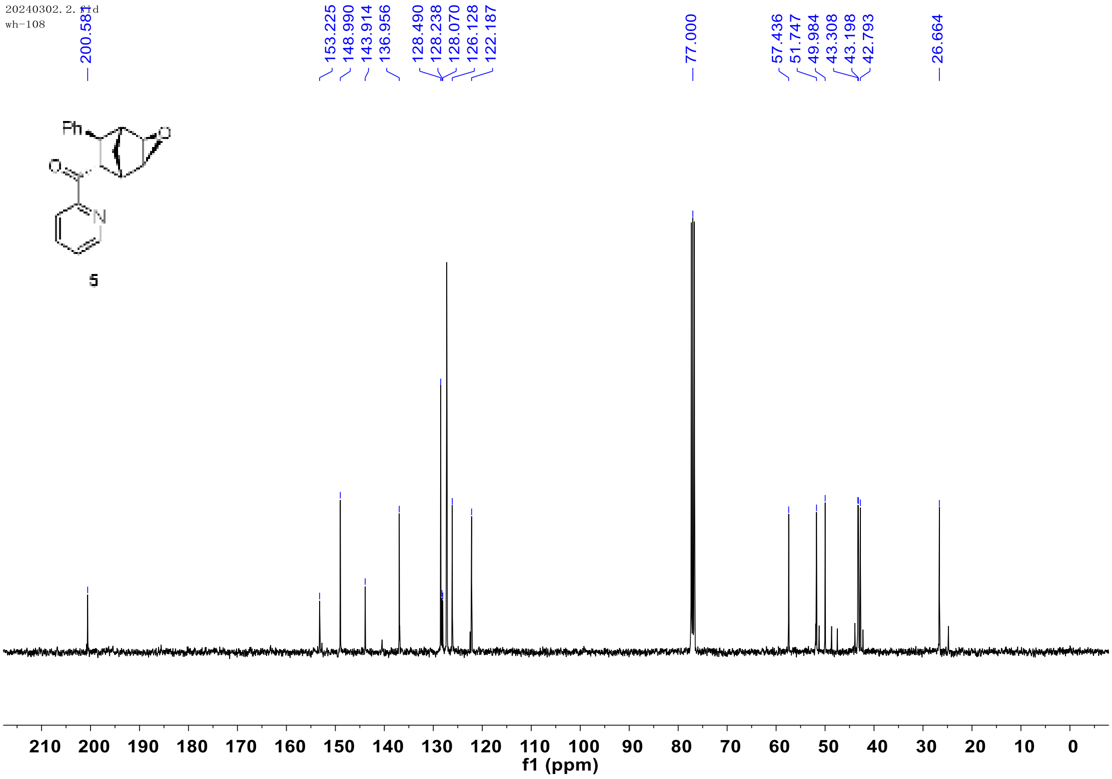

HRMS (ESI)

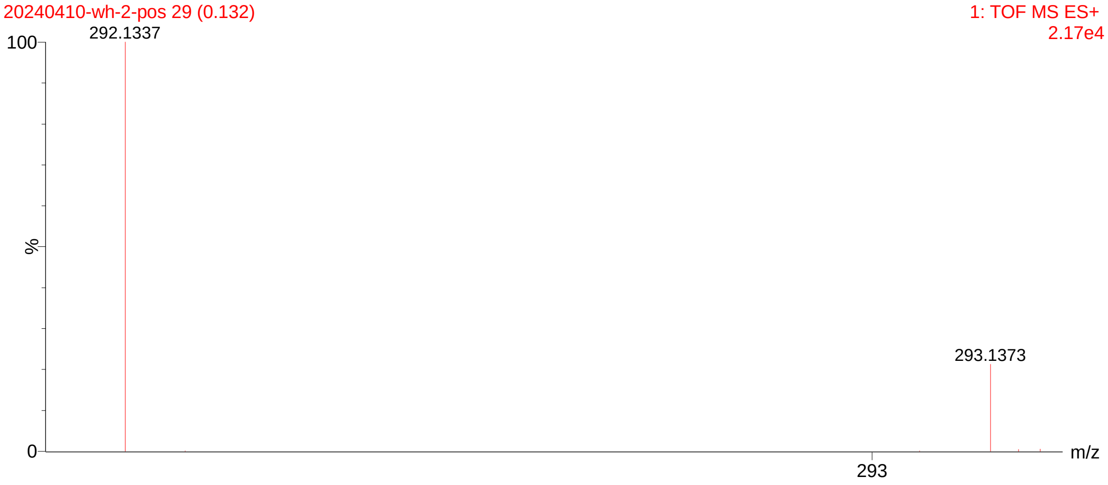

20240410-wh-2-pos 29 (0.132)
1: TOF MS ES+
2.17e4
100
292.1337
%
293.1373
0
293
m/z

(4-chlorophenyl)((1*R*,2*S*,3*S*,4*S*)-3-phenylbicyclo[2.2.1]hept-5-en-2-yl)(pyridin-2-yl)methanol (6)

^{1}H NMR (400 MHz, $CDCl_3$) three diastereoisomers

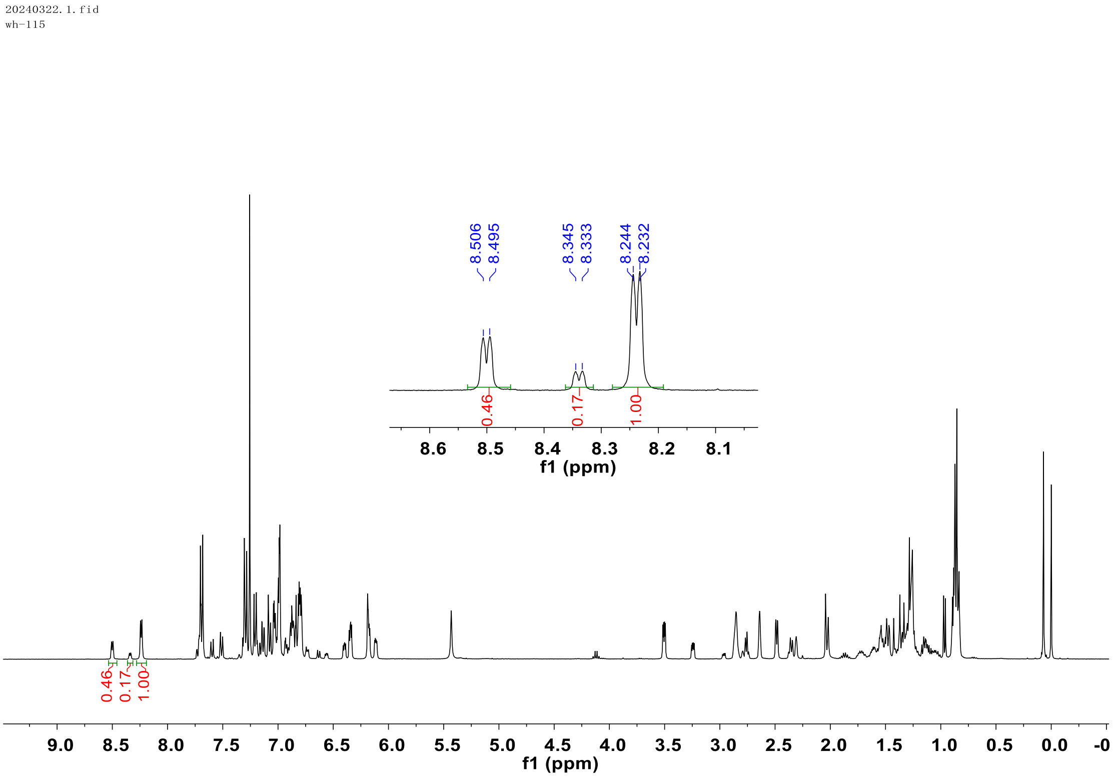

HRMS (ESI)

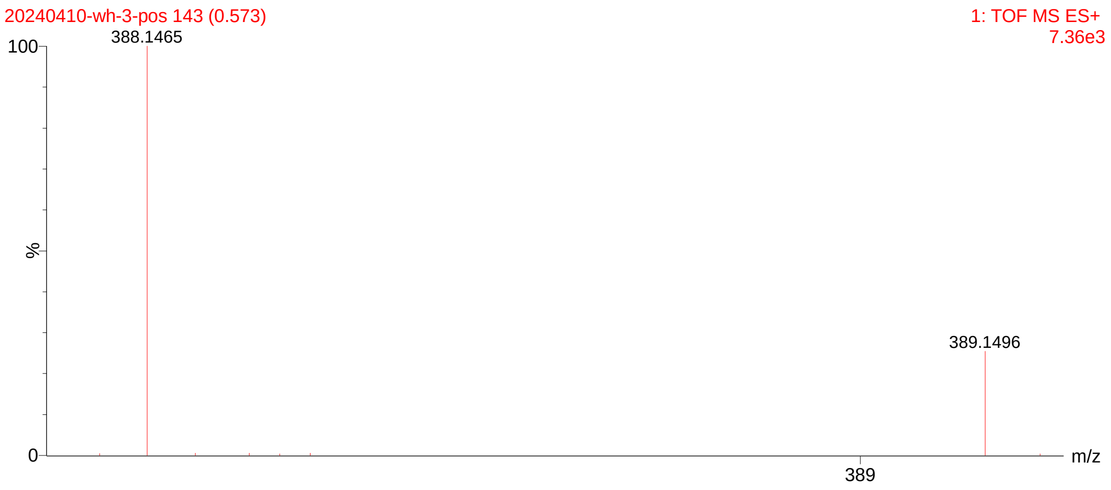

(S)-((1*R*,2*S*,3*S*,4*S*)-3-phenylbicyclo[2.2.1]hept-5-en-2-yl)(pyridin-2-yl)methanol (7)

^{1}H NMR (400 MHz, CDCl$_3$)

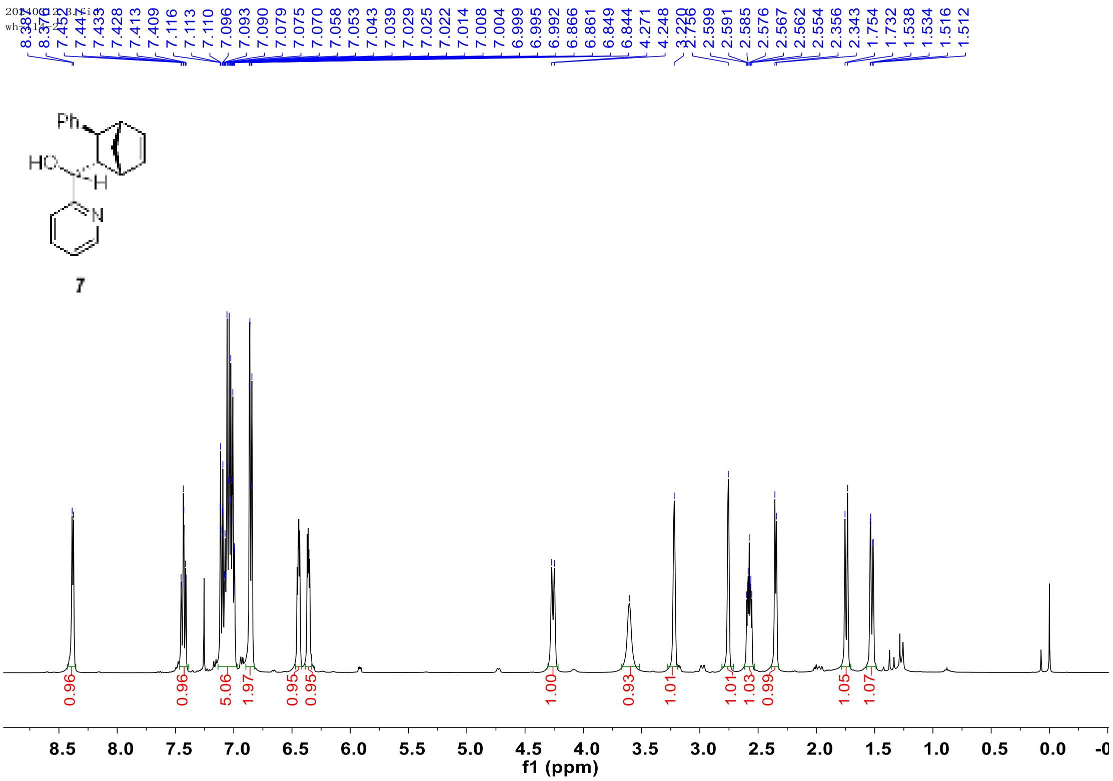

^{13}C NMR (101 MHz, CDCl$_3$)

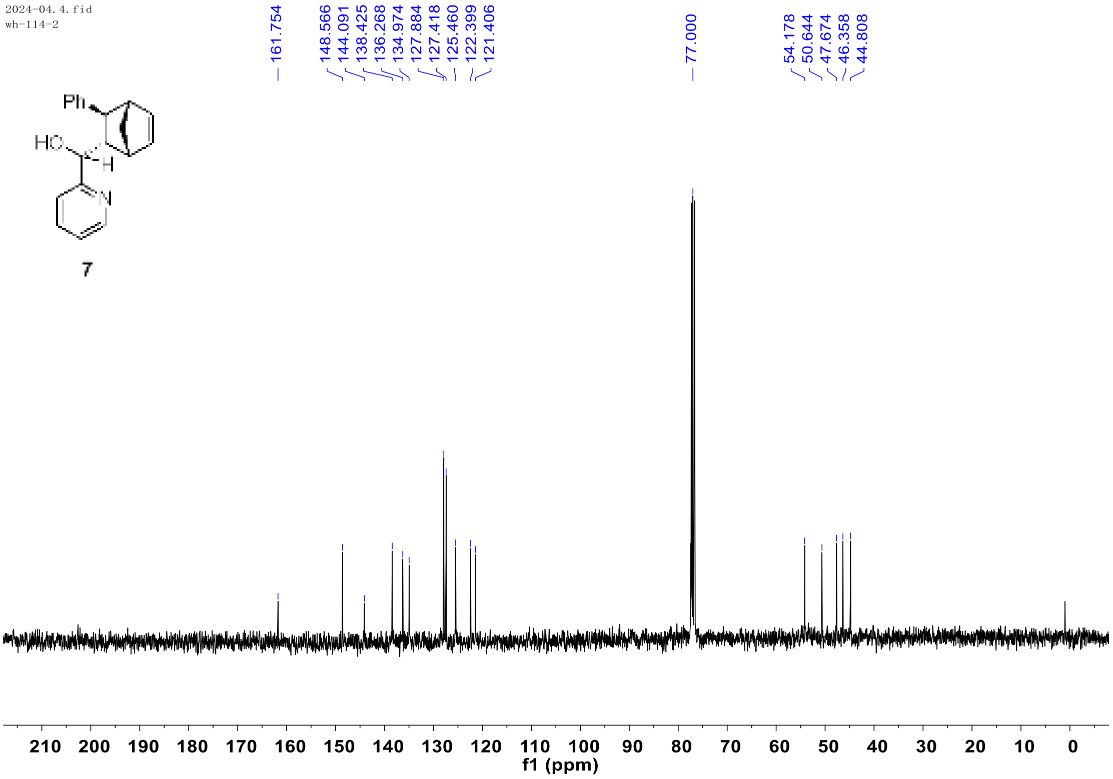

HRMS (ESI)

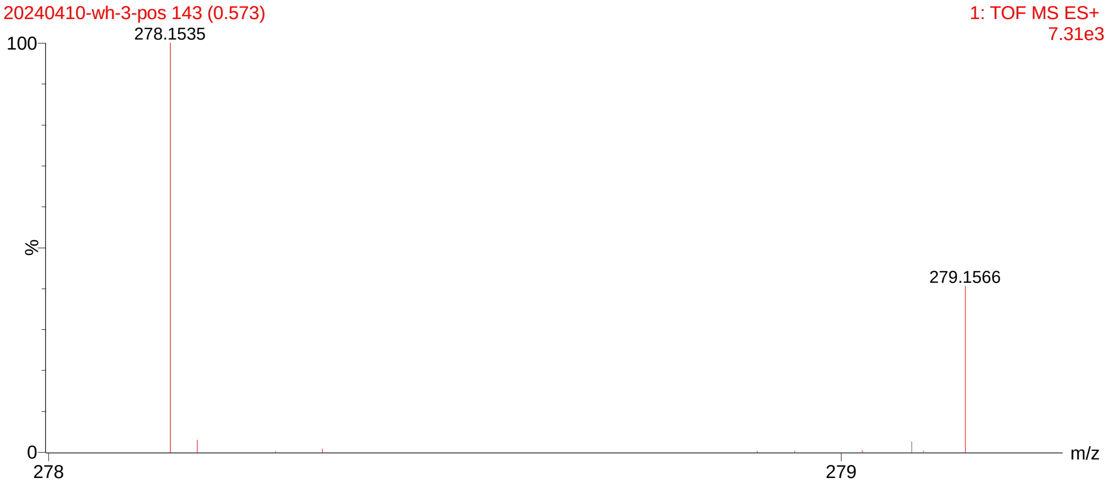

20240410-wh-3-pos 143 (0.573)
1: TOF MS ES+
7.31e3
278.1535
279.1566
100
%
0
278
279
m/z

3. HPLC Spectra

3.1 HPLC copies of compound

HPLC copies of compound (±)-3aA and (+)-3aA

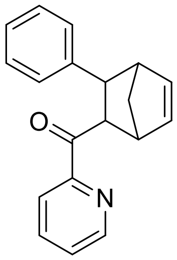

(±)3aA

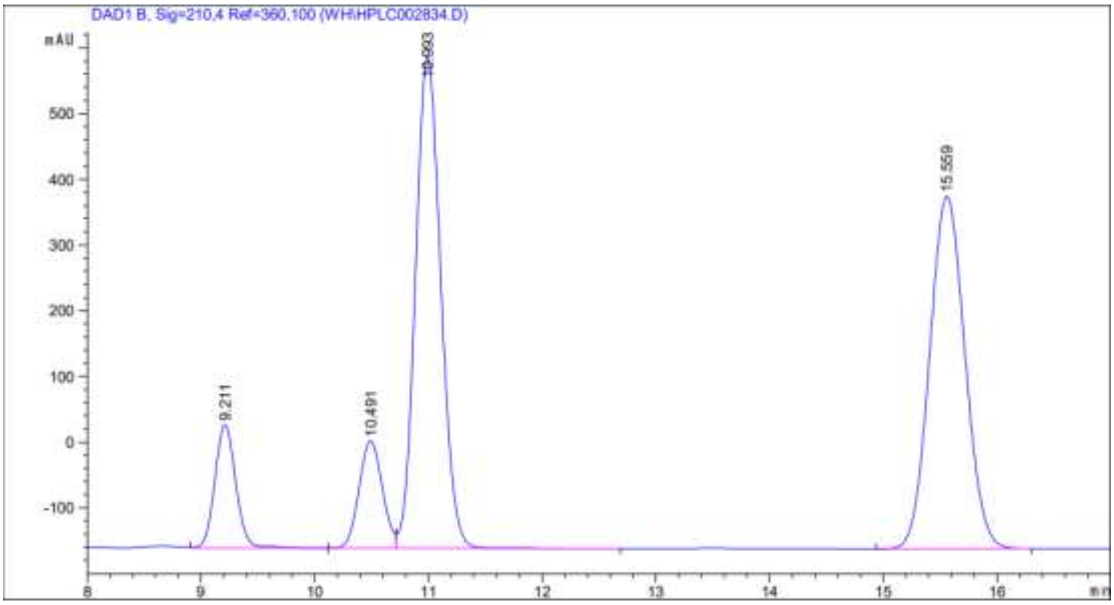

Peak #	Ret. Time [min]	Type	Width [min]	Area [mAU*s]	Height [mAU]	Area %
1	9.211	VB	0.1997	2403.19849	187.32208	8.6251
2	10.491	BV	0.2219	2326.81860	163.60168	8.3510
3	10.993	VB	0.2425	1.15814e4	748.54620	41.5656
4	15.559	BB	0.3372	1.15515e4	536.57239	41.4584

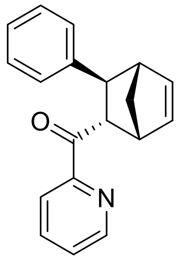

(+)3aA

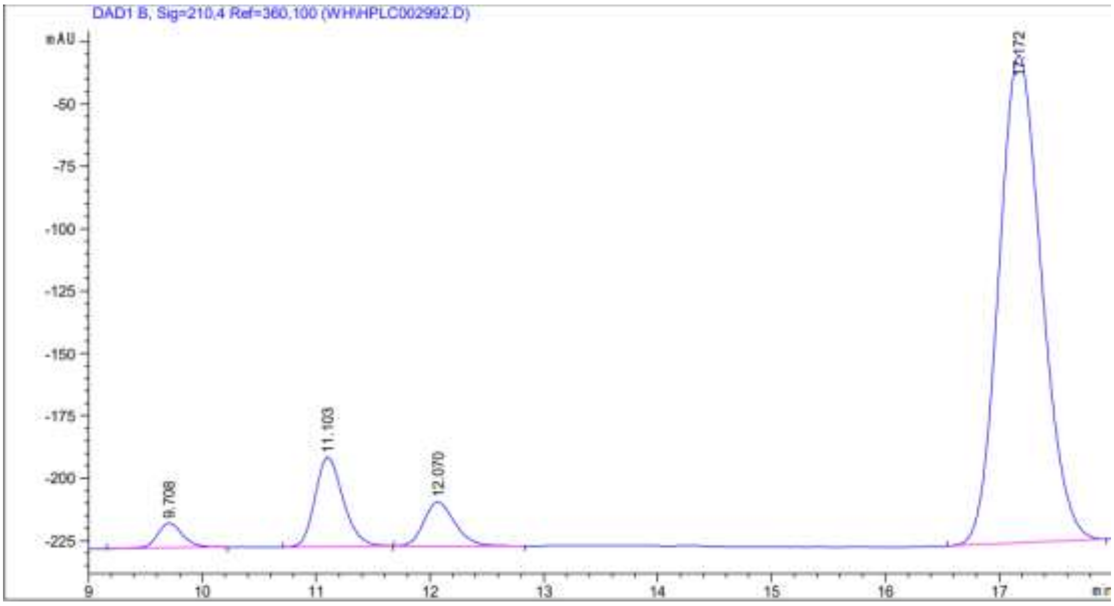

Peak #	Ret. Time [min]	Type	Width [min]	Area [mAU*s]	Height [mAU]	Area %
1	9.708	BB	0.2344	151.78452	9.92730	2.4584
2	11.103	BB	0.2641	612.79163	35.72356	9.9251
3	12.070	BB	0.2888	328.13419	17.49356	5.3146
4	17.172	BB	0.4035	5081.45313	195.32072	82.3019

HPLC copies of compound (±)-3bA and (+)-3bA

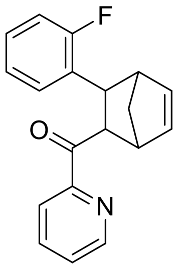

(±)3bA

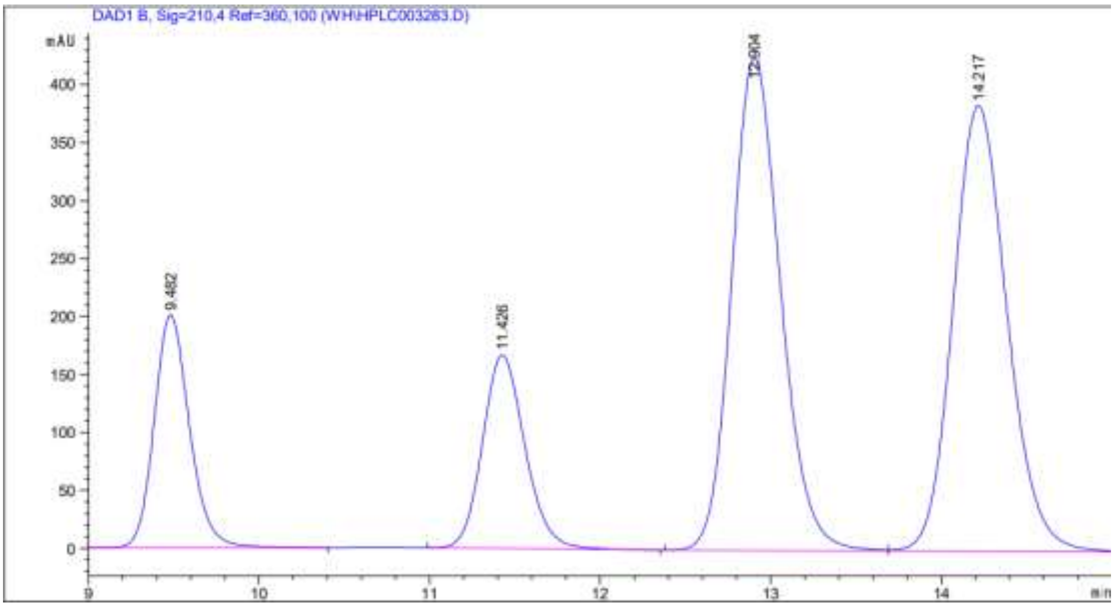

Peak	Ret. Time	Type	Width	Area	Height	Area
1	9.482	BB	0.2217	2888.54883	200.98512	12.7156
2	11.426	BB	0.2683	2893.95605	166.85451	12.7394
3	12.904	BV	0.3080	8452.45020	425.12296	37.2083
4	14.217	VB	0.3412	8481.61426	384.79788	37.3367

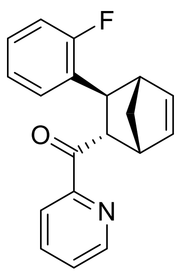

(+)3bA

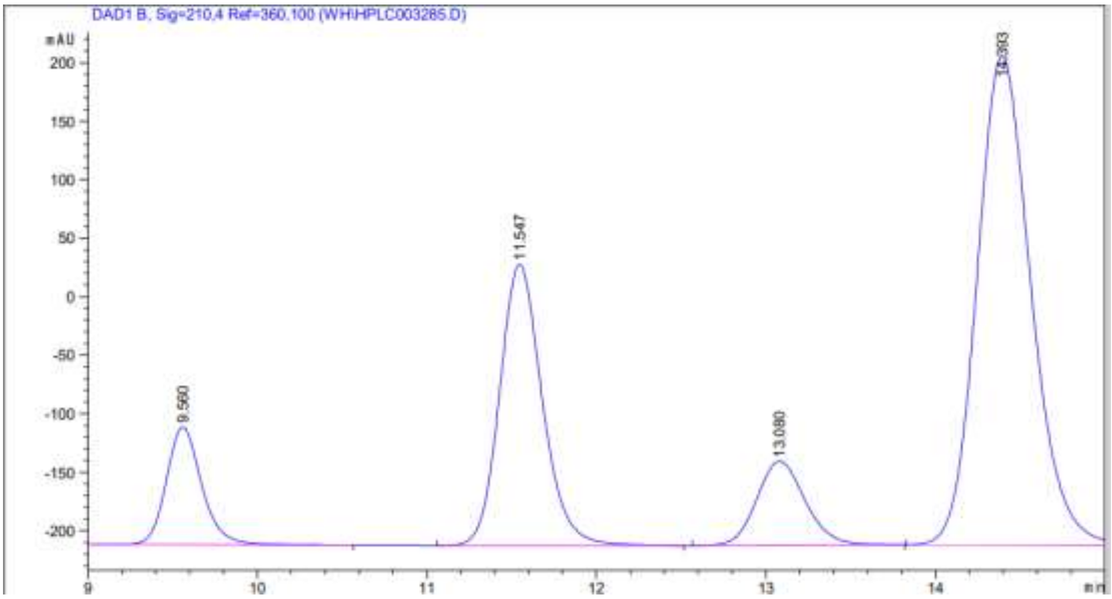

Peak	Ret. Time	Type	Width	Area	Height	Area
1	9.560	BB	0.2227	1469.83179	100.43183	8.9988
2	11.547	BB	0.2702	4202.56250	240.07852	25.7294
3	13.080	BB	0.3046	1417.42273	71.71904	8.6779
4	14.393	BB	0.3422	9243.90625	417.73291	56.5940

HPLC copies of compound (±)-3cA and (+)-3cA

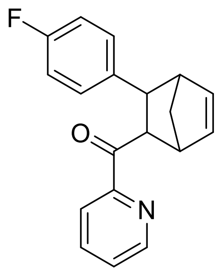

(±)3cA

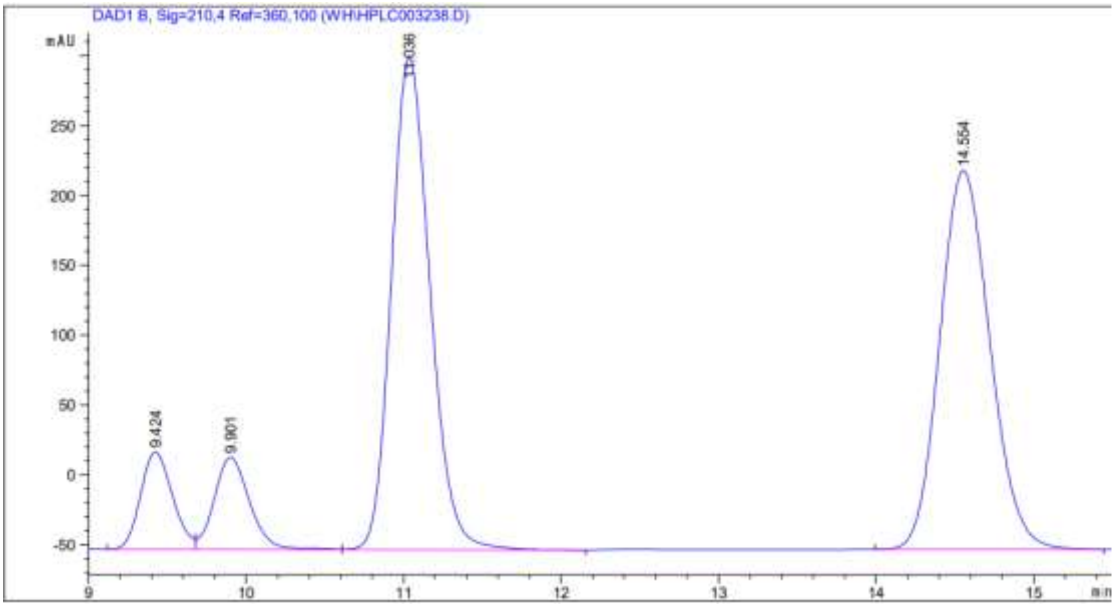

Peak	Ret. Time	Type	Width	Area	Height	Area
1	9.424	BV	0.2210	982.06696	69.45290	7.0200
2	9.901	VV	0.2397	1023.86493	65.74242	7.3188
3	11.036	VB	0.2627	5991.16211	351.78644	42.8261
4	14.554	BB	0.3465	5992.43164	270.46271	42.8351

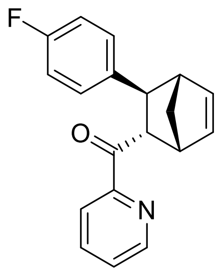

(+)3cA

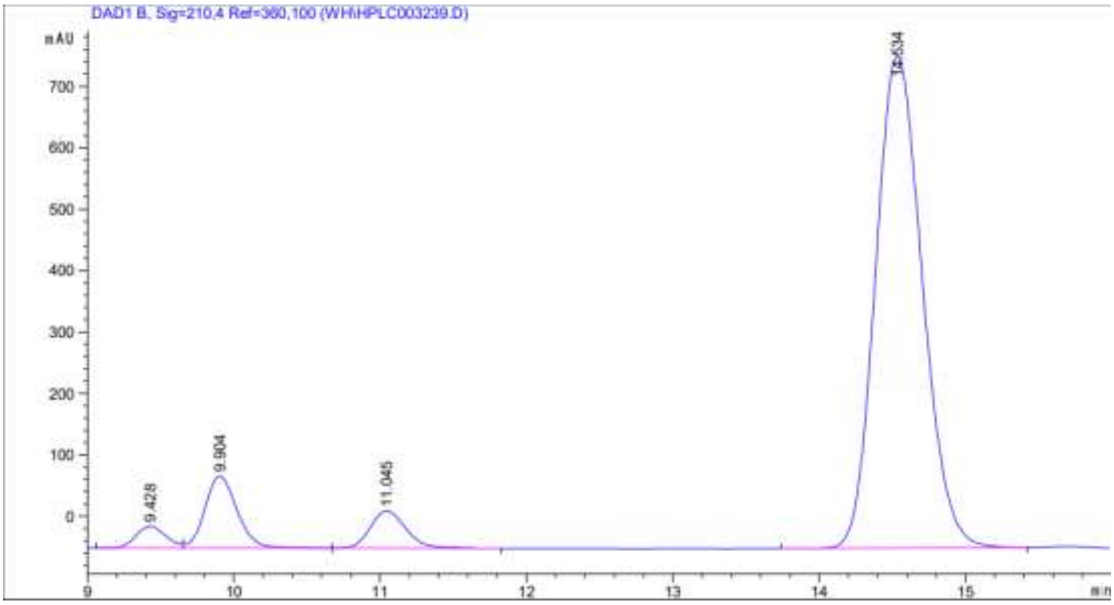

Peak	Ret. Time	Type	Width	Area	Height	Area
1	9.428	BV	0.2202	508.98660	35.72263	2.4092
2	9.904	VB	0.2361	1792.55371	117.43324	8.4847
3	11.045	BB	0.2617	1030.15784	60.79728	4.8761
4	14.534	BB	0.3488	1.77951e4	802.23138	84.2300

HPLC copies of compound (±)-3dA and (+)-3dA

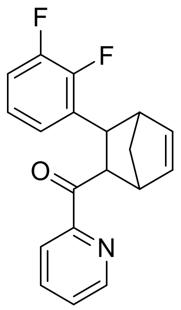

(±)3dA

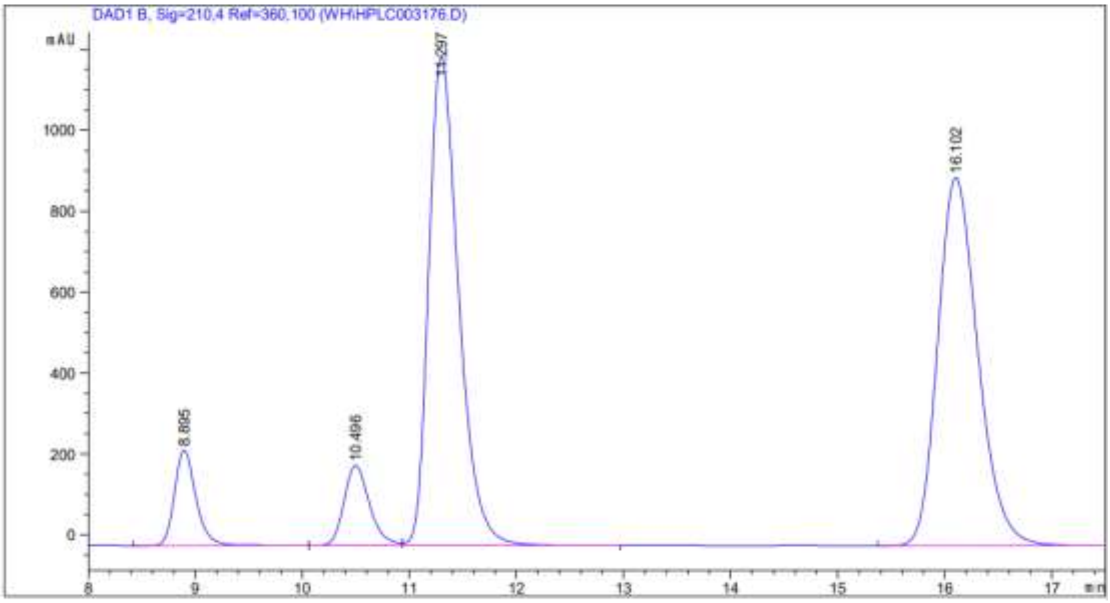

Peak	Ret. Time	Type	Width	Area	Height	Area
1	8.895	BB	0.2133	3301.12378	235.86511	6.1395
2	10.496	BV	0.2548	3272.77148	198.07375	6.0868
3	11.297	VB	0.2989	2.35380e4	1210.03369	43.7767
4	16.102	BBA	0.4013	2.36564e4	909.92371	43.9970

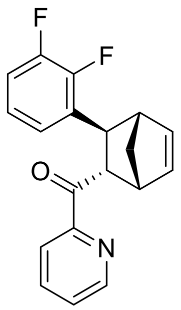

(+)3dA

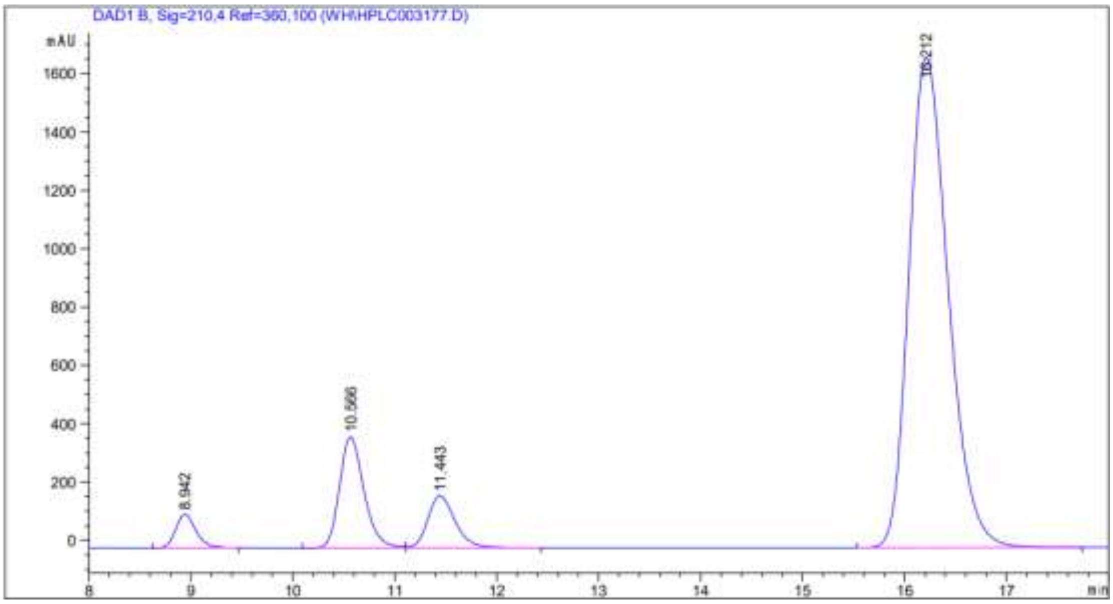

Peak	Ret. Time	Type	Width	Area	Height	Area
1	8.942	BB	0.2112	1592.05603	115.19073	2.8380
2	10.566	BV	0.2566	6348.96729	380.68417	11.3178
3	11.443	VB	0.2841	3305.66284	178.42763	5.8927
4	16.212	BB	0.4154	4.48506e4	1680.41394	79.9514

HPLC copies of compound (±)-3eA and (+)-3eA

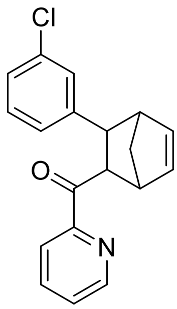

(±)3eA

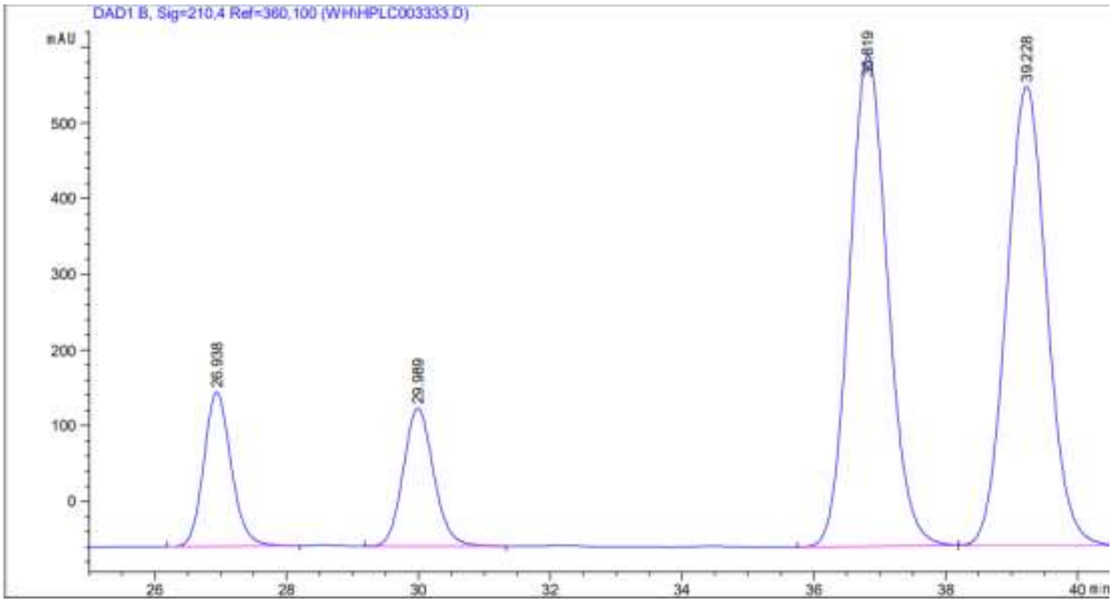

Peak	Ret. Time	Type	Width	Area	Height	Area
1	26.938	BB	0.4458	5895.80371	203.61391	9.2821
2	29.989	BB	0.4971	5857.50098	182.18330	9.2218
3	36.819	BB	0.6261	2.61264e4	648.38898	41.1322
4	39.228	BB	0.6572	2.56384e4	606.49249	40.3639

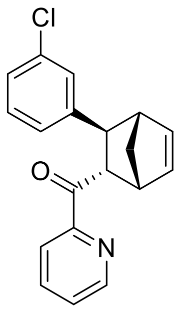

(+)3eA

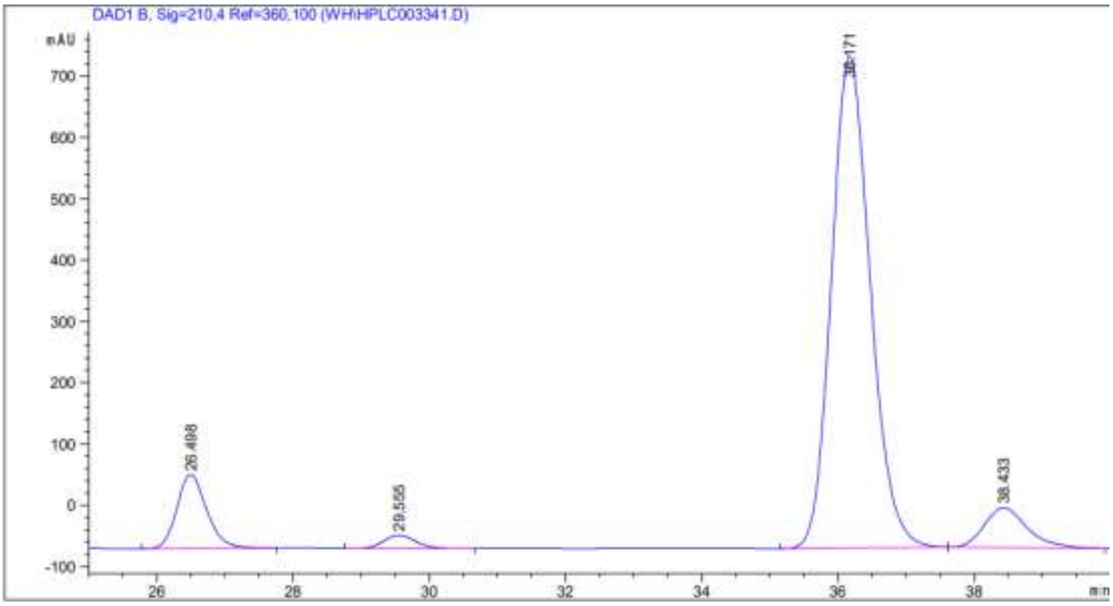

Peak	Ret. Time	Type	Width	Area	Height	Area
1	26.498	BB	0.4672	3665.80786	120.38799	9.4004
2	29.555	BB	0.4962	704.53369	21.73523	1.8067
3	36.171	BB	0.6145	3.17920e4	801.93719	81.5252
4	38.433	BB	0.6660	2834.16968	65.08999	7.2678

HPLC copies of compound (±)-3fA and (+)-3fA

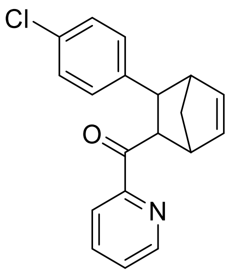

(±)3fA

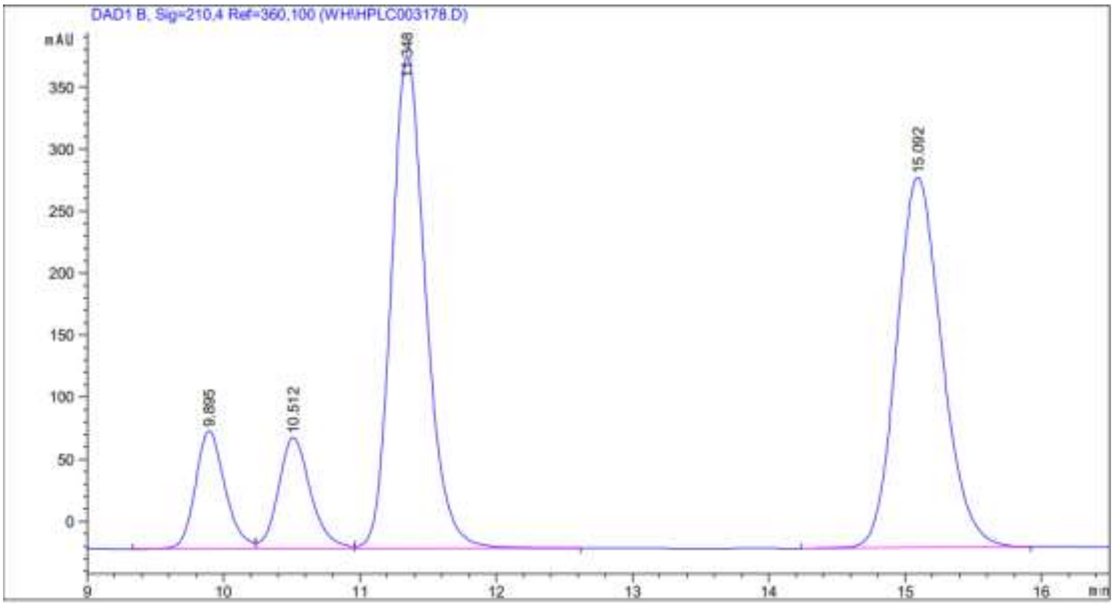

Peak	Ret. Time	Type	Width	Area	Height	Area
1	9.895	BV	0.2304	1434.57288	94.86388	8.5609
2	10.512	VV	0.2513	1468.36096	89.52876	8.7625
3	11.348	VB	0.2707	6956.37939	396.46887	41.5125
4	15.092	BB	0.3578	6897.98340	298.29584	41.1641

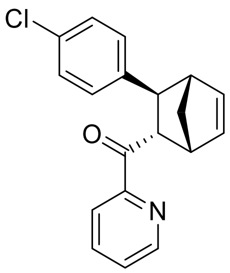

(+)3fA

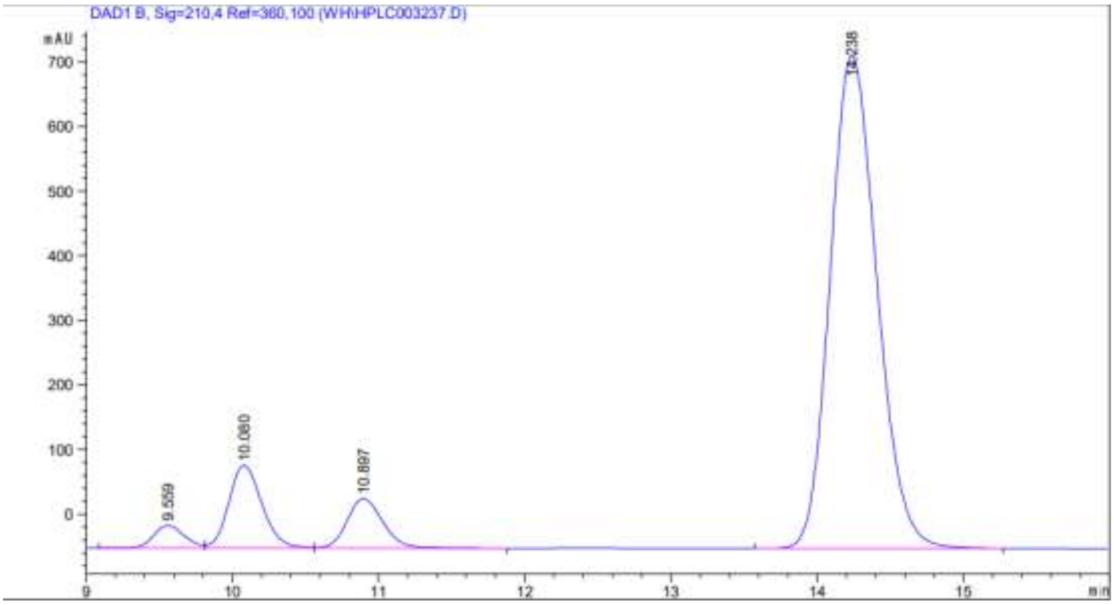

Peak	Ret. Time	Type	Width	Area	Height	Area
1	9.559	BV	0.2258	509.11459	34.98996	2.4535
2	10.080	VV	0.2433	2013.66040	128.15466	9.7042
3	10.897	VB	0.2633	1314.13318	76.91620	6.3330
4	14.238	BB	0.3466	1.69135e4	763.02411	81.5093

HPLC copies of compound (±)-3gA and (+)-3gA

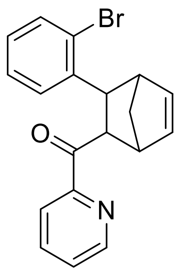

(±)3gA

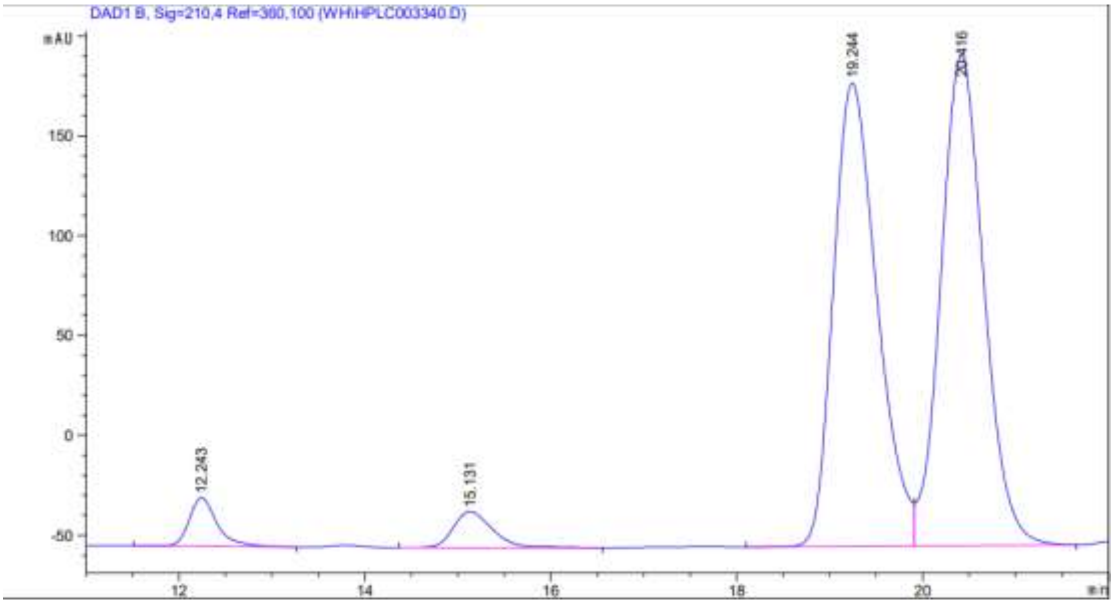

Peak	Ret. Time	Type	Width	Area	Height	Area
1	12.243	BB	0.3171	510.88876	24.31790	3.0943
2	15.131	BB	0.4512	531.50232	18.06563	3.2192
3	19.244	BV	0.5047	7572.17188	232.04483	45.8626
4	20.416	VB	0.4992	7896.00879	245.51259	47.8240

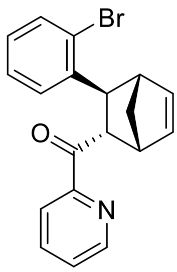

(+)3gA

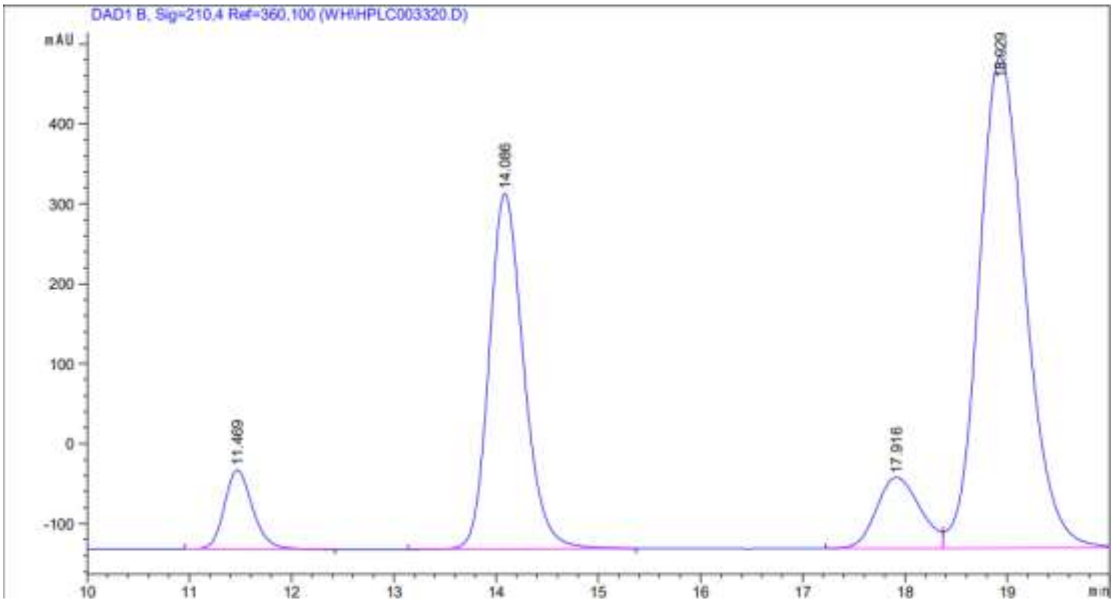

Peak	Ret. Time	Type	Width	Area	Height	Area
1	11.469	BB	0.2926	1887.37402	98.91158	5.5066
2	14.086	BB	0.3603	1.03681e4	444.22714	30.2503
3	17.916	BV	0.4618	2630.23560	88.72710	7.6740
4	18.929	VBA	0.4878	1.93888e4	615.03448	56.5691

HPLC copies of compound (±)-3hA and (+)-3hA

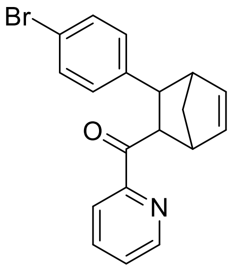

(±)3hA

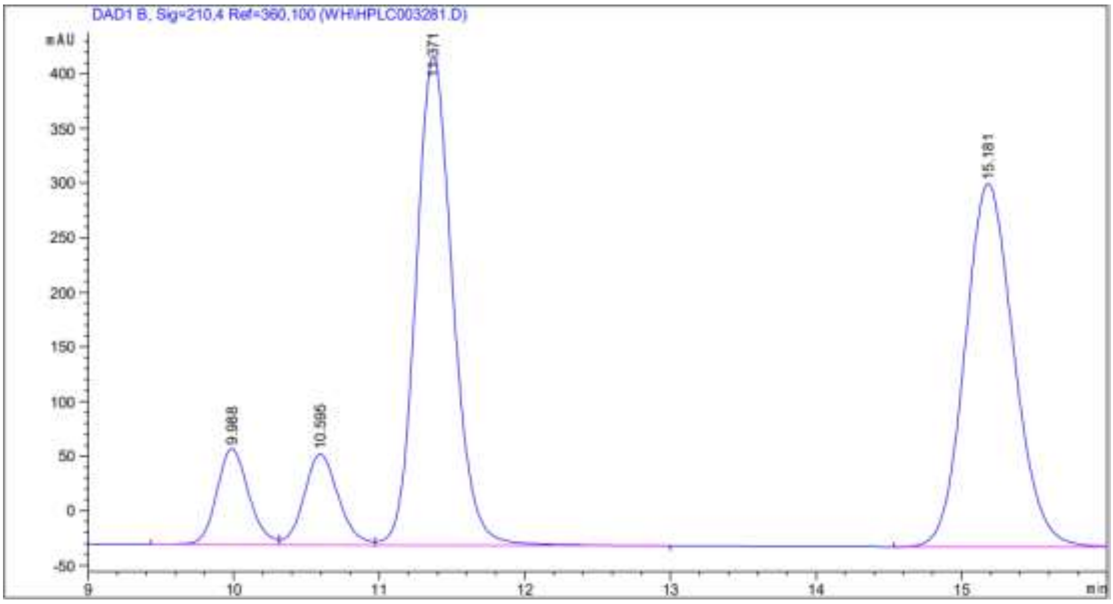

Peak	Ret. Time	Type	Width	Area	Height	Area
1	9.988	BV	0.2358	1336.82690	87.71903	7.3023
2	10.595	VV	0.2529	1359.65710	83.09606	7.4270
3	11.371	VB	0.2681	7833.34277	447.69308	42.7889
4	15.181	BBA	0.3629	7777.12891	332.49792	42.4818

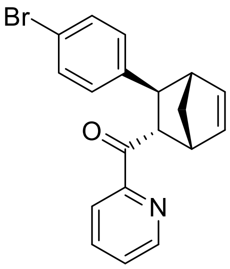

(+)3hA

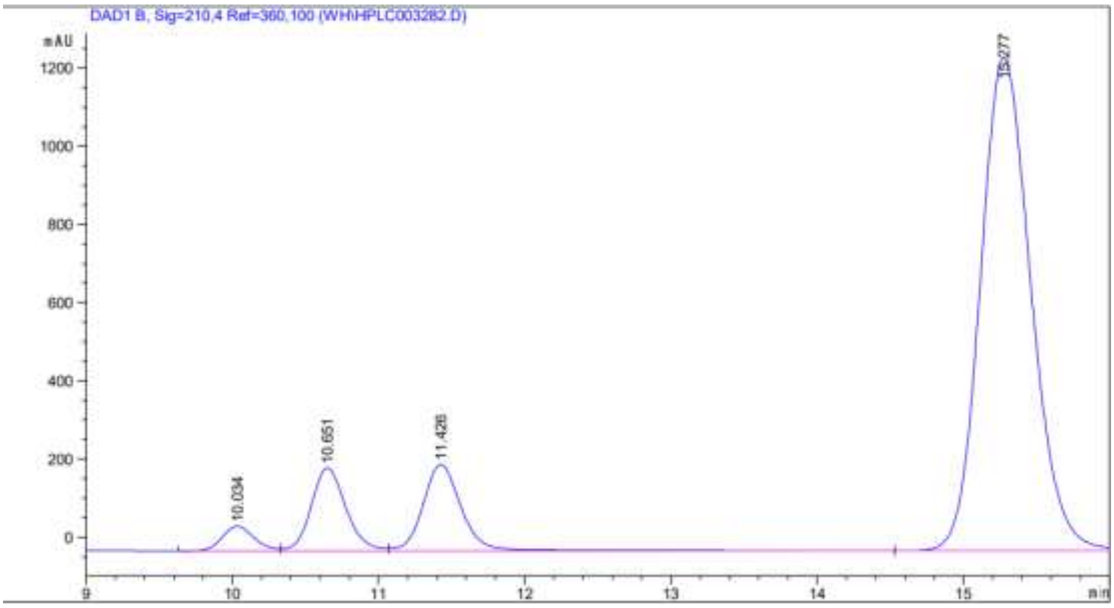

Peak	Ret. Time	Type	Width	Area	Height	Area
1	10.034	BV	0.2349	954.15656	62.92566	2.4548
2	10.651	VV	0.2515	3478.08154	211.87379	8.9483
3	11.426	VB	0.2849	4128.97754	220.01306	10.6229
4	15.277	BB	0.3723	3.03073e4	1261.45422	77.9739

HPLC copies of compound (±)-3iA and (+)-3iA

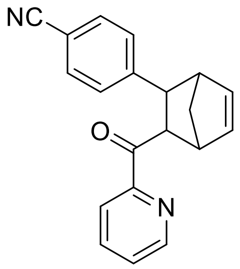

(±)3iA

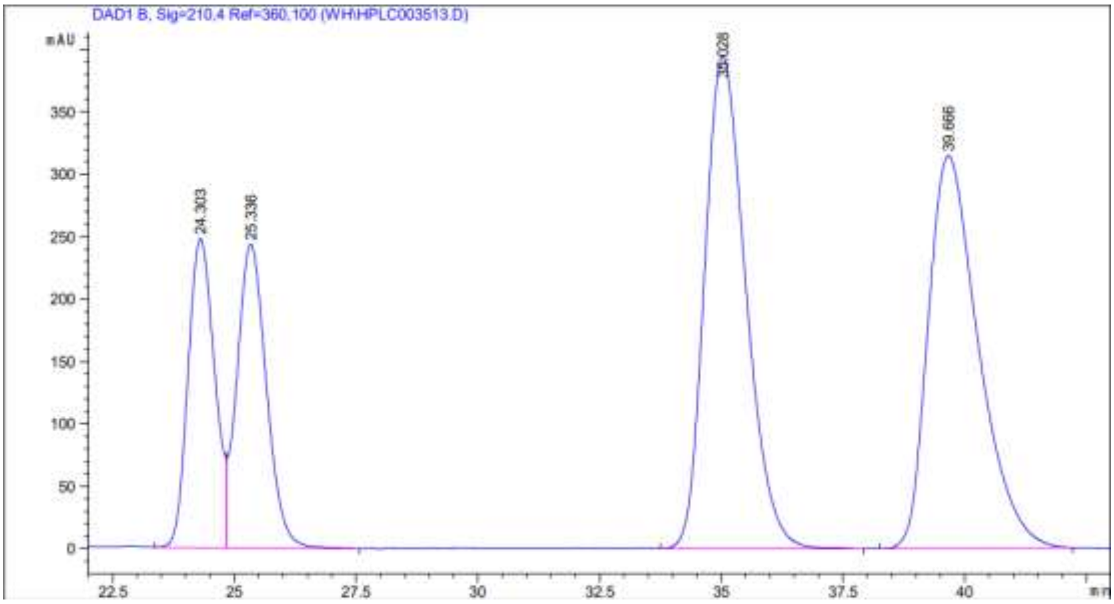

Peak	Ret. Time	Type	Width	Area	Height	Area
1	24.303	BV	0.5912	9358.27246	247.44434	14.3378
2	25.336	VB	0.6448	1.02337e4	243.26701	15.6790
3	35.028	BB	0.9110	2.31517e4	394.30707	35.4706
4	39.666	BB	1.0898	2.25264e4	314.44720	34.5126

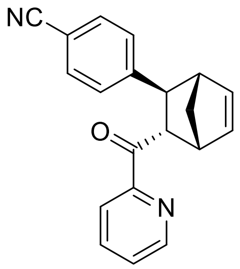

(+)3iA

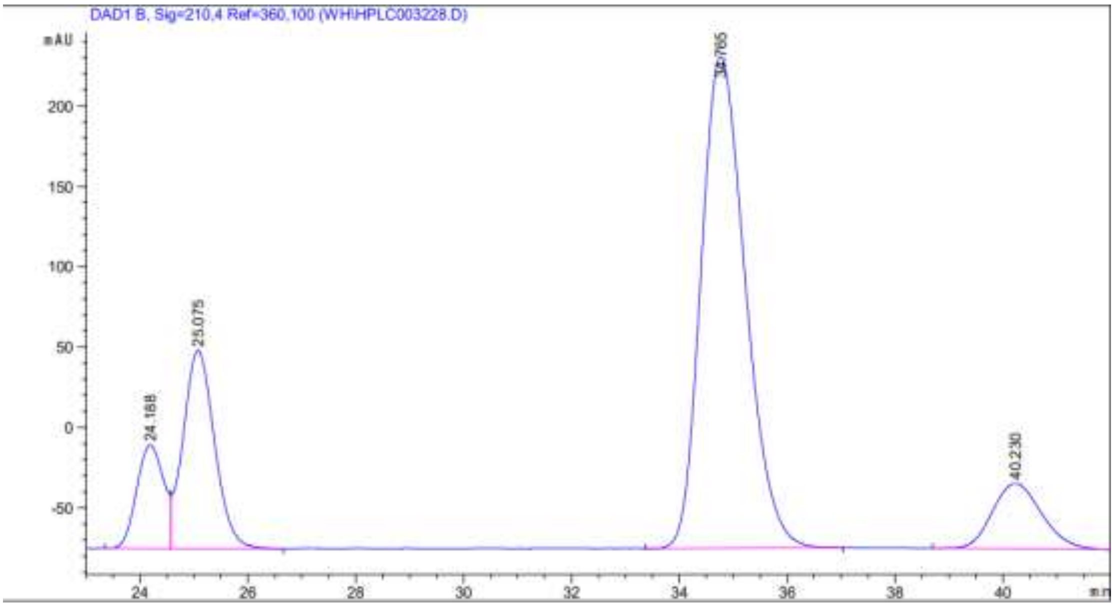

Peak	Ret. Time	Type	Width	Area	Height	Area
1	24.188	BV	0.5407	2246.55225	64.45193	8.2212
2	25.075	VB	0.6245	5016.08887	123.32589	18.3562
3	34.765	BB	0.8943	1.74744e4	305.95255	63.9470
4	40.230	BBA	0.9740	2589.32202	40.71352	9.4755

HPLC copies of compound (±)-3jA and (+)-3jA

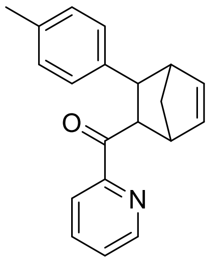

(±)3jA

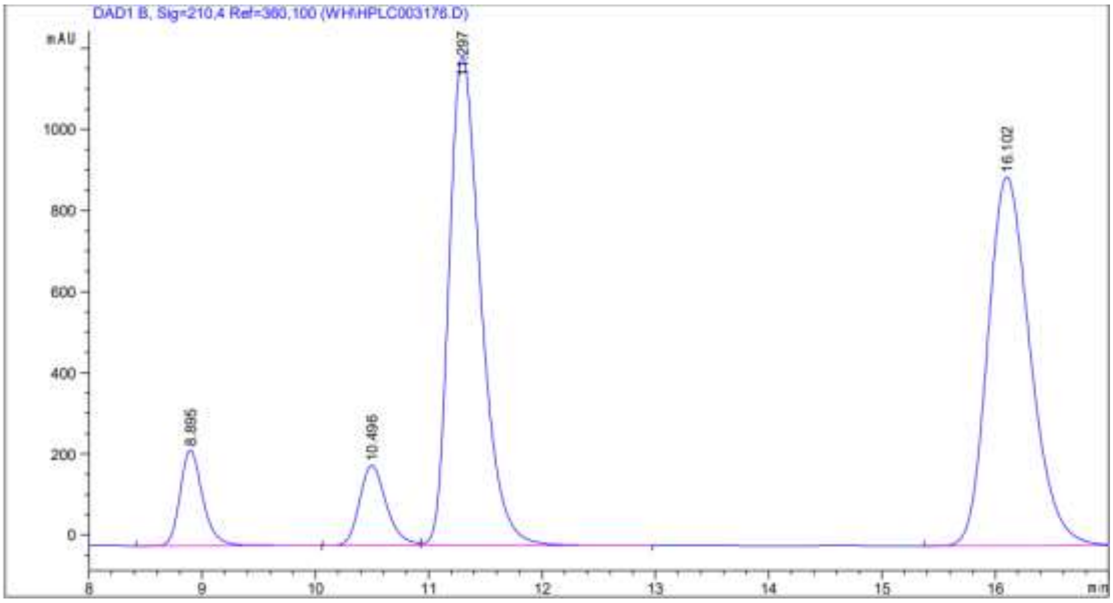

Peak	Ret. Time	Type	Width	Area	Height	Area
1	8.895	BB	0.2133	3301.12378	235.86511	6.1395
2	10.496	BV	0.2548	3272.77148	198.07375	6.0868
3	11.297	VB	0.2989	2.35380e4	1210.03369	43.7767
4	16.102	BBA	0.4013	2.36564e4	909.92371	43.9970

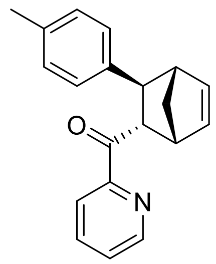

(+)3jA

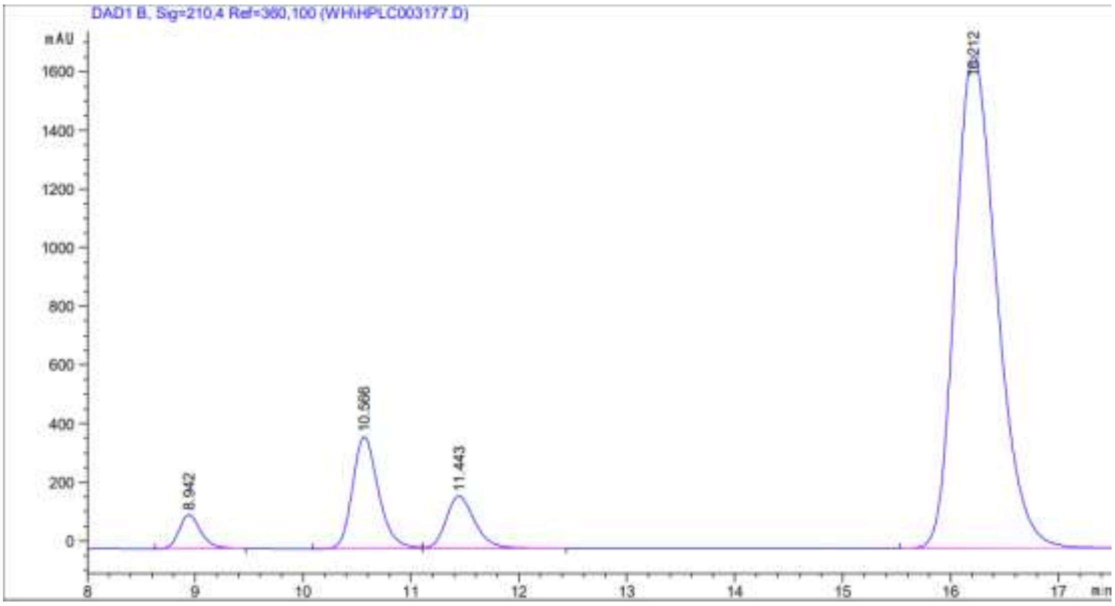

Peak	Ret. Time	Type	Width	Area	Height	Area
1	8.942	BB	0.2112	1592.05603	115.19073	2.8380
2	10.566	BV	0.2566	6348.96729	380.68417	11.3178
3	11.443	VB	0.2841	3305.66284	178.42763	5.8927
4	16.212	BB	0.4154	4.48506e4	1680.41394	79.9514

HPLC copies of compound (±)-3kA and (+)-3kA

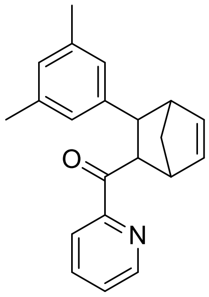

(±)3kA

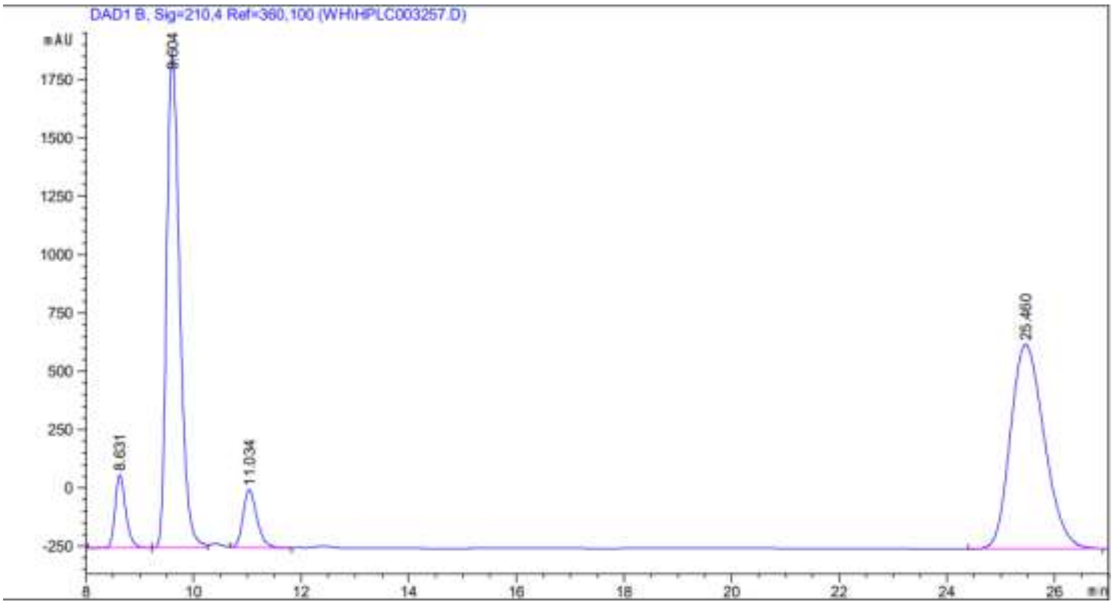

Peak	Ret. Time	Type	Width	Area	Height	Area
1	8.631	BB	0.2112	4330.30811	313.43591	5.2436
2	9.604	BV	0.2636	3.56734e4	2106.05859	43.1976
3	11.034	VB	0.2764	4474.19385	248.09222	5.4179
4	25.460	BB	0.6841	3.81041e4	874.98828	46.1409

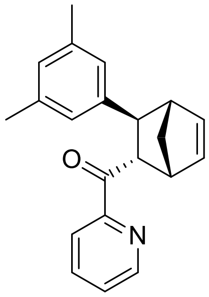

(+)3kA

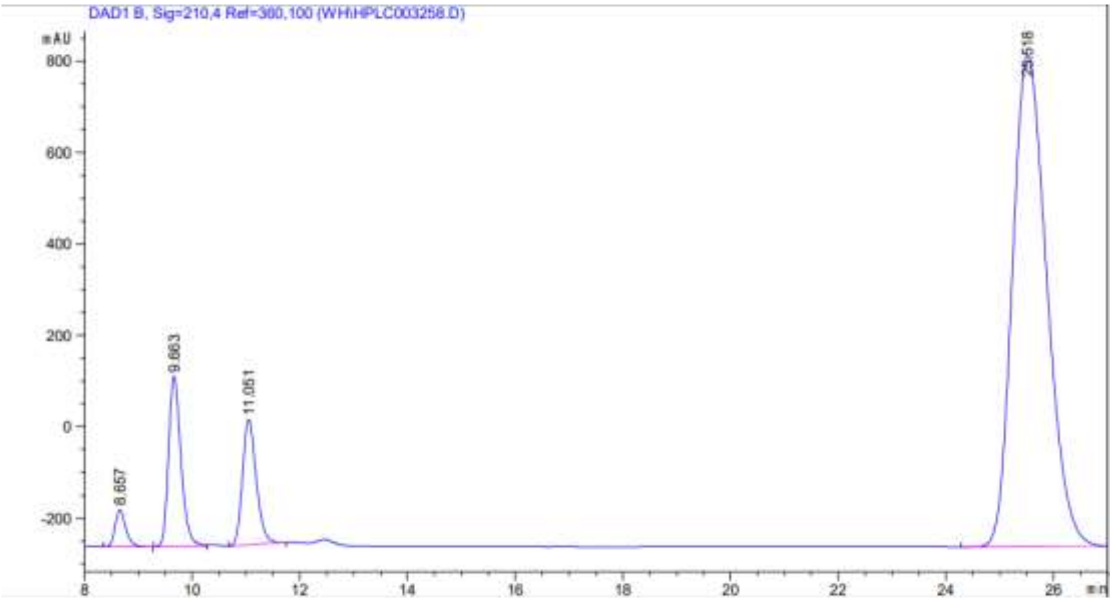

Peak	Ret. Time	Type	Width	Area	Height	Area
1	8.657	BB	0.2111	1105.67114	80.06611	1.8593
2	9.663	BV	0.2448	5957.05908	371.99872	10.0175
3	11.051	BB	0.2727	4858.52881	274.17764	8.1702
4	25.518	BBA	0.6977	4.75450e4	1075.53870	79.9529

HPLC copies of compound (±)-3lA and (+)-3lA

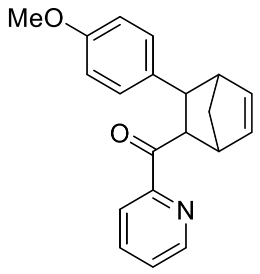

(±)3lA

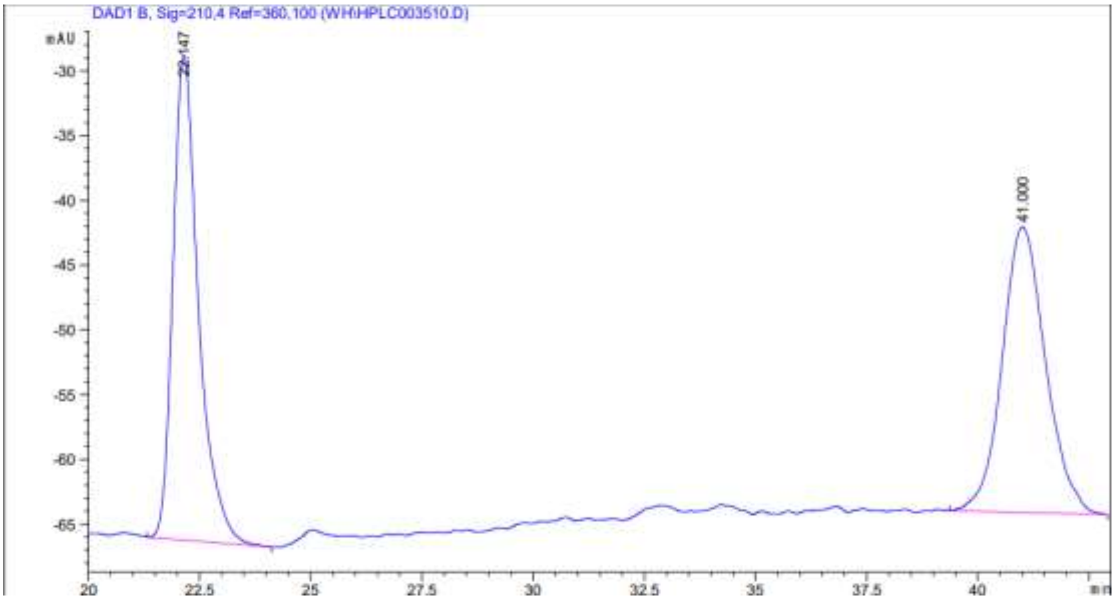

Peak	Ret. Time	Type	Width	Area	Height	Area
1	22.147	BB	0.6024	1496.41309	37.43166	50.0072
2	41.000	BBA	1.0294	1495.98169	22.05235	49.9928

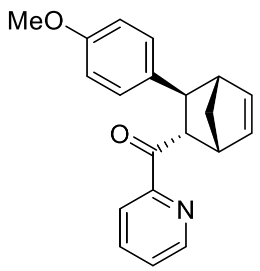

(+)3lA

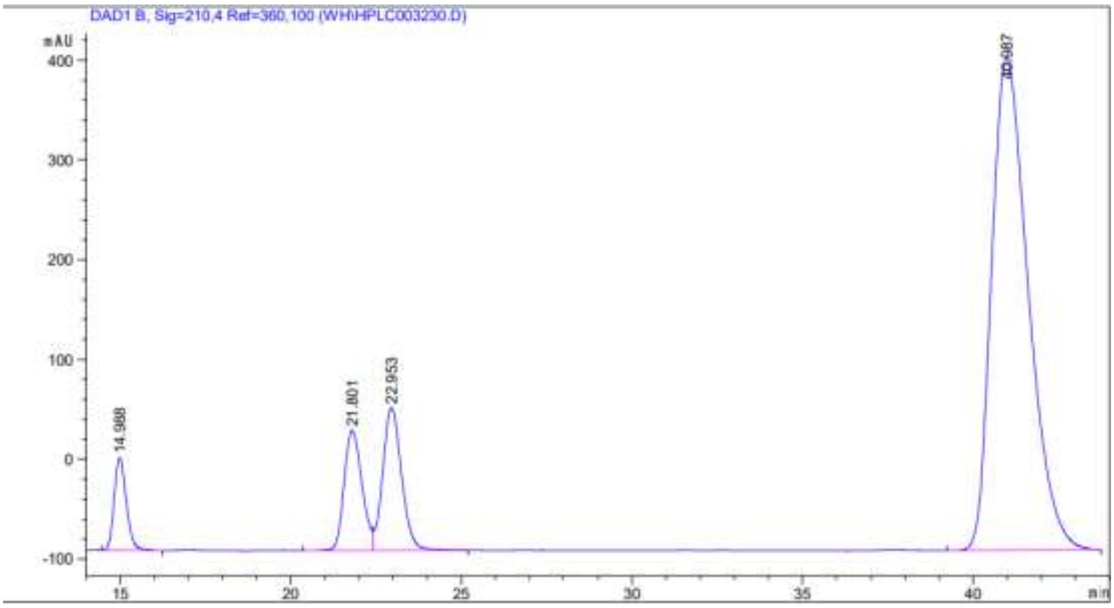

Peak	Ret. Time	Type	Width	Area	Height	Area
1	14.988	BB	0.3851	2295.14575	92.61149	4.6848
2	21.801	BV	0.5692	4392.52002	120.53552	8.9658
3	22.953	VB	0.5905	5499.12793	143.04382	11.2246
4	40.987	BBA	1.1496	3.68049e4	493.75140	75.1248

HPLC copies of compound (±)-3mA and (+)-3mA

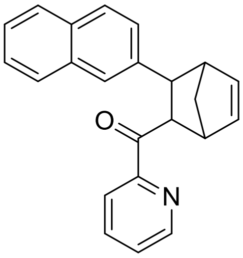

(±)3mA

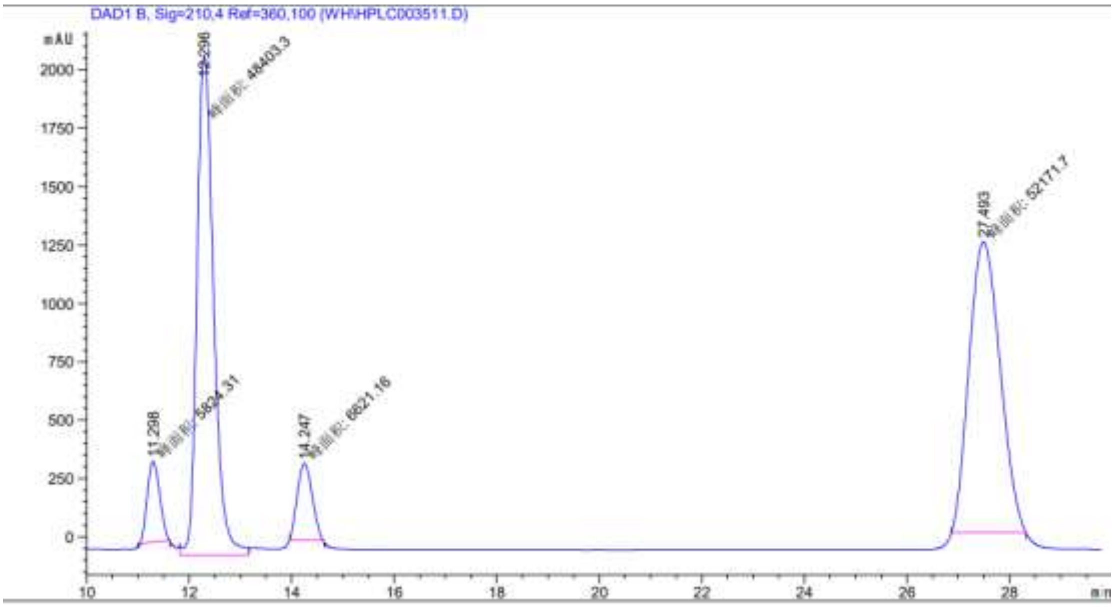

Peak	Ret. Time	Type	Width	Area	Height	Area
1	11.298	MM	0.2811	5824.31201	345.28146	5.1533
2	12.296	MM	0.3774	4.84033e4	2137.74268	42.8270
3	14.247	MM	0.3355	6621.16455	328.91306	5.8584
4	27.493	MM	0.6979	5.21717e4	1245.89954	46.1613

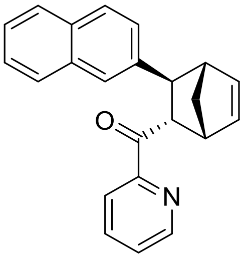

(+)3mA

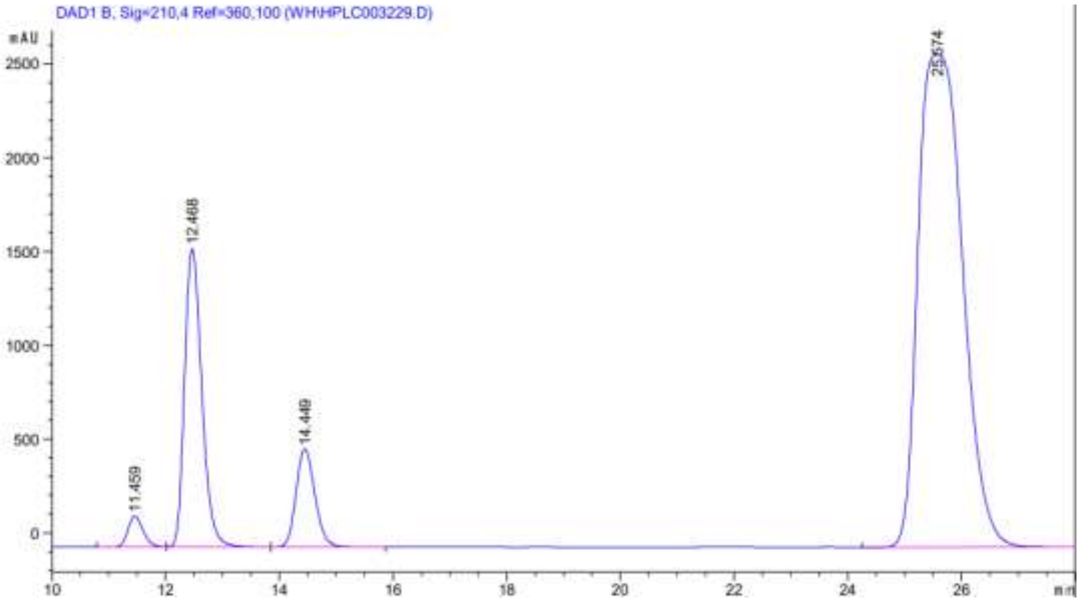

Peak	Ret. Time	Type	Width	Area	Height	Area
1	11.459	BV	0.2907	3081.22803	164.40118	1.6125
2	12.468	VB	0.3283	3.35788e4	1590.32324	17.5732
3	14.449	BB	0.3585	1.20327e4	522.86353	6.2972
4	25.574	BBA	0.7683	1.42386e5	2627.01294	74.5170

HPLC copies of compound (±)-3nA and (+)-3nA

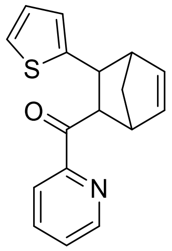

(±)3nA

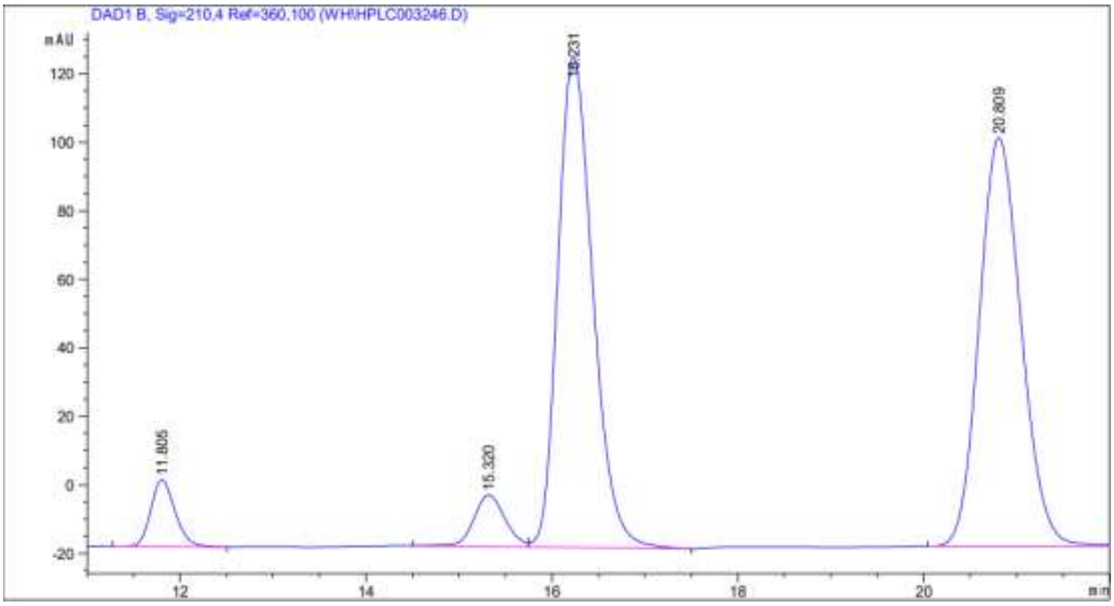

Peak	Ret. Time	Type	Width	Area	Height	Area
1	11.805	BB	0.2802	353.66711	19.44418	4.3159
2	15.320	BV	0.3637	353.48218	15.06580	4.3136
3	16.231	VB	0.4090	3748.41895	143.39276	45.7427
4	20.809	BB	0.4897	3738.99634	119.29633	45.6278

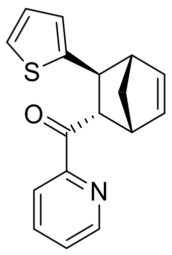

(+)3nA

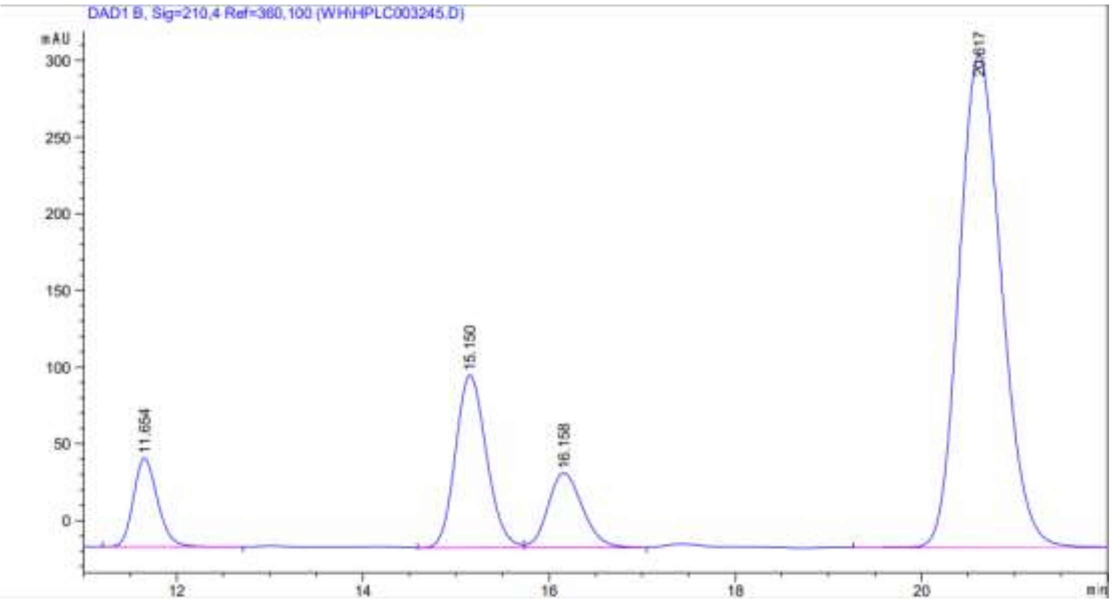

Peak	Ret. Time	Type	Width	Area	Height	Area
1	11.654	BB	0.2780	1037.21313	57.61157	6.8855
2	15.150	BV	0.3603	2606.66943	112.51872	17.3042
3	16.158	VB	0.4000	1248.36658	48.54549	8.2872
4	20.617	BB	0.4937	1.01715e4	321.02692	67.5231

HPLC copies of compound (±)-3oA and (+)-3oA

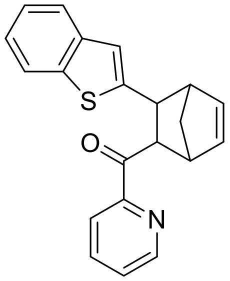

(±)3oA

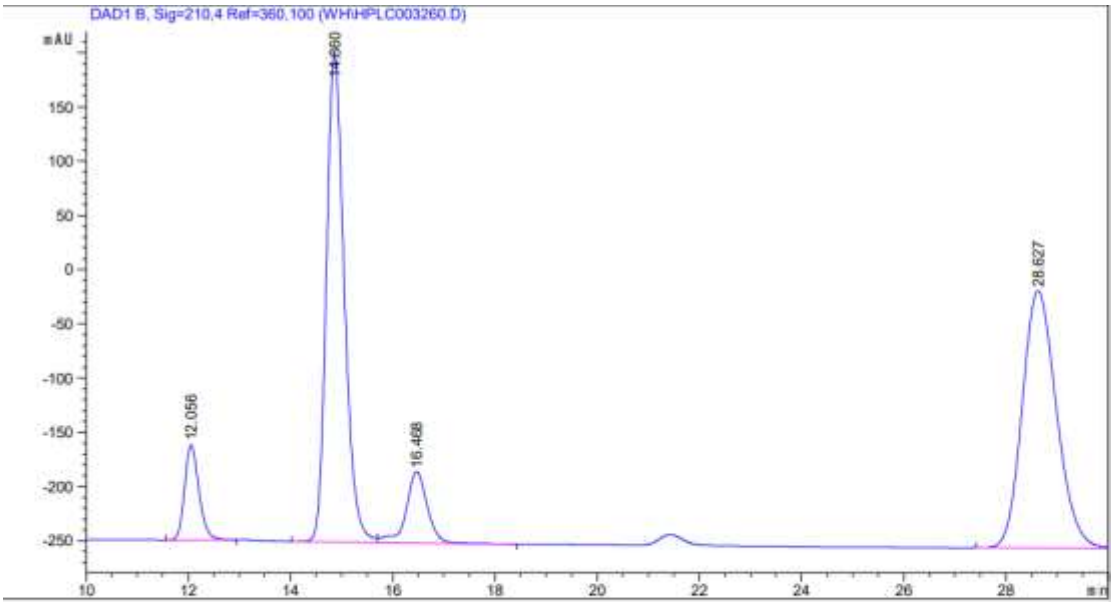

Peak	Ret. Time	Type	Width	Area	Height	Area
1	12.056	BB	0.2998	1711.71265	87.66868	6.6648
2	14.860	BV	0.3775	1.09678e4	448.15283	42.7045
3	16.468	VB	0.4395	1917.39136	65.89531	7.4656
4	28.627	BBA	0.7272	1.10861e4	237.21581	43.1651

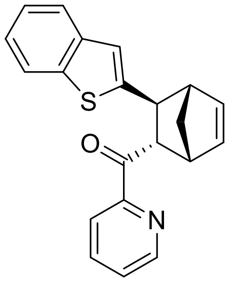

(+)3oA

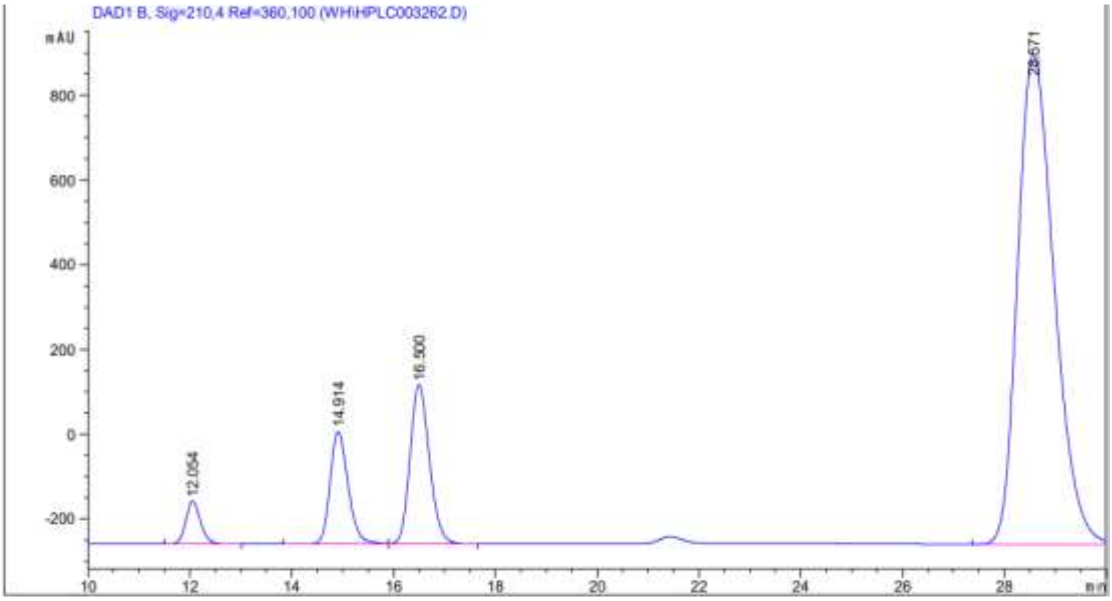

Peak	Ret. Time	Type	Width	Area	Height	Area
1	12.054	BB	0.3079	2024.54065	100.98044	2.6590
2	14.914	BV	0.3801	6558.66699	265.56158	8.6140
3	16.500	VB	0.4081	9932.83887	376.12924	13.0456
4	28.571	BB	0.7791	5.76232e4	1156.81433	75.6813

HPLC copies of compound (±)-3pA and (+)-3pA

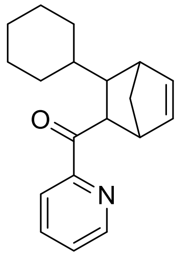

(±)3pA

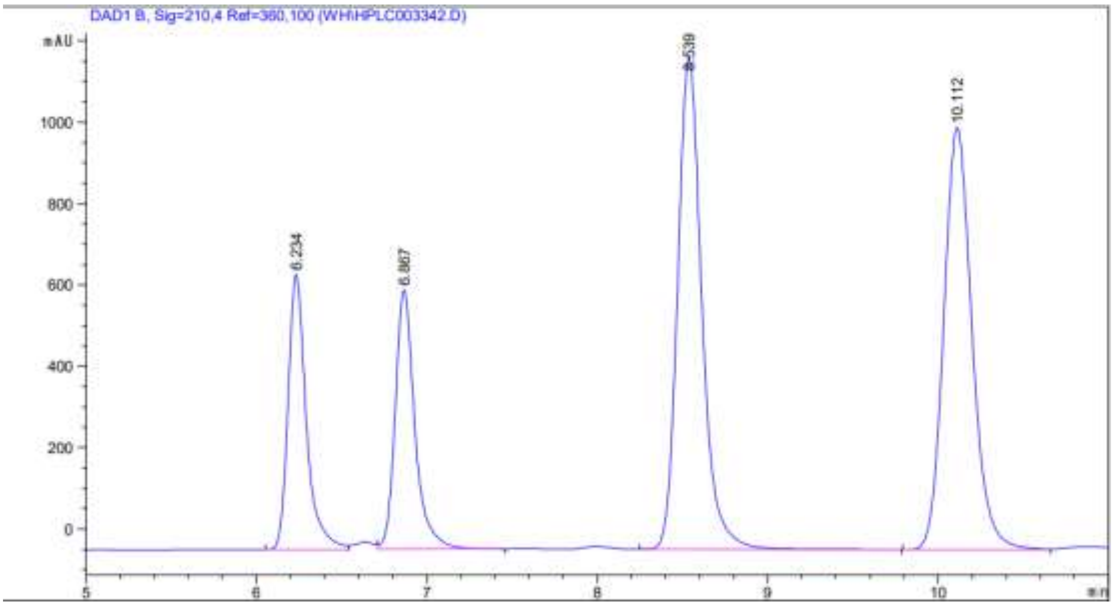

Peak	Ret. Time	Type	Width	Area	Height	Area
1	6.234	BV	0.1127	5019.05859	675.56696	14.7673
2	6.867	VB	0.1216	5111.50977	636.78082	15.0393
3	8.539	VB	0.1513	1.19489e4	1208.90906	35.1567
4	10.112	BV	0.1780	1.19081e4	1036.88074	35.0366

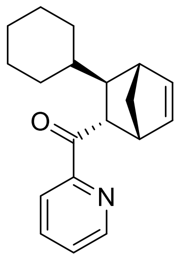

(+)3pA

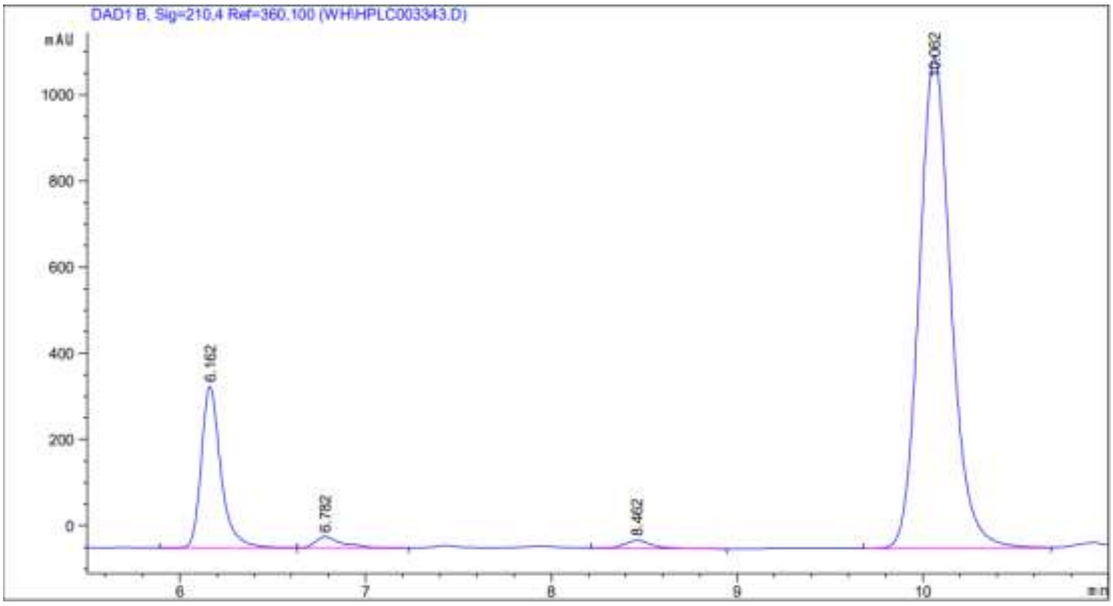

Peak	Ret. Time	Type	Width	Area	Height	Area
1	6.162	BV	0.1118	2759.23071	374.99643	16.4276
2	6.782	VB	0.1454	262.72296	26.11018	1.5642
3	8.462	BB	0.1526	181.16037	18.12891	1.0786
4	10.062	BV	0.1829	1.35932e4	1141.89087	80.9296

HPLC copies of compound (±)-3qA and (+)-3qA

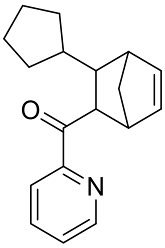

(±)3qA

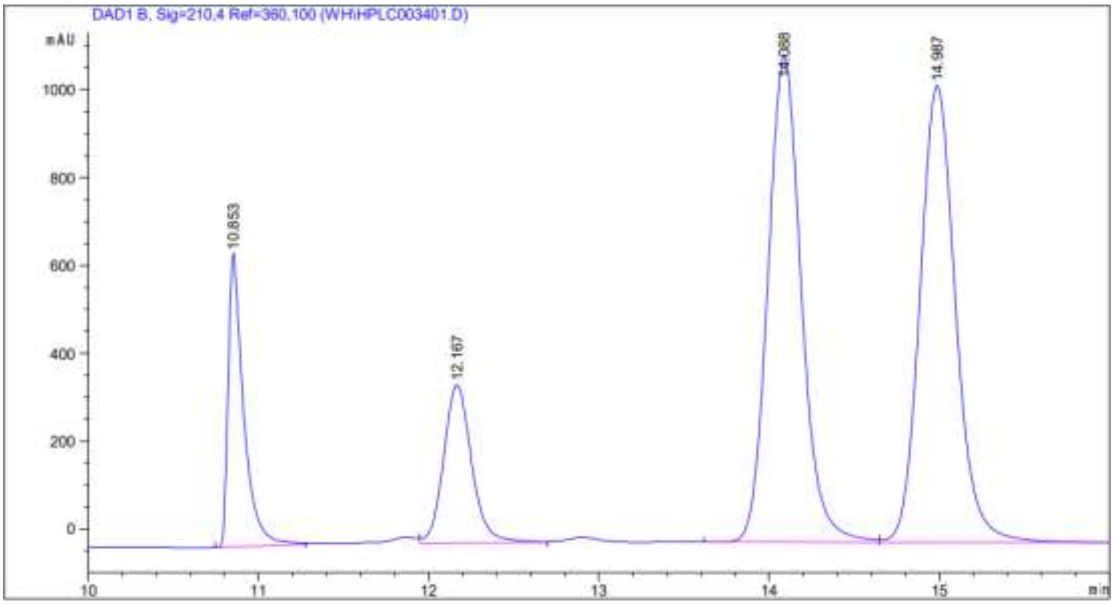

Peak	Ret. Time	Type	Width	Area	Height	Area
1	10.853	BV	0.0892	4189.71777	667.63000	10.8722
2	12.167	VV	0.1755	4108.54395	359.16403	10.6616
3	14.088	BV	0.2093	1.51104e4	1106.41772	39.2111
4	14.987	VB	0.2237	1.51274e4	1039.87561	39.2551

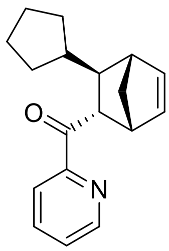

(+)3qA

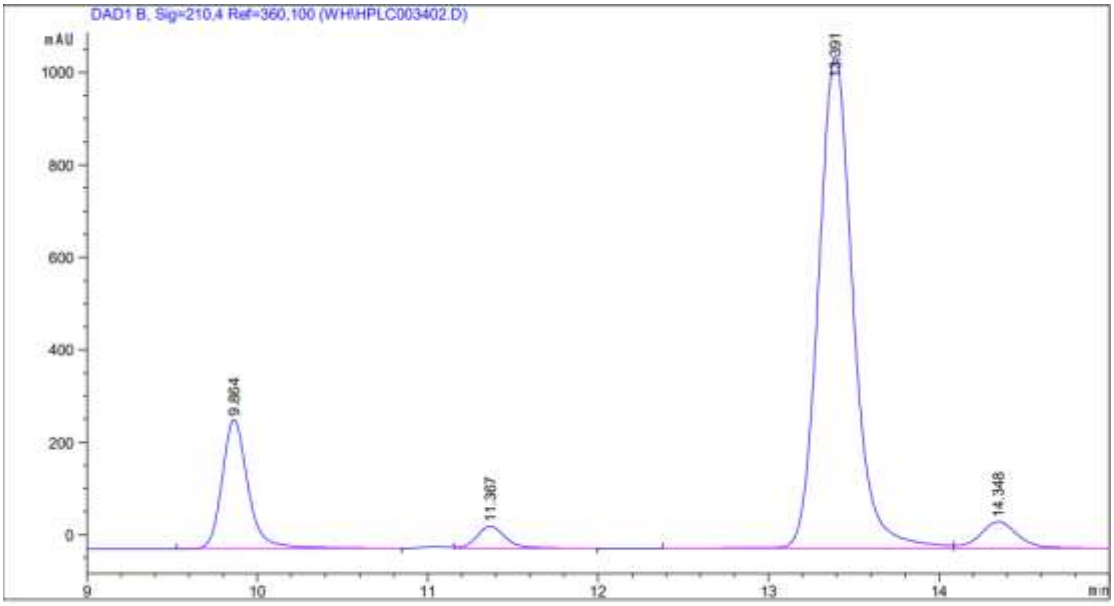

Peak	Ret. Time	Type	Width	Area	Height	Area
1	9.864	BV	0.1618	2962.91846	279.20105	15.2533
2	11.367	VB	0.1796	566.14777	47.99360	2.9146
3	13.391	BV	0.2163	1.50051e4	1065.36328	77.2475
4	14.348	VB	0.2339	890.54810	57.74794	4.5846

HPLC copies of compound (±)-3rA and (+)-3rA

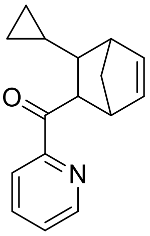

(±)3rA

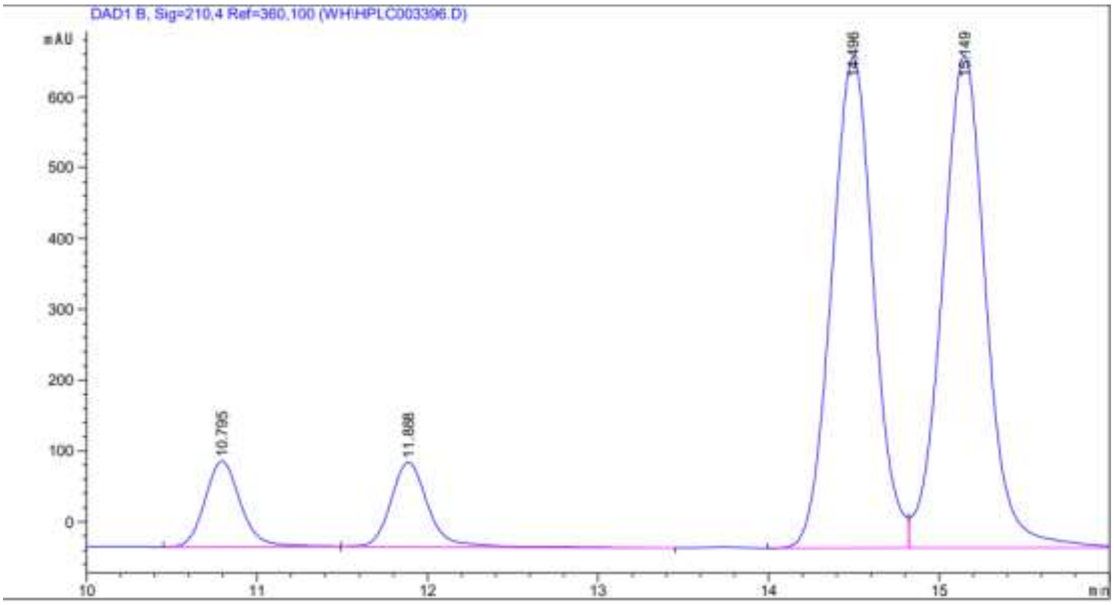

Peak	Ret. Time	Type	Width	Area	Height	Area
1	10.795	BB	0.2217	1734.79773	120.66353	6.2350
2	11.888	BB	0.2295	1790.64905	119.05792	6.4357
3	14.496	BV	0.2631	1.18346e4	693.34631	42.5346
4	15.149	VBA	0.2751	1.24634e4	695.30939	44.7946

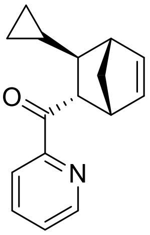

(+)3rA

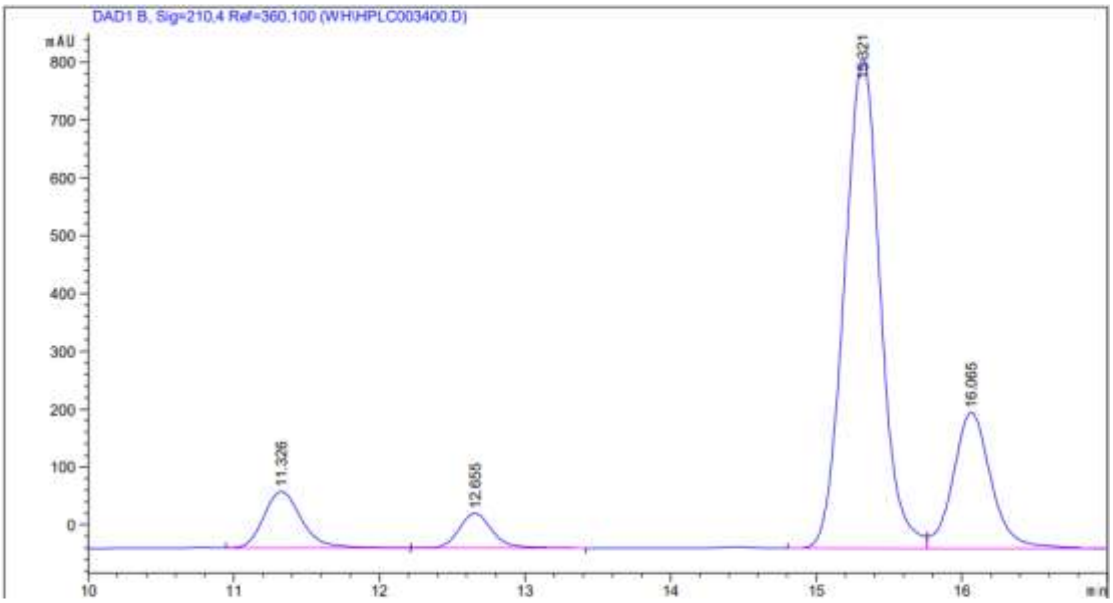

Peak	Ret. Time	Type	Width	Area	Height	Area
1	11.326	VB	0.2786	1759.27808	97.47600	8.1594
2	12.655	BB	0.2408	938.33496	59.89938	4.3519
3	15.321	BV	0.2657	1.45263e4	848.63776	67.3720
4	16.065	VB	0.2786	4337.42334	235.77260	20.1166

HPLC copies of compound (±)-3aB and (+)-3aB

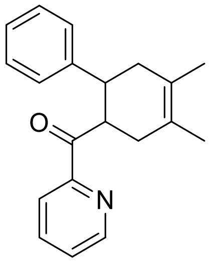

(±)3aB

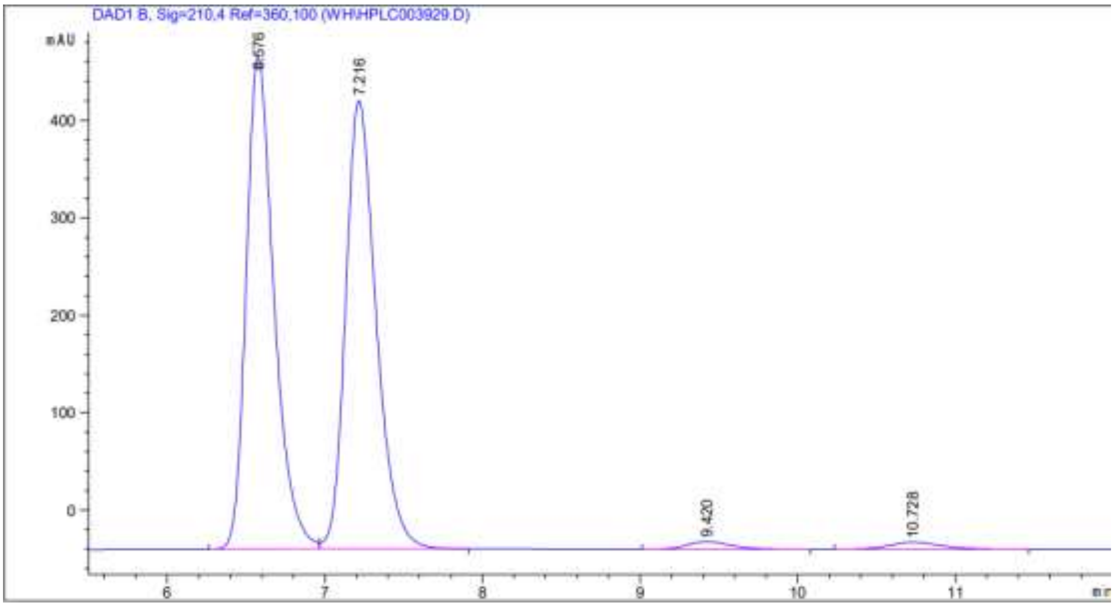

Peak	Ret. Time	Type	Width	Area	Height	Area
1	6.576	BV	0.1873	6215.53125	506.10355	48.7906
2	7.216	VB	0.2052	6204.85107	460.54266	48.7067
3	9.420	BB	0.2908	149.08392	7.87837	1.1703
4	10.728	BB	0.3573	169.73848	7.24493	1.3324

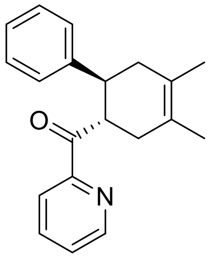

(+)3aB

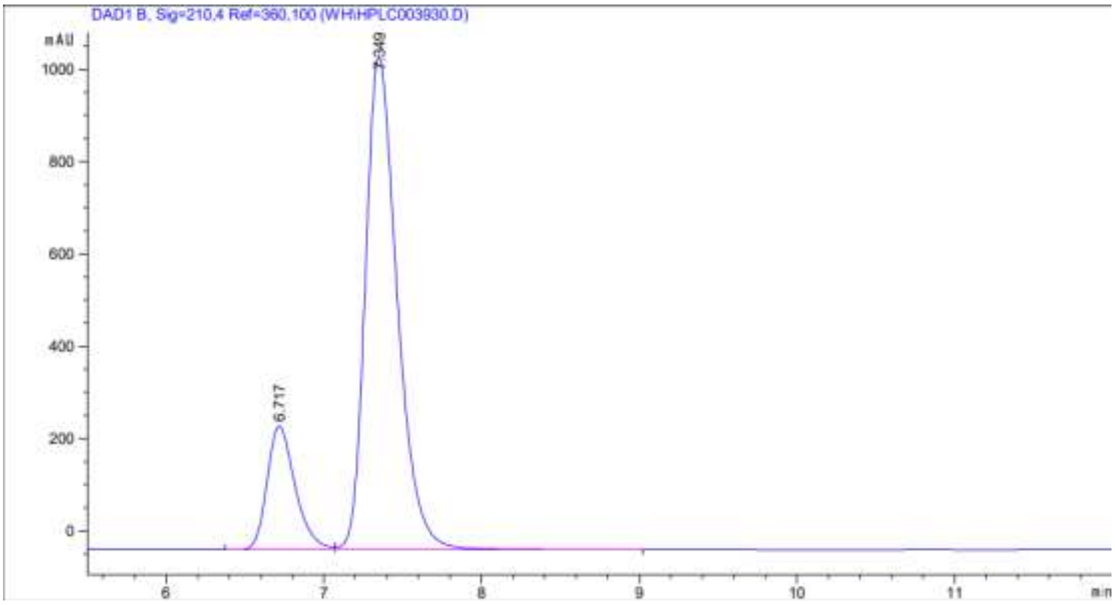

Peak	Ret. Time	Type	Width	Area	Height	Area
1	6.717	BV	0.1884	3320.90112	268.24289	18.1177
2	7.349	VB	0.2158	1.50087e4	1068.76050	81.8823

3.2 HPLC copies of optimization of the reaction conditions.

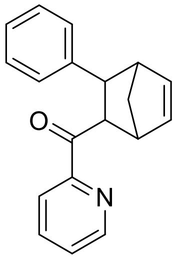

(±)3aA

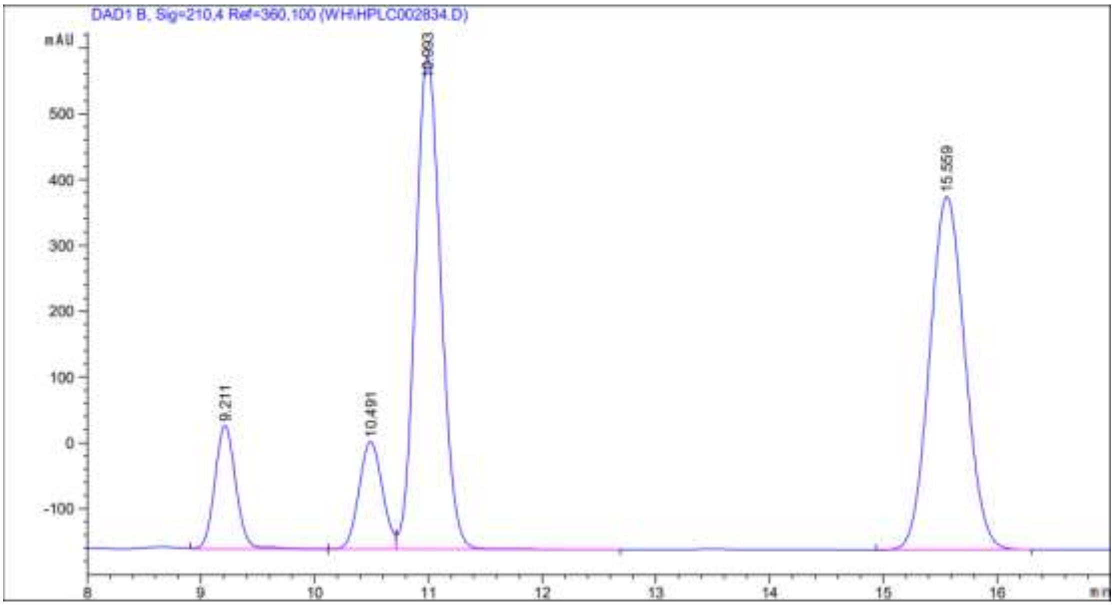

Peak #	Ret. Time [min]	Type	Width [min]	Area [mAU*s]	Height [mAU]	Area %
1	9.211	VB	0.1997	2403.19849	187.32208	8.6251
2	10.491	BV	0.2219	2326.81860	163.60168	8.3510
3	10.993	VB	0.2425	1.15814e4	748.54620	41.5656
4	15.559	BB	0.3372	1.15515e4	536.57239	41.4584

Optimization of PyBim-1

La(OTf)$_3$ (10 mol%), PyBim-1 (12 mol%), N_2, 25 °C, THF, 48 h

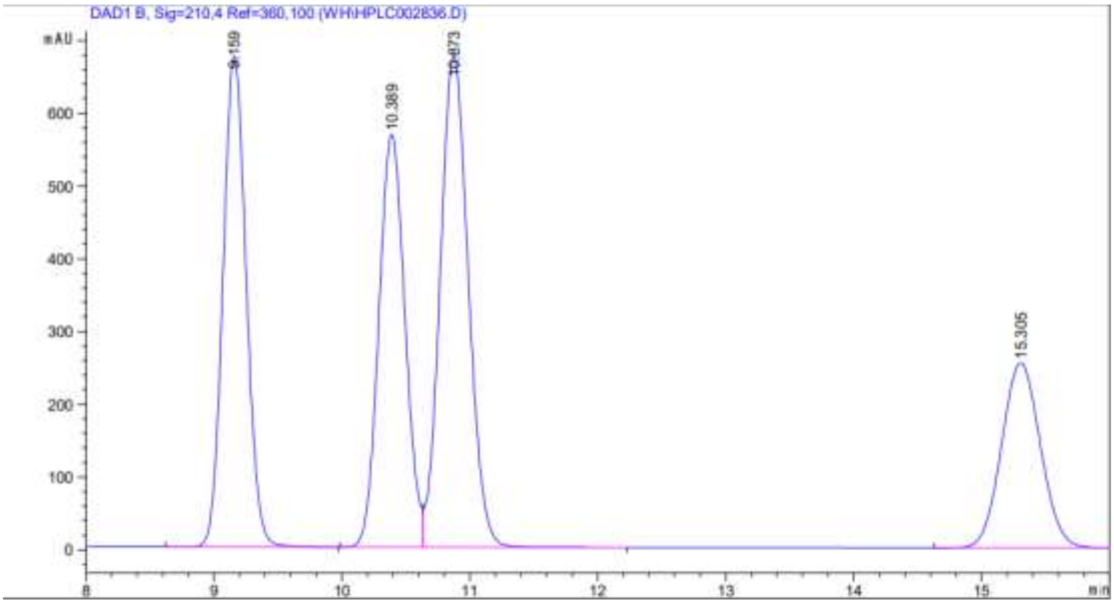

Peak	Ret. Time	Type	Width	Area	Height	Area
1	9.159	BB	0.1969	8494.58594	674.79004	26.3237
2	10.389	BV	0.2216	8054.78271	567.46637	24.9608
3	10.873	VB	0.2405	1.03427e4	676.15057	32.0506
4	15.305	BB	0.3308	5377.72168	254.25807	16.6649

La(OTf)$_3$ (10 mol%), PyBim-1 (12 mol%), N_2, 0 °C, THF, 48 h

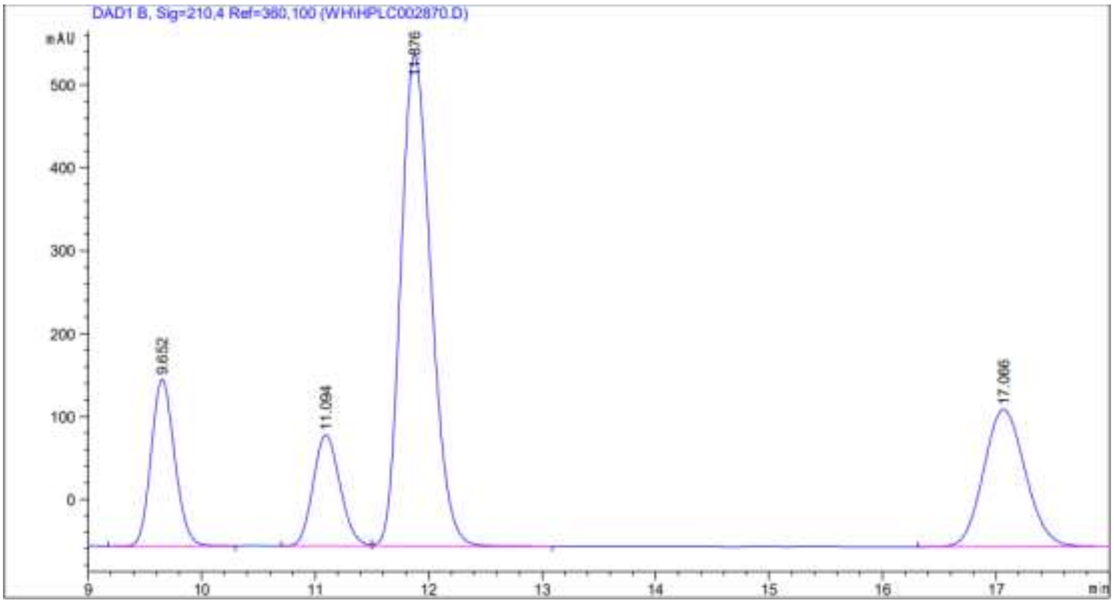

Peak	Ret. Time	Type	Width	Area	Height	Area
1	9.652	BB	0.2238	2894.84155	201.25471	14.2545
2	11.094	BV	0.2592	2222.65747	134.22163	10.9446
3	11.876	VB	0.2900	1.09744e4	592.76593	54.0393
4	17.066	BB	0.3993	4216.32178	165.45448	20.7616

Pr(OTf)$_3$ (10 mol%), PyBim-1 (12 mol%), N_2, 25 °C, THF, 48 h

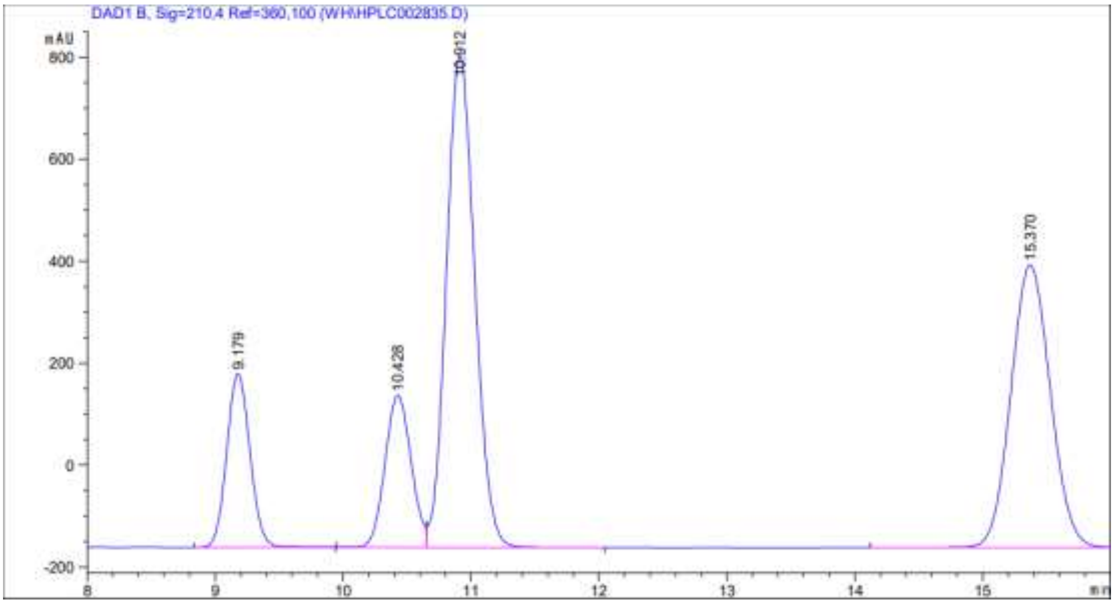

Peak	Ret. Time	Type	Width	Area	Height	Area
1	9.179	BB	0.1970	4290.80566	340.57648	12.1272
2	10.428	BV	0.2236	4233.00977	298.18628	11.9639
3	10.912	VB	0.2421	1.49088e4	965.57233	42.1372
4	15.370	BB	0.3373	1.19490e4	554.72888	33.7717

Ce(OTf)$_3$ (10 mol%), PyBim-1 (12 mol%), N_2, 25 °C, THF, 48 h

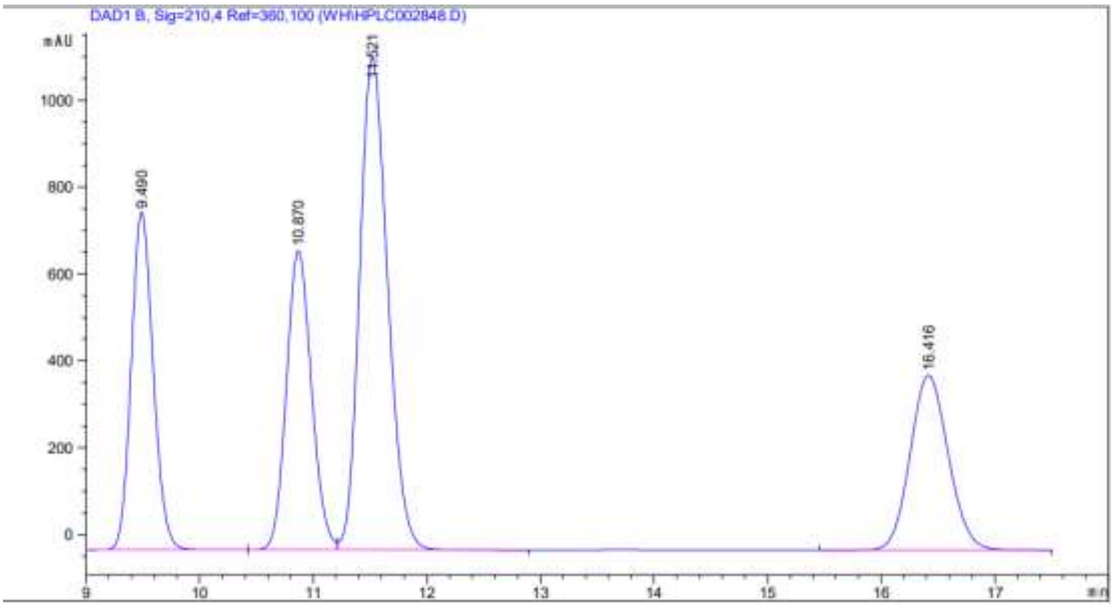

Peak	Ret. Time	Type	Width	Area	Height	Area
1	9.490	BB	0.2085	1.04334e4	777.68109	20.9401
2	10.870	BV	0.2391	1.05714e4	688.71698	21.2170
3	11.521	VB	0.2682	1.94422e4	1133.07043	39.0212
4	16.416	BBA	0.3640	9377.89355	402.21976	18.8217

Sm(OTf)$_3$ (10 mol%), PyBim-1 (12 mol%), N_2, 25 °C, THF, 48 h

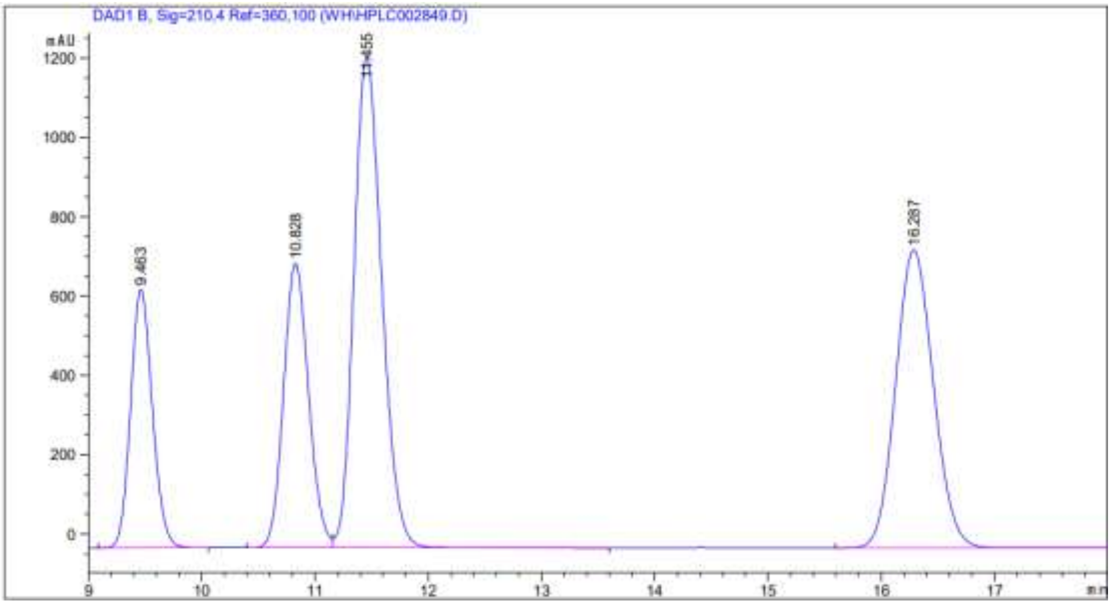

Peak	Ret. Time	Type	Width	Area	Height	Area
1	9.463	BB	0.2057	8693.43848	651.59631	14.9257
2	10.828	BV	0.2368	1.09874e4	716.92188	18.8642
3	11.455	VB	0.2676	2.11624e4	1236.99756	36.3336
4	16.287	BBA	0.3603	1.74015e4	751.02069	29.8765

Sc(OTf)$_3$ (10 mol%), PyBim-1 (12 mol%), N_2, 25 °C, THF, 48 h

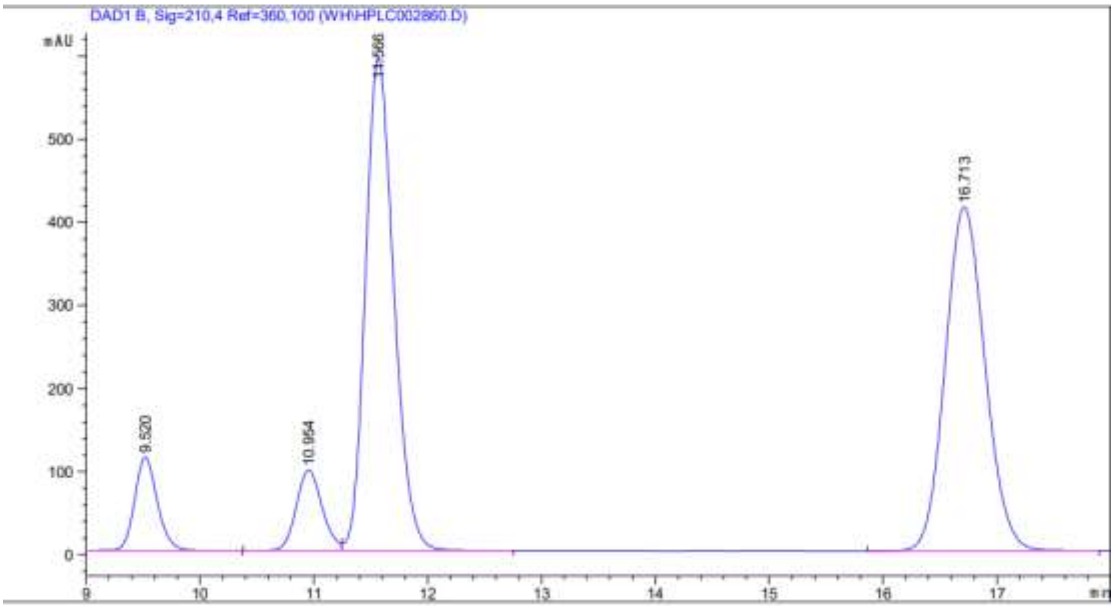

Peak	Ret. Time	Type	Width	Area	Height	Area
1	9.520	BB	0.2137	1558.98059	112.44801	6.6667
2	10.954	BV	0.2421	1524.98328	96.63659	6.5213
3	11.566	VB	0.2683	1.02798e4	592.94055	43.9593
4	16.713	BB	0.3768	1.00210e4	413.40100	42.8527

Yb(OTf)$_3$ (10 mol%), PyBim-1 (12 mol%), N_2, 25 °C, THF, 48 h

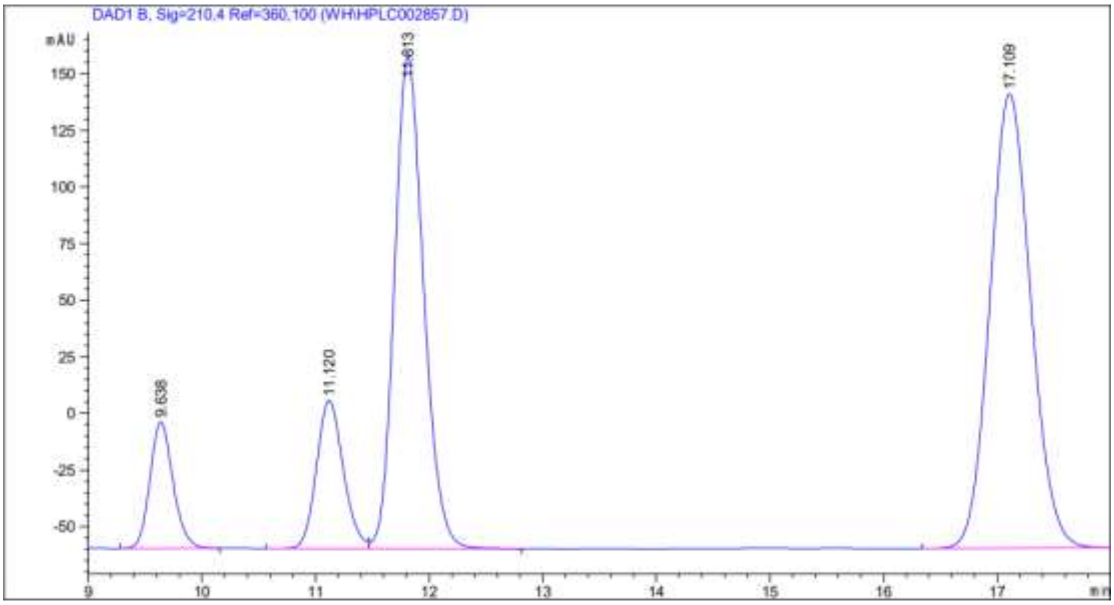

Peak	Ret. Time	Type	Width	Area	Height	Area
1	9.638	BB	0.2163	787.39093	55.89711	7.3194
2	11.120	BV	0.2535	1073.96692	65.43671	9.9834
3	11.813	VB	0.2741	3877.71655	217.35930	36.0464
4	17.109	BBA	0.3878	5018.48145	200.63766	46.6508

Gd(OTf)3 (10 mol%), PyBim-1 (12 mol%), N2, 25 °C, THF, 48 h

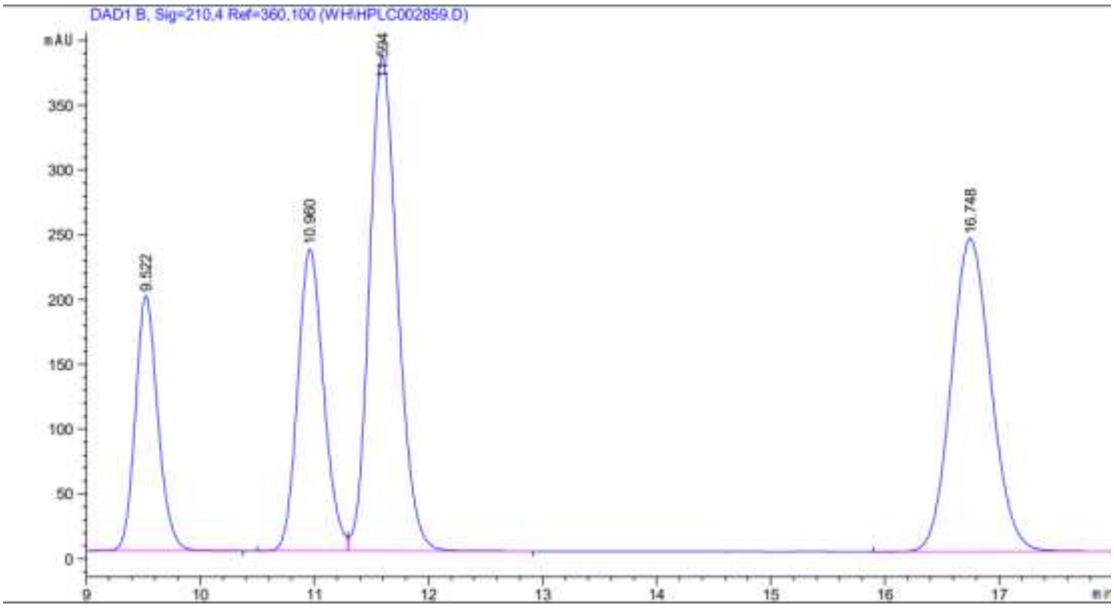

Peak	Ret. Time	Type	Width	Area	Height	Area
1	9.522	BB	0.2098	2695.57886	196.76492	14.3633
2	10.960	BV	0.2418	3670.33618	233.03581	19.5572
3	11.594	VB	0.2665	6558.51563	381.55621	34.9468
4	16.748	BB	0.3763	5842.71729	241.48708	31.1327

Y(OTf)3 (10 mol%), PyBim-1 (12 mol%), N2, 25 °C, THF, 48 h

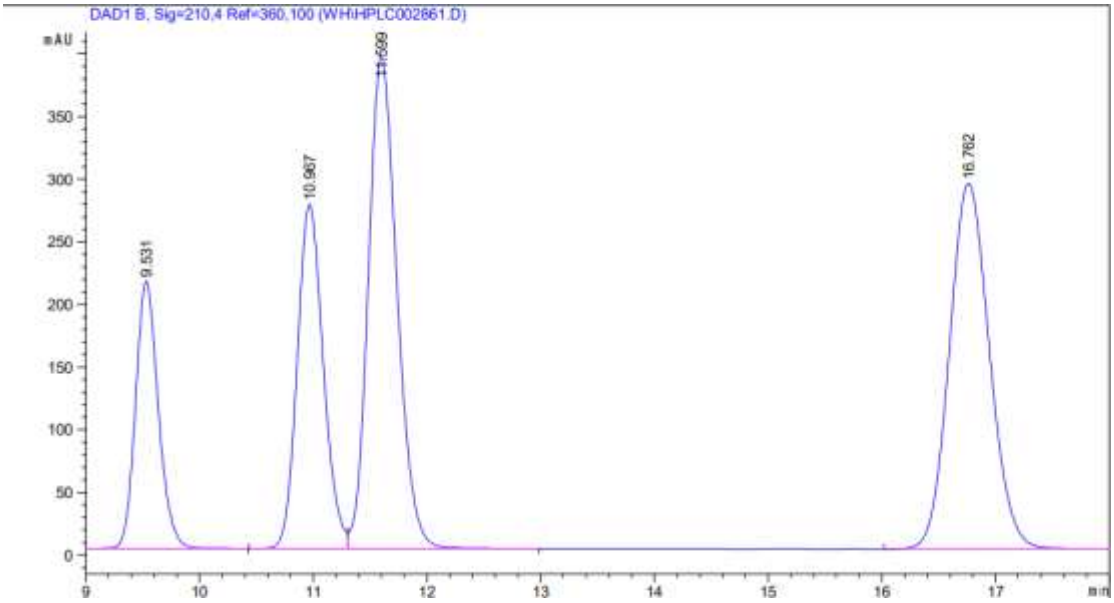

Peak	Ret. Time	Type	Width	Area	Height	Area
1	9.531	BB	0.2143	2966.61182	213.23930	13.9066
2	10.967	BV	0.2457	4375.40527	274.81635	20.5107
3	11.599	VB	0.2698	6864.30518	393.00217	32.1779
4	16.762	BB	0.3788	7126.01025	291.88556	33.4047

Lu(OTf)$_3$ (10 mol%), PyBim-1 (12 mol%), N_2, 25 °C, THF, 48 h

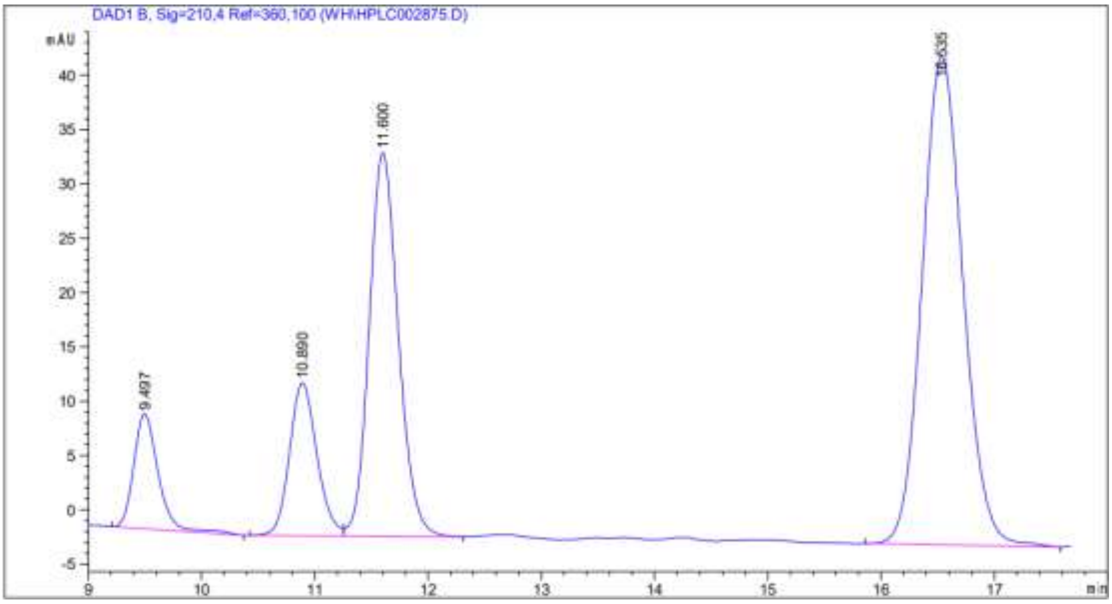

Peak	Ret. Time	Type	Width	Area	Height	Area
1	9.497	BB	0.2296	159.57446	10.59915	7.3974
2	10.890	BV	0.2582	236.92990	14.08504	10.9833
3	11.600	VB	0.2745	631.58966	35.33377	29.2785
4	16.535	BB	0.3888	1129.08582	44.99483	52.3408

Tb(OTf)$_3$ (10 mol%), PyBim-1 (12 mol%), N_2, 25 °C, THF, 48 h

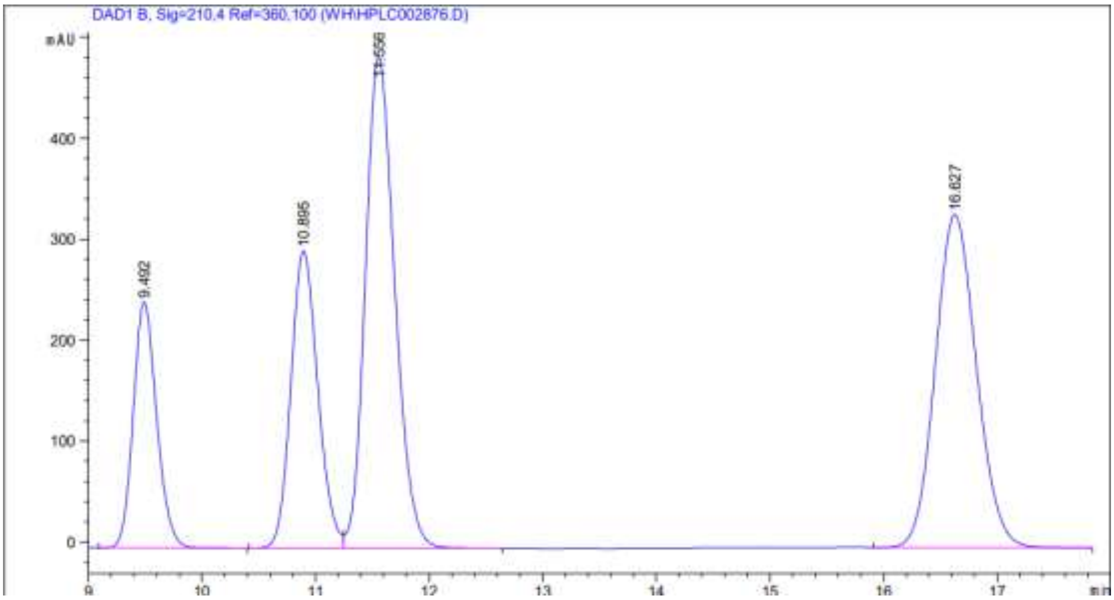

Peak	Ret. Time	Type	Width	Area	Height	Area
1	9.492	BB	0.2215	3498.54468	243.66017	13.7548
2	10.895	BV	0.2545	4854.97412	294.28790	19.0877
3	11.556	VB	0.2781	8777.56641	487.31903	34.5097
4	16.627	BBA	0.3916	8303.99316	329.96619	32.6478

Ho(OTf)$_3$ (10 mol%), PyBim-1 (12 mol%), N_2, 25 °C, THF, 48 h

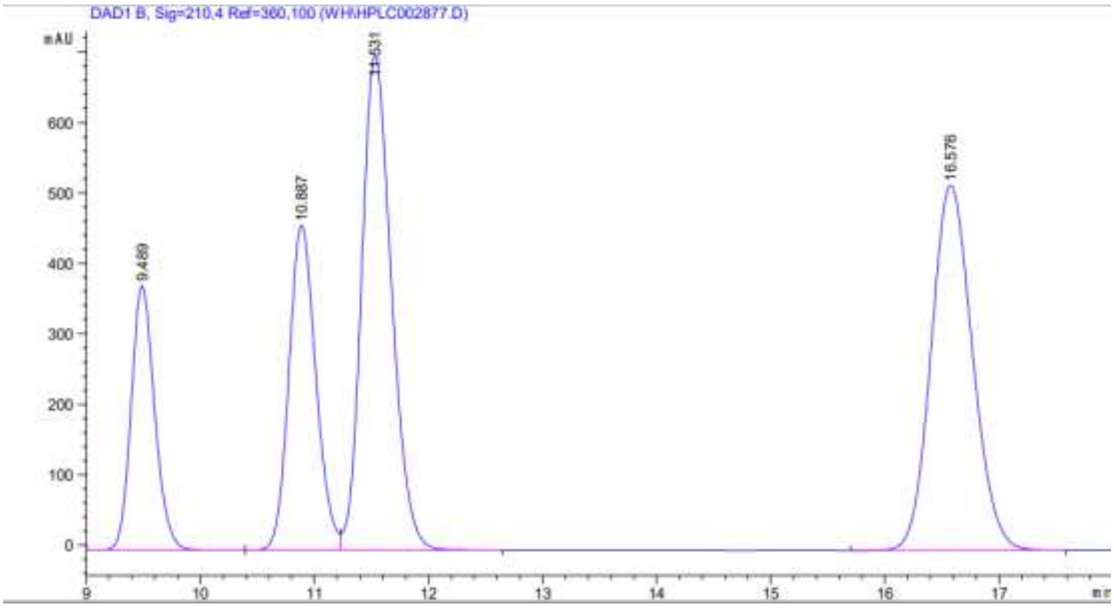

Peak	Ret. Time	Type	Width	Area	Height	Area
1	9.489	BB	0.2208	5367.29590	375.42078	13.8509
2	10.887	BV	0.2534	7574.23877	461.59921	19.5462
3	11.531	VB	0.2820	1.27679e4	702.69275	32.9490
4	16.576	BB	0.3913	1.30410e4	518.79517	33.6538

Er(OTf)$_3$ (10 mol%), PyBim-1 (12 mol%), N_2, 25 °C, THF, 48 h

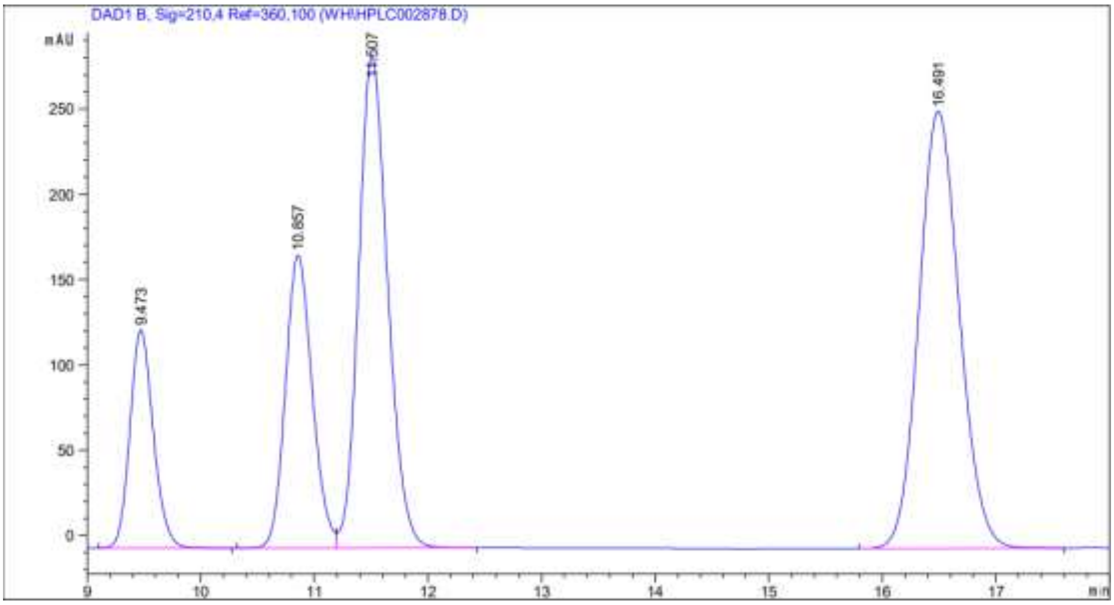

Peak	Ret. Time	Type	Width	Area	Height	Area
1	9.473	BB	0.2186	1823.14856	127.70926	11.3098
2	10.857	BV	0.2534	2814.20923	171.50891	17.4578
3	11.507	VB	0.2741	5135.66992	287.91956	31.8589
4	16.491	BB	0.3850	6347.03223	256.25162	39.3735

La(OTf)$_3$ (10 mol%), PyBim-1 (12 mol%), N_2, 25 °C, Toluene, 48 h

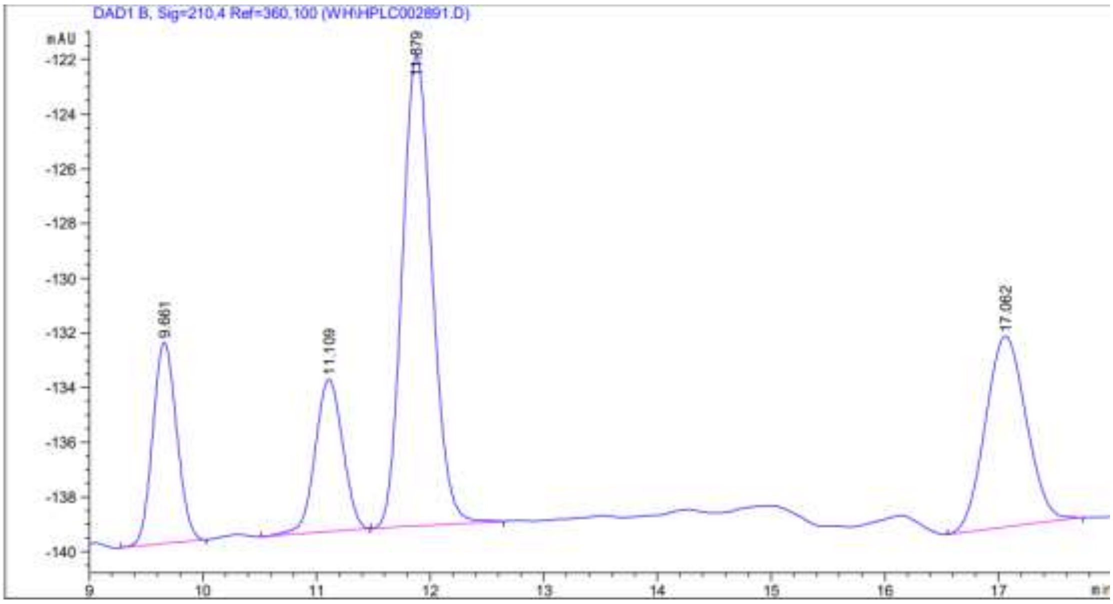

Peak	Ret. Time	Type	Width	Area	Height	Area
1	9.661	BB	0.2284	107.55263	7.36202	15.6140
2	11.109	BB	0.2627	94.05733	5.57941	13.6548
3	11.879	BB	0.2862	313.05081	17.21259	45.4473
4	17.062	BB	0.3890	174.16089	6.98229	25.2839

La(OTf)$_3$ (10 mol%), PyBim-1 (12 mol%), N_2, 25 °C, DCE, 48 h

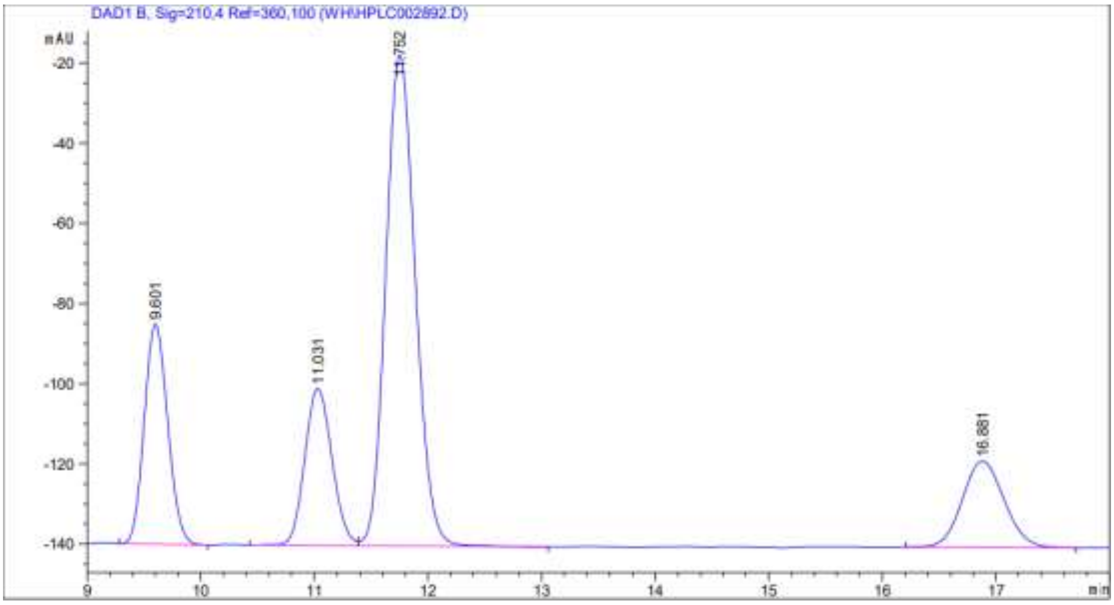

Peak	Ret. Time	Type	Width	Area	Height	Area
1	9.601	BB	0.2257	788.72308	54.86765	18.7422
2	11.031	BV	0.2624	652.31683	39.14171	15.5008
3	11.752	VB	0.2831	2215.28125	122.41315	52.6411
4	16.881	BB	0.3994	551.95575	21.50798	13.1160

La(OTf)$_3$ (10 mol%), PyBim-1 (12 mol%), N$_2$, 25 °C, THF, 48 h

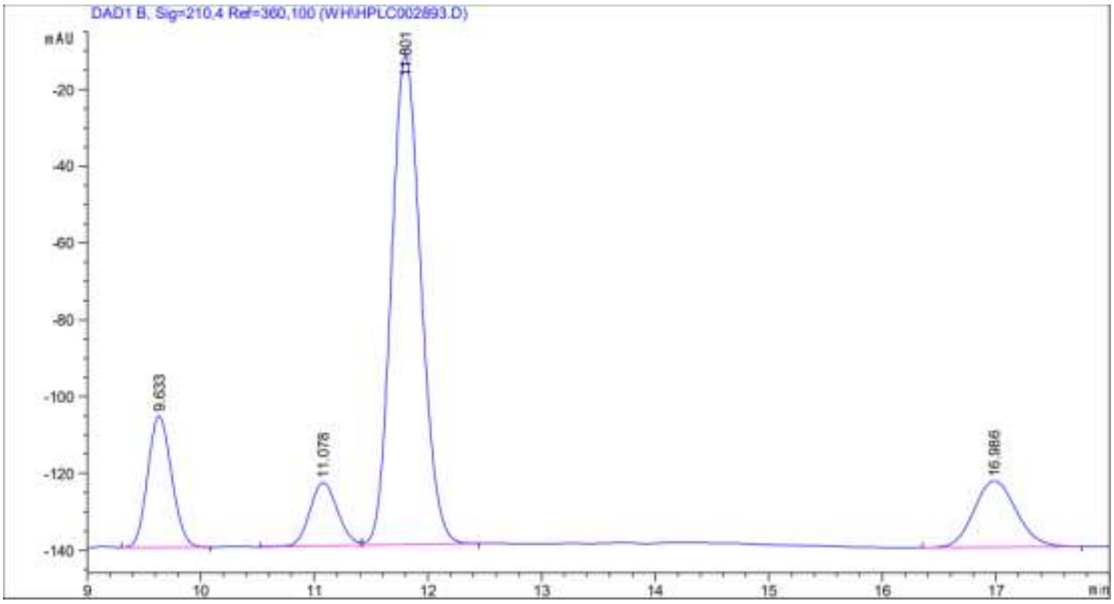

Peak	Ret. Time	Type	Width	Area	Height	Area
1	9.633	BB	0.2285	499.69839	34.19902	14.1551
2	11.078	BV	0.2655	278.55069	16.45621	7.8906
3	11.801	VB	0.2850	2304.09741	127.41353	65.2689
4	16.986	BB	0.4059	447.81210	17.30408	12.6853

$La(OTf)_3$ (10 mol%), PyBim-1 (12 mol%), N_2, 0 °C, THF, 48 h

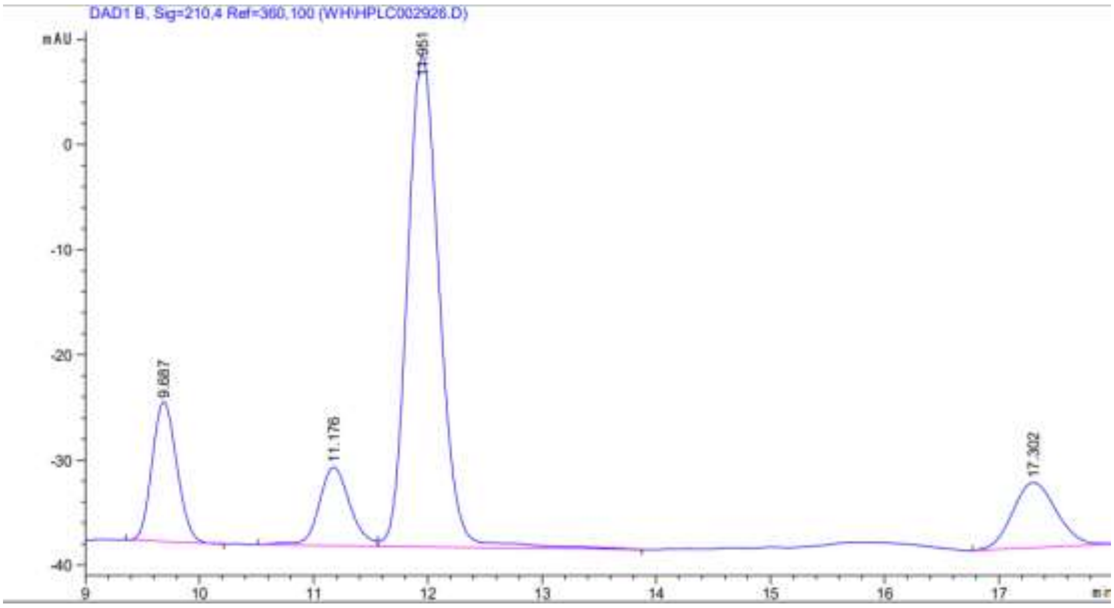

Peak	Ret. Time	Type	Width	Area	Height	Area
1	9.687	BB	0.2373	199.41591	13.26998	14.0900
2	11.176	BV	0.2908	139.70407	7.44806	9.8710
3	11.951	VB	0.3035	911.29480	46.73104	64.3890
4	17.302	BB	0.4130	164.88185	6.18482	11.6500

La(OTf)$_3$ (10 mol%), PyBim-1 (12 mol%), N_2, 25 °C, MeCN, 48 h

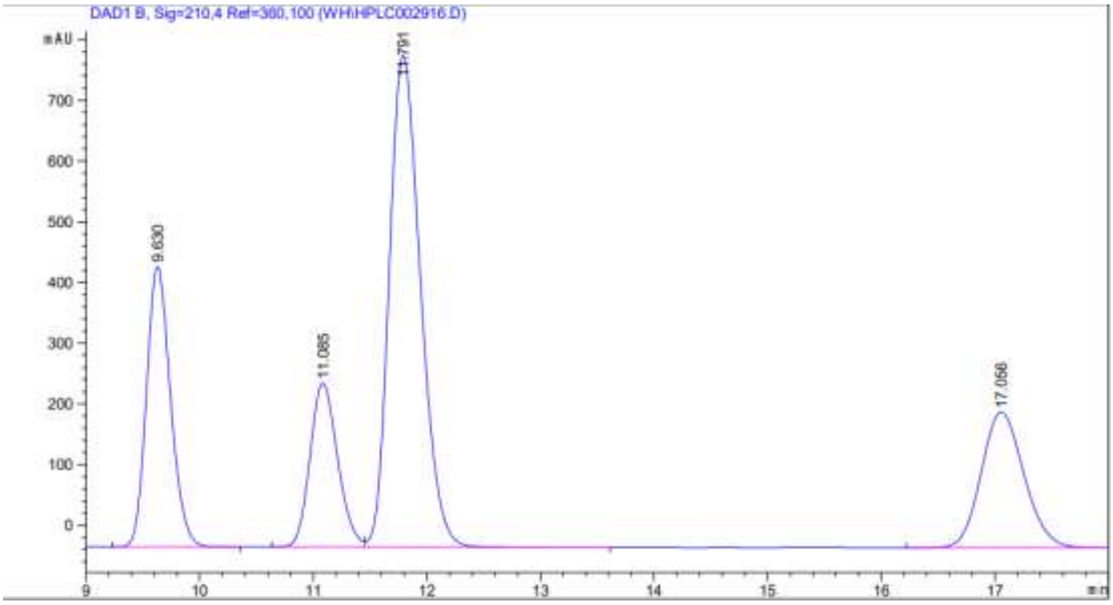

Peak	Ret. Time	Type	Width	Area	Height	Area
1	9.630	BB	0.2270	6770.30664	461.90839	20.7506
2	11.085	BV	0.2607	4549.54541	269.80151	13.9441
3	11.791	VB	0.2965	1.54531e4	810.29358	47.3629
4	17.056	BB	0.4055	5854.03223	223.55402	17.9423

La(OTf)$_3$ (10 mol%), PyBim-1 (12 mol%), N_2, 25 °C, EA, 48 h

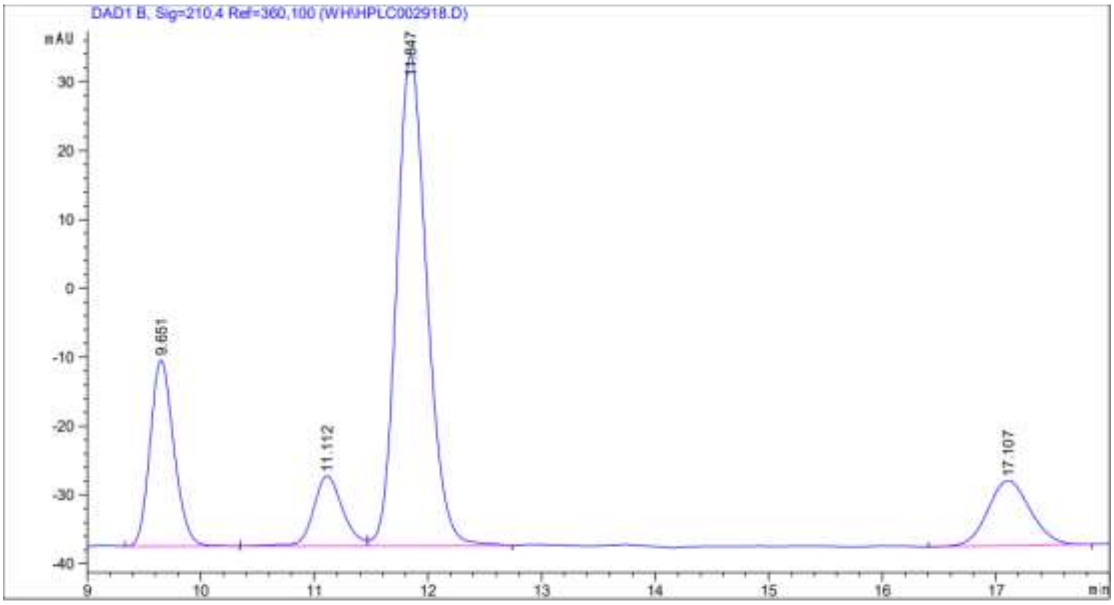

Peak	Ret. Time	Type	Width	Area	Height	Area
1	9.651	BB	0.2256	393.10529	27.04043	18.3859
2	11.112	BV	0.2712	180.58505	10.16800	8.4461
3	11.847	VB	0.2860	1318.48889	71.21951	61.6668
4	17.107	BB	0.4019	245.90456	9.43891	11.5012

La(OTf)$_3$ (10 mol%), PyBim-1 (12 mol%), N$_2$, 25 °C, Et$_2$O, 48 h

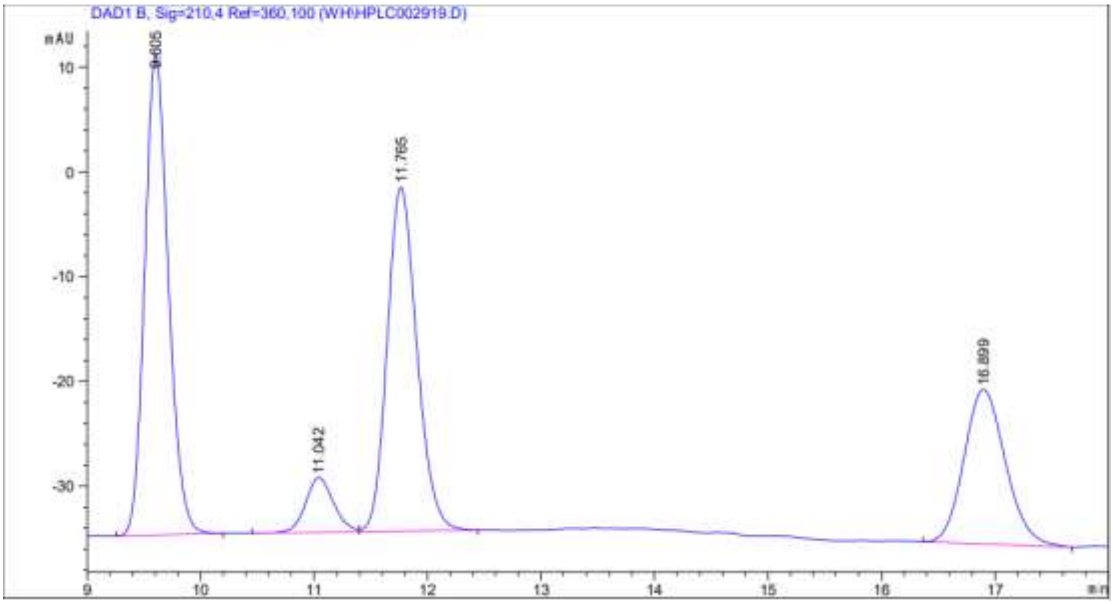

Peak	Ret. Time	Type	Width	Area	Height	Area
1	9.605	BB	0.2233	664.27942	45.78436	38.5622
2	11.042	BV	0.2635	88.80546	5.19438	5.1553
3	11.765	VB	0.2802	596.69739	32.80596	34.6390
4	16.899	BB	0.3895	372.83530	14.71765	21.6435

La(OTf)$_3$ (10 mol%), PyBim-1 (12 mol%), N_2, 25 °C, $CHCl_3$, 48 h

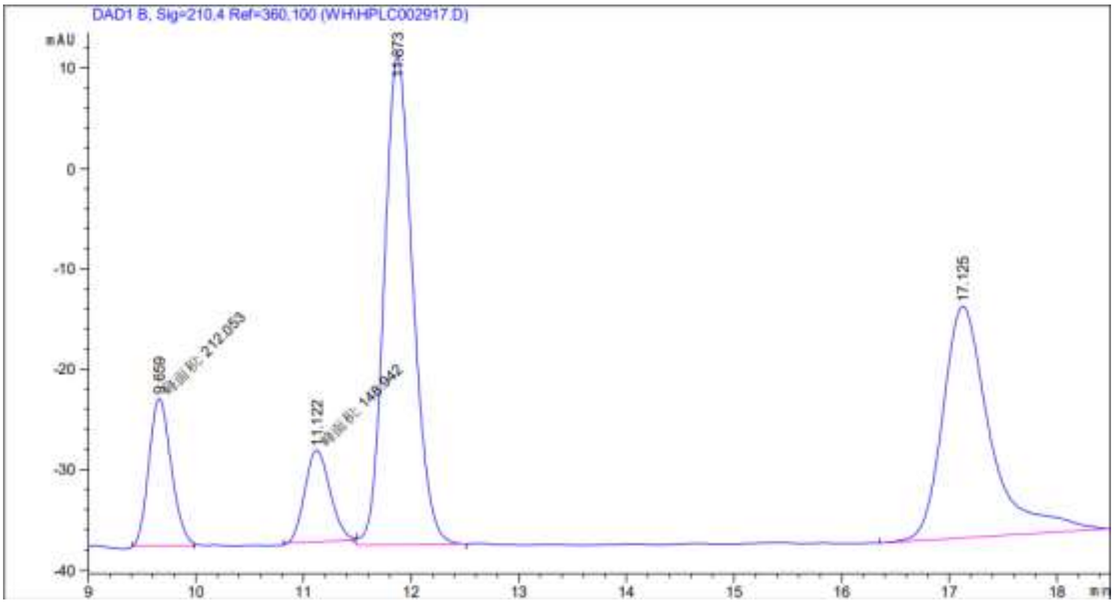

Peak	Ret. Time	Type	Width	Area	Height	Area
1	9.659	MM	0.2403	212.05312	14.70938	10.7933
2	11.122	MM	0.2712	148.94235	9.15177	7.5810
3	11.873	VB	0.2857	899.21722	48.62716	45.7692
4	17.125	BBA	0.4578	704.46594	23.09824	35.8565

La(OTf)$_3$ (10 mol%), PyBim-1 (12 mol%), N_2, 25 °C, CH_3COCH_3, 48 h

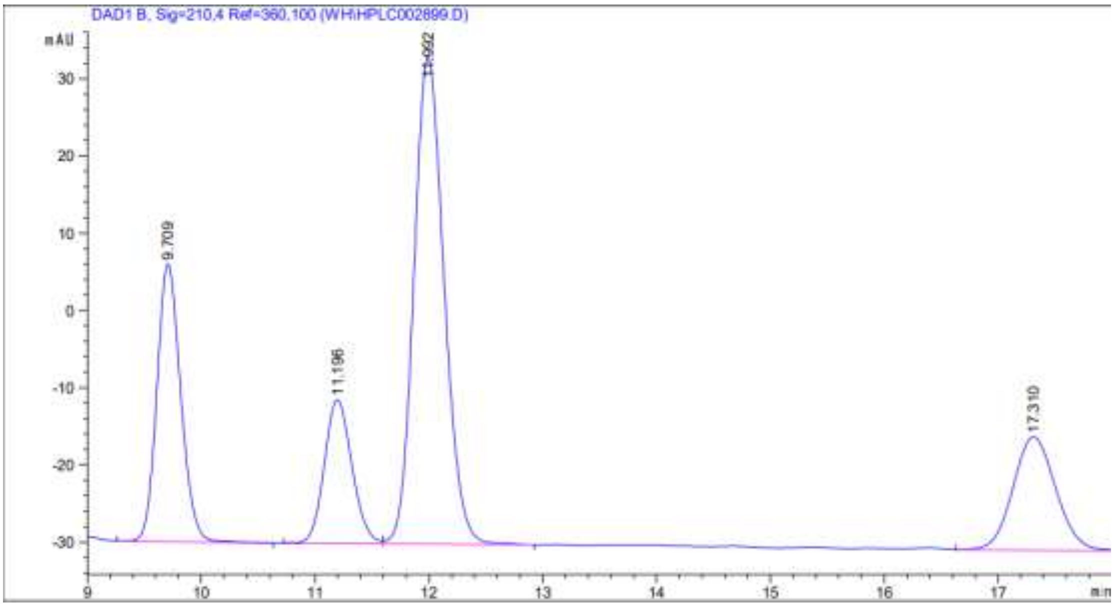

Peak	Ret. Time	Type	Width	Area	Height	Area
1	9.709	BB	0.2257	522.99115	35.94527	21.9663
2	11.196	BV	0.2605	313.31125	18.59886	13.1595
3	11.992	VB	0.2845	1160.02625	63.09098	48.7225
4	17.310	BB	0.4079	384.55347	14.66761	16.1517

Optimization of PyBox-1

La(OTf)$_3$ (10 mol%), PyBox-1 (12 mol%), N_2, 25 °C, THF, 48 h

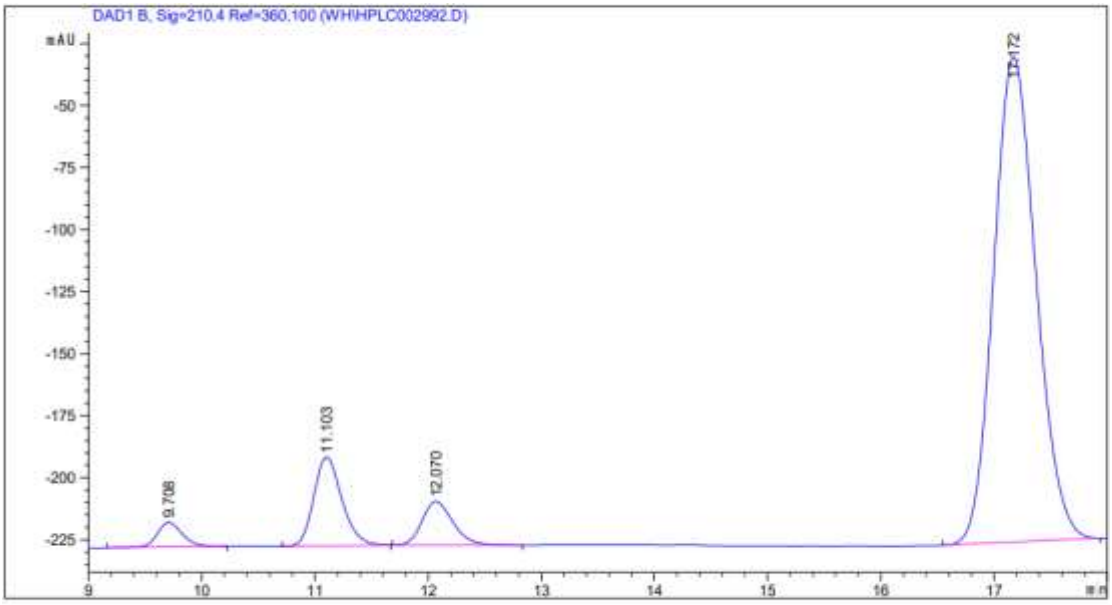

Peak	Ret. Time	Type	Width	Area	Height	Area
1	9.708	BB	0.2344	151.78452	9.92730	2.4584
2	11.103	BB	0.2641	612.79163	35.72356	9.9251
3	12.070	BB	0.2888	328.13419	17.49356	5.3146
4	17.172	BB	0.4035	5081.45313	195.32072	82.3019

Pr(OTf)$_3$ (10 mol%), PyBox-1 (12 mol%), N_2, 25 °C, THF, 48 h

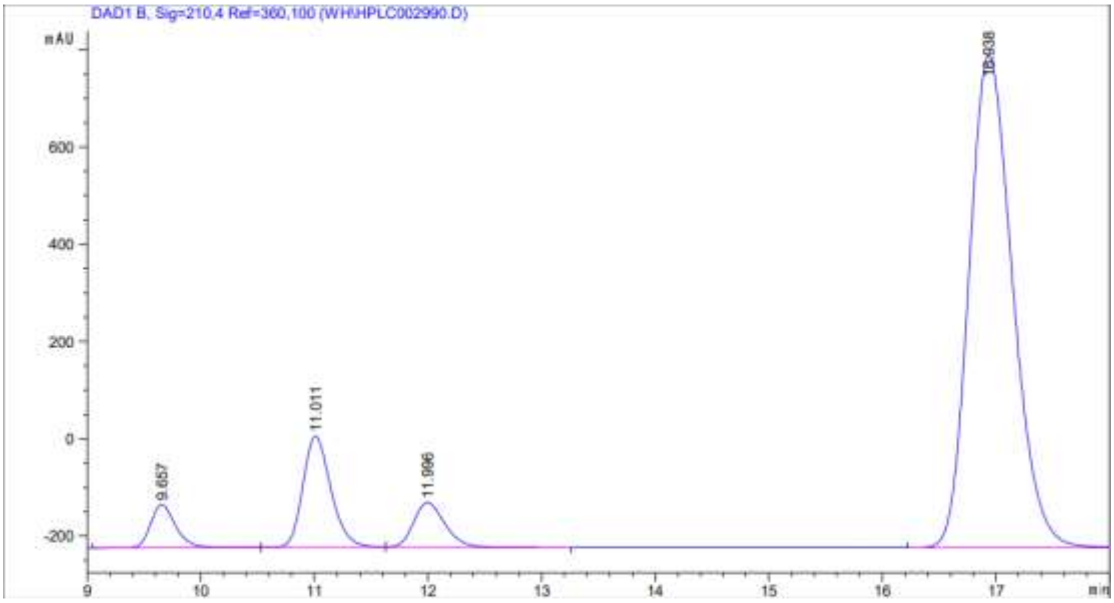

Peak	Ret. Time	Type	Width	Area	Height	Area
1	9.657	BB	0.2318	1350.48340	88.59172	3.9582
2	11.011	BV	0.2644	3924.60596	228.50079	11.5028
3	11.996	VB	0.2974	1781.58459	92.23235	5.2217
4	16.938	BBA	0.4158	2.70622e4	1012.50031	79.3174

Ce(OTf)$_3$ (10 mol%), PyBox-1 (12 mol%), N$_2$, 25 °C, THF, 48 h

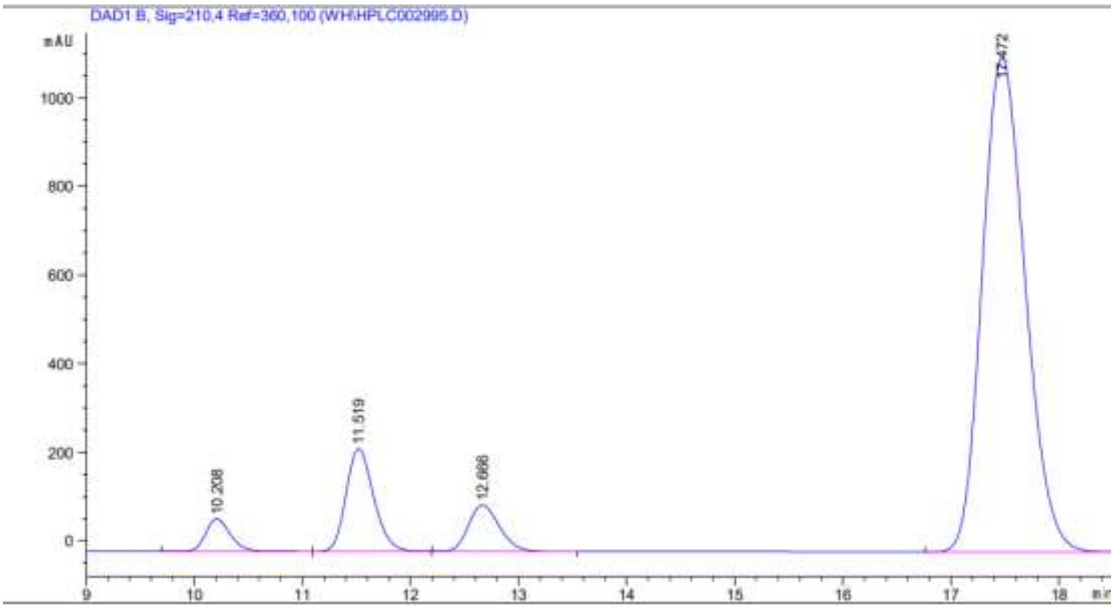

Peak	Ret. Time	Type	Width	Area	Height	Area
1	10.208	BB	0.2422	1157.69141	73.33977	3.0358
2	11.519	BB	0.2749	4104.03320	231.40385	10.7618
3	12.666	BB	0.3106	2078.68115	104.24421	5.4508
4	17.472	BB	0.4343	3.07948e4	1114.71375	80.7516

Sm(OTf)$_3$ (10 mol%), PyBox-1 (12 mol%), N_2, 25 °C, THF, 48 h

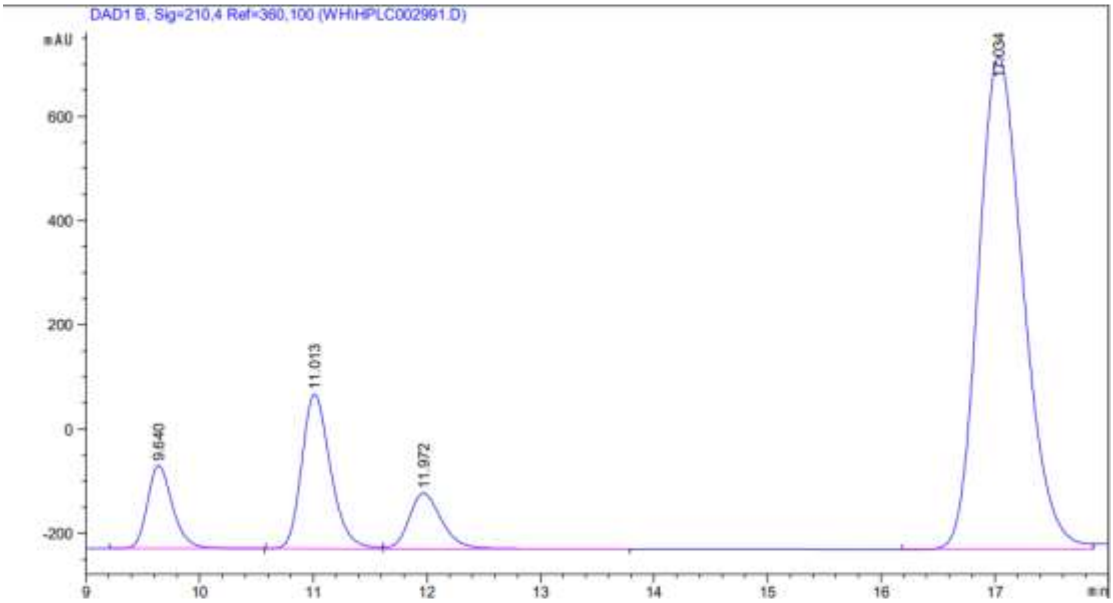

Peak	Ret. Time	Type	Width	Area	Height	Area
1	9.640	BB	0.2352	2444.97119	159.19646	6.8427
2	11.013	BV	0.2697	5220.72705	296.15381	14.6112
3	11.972	VB	0.3039	2113.21899	106.31782	5.9142
4	17.034	BV	0.4263	2.59522e4	945.27118	72.6319

Sc(OTf)$_3$ (10 mol%), PyBox-1 (12 mol%), N_2, 25 °C, THF, 48 h

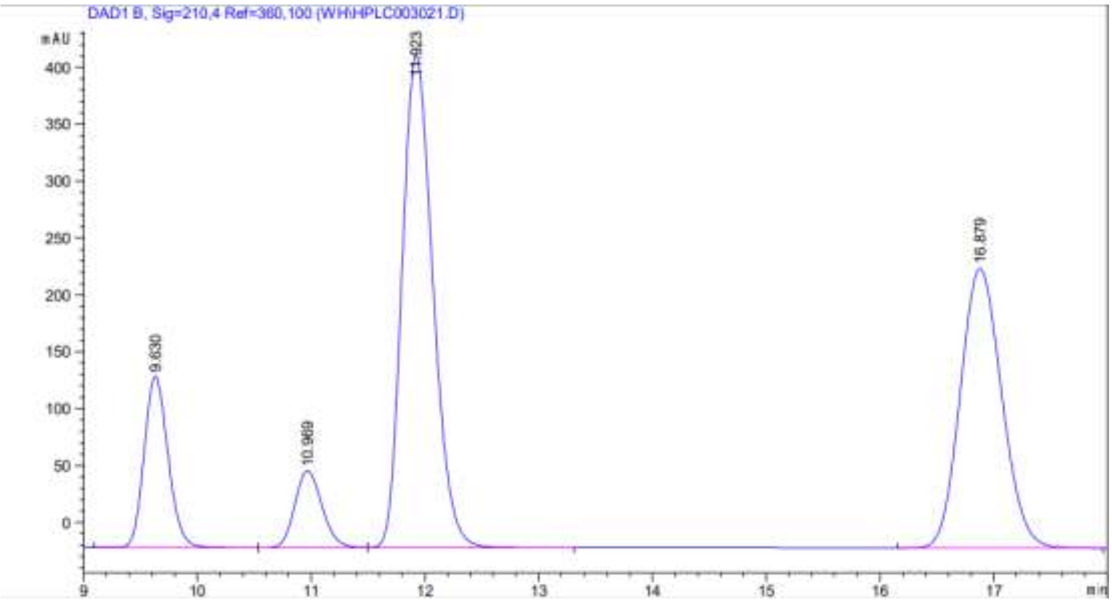

Peak	Ret. Time	Type	Width	Area	Height	Area
1	9.630	BB	0.2269	2200.72900	150.24831	12.4350
2	10.969	BV	0.2610	1128.55298	67.53554	6.3768
3	11.923	VB	0.2935	8147.88672	433.05783	46.0387
4	16.879	BB	0.3954	6220.73242	245.69885	35.1495

Yb(OTf)$_3$ (10 mol%), PyBox-1 (12 mol%), N_2, 25 °C, THF, 48 h

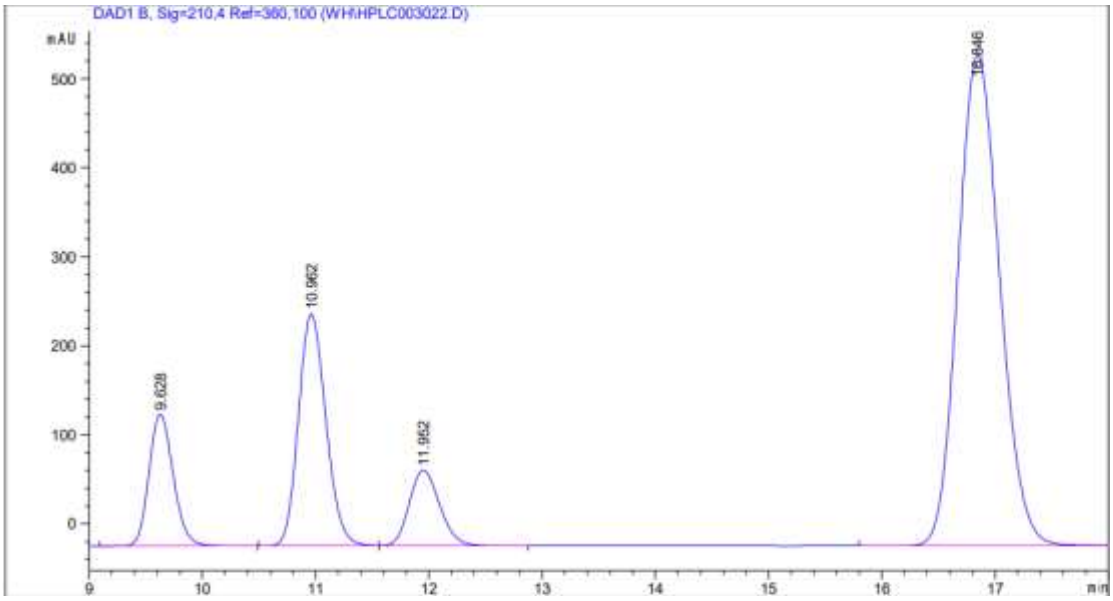

Peak	Ret. Time	Type	Width	Area	Height	Area
1	9.628	BB	0.2312	2199.16333	148.10686	9.8622
2	10.962	BV	0.2625	4382.52002	260.26407	19.6534
3	11.952	VB	0.2919	1582.19006	84.70247	7.0953
4	16.846	BBA	0.4034	1.41351e4	550.80951	63.3891

$Y(OTf)_3$ (10 mol%), PyBox-1 (12 mol%), N_2, 25 °C, THF, 48 h

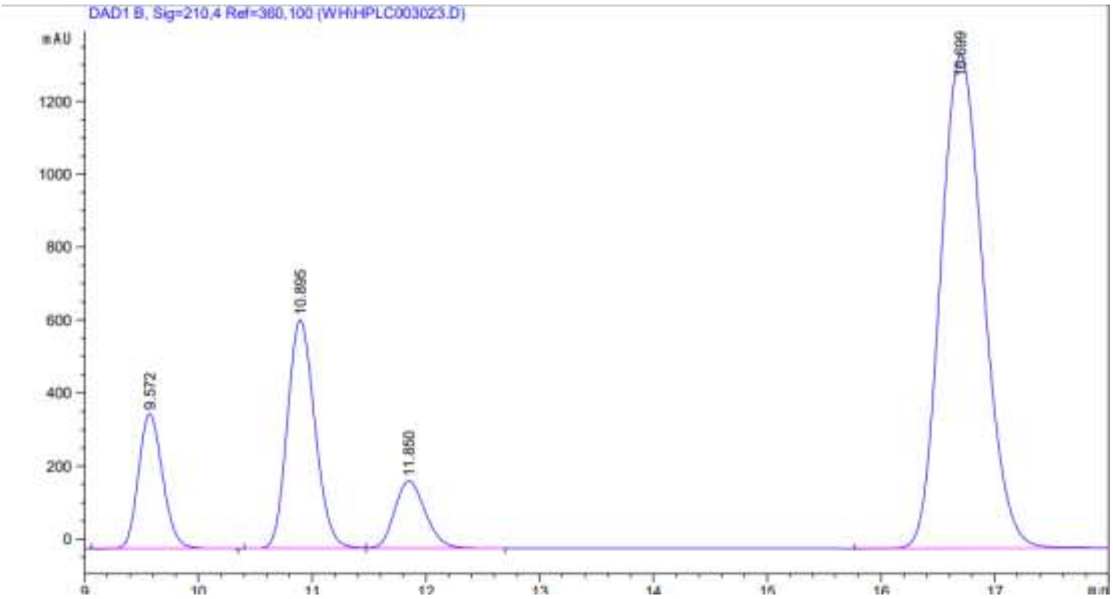

Peak	Ret. Time	Type	Width	Area	Height	Area
1	9.572	BB	0.2305	5466.66846	369.70657	9.9886
2	10.895	BV	0.2626	1.05565e4	626.46082	19.2887
3	11.850	VB	0.2933	3475.00708	184.84813	6.3495
4	16.699	BB	0.4099	3.52308e4	1352.49878	64.3732

Lu(OTf)$_3$ (10 mol%), PyBox-1 (12 mol%), N_2, 25 °C, THF, 48 h

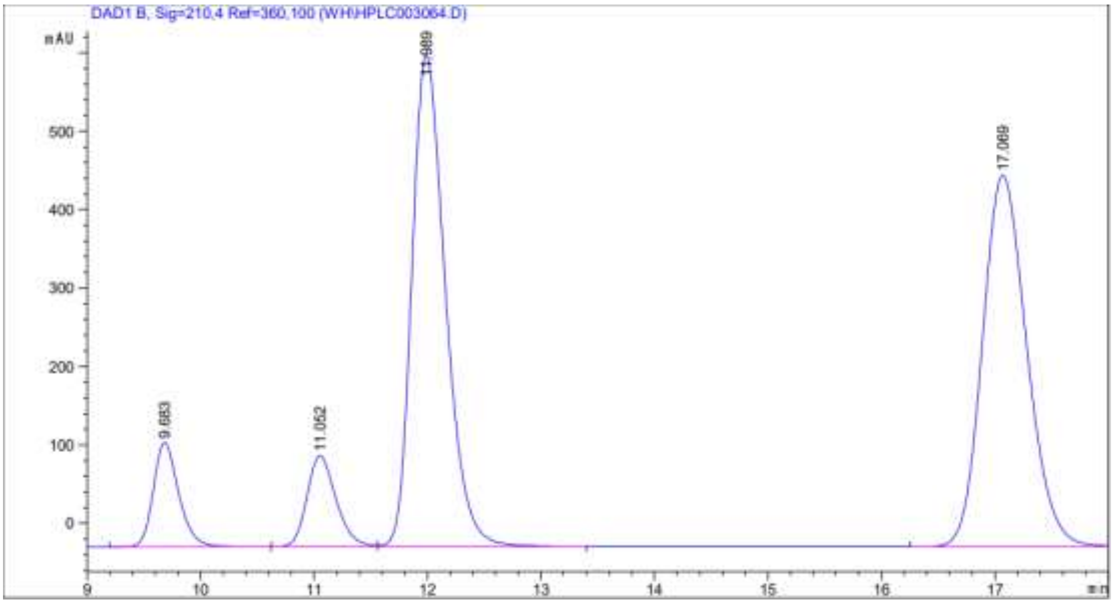

Peak	Ret. Time	Type	Width	Area	Height	Area
1	9.683	BB	0.2369	2060.03564	132.82394	6.9061
2	11.052	BV	0.2711	2055.23462	115.80403	6.8900
3	11.989	VB	0.3171	1.28466e4	626.73065	43.0670
4	17.069	BB	0.4211	1.28674e4	473.37277	43.1369

Tb(OTf)3 (10 mol%), PyBox-1 (12 mol%), N2, 25 °C, THF, 48 h

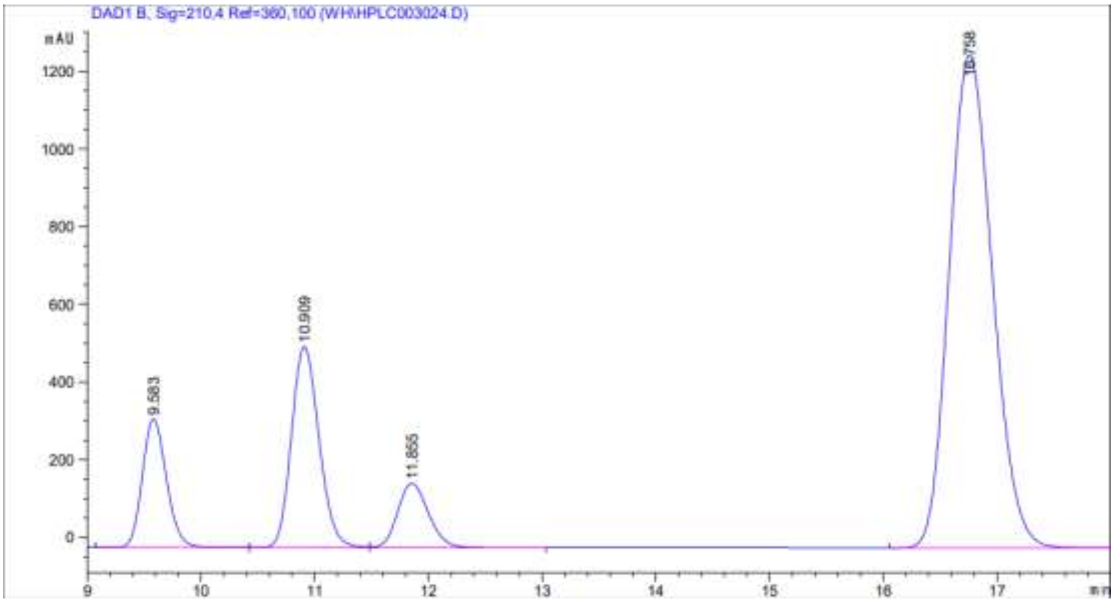

Peak	Ret. Time	Type	Width	Area	Height	Area
1	9.583	BB	0.2299	4862.71875	330.08298	9.6964
2	10.909	BV	0.2624	8696.91406	516.65948	17.3419
3	11.855	VB	0.2937	3098.35278	164.52449	6.1782
4	16.758	BB	0.4160	3.34917e4	1268.63208	66.7835

Ho(OTf)3 (10 mol%), PyBox-1 (12 mol%), N2, 25 °C, THF, 48 h

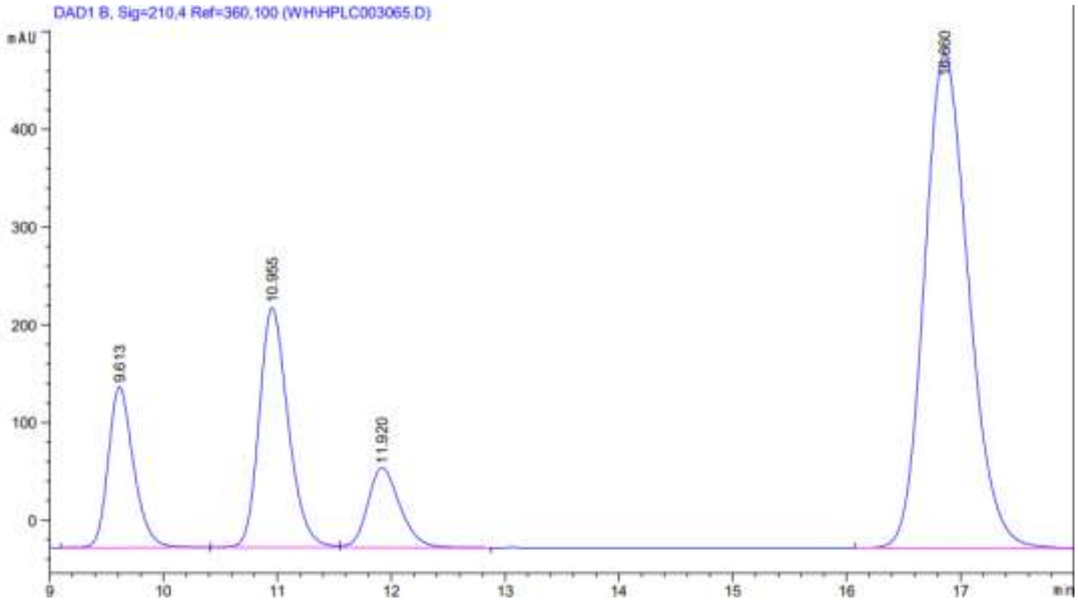

Peak	Ret. Time	Type	Width	Area	Height	Area
1	9.613	BB	0.2339	2532.54736	164.20514	11.4340
2	10.955	BV	0.2724	4341.19873	245.36513	19.5997
3	11.920	VB	0.3033	1603.68115	81.60186	7.2403
4	16.860	BB	0.4196	1.36718e4	505.45746	61.7259

Er(OTf)$_3$ (10 mol%), PyBox-1 (12 mol%), N_2, 25 °C, THF, 48 h

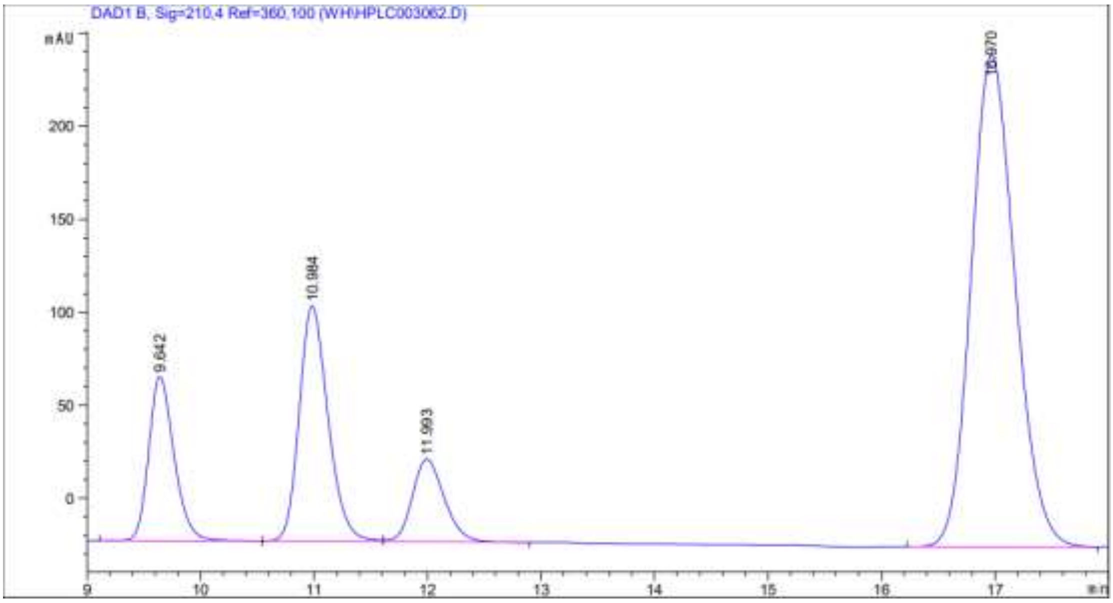

Peak	Ret. Time	Type	Width	Area	Height	Area
1	9.642	BB	0.2370	1369.85486	88.31416	11.8299
2	10.984	BV	0.2701	2234.32617	126.45522	19.2953
3	11.993	VB	0.3025	868.52197	44.33752	7.5004
4	16.970	BB	0.4177	7106.93066	264.29865	61.3744

Tm(OTf)$_3$ (10 mol%), PyBox-1 (12 mol%), N_2, 25 °C, THF, 48 h

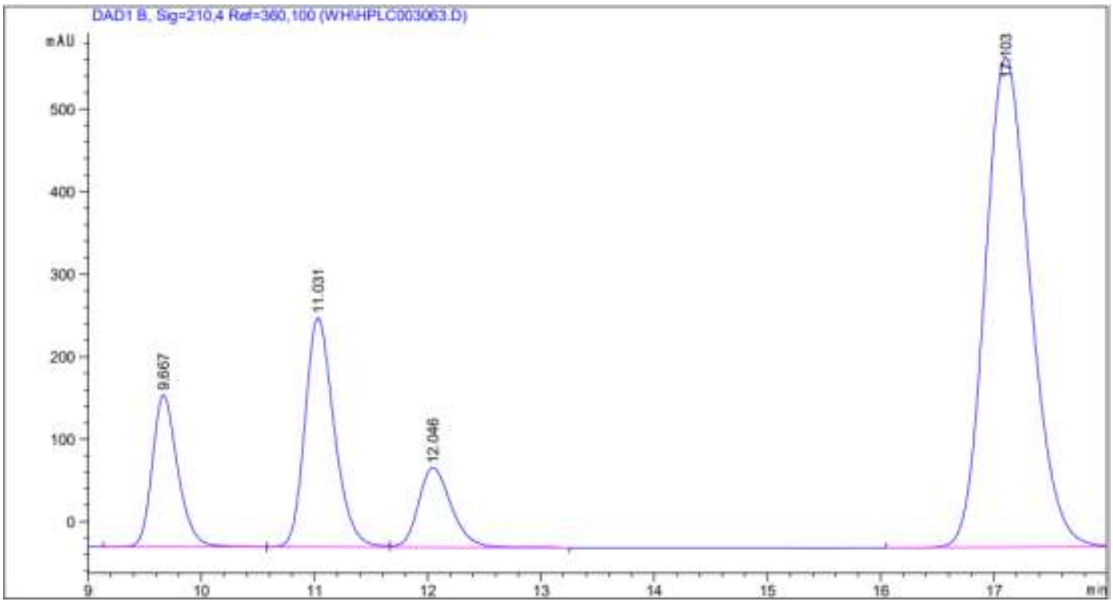

Peak	Ret. Time	Type	Width	Area	Height	Area
1	9.667	BB	0.2376	2863.35181	183.96692	10.9456
2	11.031	BV	0.2722	4956.33105	277.77377	18.9464
3	12.046	VB	0.3079	1934.12170	96.46944	7.3935
4	17.103	BB	0.4301	1.64059e4	594.17285	62.7144

Eu(OTf)$_3$ (10 mol%), PyBox-1 (12 mol%), N_2, 25 °C, THF, 48 h

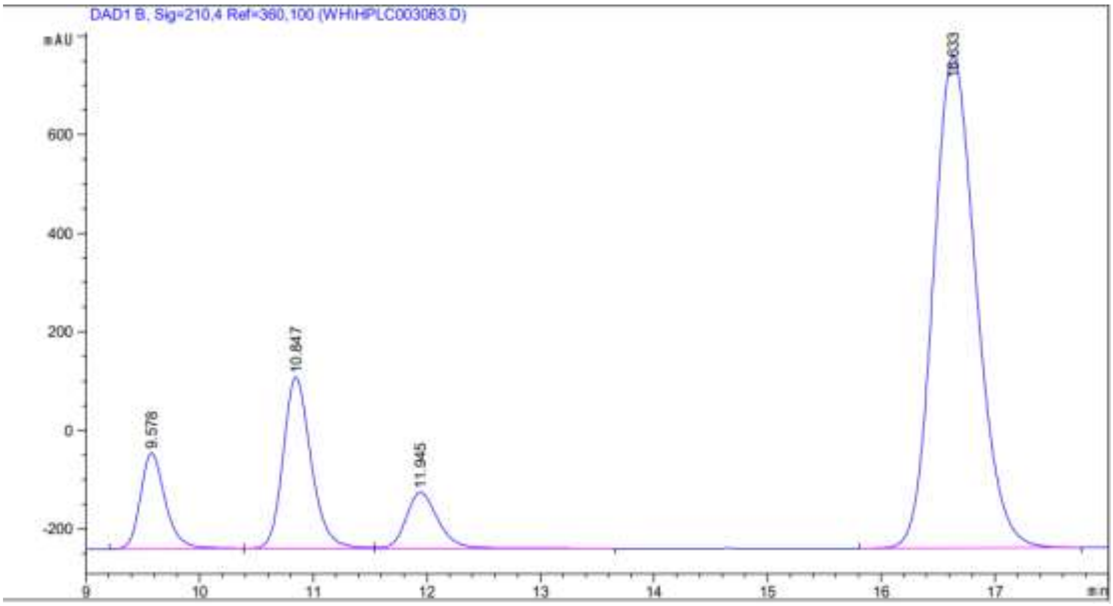

Peak	Ret. Time	Type	Width	Area	Height	Area
1	9.578	BB	0.2306	2939.13330	194.14799	7.8911
2	10.847	BV	0.2614	5935.17383	347.23926	15.9349
3	11.945	VB	0.3009	2223.47925	113.30730	5.9697
4	16.633	BB	0.4079	2.61486e4	997.32831	70.2044

Gd(OTf)₃ (10 mol%), PyBox-1 (12 mol%), N₂, 25 °C, THF, 48 h

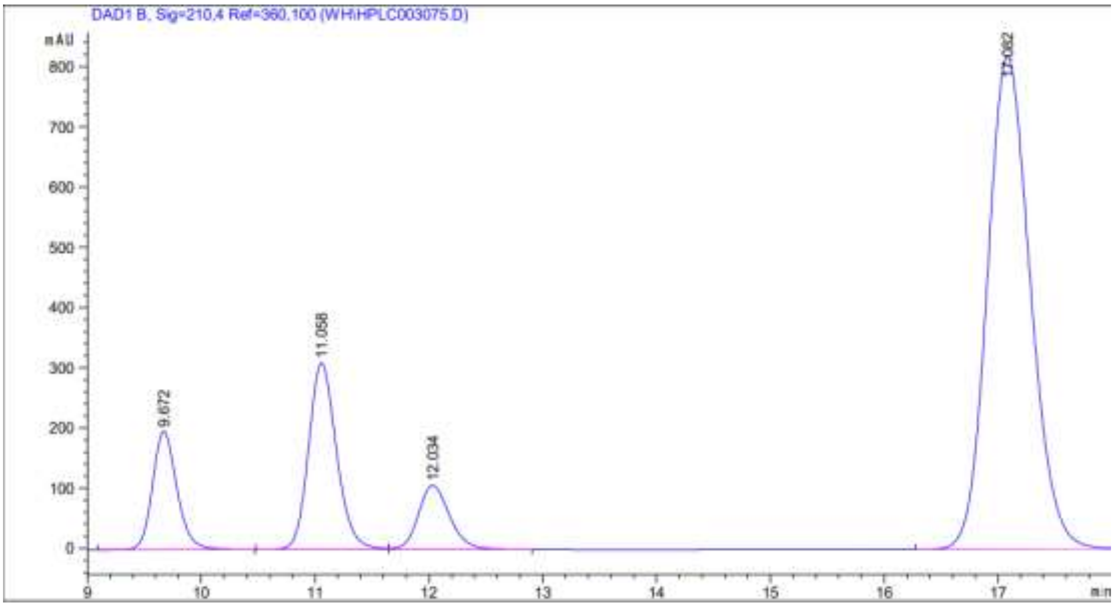

Peak	Ret. Time	Type	Width	Area	Height	Area
1	9.672	BB	0.2294	2914.40259	196.03452	9.2098
2	11.058	BV	0.2626	5272.02051	309.76868	16.6602
3	12.034	VB	0.2920	2025.13464	106.40716	6.3996
4	17.082	BB	0.4077	2.14329e4	818.05536	67.7304

La(OTf)$_3$ (10 mol%), PyBox-1 (12 mol%), N$_2$, 25 °C, THF (4 mL), 48 h

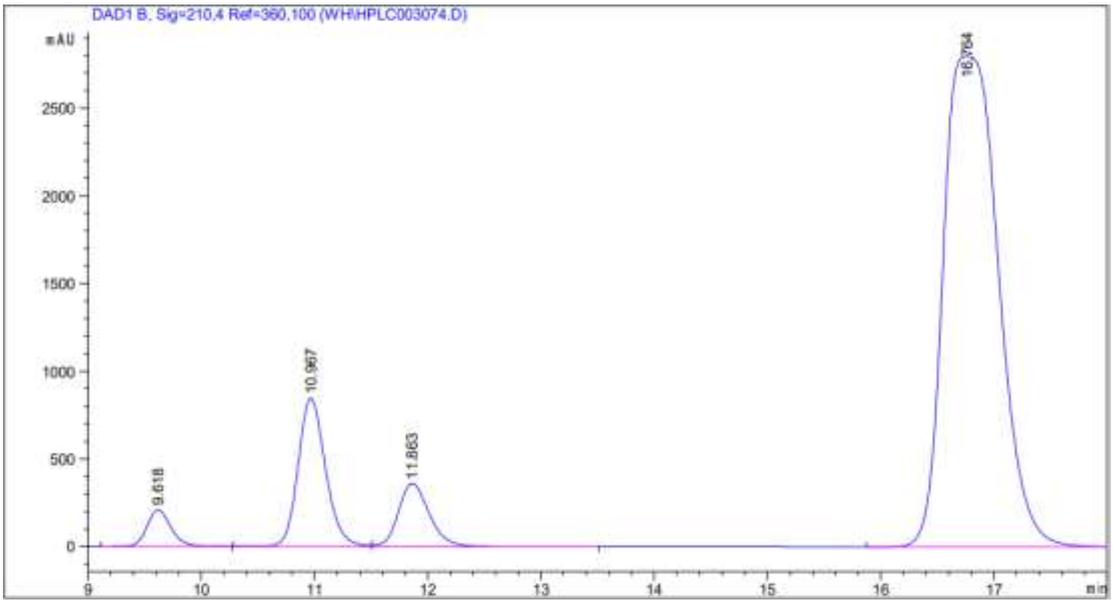

Peak	Ret. Time	Type	Width	Area	Height	Area
1	9.618	BB	0.2245	3031.96094	207.45012	2.5699
2	10.967	BV	0.2580	1.41925e4	844.66736	12.0297
3	11.863	VB	0.2924	6780.96973	358.94214	5.7476
4	16.764	BB	0.4761	9.39735e4	2795.92627	79.6528

La(OTf)$_3$ (10 mol%), PyBox-1 (12 mol%), N$_2$, 25 °C, Et$_2$O , 48 h

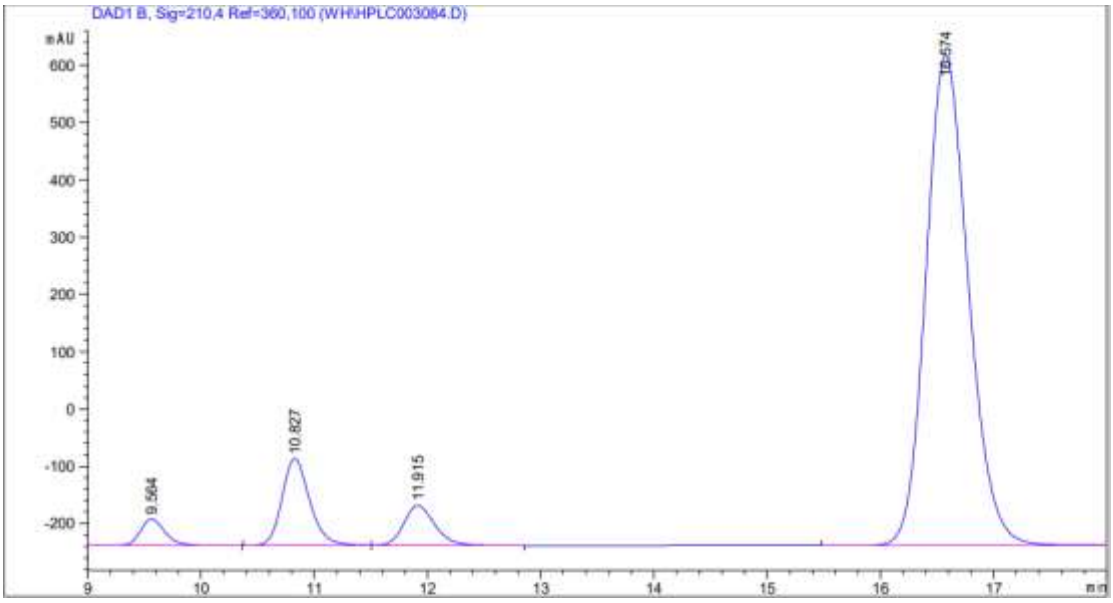

Peak	Ret. Time	Type	Width	Area	Height	Area
1	9.564	BB	0.2296	701.63226	46.60556	2.6065
2	10.827	BV	0.2606	2576.03540	151.33366	9.5696
3	11.915	VB	0.2947	1340.30725	69.60465	4.9791
4	16.574	BB	0.4062	2.23009e4	855.39014	82.8449

La(OTf)$_3$ (10 mol%), PyBox-1 (12 mol%), N_2, 25 °C, DCE:THF=1:1 , 48 h

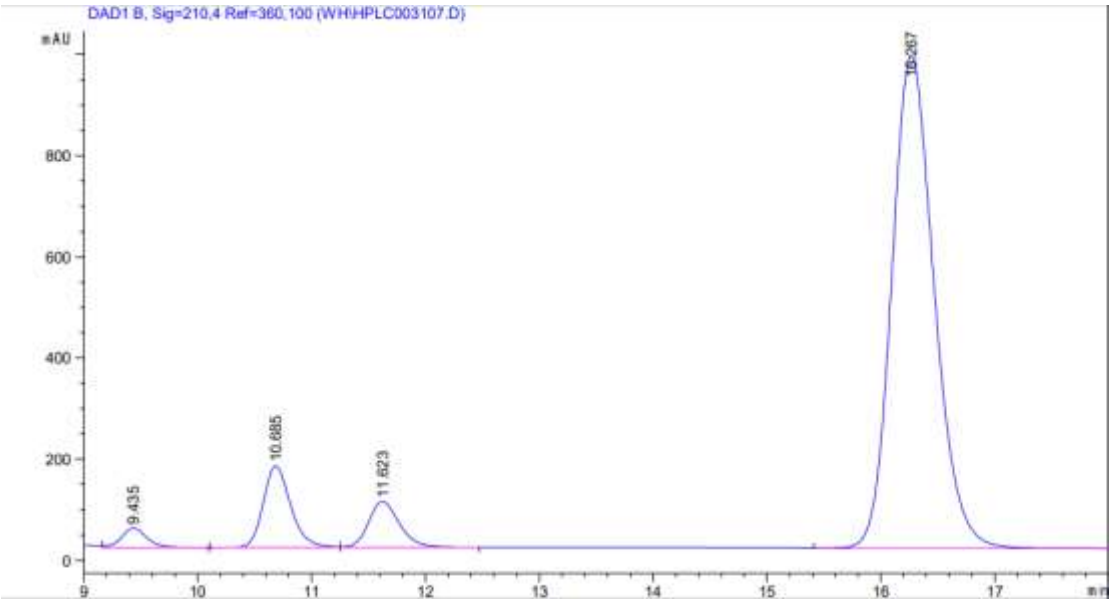

Peak	Ret. Time	Type	Width	Area	Height	Area
1	9.435	VB	0.2373	615.10461	39.14191	2.0363
2	10.685	BV	0.2556	2698.86060	160.95085	8.9347
3	11.623	VB	0.2894	1708.29468	90.83689	5.6554
4	16.267	BBA	0.3996	2.51841e4	974.30316	83.3735

La(OTf)$_3$ (10 mol%), PyBox-1 (12 mol%), N$_2$, 25 °C, DCE:THF=1:3 , 48 h

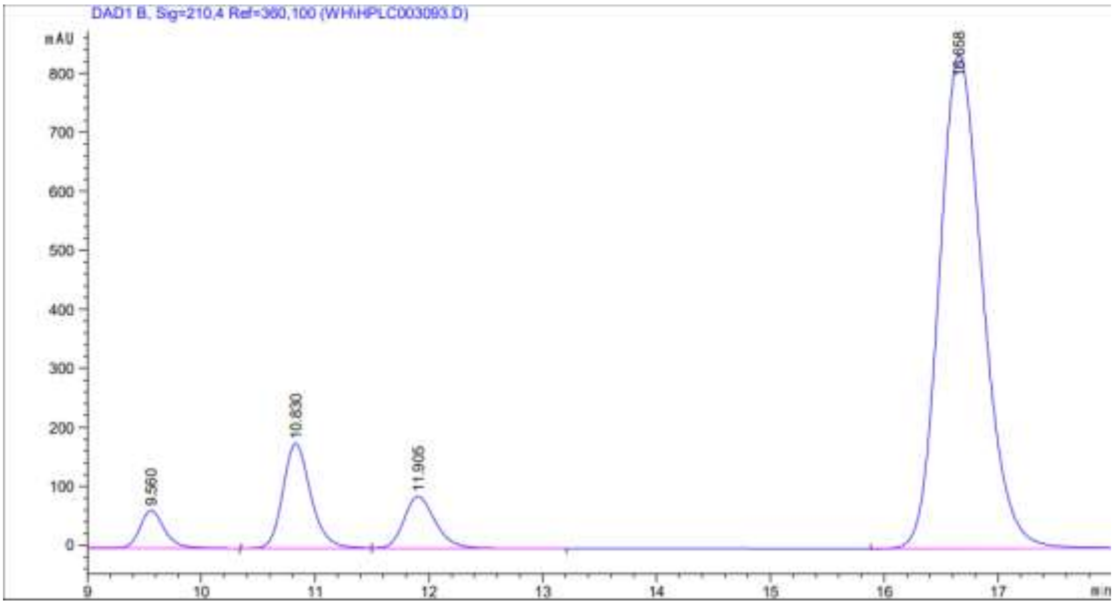

Peak	Ret. Time	Type	Width	Area	Height	Area
1	9.560	BB	0.2293	958.15247	63.75191	3.4444
2	10.830	BV	0.2607	3024.68750	177.56520	10.8733
3	11.905	VB	0.2963	1691.88464	87.99960	6.0821
4	16.658	BB	0.4092	2.21428e4	835.62244	79.6002

La(OTf)$_3$ (10 mol%), PyBox-1 (12 mol%), N_2, 25 °C, CH_3COCH_3:THF=1:1 , 48 h

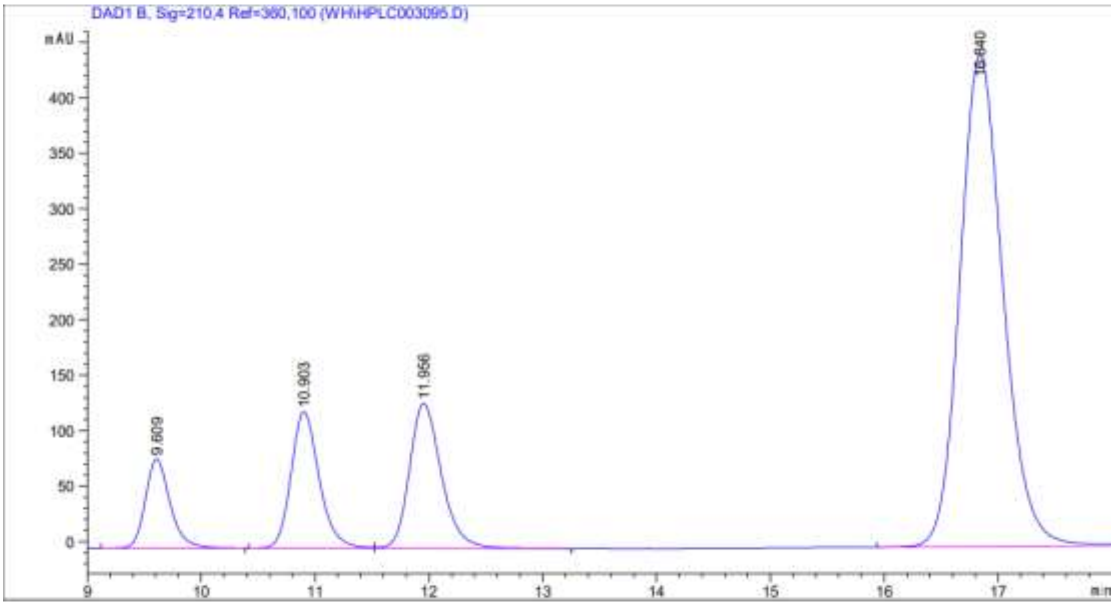

Peak	Ret. Time	Type	Width	Area	Height	Area
1	9.609	BB	0.2304	1216.32068	80.43851	6.9196
2	10.903	BV	0.2628	2115.34741	122.93173	12.0342
3	11.956	VB	0.2976	2549.30591	130.68333	14.5030
4	16.840	BB	0.4063	1.16968e4	442.68738	66.5432

La(OTf)$_3$ (10 mol%), PyBox-1 (12 mol%), N_2, 25 °C, MeCN:THF=1:1 , 48 h

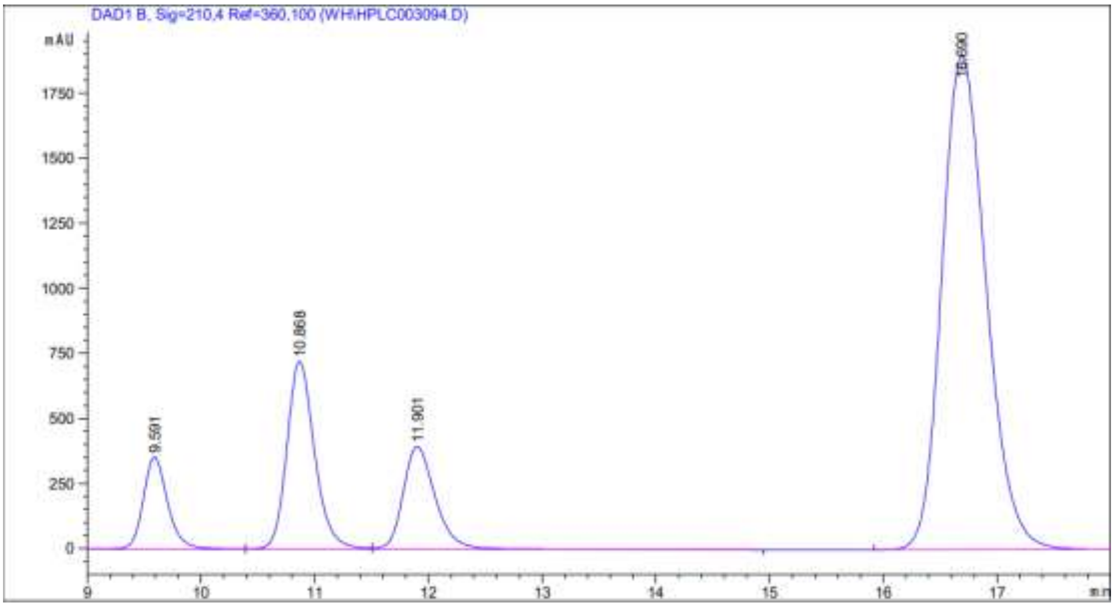

Peak	Ret. Time	Type	Width	Area	Height	Area
1	9.591	BV	0.2276	5326.04883	353.88031	6.9366
2	10.868	VV	0.2601	1.22621e4	722.11218	15.9700
3	11.901	VB	0.3035	7825.35303	394.40002	10.1917
4	16.690	BB	0.4224	5.13685e4	1894.33264	66.9017

La(OTf)$_3$ (10 mol%), PyBox-1 (12 mol%), N_2, 25 °C, Et_2O:THF=1:1 , 48 h

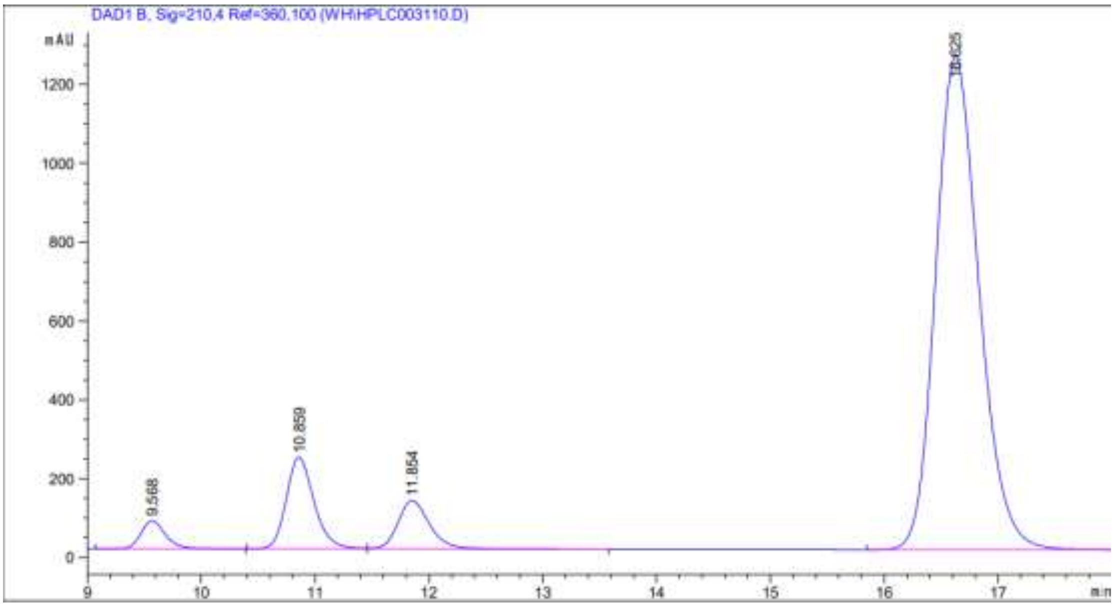

Peak	Ret. Time	Type	Width	Area	Height	Area
1	9.568	BB	0.2287	1062.37451	70.92886	2.6091
2	10.859	BV	0.2607	3952.59839	232.10277	9.7074
3	11.854	VB	0.2980	2397.53223	122.70145	5.8882
4	16.625	BBA	0.4105	3.33050e4	1251.29651	81.7953

Optimization of Ligand

La(OTf)$_3$ (10 mol%), PyBim-1 (12 mol%), N_2, 25 °C, Et_2O, 48 h

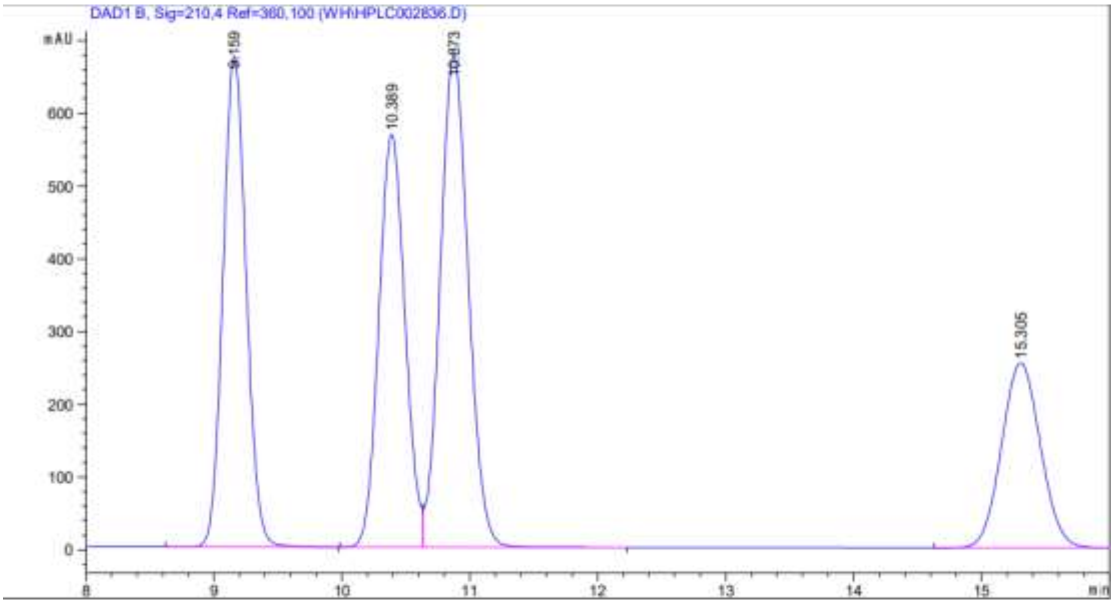

Peak	Ret. Time	Type	Width	Area	Height	Area
1	9.159	BB	0.1969	8494.58594	674.79004	26.3237
2	10.389	BV	0.2216	8054.78271	567.46637	24.9608
3	10.873	VB	0.2405	1.03427e4	676.15057	32.0506
4	15.305	BB	0.3308	5377.72168	254.25807	16.6649

La(OTf)$_3$ (10 mol%), PyBox-1 (12 mol%), N_2, 25 °C, Et_2O, 48 h

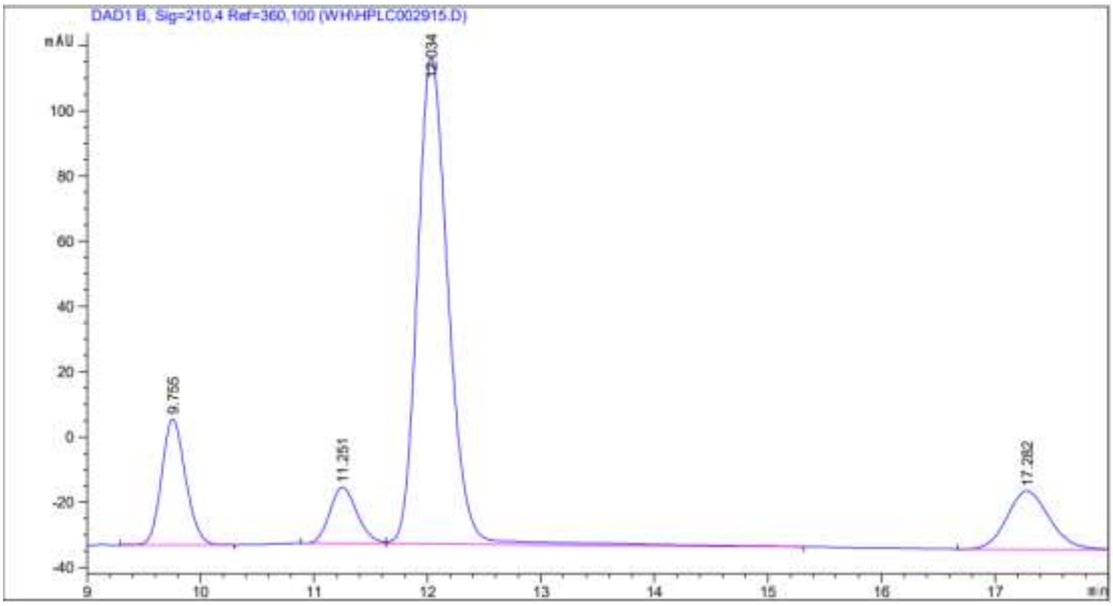

Peak	Ret. Time	Type	Width	Area	Height	Area
1	9.755	BB	0.2244	564.81989	38.66180	13.5416
2	11.251	BV	0.2609	290.83237	17.23337	6.9727
3	12.034	VB	0.2922	2840.79102	149.18312	68.1082
4	17.282	BB	0.4055	474.55634	17.88794	11.3775

La(OTf)$_3$ (10 mol%), PyBox-1 (12 mol%), N_2, 25 °C, Et_2O, 48 h

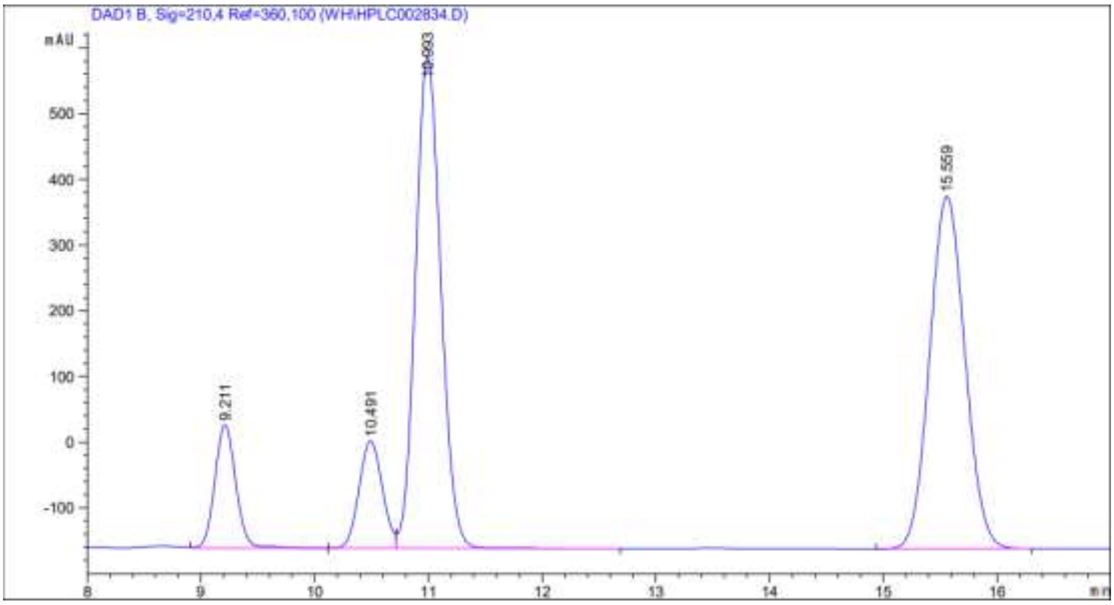

Peak #	Ret. Time [min]	Type	Width [min]	Area [mAU*s]	Height [mAU]	Area %
1	9.211	VB	0.1997	2403.19849	187.32208	8.6251
2	10.491	BV	0.2219	2326.81860	163.60168	8.3510
3	10.993	VB	0.2425	1.15814e4	748.54620	41.5656
4	15.559	BB	0.3372	1.15515e4	536.57239	41.4584

La(OTf)$_3$ (10 mol%), PyBox-2 (12 mol%), N_2, 25 °C, Et_2O, 48 h

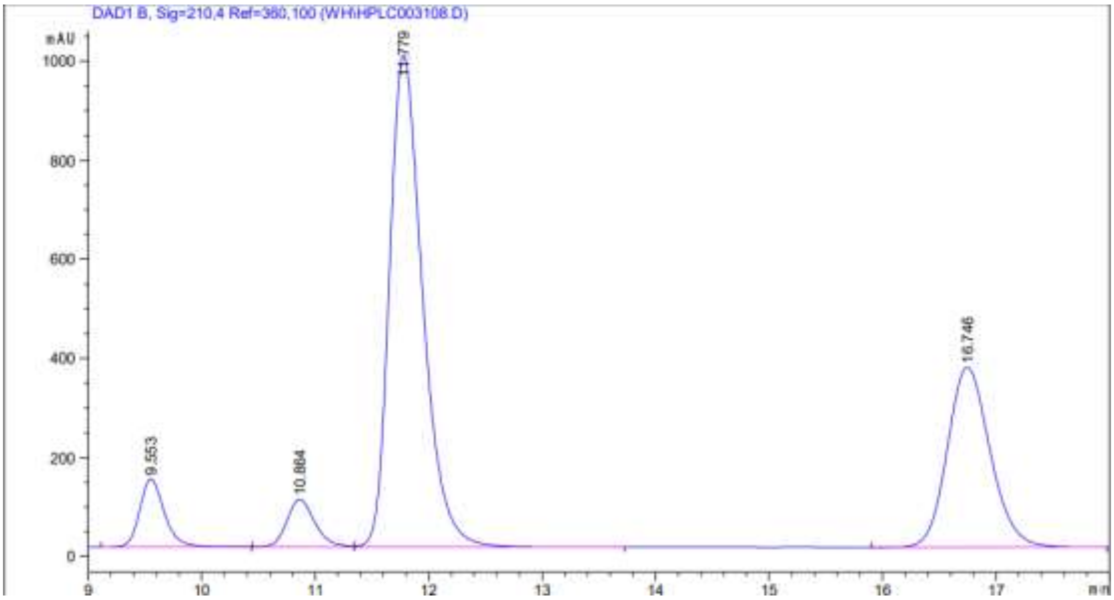

Peak	Ret. Time	Type	Width	Area	Height	Area
1	9.553	BB	0.2290	2056.35767	137.09348	6.1613
2	10.864	BV	0.2612	1628.47485	95.37479	4.8793
3	11.779	VB	0.3127	2.01383e4	992.62317	60.3388
4	16.746	BB	0.4064	9552.23730	363.70584	28.6206

La(OTf)$_3$ (10 mol%), PhenOx-1 (12 mol%), N_2, 25 °C, Et_2O, 48 h

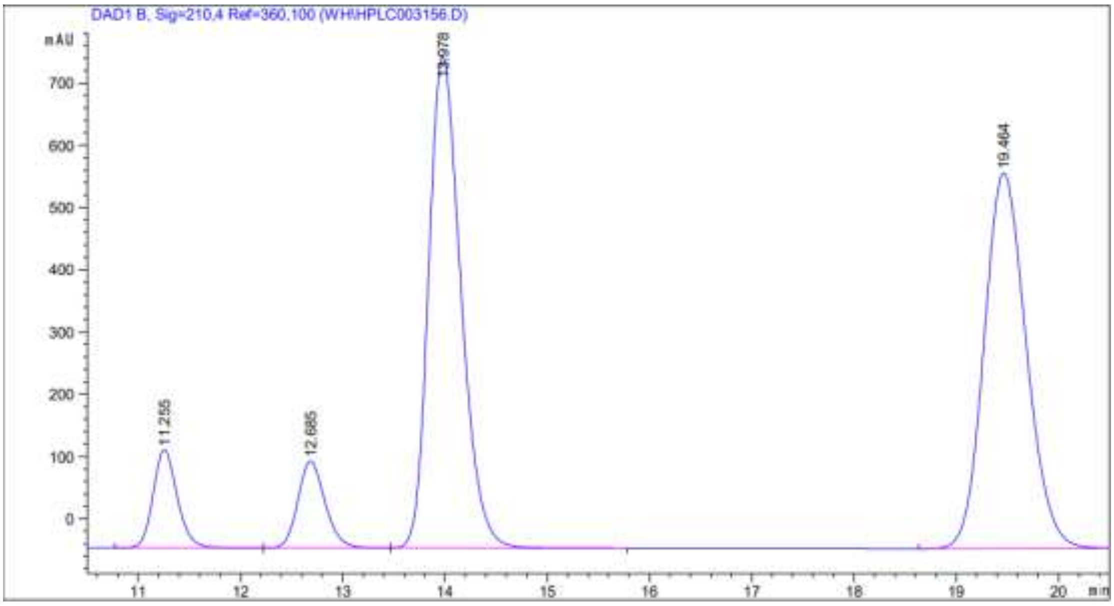

Peak	Ret. Time	Type	Width	Area	Height	Area
3	13.978	VB	0.3395	1.73010e4	790.04669	43.5110
4	19.464	BBA	0.4465	1.72919e4	603.15576	43.4882
1	11.255	BB	0.2537	2588.86133	157.57431	6.5109
2	12.685	BV	0.2841	2580.55640	139.30196	6.4900

$LaCl_3$ (10 mol%), PyBox-1 (12 mol%), N_2, 25 °C, Et_2O, 48 h

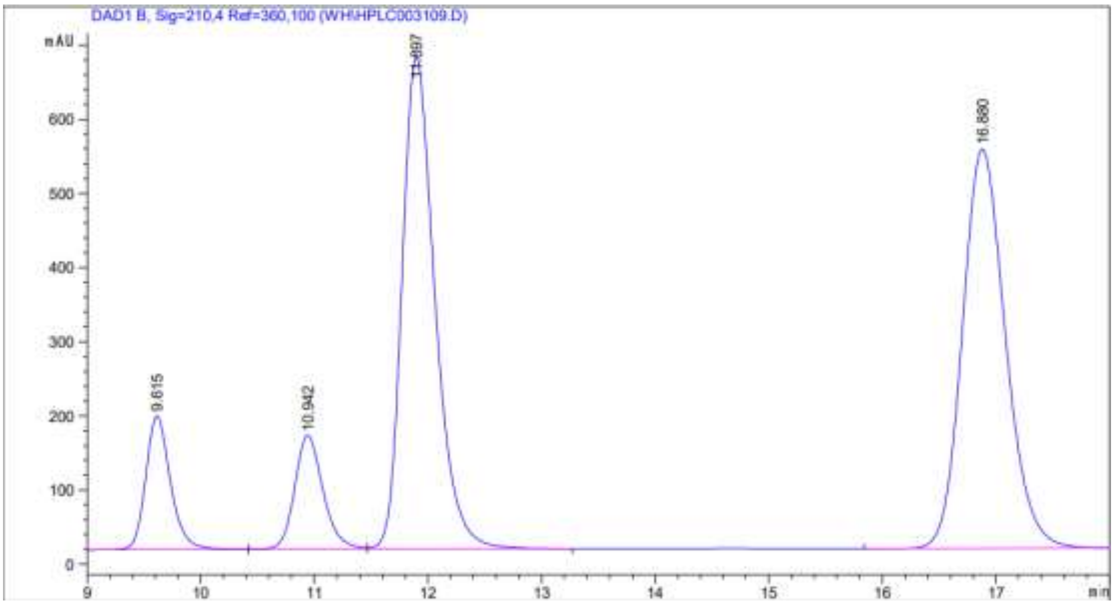

Peak	Ret. Time	Type	Width	Area	Height	Area
1	9.615	BB	0.2315	2742.81299	180.27884	8.3196
2	10.942	BV	0.2645	2666.35791	153.64720	8.0877
3	11.897	VB	0.3087	1.33404e4	663.01758	40.4646
4	16.880	BB	0.4079	1.42185e4	538.77820	43.1281

$La(BF_4)_3$ (10 mol%), PyBox-1 (12 mol%), N_2, 25 °C, Et_2O, 48 h

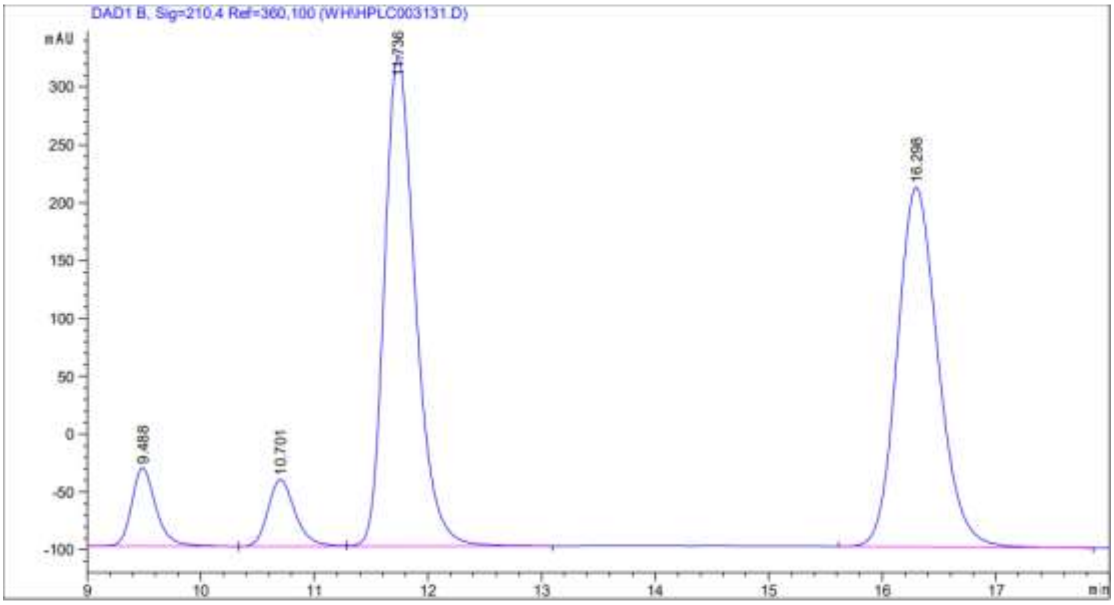

Peak	Ret. Time	Type	Width	Area	Height	Area
1	9.488	BB	0.2291	1017.59918	67.80150	5.7356
2	10.701	BV	0.2538	960.55725	57.83517	5.4141
3	11.736	VB	0.2903	8011.69727	424.33328	45.1575
4	16.298	BB	0.3872	7751.82861	310.54916	43.6927

3.3 HPLC copies of gram scale reaction.

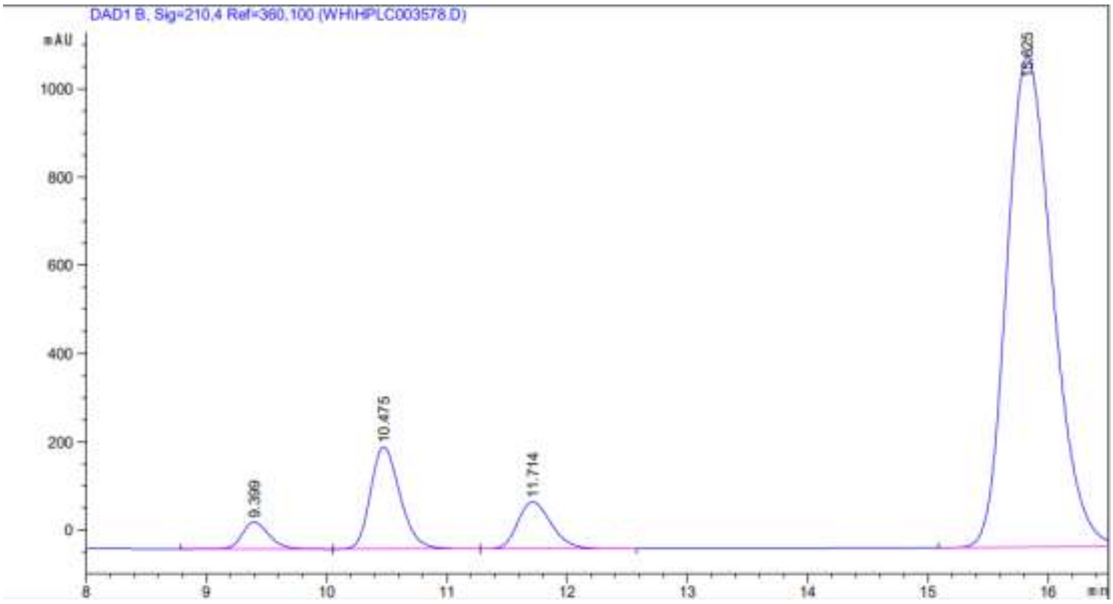

Peak	Ret. Time	Type	Width	Area	Height	Area
1	9.399	BB	0.2397	952.36023	61.16550	2.6199
2	10.475	BB	0.2624	3932.71680	231.25275	10.8188
3	11.714	BB	0.3000	2063.41577	106.50370	5.6764
4	15.825	BBA	0.4122	2.94022e4	1112.92017	80.8848
